# CDK1-mediated phosphorylation at H2B serine 6 is required for mitotic chromosome segregation

**DOI:** 10.1083/jcb.201806057

**Published:** 2019-02-14

**Authors:** Markus Seibert, Marcus Krüger, Nikolaus A. Watson, Onur Sen, John R. Daum, Johan A. Slotman, Thomas Braun, Adriaan B. Houtsmuller, Gary J. Gorbsky, Ralf Jacob, Michael Kracht, Jonathan M.G. Higgins, M. Lienhard Schmitz

**Affiliations:** 1 Institute of Biochemistry, Justus-Liebig-University, Member of the German Center for Lung Research, Giessen, Germany; 2 Institute for Genetics, Cologne Excellence Cluster on Cellular Stress Responses in Aging-Associated Diseases (CECAD), Cologne, Germany; 3 Center for Molecular Medicine (CMMC), University of Cologne, Cologne, Germany; 4 Cell Division Biology Research Group, Institute for Cell and Molecular Biosciences, Newcastle University, Newcastle upon Tyne, England, UK; 5 Cell Cycle and Cancer Biology Research Program, Oklahoma Medical Research Foundation, and Department of Cell Biology, University of Oklahoma Health Sciences Center, Oklahoma City, OK; 6 Department of Pathology, Josephine Nefkens Institute, Erasmus Optical Imaging Centre, Erasmus MC, Rotterdam, Netherlands; 7 Max Planck Institute for Heart and Lung Research, Bad Nauheim, Germany; 8 Department of Cell Biology and Cell Pathology, Philipps University of Marburg, Marburg, Germany; 9 Rudolf-Buchheim-Institute of Pharmacology, Justus-Liebig-University, Member of the German Center for Lung Research, Giessen, Germany

## Abstract

Faithful mitotic chromosome segregation is required for the maintenance of genomic stability. We discovered the phosphorylation of histone H2B at serine 6 (H2B S6ph) as a new chromatin modification site and found that this modification occurs during the early mitotic phases at inner centromeres and pericentromeric heterochromatin. This modification is directly mediated by cyclin B1–associated CDK1, and indirectly by Aurora B, and is antagonized by PP1-mediated dephosphorylation. H2B S6ph impairs chromatin binding of the histone chaperone SET (I2PP2A), which is important for mitotic fidelity. Injection of phosphorylation-specific H2B S6 antibodies in mitotic cells caused anaphase defects with impaired chromosome segregation and incomplete cytokinesis. As H2B S6ph is important for faithful chromosome separation, this modification may contribute to the prevention chromosomal instability and aneuploidy which frequently occur in cancer cells.

## Abstract (precis)

Seibert et al. identify a new phosphorylation event of histone H2B at serine 6 during mitosis with CDK1 and PP1 as writers and erasers of this modification. This phosphorylation contributes to chromatin dislocation of the histone chaperone SET during mitosis and is required for mitotic chromosome segregation.

## Introduction

The precise temporal and spatial control of events during cell cycle progression depends on many enzymatic activities. This is well exemplified by the process of mitosis, which is characterized by a widespread increase in phosphorylation site occupancy, as revealed by quantitative phosphoproteomics (Olsen et al., 2010). The spatiotemporal coordination of the mitotic phases requires the restriction of the activity time and subcellular localization of mitotic kinases and phosphatases. Mitotic phosphorylations prominently occur on proteins controlling metabolic processes, mitosis regulators, and on kinetochore proteins and histones (Johnson, 2011; Funabiki and Wynne, 2013; Swaffer et al., 2016). Histone modifications help orchestrate chromosome congression and segregation, but also may participate in the down-regulation or reestablishment of gene expression (Wang and Higgins, 2013).

Kinases controlling mitosis include the master regulator CDK1-cyclin B1, which phosphorylates multiple substrates, including linker histone H1, and is indispensable for early mitotic events (Langan et al., 1989; Domingo-Sananes et al., 2011). Another important mitotic kinase is Aurora B which, together with INCENP, Borealin, and Survivin, forms the chromosomal passenger complex (CPC). This complex is found at several locations during mitosis, including centromeres, where it monitors bi-orientation of chromosomes and is required for the correction of spindle-kinetochore attachment errors and spindle assembly checkpoint signaling (Carmena et al., 2012). Aurora B also phosphorylates histones H3 and H1.4, resulting in displacement of heterochromatin protein 1 from chromosomes in mitosis (Van Hooser et al., 1998; Goto et al., 2002; Fischle et al., 2005; Hirota et al., 2005; Hergeth et al., 2011). While Aurora B–mediated histone phosphorylation occurs all along the chromosomes, other histone phosphorylation marks show more distinct localization at centromeres or pericentromeric heterochromatin. For example, mitotic phosphorylation of H3 T3 and H3.3 S31 is enriched at inner centromeres, the chromatin regions between the kinetochore-bound centromeres that are enriched in di- and trimethylated H3 lysine 9 (H3 K9me2/3; Goto et al., 2002; Dai et al., 2005; Hake et al., 2005; Yamagishi et al., 2010; Müller and Almouzni, 2017).

Mitotic kinases are counterregulated by phosphatases such as PP1 and PP2A, which occur in complexes consisting of catalytic subunits in association with different regulatory and targeting factors (Brautigan, 2013; Grallert et al., 2015). These phosphatases are also controlled by kinase signaling, as, for example, Aurora B and CDK1-cyclin B1 can inhibit PP1 action in early mitosis (Liu et al., 2010; Vagnarelli et al., 2011; Qian et al., 2013, 2015; Nasa et al., 2018). The phosphatase PP2A can be negatively regulated by the nuclear oncoprotein SET (also known as I2PP2A), a mechanism with importance for sister chromatid resolution (Li et al., 1996; Chambon et al., 2013; Moshkin et al., 2013; Qi et al., 2013).

However, the SET protein has been associated with several different functions, including a role as a component of the inhibitor of acetyltransferases complex (Seo et al., 2001) and a function as a histone chaperone with the ability to bind H2B and H3 in vitro (Muto et al., 2007; Karetsou et al., 2009). SET also contributes to the removal of phosphorylated histone H1 and Shugoshins from chromosomes during cell division (Krishnan et al., 2017). Shugoshin-like (Sgo) proteins collaborate with PP2A to prevent untimely removal of centromeric cohesin, a ring-shaped multi-protein complex containing the Rad21 protein that holds sister chromatids together until anaphase (Nasmyth and Haering, 2009).

Here, we report the identification of H2B S6 phosphorylation as a novel histone tail modification that occurs between prophase and anaphase in mitosis and is enriched at the inner centromeres. Its highly regulated spatial and temporal occurrence is controlled by an interplay between cyclin B1–associated CDK1 activity and PP1 phosphatases. This phosphorylation weakens the association of SET with histones and perturbation of H2B S6ph by injection of phospho-specific antibodies impairs the fidelity of chromosome segregation.

## Results

### H2B S6ph occurs in early mitosis

**Figure 1.**
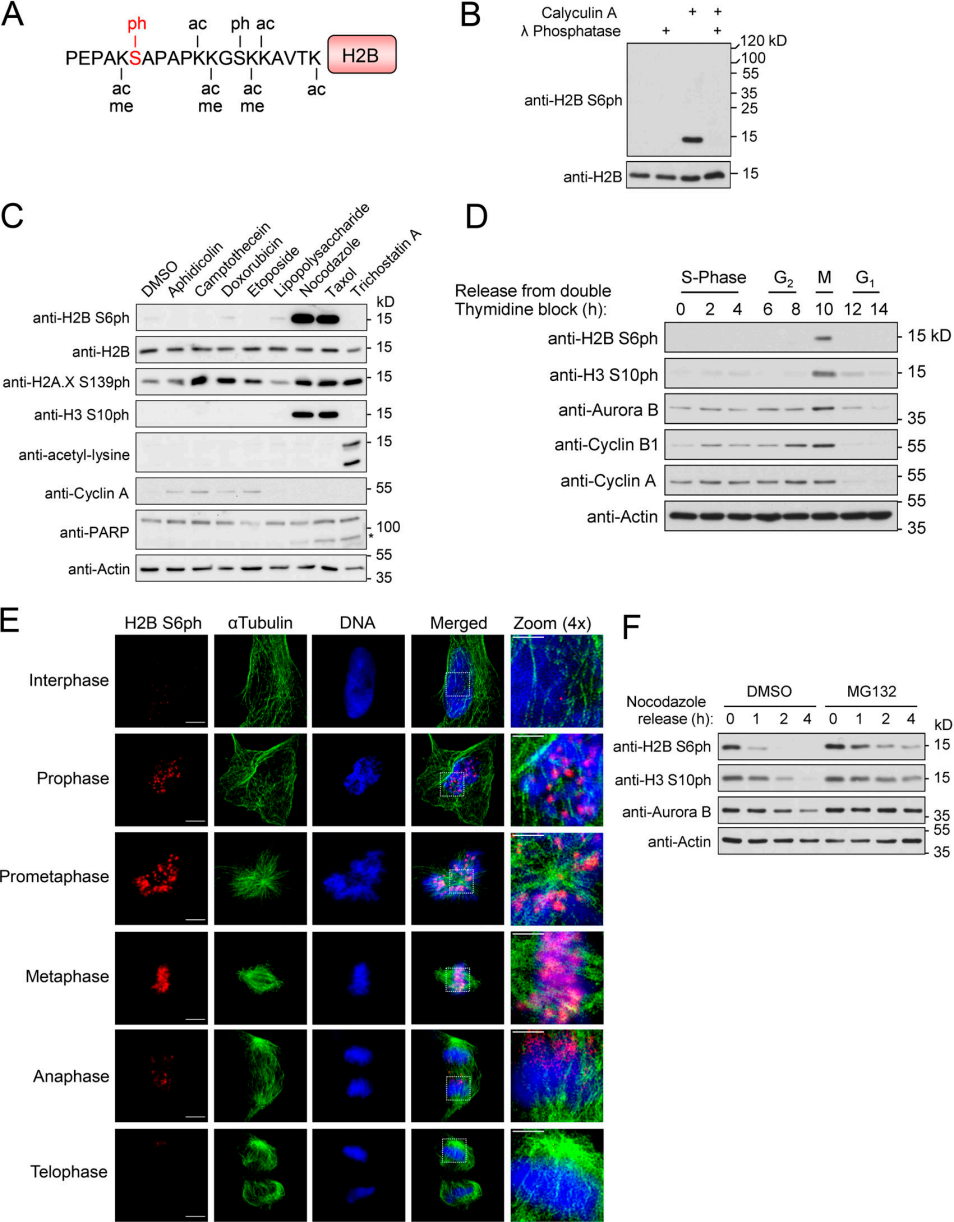
**Identification of mitotic H2B S6ph. (A)** The sites of the H2B N-terminal tail known to be modified by phosphorylation (ph), acetylation (ac), methylation (me), or ubiquination (ub) are shown in black; the novel phosphorylation of S6 is marked in red. **(B)** 293T cells were treated with 50 nM calyculin A for 30 min, and lysates were incubated with λ phosphatase as shown. Equal amounts of protein contained in cell lysates were analyzed by Western blotting for the occurrence and phosphorylation of H2B with specific antibodies. The positions of molecular weight markers are indicated. **(C)** HeLa cells were treated with the indicated reagents or the DMSO vehicle control for 16 h and lysed with SDS sample buffer. Lysates were analyzed by SDS-PAGE and Western blotting using the indicated antibodies; the cleaved form of the caspase substrate PARP is indicated by an asterisk. **(D)** HeLa cells were synchronized by a double thymidine block and released into S phase. Cells were lysed at the indicated time points with SDS sample buffer. Lysates were analyzed by SDS-PAGE and Western blotting using the indicated antibodies. **(E)** Diploid RPE-1 cells were fixed, and immunostaining was performed with antibodies against H2B S6ph and α-tubulin. DNA was visualized with Hoechst 33342. Bars: 5 µm (main); 2 µm (magnification). 3D-SIM was used to reveal the occurrence and localization of the stained proteins during the indicated mitotic phases; the boxed areas are displayed in 4× magnification. **(F)** HeLa cells were synchronized by treatment with nocodazole for 16 h and released in the presence of 10 µM MG132 or DMSO. Cells were lysed at the indicated time points with SDS sample buffer and analyzed by SDS-PAGE and Western blotting using the indicated antibodies.

During a search for new histone phosphorylation sites by mass spectrometry (MS), we discovered modification of H2B S6 by in vitro kinase assays. The sequence context of this novel site is conserved in vertebrates and surrounded by many further posttranslational modifications at the N-terminal H2B tail (Fig. 1 A; Cheung et al., 2003; Beck et al., 2006; Tan et al., 2011; Cao et al., 2013; Huang et al., 2014). Phospho-specific antibodies against phosphorylated H2B S6 (anti-H2B S6ph) were raised, affinity purified, and tested by Western blotting. Extracts from cells treated with the PP1/PP2A inhibitor calyculin A allowed the detection of H2B S6ph, while no signal was detected in extracts from untreated cells. The signal was absent in extracts that were incubated with λ phosphatase, indicating a phosphorylation-specific recognition of H2B S6 by antibodies (Fig. 1 B). Furthermore, a phosphorylation-deficient H2B S6A mutant protein was not recognized, while a phospho-mimetic H2B S6E mutant was detected, showing the specificity of the antibody (Fig. S1 A). A further characterization of the antibodies by ELISA experiments showed that anti-H2B S6ph antibodies specifically detect H2B S6ph irrespective of mono-methylation or acetylation at the adjacent K5 (Fig. S1 B).

To identify signals leading to H2B S6ph, cells were exposed to a variety of different stimuli representing DNA damage, inflammatory conditions, or microtubule poisons. Among these agents, only treatment with nocodazole or taxol resulted in a strong increase of H2B S6ph (Fig. 1 C). As nocodazole or taxol interfere with the formation of mitotic spindles, we then tested whether H2B S6ph occurs in mitosis.

Extracts from synchronized HeLa cells showed that H2B S6ph occurred during mitosis and disappeared coincident with the decay of cyclins A and B1, Aurora B, and H3 S10ph (Fig. 1 D). Super-resolution 3D structured illumination microscopy (3D-SIM) was used to investigate the occurrence and localization of mitotic H2B S6ph and α-tubulin in diploid RPE-1 cells. H2B S6ph was not detectable in interphase cells, but increased from prophase to metaphase and decreased rapidly during anaphase (Fig. 1 E). The exact timing and unidirectional mitotic progression involves the ubiquitin-/proteasome-mediated degradation of key regulators such as Aurora B and cyclin B1 (Wieser and Pines, 2015). To test whether ubiquitin-/proteasome-dependent processes affect the kinetics of mitotic H2B S6ph, HeLa cells were treated with nocodazole, and prometaphase-arrested cells were collected by mitotic shake-off and released in the absence or presence of the proteasome inhibitor MG132. In contrast to H3 S10 phosphorylation, H2B S6ph already strongly decreased within 1 h. In MG132-treated cells phosphorylated H2B S6 and H3 S10 still occurred 4 h after release, although H2B S6ph declined slightly faster than H3 S10ph (Fig. 1 F).

### H2B S6 phosphorylation is found on pericentromeric heterochromatin and highly enriched at inner centromeres

**Figure 2.**
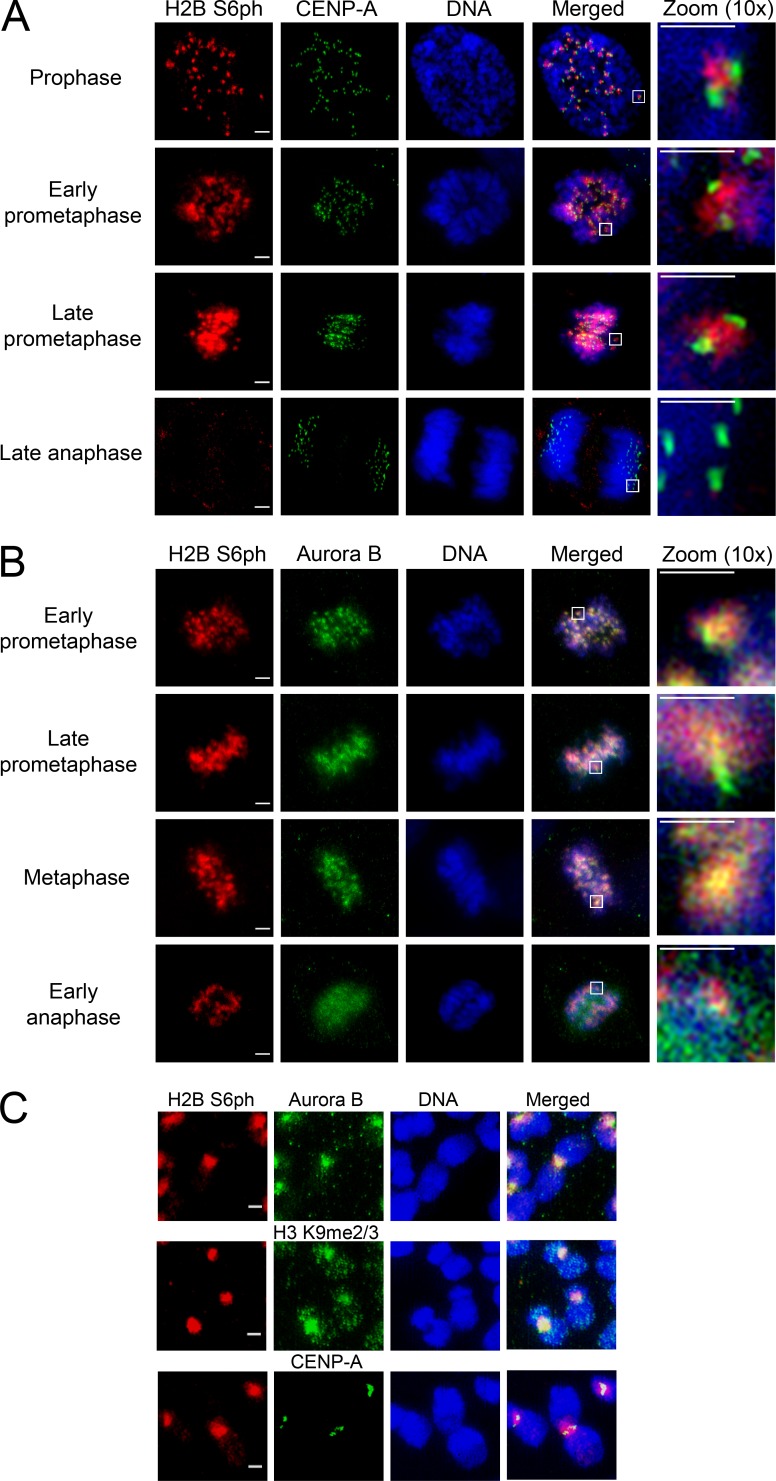
**Analysis of chromosomal localization of H2B S6ph by 3D-SIM. (A)** RPE-1 cells were stained for H2B S6ph and CENP-A localization with specific antibodies, DNA was revealed by Hoechst dye. Different mitotic phases are shown. Bars: 2 µm (main); 1 µm (magnification). **(B)** The experiment was done as in A, with the difference that cells were stained for H2B S6ph and Aurora B. The boxed areas in A and B are displayed in 10× magnification. Bars: 2 µm (main); 1 µm (magnification). **(C)** RPE-1 cells were arrested at G_2_/M transition with the CDK1 inhibitor RO 3306 for 16 h and released into mitosis for 1 h in the presence of nocodazole. Chromosome spreads were produced for immunostaining against H2B S6ph, Aurora B, H3 K9 me2/3, or CENP-A as shown. Pictures show chromosome spreads of representative cells. Bars, 1 µm.

Immunofluorescence staining did not only reveal restriction of H2B S6ph to early mitosis, but also showed localized phosphorylation at distinct chromosomal regions. To investigate whether H2B S6ph occurs at centromeres, RPE-1 cells were stained with antibodies against phosphorylated H2B S6 and the histone variant CENP-A, a marker of the centromere (Foltz et al., 2006). Phosphorylated H2B S6 and CENP-A exhibited largely nonoverlapping but proximal localization during the various mitotic phases (Fig. 2 A) and a clear colocalization with the CPC subunit Aurora B (Fig. 2 B), which is known to be localized to inner centromeres until anaphase (Wang et al., 2011).

**Figure 3.**
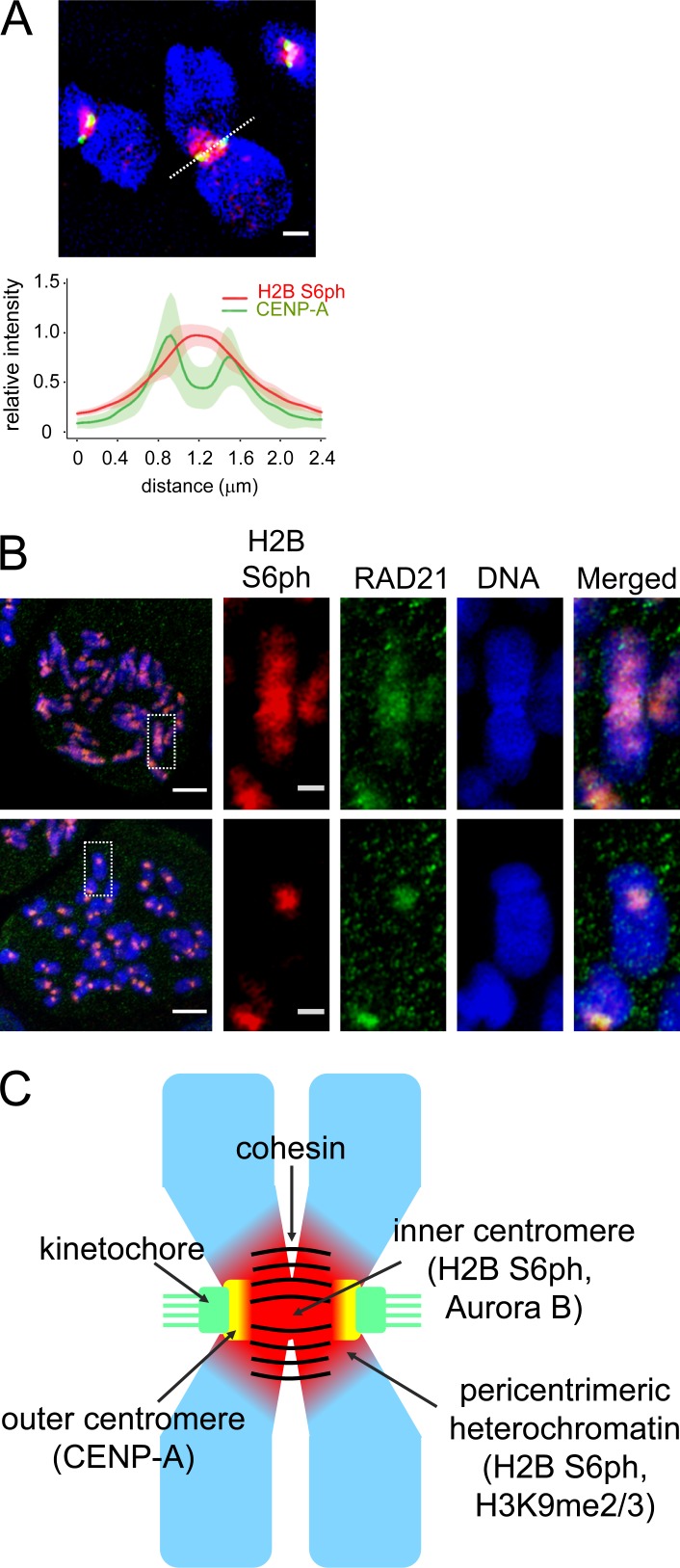
**Analysis of inner centromeric H2B S6ph localization. (A)** Synchronized RPE-1 cells were used to produce chromosome spreads, which were stained with antibodies specific for H2B S6ph (red) and CENP-A (green). Bar, 1 µm. The images were quantitatively analyzed with ImageJ for the distribution of H2B S6ph and CENP-A along the centromere cross section, as shown by the dotted line (top). The maximum of fluorescence intensity was set as 1, and shaded areas indicate standard deviations (*n* = 8 cells/368 centromere pairs). **(B)** The experiment was done as in Fig. 2 C, with the difference that chromosomes were stained for H2B S6ph and the cohesin component RAD21. Representative examples are shown; the boxed area is displayed in the bottom panel in 4× magnification. Bars: 5 µm (main); 1 µm (magnification). **(C)** Schematic representation of the chromosomal localization of H2B S6ph on mitotic chromatids.

To map the exact localization of phosphorylated H2B S6, chromosome spreads were prepared from mitotic RPE-1 cells and stained with antibodies against phosphorylated H2B S6 and different proteins and chromatin markers (Fig. 2 C). Consistently, the localization of H2B S6ph matched with that of Aurora B. Phosphorylated H2B S6 showed overlapping costaining patterns with H3 K9me2/3 (Fig. 2 C), a marker of pericentromeric heterochromatin (McManus et al., 2006). Also, the quantitative analysis of H2B S6ph and CENP-A localization revealed the strongest enrichment of this phosphorylation at the inner centromere being between two CENP-A spots (Fig. 3 A).

During these experiments, we noted differential localization of H2B S6ph depending on the resolution status of the chromatid arms. Chromosomes from prophase cells, which displayed cohesin along the chromatids, showed a more extended H2B S6ph, while prometaphase chromosomes showed cohesin RAD21 and H2B S6ph only at the inner centromeres and pericentromeric heterochromatin (Fig. 3 B), as schematically summarized in Fig. 3 C.

**Figure 4.**
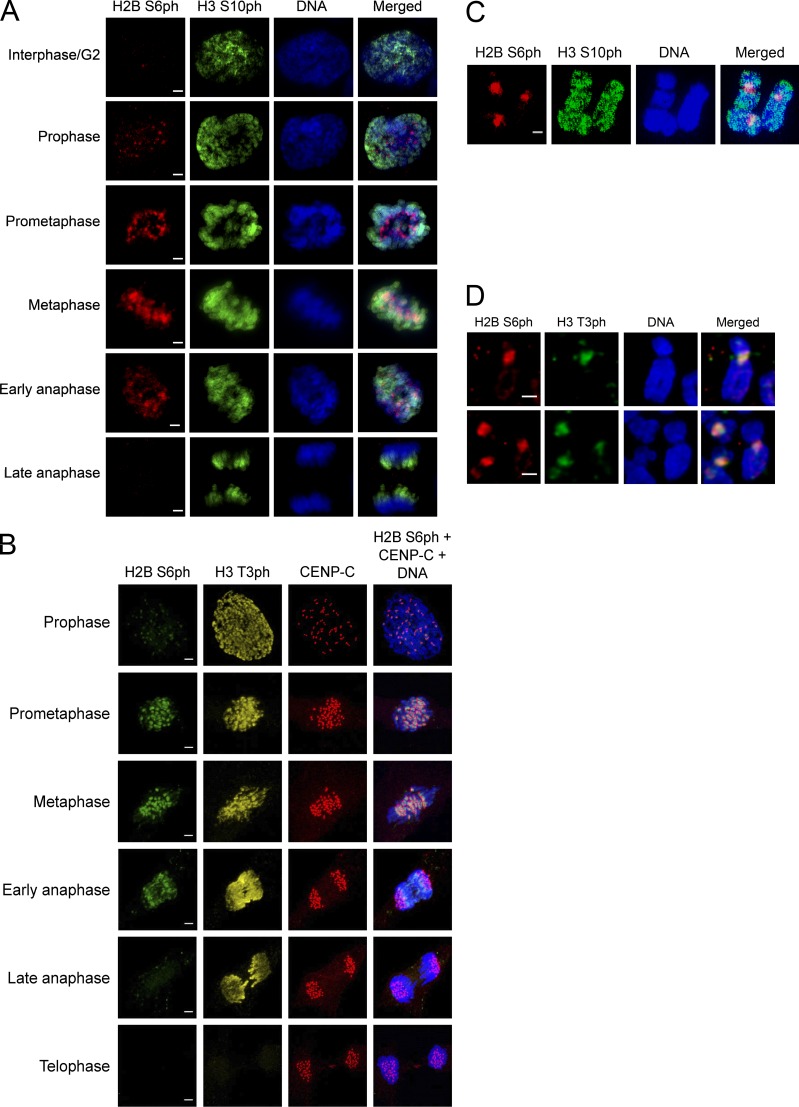
**Comparative analysis of mitotic histone phosphorylations. (A)** RPE-1 cells were stained for H2B S6ph and H3 S10ph in various phases of mitosis with specific antibodies; DNA was revealed by Hoechst dye. Different mitotic phases are shown. Bars, 2 µm. **(B)** RPE-1 cells were stained for H2B S6ph, H3 T3ph, and the kinetochore marker protein CENP-C during various mitotic phases. Bars, 2 µm. **(C)** Chromosome spreads were produced from nocodazole-arrested cells, which were stained for phosphorylation of H2B S6 and H3 S10. Bars, 1 µm. **(D)** RPE-1 cells were treated for 2 h with 2 µM nocodazole, and chromosome spreads were produced to stain for H2B S6ph and H3 T3ph. Bars, 1 µm.

Further immunofluorescence experiments were performed to compare the phosphorylation kinetics of H2B S6 with that of H3 S10 (Fig. 4 A) and H3 T3 (Fig. 4 B). H3 S10 phosphorylation differed from H2B S6 modification, as it already increased during late G_2_ and decreased only in late anaphase, consistent with published literature (Hendzel et al., 1997). Similarly, H3 T3ph was detectable in prophase earlier than H2B S6ph and persisted longer in anaphase (Fig. 4 B; Dai et al., 2005). The analysis of chromosome spreads showed remarkable differences in chromosomal localization, as phosphorylated H3 S10 was found all along the chromosome arms, while H2B S6 was highly enriched at inner centromeres (Fig. 4 C). In contrast, the chromosomal localization of phosphorylated H3 T3 and H2B S6 showed strong overlap at the inner centromeres (Fig. 4 D), though H3 T3ph was more visible than H2B S6ph on chromosome arms in cell preparations that were not subject to nocodazole arrest and spreading (Fig. 4 B).

### Identification of cyclin B1–associated CDK1 as H2B S6 kinase

**Figure 5.**
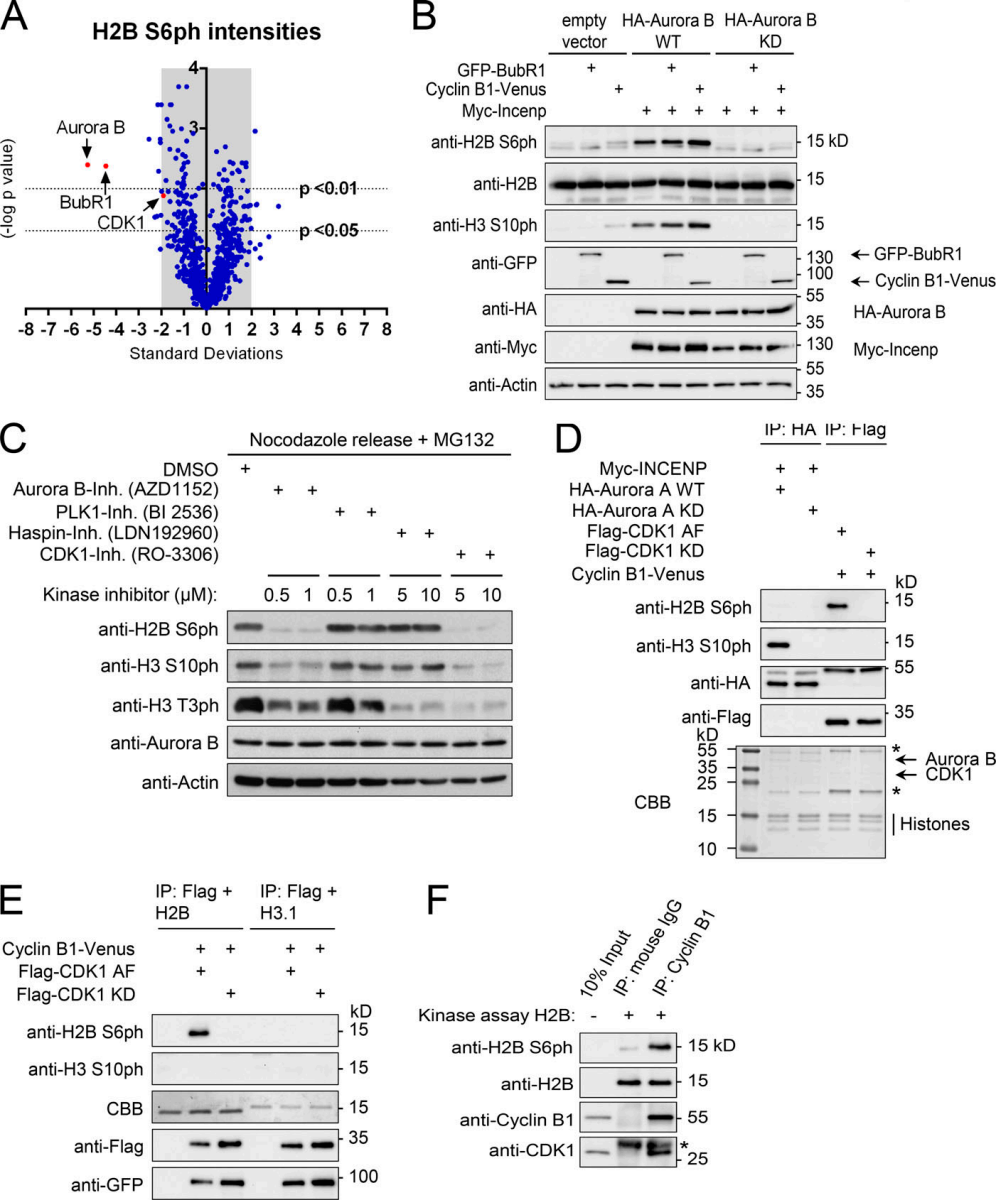
**Analysis of H2B S6 phosphorylation networks. (A)** HeLa cells were transfected with a Dharmacon siRNA library against kinase and kinase-related genes. After 42 h, cells were treated with 200 nM nocodazole for 6 h and prepared for immunofluorescence staining against phosphorylated H2B S6. Phosphorylation intensities were quantified using a wide field Nikon microscope (Eclipse Ti-E) and high-content analysis software. Volcano plot shows standard deviations of average scores from mean H2B S6 phosphorylation intensity (x axis) and their statistical significance (P values; y axis). Statistical analysis was performed using GraphPad Prism. Aurora B, BubR1, and CDK1 are shown in red. **(B)** GFP-BubR1 and cyclin B1-Venus were expressed in 293T cells in the absence or presence of Myc-INCENP and HA-Aurora B WT or kinase dead (KD). Cells were lysed with SDS sample buffer and analyzed by SDS-PAGE and Western blotting using the indicated antibodies. **(C)** HeLa cells were arrested at prometaphase with nocodazole for 16 h and released in the presence of MG132 together with various concentrations of the indicated kinase inhibitors. Cells were lysed with SDS sample buffer and further analyzed by immunoblotting as shown. **(D)** Different plasmids encoding the indicated proteins, including constitutively active (AF) or kinase dead (KD) kinases, were expressed in 293T cells as shown. Aurora B was immunoprecipitated with HA antibodies and CDK1 with anti-Flag M2 affinity gel for a subsequent in vitro kinase assay with recombinant histone octamers as substrate. Samples were analyzed by SDS-PAGE, CBB staining, and immunoblotting using the indicated antibodies; unspecific IgG bands are indicated by asterisks. **(E)** Cyclin B1-Venus and Flag-tagged CDK1 forms were coexpressed in 293T cells. Cells were lysed with NP-40 buffer, and CDK1 was immunoprecipitated with anti-Flag M2 affinity gel for in vitro kinase assay using H2B and H3.1 as substrate proteins. Samples were analyzed by SDS-PAGE, CBB staining, and Western blotting using the indicated antibodies. **(F)** Lysates from nocodazole-arrested HeLa cells were used for IP of endogenous cyclin B1, followed by in vitro kinase assays to test phosphorylation of recombinant H2B. Specific antibodies were used to reveal H2B S6ph and precipitation of cyclin B1 and the associated CDK1. The asterisk indicates the position of the light chain from the precipitating antibodies.

To identify the H2B S6 kinase by an unbiased approach, we performed an siRNA screen. HeLa cells were transfected with a Dharmacon siRNA library against kinases and kinase-related proteins. Cells were treated with nocodazole and stained with anti-H2B S6ph antibodies and, as a control, also with anti-H3 T3ph antibodies. The known kinase Haspin was the top candidate for H3 T3ph, showing the functionality of this screening approach (data not shown). High ranking candidates for H2B S6ph were Aurora B, BubR1, and CDK1 (Fig. 5 A and Table S1). As this screening approach can detect direct and also indirect kinases for H2B S6ph, we performed follow up studies. Overexpression of cyclin B1 weakly increased H2B S6ph, while coexpression of Aurora B and INCENP strongly triggered this modification (Fig. 5 B). No induction of H2B S6ph was seen after expression of the checkpoint protein BubR1 (Fig. 5 B). To test the contribution of Aurora B, CDK1, and further mitotic kinases for H2B S6ph by a pharmacological approach, nocodazole-arrested HeLa cells were collected by mitotic shake-off and released in the presence of MG132 (to preserve H2B S6ph) and different kinase inhibitors. Phosphorylation was absent in cells treated with the Aurora B inhibitor AZD1152 or the CDK1 inhibitor RO-3306, while inhibition of Plk1 or Haspin did not affect H2B S6ph (Fig. 5 C and Fig. S2).

**Figure 6.**
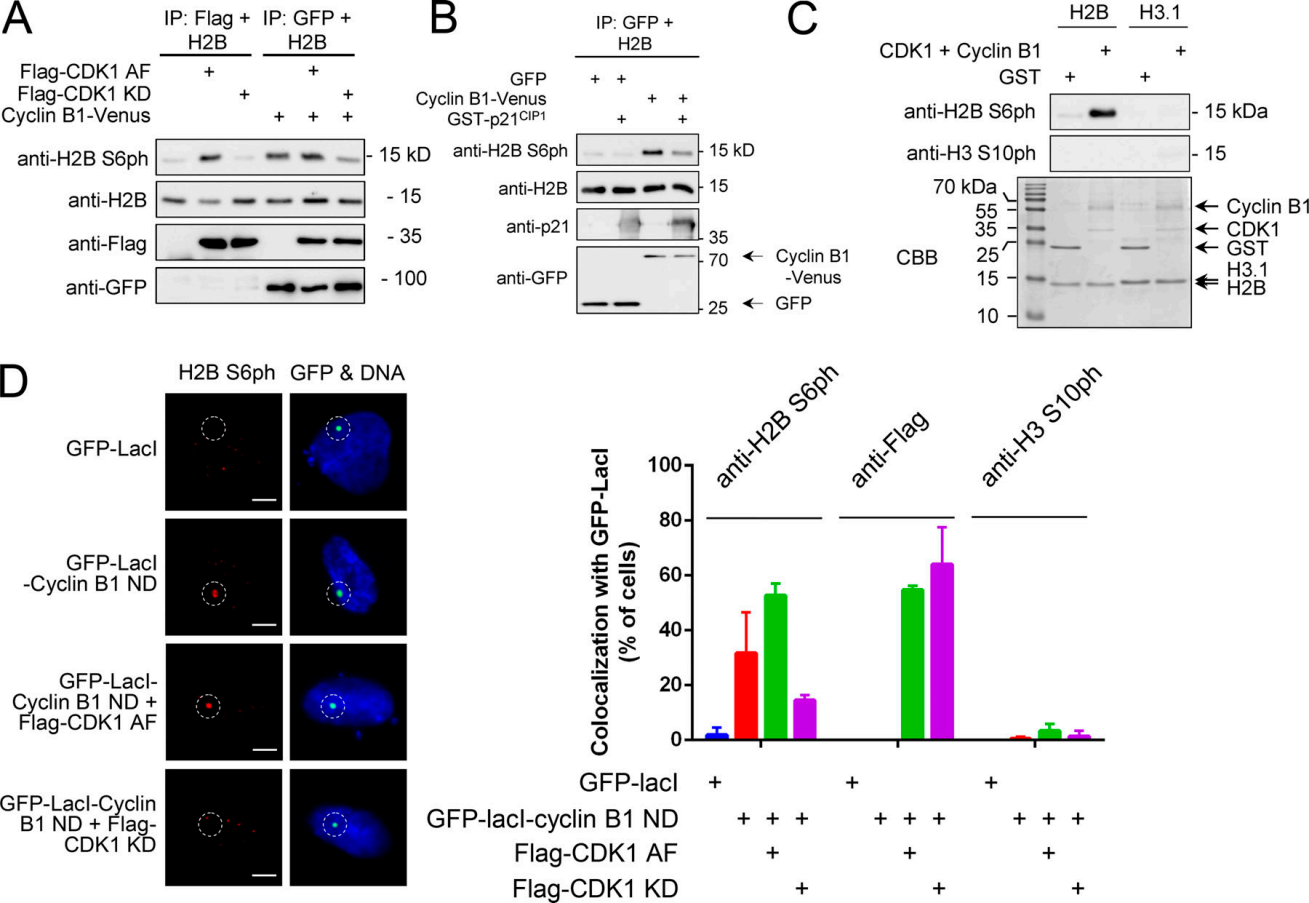
**Identification of CDK1 as a direct H2B S6 kinase. (A)** Flag-tagged CDK1 forms were expressed with cyclin B1-Venus in 293T cells. CDK1 was immunoprecipitated with anti-Flag M2 affinity gel and cyclin B1 with GFP-Trap beads for a subsequent in vitro kinase assay with recombinant H2B. Samples were analyzed by SDS-PAGE and immunoblotting using the indicated antibodies. **(B)** 293T cells were transfected to express cyclin B1-Venus or GFP as shown. Cells were lysed with NP-40 buffer and GFP, and cyclin B1-Venus were immunoprecipitated with the GFP-Trap for a subsequent in vitro kinase assay using recombinant H2B as a substrate protein. The reactions were incubated in the presence or absence of 1 µg recombinant p21^CIP1^ protein (Sigma) as shown, H2B S6ph was analyzed by immunoblotting. **(C)** 1 µg of recombinant and purified CDK1-cyclin B1 (Thermo Fisher) or 2 µg GST control (Sigma) were incubated with recombinant H2B (2 µg) in the presence of ATP. The reaction was analyzed for histone phosphorylation by Western blotting (top) and protein integrity by SDS-PAGE and CBB staining. **(D)** U2OS F4 2B8 cells with an array of lacO sites integrated close to the centromere of chromosome 2 were transfected with GFP-lacI alone or a nondegradable form of cyclin B1 (cyclin ND) fused to GFP-lacI and Flag-tagged forms of CDK1 AF and CDK1 KD. After 24 h, cells expressing moderate amounts of the GFP-lacI fusion proteins were fixed for immunofluorescence and analyzed for phosphorylation of H2B S6 and the H3 S10 control as well as for Flag-CDK1 localization. The left part shows representative results from the immunofluorescence studies. The right part displays the ratio of cells showing colocalization of GFP-lacI-cyclin B1 and H2B S6ph, Flag-CDK1, or H3 S10ph stained with the indicated antibodies. *n* > 100 cells from two independent experiments were analyzed for each condition; error bars show standard deviations between the two biological replicates. Bars, 5 µm.

Since both inhibitors also showed indirect effects on other modifications (e.g., Haspin-mediated H3 T3ph), we performed in vitro kinase experiments to test if one of the kinases can directly phosphorylate H2B S6. Aurora B was coexpressed together with INCENP in 293T cells and immunoprecipitated for in vitro kinase assays using recombinant histone octamers as substrate. Although Aurora B was able to phosphorylate its known substrate, H3 S10, no phosphorylation-specific signal could be detected for H2B S6, arguing against Aurora B as H2B S6 kinase. In contrast, immunoprecipitated constitutively active CDK1 (T14A/Y15F), but not kinase-inactive CDK1 (D145N), efficiently phosphorylated H2B S6 in histone octamers (Fig. 5 D) and showed in vitro kinase activity toward S6 in recombinant H2B, but not for S10 in recombinant H3 (Fig. 5 E). Furthermore, endogenous cyclin B1 from mitotic HeLa cells was found to be associated with CDK1 activity leading to H2B S6 in vitro phosphorylation (Fig. 5 F). Further in vitro kinase assays using immunoprecipitated cyclin B1-Venus showed that coexpression of constitutively active CDK1 augmented cyclin B1–associated kinase activity, while the kinase-inactive CDK1 diminished this phosphorylation (Fig. 6 A). The cyclin B1–associated in vitro kinase activity was also diminished after inhibition of CDKs with the small molecule inhibitor, Roscovitine (data not shown), or the recombinant p21^CIP1^ protein, an inhibitor of CDK activity (Fig. 6 B; Satyanarayana et al., 2008). To test H2B S6ph in a fully reconstituted system, we performed in vitro phosphorylation assays using recombinant and purified CDK1 together with its cognate cyclin B1. These experiments revealed strong H2B S6 phosphorylation by CDK1-cyclin B1, while no H3 S10ph was detected in controls (Fig. 6 C).

We then used a tethering system to investigate the contribution of CDK1 and cyclin B1 for H2B S6ph in vivo, while minimizing indirect and confounding effects on mitosis, which can be caused by overexpression of constitutively active CDK1 (Szmyd et al., 2018). U2OS F4 2B8 cells containing an array of lacO sites stably integrated close to the centromere of chromosome 2 (Jegou et al., 2009) were transfected to express a nondegradable form of cyclin B1 (R42A/L45A; Vázquez-Novelle et al., 2014) fused with GFP and the lacO-binding lacI protein in the absence or presence of CDK1. Expression of nondegradable cyclin B1 alone caused the cell cycle–independent occurrence of H2B S6ph at the lacO repeats (Fig. 6 D). While coexpression of constitutively active CDK1 augmented focal H2B S6ph, the expression of kinase-inactive CDK1 reduced this phosphorylation (Fig. 6 D). In summary, these different gain-of-function and loss-of-function experiments show the direct and prominent contribution of CDK1-cyclin B1 for H2B S6ph.

### The centromeric localization of H2B S6ph is controlled by mitotic phosphatases

**Figure 7.**
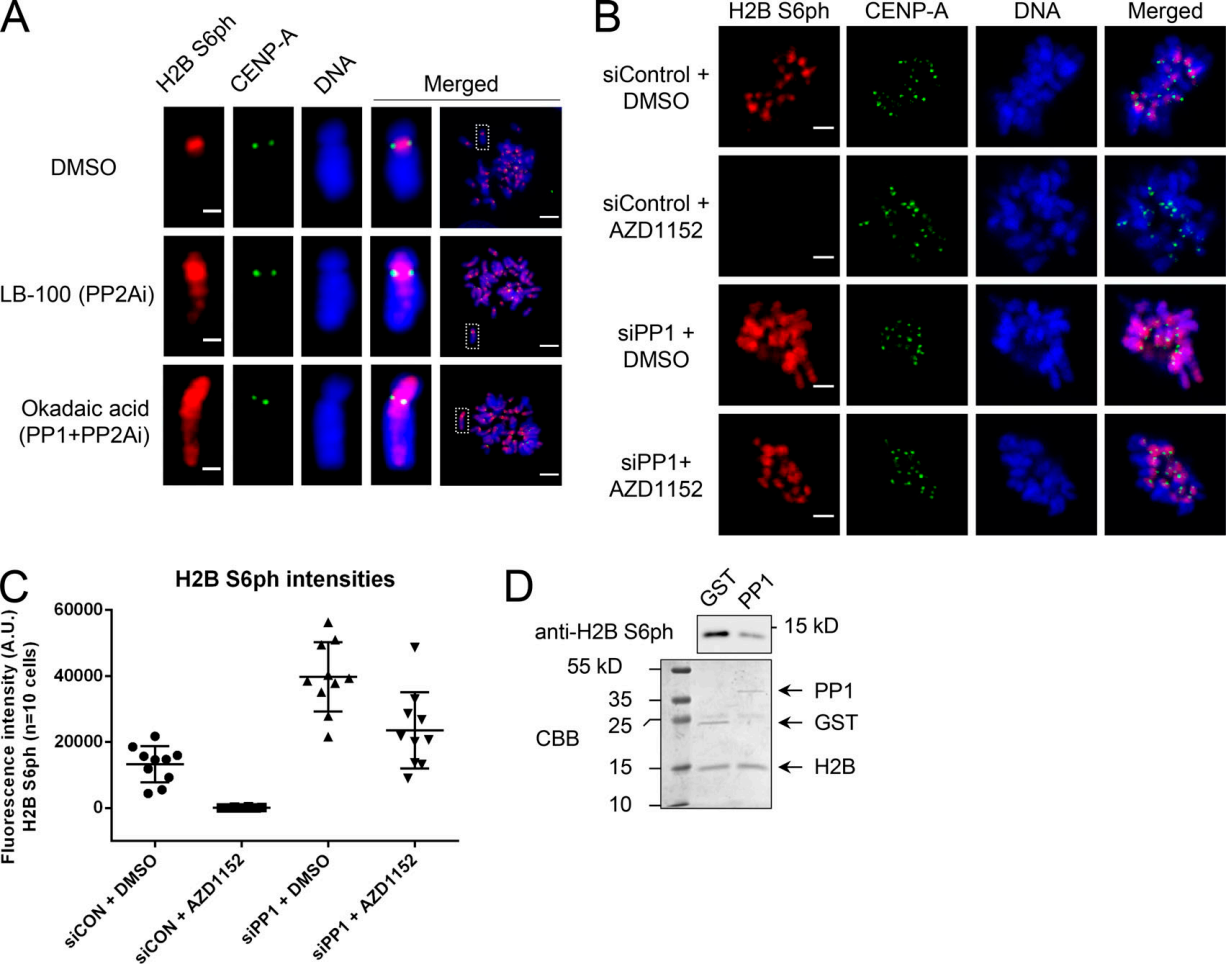
**Identification of H2B S6ph phosphatases. (A)** RPE-1 cells were arrested for 16 h with RO-3306 and then released for 1 h in the presence of nocodazole and okadaic acid (1 µM), LB-100 (10 µM), or DMSO (control) for 1 h. Chromosome spreads were analyzed by immunofluorescence for H2B S6ph and CENP-A localization. Bars: 1 µm (magnification); 5 µm (main). **(B)** Cells were transfected with siRNAs targeting catalytic subunits of PP1α, β, and γ or control siRNAs (Origene). Knockdown efficiency was confirmed by qPRC (see Fig. S3 B). After 24 h, cells were arrested at G_1_/S by a thymidine block and released, followed by the addition of nocodazole for 6.5 h. The mitotic cells were further treated for 30 min with AZD1152 or the DMSO control and stained as shown. Bars, 2 µm. **(C)** The experiment was performed as in B, and fluorescence intensities (given in artitrary units; A.U.) were quantified from 10 representative cells per condition using ImageJ and GraphPad Prism. **(D)** The recombinant H2B protein was phosphorylated by an in vitro kinase assay as described for Fig. 6 A. CDK1-cyclin B1 was removed from the extracts by immuno-depletion and the supernatant containing 2 µg of phosphorylated H2B protein was taken and incubated with 0.2 µg of recombinant PP1α (Novus Biologicals) or GST control (Sigma) for 30 min at 37°C. The reaction was analyzed for histone phosphorylation by Western blotting (top) and protein integrity by SDS-PAGE and CBB staining as shown.

The localization of the H2B S6 kinase CDK1 is not restricted to centromeres (Pines and Hunter, 1991), suggesting that additional mechanisms ensure the spatially defined localization of H2B S6ph. To investigate whether the localization and intensity of H2B S6ph can be controlled by PP1/PP2A phosphatases, RPE-1 cells were released from a RO-3306 block into medium containing nocodazole and inhibitors with different specificities for PP1 and/or PP2A. Selective inhibition of PP2A with LB-100 (Cervone et al., 2018) resulted only in a slight extension of H2B S6ph to areas outside from the centromere, while simultaneous inhibition of PP1 and PP2A by okadaic acid (Fig. 7 A) resulted in a higher intensity of H2B S6ph and its spreading along the chromosome arms. These results were also confirmed in unsynchronized cells, where okadaic acid or calyculin A caused an increase in the intensity and localization of H2B S6ph in mitotic cells, while selective inhibition of PP2A by LB-100 remained without a significant impact (Fig. S3 A). Similarly, the knockdown of PP1 catalytic subunits with reported siRNAs (Qian et al., 2011) worked well (Fig. S3 B) and resulted in increased intensity and extended localization of H2B S6ph (Fig. 7 B). Aurora B regulates the activity of PP1 in mitosis (Liu et al., 2010; Nasa et al., 2018), so it was interesting to test the effect of Aurora B on H2B S6ph in the absence of PP1 phosphatases. In contrast to control siRNA treatment, where Aurora B inhibition abolished H2B S6ph, H2B S6ph was only slightly reduced by Aurora B inhibition in PP1 knockdown cells (Fig. 7 C). In vitro phosphatase assays showed the dephosphorylation of H2B S6 by the purified catalytic subunit of PP1α (Fig. 7 D), thus corroborating the role of PP1 as a H2B S6 phosphatase. Collectively, these data raise the possibility that Aurora B contributes to the restriction of PP1 activity to indirectly control the phosphorylation of H2B S6.

### H2B S6ph is required for mitotic chromosome segregation

**Figure 8.**
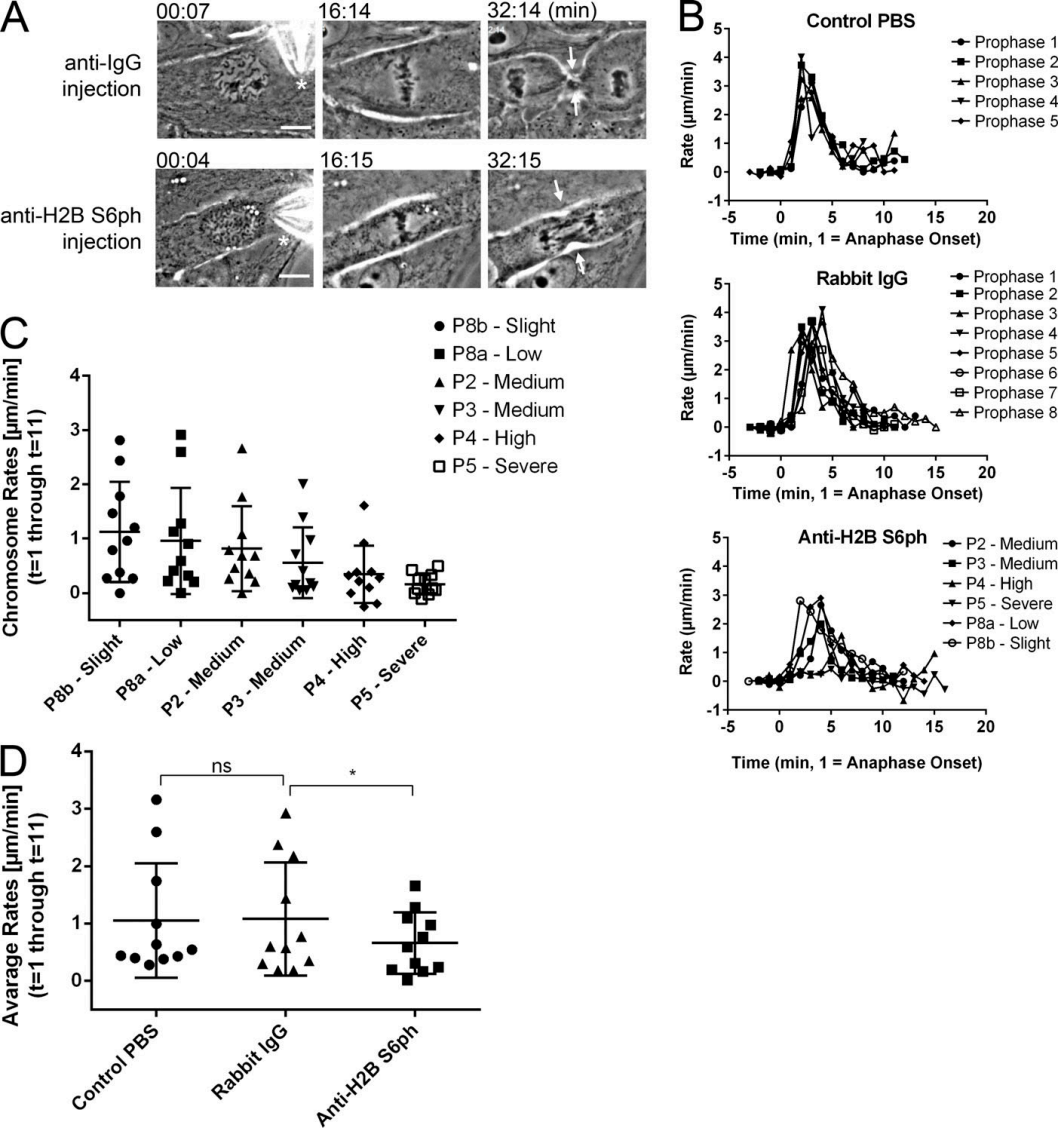
**Identification of mitotic H2B S6ph functions. (A)** Prophase LLC-PK cells were injected with anti-H2B S6ph antibodies or rabbit IgG at the onset of mitosis and further traced by live cell imaging. Pictures show representative phenotypes after injection of rabbit IgG (top) or anti-H2B S6ph antibodies (bottom). Time points after injection of antibodies are indicated (min:s), areas of microinjections are marked with asterisks (*) and the position of the cleavage furrows by arrows. Bars, 10 µm. **(B)** Prophase LLC-PK cells were microinjected with PBS (*n* = 5), rabbit IgG (*n* = 8), or anti-H2B S6ph antibodies (*n* = 6). Movement rates (changes in µm/min) during anaphase are displayed. Time lapse images after microinjection of antibody or PBS were collected at 1-min intervals. Chromosome movement rates just before and during anaphase were quantified by measuring the change in distance between leading edges of the chromosome masses as they moved poleward. Each line represents the chromosome movement rates obtained from individual cells. Each cell’s data were aligned so that minute “1” represents the first minute where anaphase movements were observed. **(C)** Chromosome movement rates during anaphase (t = 1 min through t = 11 min) were compared between samples from individual anti-H2B S6ph injections. Samples were labeled and arranged according to their phenotypes (ranging from slight to severe chromosome segregation defects). Lines show mean movement rates and standard deviations between the time points. **(D)** Average chromosome movement rates for every time point during anaphase (t = 1 min through t = 11 min) were calculated and compared between the three conditions using GraphPad Prism. Lines show mean movement rates and standard deviations between the time points. The asterisk (*) indicates a significant P value < 0.04; ns = not significant (paired *t* test).

For the investigations on the function of H2B S6ph, we did not interfere with CDK1-cyclin B1 activity, as the inhibition would necessarily affect the process of mitosis as such and modification of many substrates. To explore the specific role of H2B S6ph, phosphorylation-specific H2B S6 antibodies were injected into LLC-PK pig kidney cells, which are specifically suited for antibody injection assays. These cells also show mitotic H2B S6ph similar to human cells (data not shown). After antibody injection in prophase, progression of mitosis was followed by live cell imaging until telophase. Control injections with rabbit IgGs did not cause any mitotic abnormalities (Fig. 8 A; Fig. S4 A; and Video 1). Injection of antibodies recognizing H2B S6ph did not cause significant differences in mitotic progression between nuclear envelope breakdown and anaphase onset (Fig. S4 B), but caused chromosome segregation defects with various severities, according to the phenotypes described in Fig. S4 C. Injected cells progressed normally through mitosis until metaphase without defects in chromosome alignment (Fig. 8 B; Fig. S4 D; and Video 2). During anaphase, chromosome segregation appeared uncoordinated and slower than controls. Formation and ingression of the cleavage furrow started at the usual time, but before complete chromatid separation. Some chromosomes formed bridges or were trapped in the cleavage furrow, a feature known as a cell untimely torn (CUT) phenotype (Hirano et al., 1986). In the most severe case, chromosomes did not move and were forced into one daughter cell without separating (Fig. S4 C). Quantification of the chromosome movements during anaphase confirmed that segregation rates were indeed decreased after anti-H2B S6ph injection, compared with controls. The decline in chromosome movement rates correlated with the severity of the phenotype (Fig. 8, B and C). The average rates of chromosome movement were comparable after PBS and rabbit IgG injection, but significantly reduced to about half of the speed after anti-H2B S6ph injection (Fig. 8 D). This phenotype is markedly distinct from that of the anti–phospho-H3 T3 antibodies, which delocalize Aurora B from mitotic centromeres (Wang et al., 2010), or anti–phospho-H3.3 S31 antibodies, which abrogate the p53 response to chromosome missegregation (Hinchcliffe et al., 2016).

### H2B S6ph lowers binding of SET to chromatin

**Figure 9.**
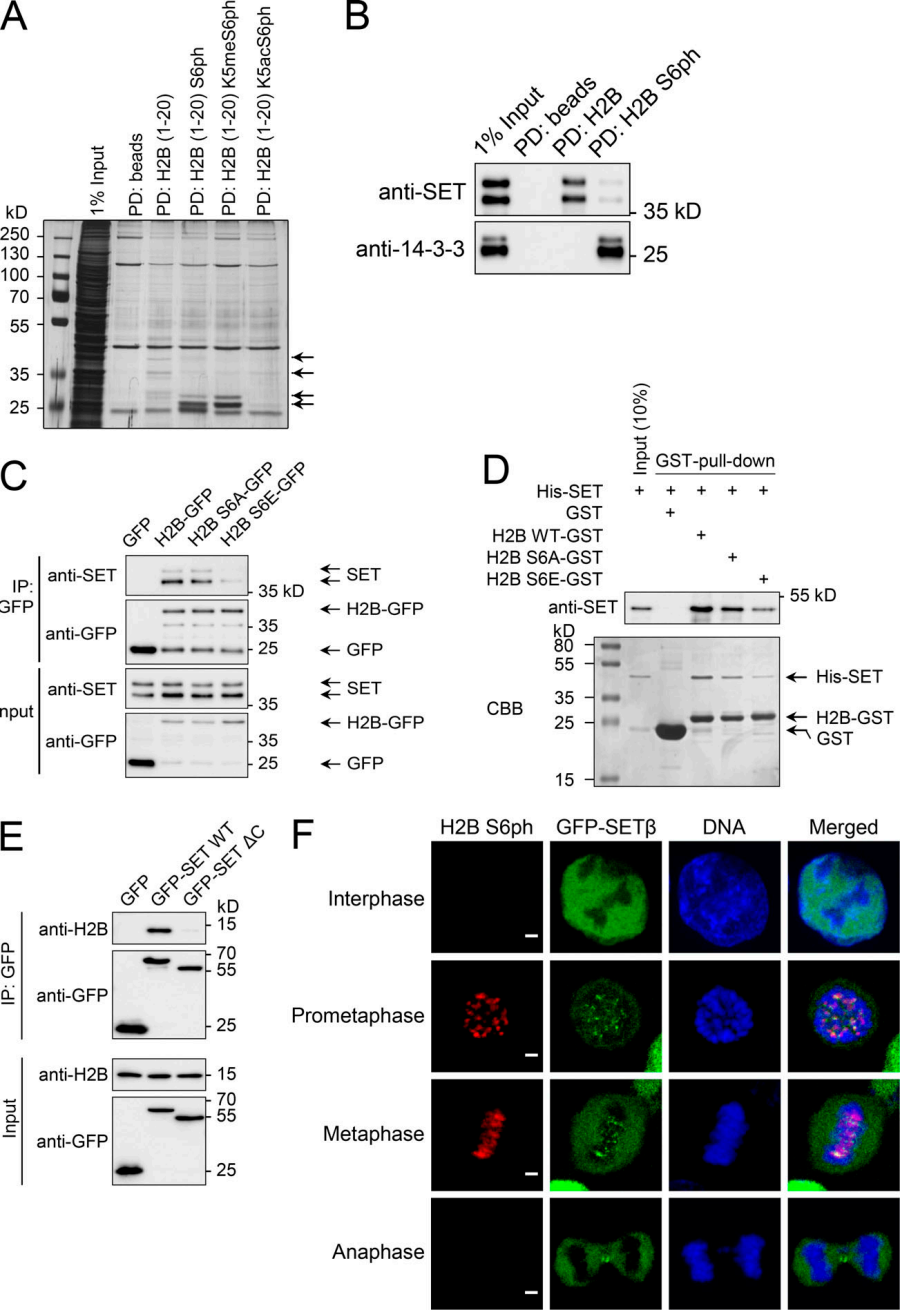
**Identification of proteins showing S6ph-dependent H2B binding. (A)** HeLa cells were arrested in mitosis with nocodazole for 16 h and lysed with NP-40 buffer. The indicated biotin-coupled peptides with unmodified or modified amino acids encompassing the first 20 amino acids from H2B were used for pulldown assays using StrepTactin beads (Qiagen). One fraction of the eluates was analyzed by SDS-PAGE and silver staining. Arrows indicate phosphorylation-dependent interactors. **(B)** Samples from peptide pulldown experiments were analyzed by SDS-PAGE and Western blotting using SET and 14-3-3 antibodies; arrows show the SET and 14-3-3 isoforms. **(C)** GFP-tagged H2B WT or S6 phosphorylation site mutants were expressed in 293T cells. Cells were lysed with NP-40 buffer, sonicated, and treated with Benzonase (Millipore). H2B-GFP was immunoprecipitated using the GFP-Trap and SET binding was analyzed by immunoblotting. **(D)** (His)6-tagged SET (His-SET) and the indicated H2B-GST fusion proteins were purified from *E. coli BL21* and then mixed as shown. Following incubation for 2 h of 1 µg GST fusion proteins or 5 µg GST and 0.5 µg his-SET, a GST pulldown was performed and the proteins were detected by CBB staining and immunoblotting as shown. **(E)** 293T cells were transfected to express GFP, GFP-SET, or GFP-SETΔC which lacks the 59 C-terminal amino acids. The experiment was further performed as in C, with the exception that endogenous H2B was detected by antibodies. **(F)** SET expression was eliminated in HCT116 cells by CRISPR-Cas9–mediated gene deletion, and a tet-inducible GFP-SET expression plasmid was stably introduced by puromycin selection. Following induction of GFP-SET expression by 1 µg/ml doxycycline for 24 h, cells were arrested with RO-3306 for 16 h and released into mitosis. Cells were stained against H2B S6ph, and the localization of H2B S6ph and GFP-SET in different mitotic phases is displayed. Bars, 2 µm.

To investigate whether protein–protein interactions can be regulated by H2B S6ph, we performed in vitro pulldown assays using biotinylated peptides encompassing the first 20 amino acids of H2B. Peptides were either unmodified or phosphorylated at S6 or comodified with acetylated or mono-methylated K5. Pulldown experiments were performed using native extracts from nocodazole-arrested HeLa cells, and bound proteins were analyzed by SDS-PAGE and silver staining (Fig. 9 A). Two protein populations in molecular weight range between 35 and 45 kD were found to interact preferentially with unmodified H2B, whereas phosphorylation of S6 led to an enrichment of proteins of sizes between 25 and 35 kD. Phosphorylation-dependent interactions were significantly diminished by H2B K5 acetylation. To identify the phosphorylation-dependent interactors, samples were analyzed by MS (Table S2). Label-free quantification of bound proteins revealed binding of 14-3-3 proteins to phosphorylated peptides. In contrast, S6 phosphorylation diminished binding of the SET protein. Western blot analysis with SET and 14-3-3 antibodies confirmed the phosphorylation-dependent interactions with H2B peptides (Fig. 9 B). Two isoforms of SET (SETα and SETβ) were detected to bind preferentially to the unmodified H2B tail, whereas interaction with 14-3-3 proteins was limited to the phosphorylated peptides.

The 14-3-3 protein family is involved in many cellular functions and cell cycle regulation (Gardino and Yaffe, 2011; Sawicka and Seiser, 2014; Sluchanko and Gusev, 2017). We did not detect 14-3-3 proteins in the chromatin fraction containing H2B S6ph (data not shown) and could not detect 14-3-3 proteins on mitotic chromosomes in colocalization with CENP-A or H3 S10ph (Fig. S5 A), arguing against the in vivo relevance of 14-3-3 protein binding to H2B S6ph, at least during mitosis. To test the interaction of H2B and SET, GFP-tagged H2B and phosphorylation-deficient or phospho-mimetic mutants thereof were expressed in 293T cells and immunoprecipitated from lysates after sonication and benzonase treatment to allow extraction of H2B-GFP from nucleosomes (Fig. 9 C). Binding of endogenous SET was detected for H2B-GFP and H2B S6A-GFP, while the S6E mutation drastically reduced interaction with SET. To test whether this interaction is direct, we examined the binding between recombinant and purified full-length SET and H2B (amino acids 1–35) fused to GST. H2B-GST and its phosphorylation-defective and phospho-mimicking mutants and His-tagged SET were expressed and purified from *Escherichia coli*, and the interaction was tested by GST pulldown assays. These experiments showed direct interaction between the proteins, which was reduced for the phospho-mimicking H2B variant (Fig. 9 D). Co-immunoprecipitation (IP) experiments showed the necessity of the C-terminal acidic domain of SET for interaction with endogenous H2B in extracts from 293T cells (Fig. 9 E), as previously shown for SET binding to other histones (Seo et al., 2001; Kim et al., 2012).

To determine the degree of colocalization between SET and H2B S6ph during mitosis in the absence of a suitable antibody faithfully detecting endogenous SET, we determined SET localization by scalable expression of GFP-SET in a SET CRISPR-Cas9 knockout background (Krishnan et al., 2017) to avoid overexpression artifacts (Fig. S5 B). This analysis showed the relocation of SET from the nucleus of interphase cells to inner centromeres and the nonchromatin fraction during early mitosis, as previously reported (Lam et al., 2012; Krishnan et al., 2017). GFP-SET and H2B S6ph displayed adjacent and also partially overlapping localizations at centromeres during prometaphase and metaphase, and GFP-SET was largely absent from chromosomes in anaphase (Fig. 9 F).

**Figure 10.**
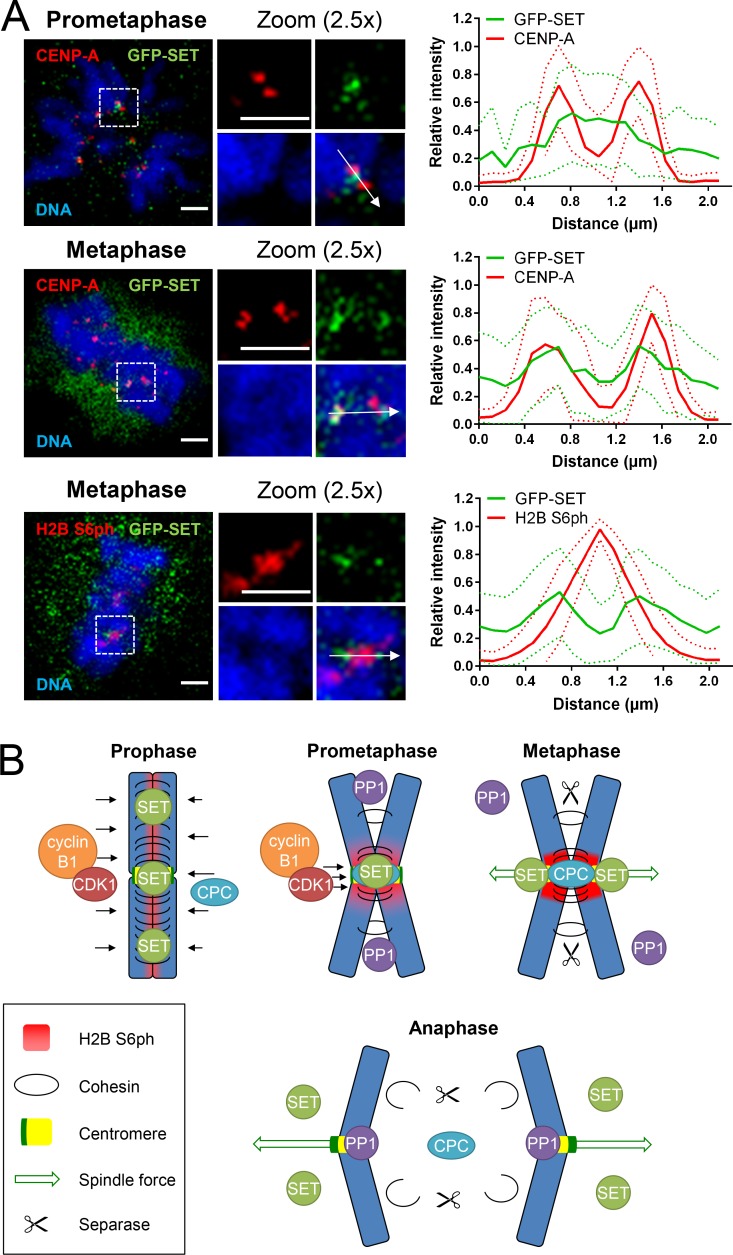
**Spatial distribution of H2B S6ph and SET during mitosis. (A)** The HCT116 cells described in Fig. 9 (F) were induced to trigger GFP-SET expression by the addition of 1 µg/ml doxycycline for 24 h. The top and middle panels show representative examples for fixed cells that were analyzed by confocal microscopy for the distribution of GFP-SET and CENP-A in prometaphase and metaphase cells. Line scan analysis was performed for 24 centromere pairs from three different prometaphase and metaphase cells, respectively. Quantification was done using ImageJ and GraphPad Prism; the maximum of fluorescence intensity was set as 1, and the dotted lines indicate standard deviations. The bottom panel shows the comparison of H2B S6ph and GFP-SET distribution in metaphase cells; line scan analysis was done by quantitative evaluation of 48 centromere pairs from 12 different cells. Bars, 2 µm. **(B)** Schematic summary depicting the possible molecular mechanisms leading to spatial and temporal control of H2B S6ph. Mitotic chromosomes are shown in blue, and the position of phosphorylated H2B is indicated in red, CENP-A chromatin in yellow, and kinetochores in dark green. Black arrows represent kinase activity of CDK1-cyclin B1 and Aurora B as part of the CPC.

We also used the GFP-SET expressing cells to compare the localization of SET and CENP-A between prometaphase and metaphase cells (Fig. 10 A). While prometaphase cells showed SET mainly between the centromeres, metaphase cells showed largely cytosolic SET with the remaining chromatin-bound SET at centromeres. Direct costaining between SET and H2B S6ph in metaphase cells confirmed the localization of H2B S6ph at the inner centromere, while most of the SET protein was found in the adjacent centromere regions (Fig. 10 A). These data suggest that colocalization between SET and H2B S6ph is still possible during prometaphase, but largely absent during metaphase when this phosphorylation has reached its maximum and highest local density.

## Discussion

Here, we identify H2B S6 as a novel histone phosphorylation site that is conserved among vertebrates. The N-terminal domain of human H2B contains many different modification sites, and future studies must reveal whether H2B S6ph has an impact on any of the neighboring modifications or vice versa. We did not detect significant amounts of H2B S6ph during interphase or in response to various agents causing cell stress, but this does not exclude the possibility that H2B S6ph can be triggered in response to further, yet unknown cues. The spatiotemporal occurrence of H2B S6ph is tightly regulated. During early mitosis, this modification is found at the inter-chromatid axis, while prometaphase chromosomes show this modification almost exclusively at inner centromeres.

H2B S6ph occurs coincident with chromosome condensation and spindle formation during early mitotic phases. These time points are characterized by ongoing CDK1-cyclin B1 and Aurora B activity, and accordingly, these kinase activities are required for H2B S6ph. H2B S6ph colocalizes with Aurora B at inner centromeres, where Aurora B contributes to the control of timing and distribution of H2B S6ph, though the used mechanisms are likely to be indirect and must be elucidated in the future. One possibility would be an indirect Aurora B–mediated control on downstream H2B S6 kinases such as CDK1, while another option is the Aurora B–dependent restriction of PP1 activity, as revealed in this study. This finding fits previous reports showing that Aurora B opposes PP1 function in mitosis by phosphorylating PP1-targeting proteins (Liu et al., 2010; Qian et al., 2013; Nasa et al., 2018). The direct phosphorylation appears to be performed by cyclin B1–associated CDK1, although we cannot exclude the participation of further kinases. CDK1 is known to be activated by cyclin B, but also by association with cyclin A (Katsuno et al., 2009), a cyclin that also associates with CDK2 to allow regulation of S phase and mitotic centrosome doubling (Tsai et al., 1993; Hinchcliffe and Sluder, 2002). We tested the ability of cyclin A–associated CDK1 and CDK2 for their ability to phosphorylate H2B S6 by in vitro kinase assays (Fig. S5 C). These assays showed that cyclin A2–associated CDK1 was capable of phosphorylating H2B S6, while CDK2 showed a reduced ability to phosphorylate H2B S6. Together, all experiments point to a crucial role of cyclin B1–associated CDK1 for mitotic H2B S6ph, but a possible contribution of further CDK1-associated cyclins or even other CDKs need to be investigated in future studies. A previous study showed that CDK substrates lacking the classical (S/T)-P motifs are specifically dependent on the presence of C-terminal R/K residues, and phosphorylation is enhanced by N-terminal P residues (Suzuki et al., 2015), which matches with the sequence of the H2B tail (see Fig. 1 A). Further support for H2B S6 being a CDK substrate comes from the notion that this sequence is P-rich and lacks any secondary structure, in line with the preferences of CDK substrate regions to be highly unstructured (Moses et al., 2007).

The decay in H2B S6ph seen after metaphase-to-anaphase transition likely results from the differential activities of CDK1-cyclin B1, Aurora B, and PP1. Cyclin B1 is rapidly degraded at anaphase, and Aurora B transitions from chromosome arms and inner centromeres in early mitosis to midzone spindle microtubules at anaphase (Carmena et al., 2012). Conceivably, H2B S6 phosphorylation could also be controlled by SET, which was identified as an interactor of cyclin B1 and a CDK1 inhibitor (Canela et al., 2003), raising the possibility that SET also contributes to a negative feedback loop antagonizing H2B S6 kinase activity. Furthermore, diminished H2B S6 phosphorylation could also be caused by changes in the localization of these regulators during sister chromatid separation and chromosome segregation. In early mitosis, CDK1-cyclin B1 and Aurora B phosphorylate substrates along chromosomes such as cohesin regulator Sororin to release cohesin from chromosome arms (Nishiyama et al., 2013). Cyclin B1 also localizes to unattached kinetochores (Bentley et al., 2007; Chen et al., 2008). Consistently, we observe H2B S6ph enriched at inner centromeres on chromosomes from prometaphase cells. The spatial regulation of H2B S6ph also involves a process of active dephosphorylation by PP1 phosphatases. Transition of Aurora B from inner centromeres to spindle microtubules during chromosome segregation (Goto et al., 2006) could thus enable H2B S6 dephosphorylation by PP1 in the absence of CDK1 and Aurora B activity.

As schematically depicted in Fig. 10 B, PP1 phosphatase activity could efficiently eliminate H2B S6ph at regions with low de novo phosphorylation. High CDK1 directly phosphorylates H2B S6 and Aurora B activity opposes phosphatase activity at centromeres to generate H2B S6ph. Focal H2B S6ph requires not only the delicate balance between phosphorylating and dephosphorylating activities, but may also depend on the coordinated progression of chromosome condensation and sister chromatid separation.

We suggest that chromatin association of SET may be controlled by various mechanisms. One mechanism would involve competition between available binding sites and repelling modifications. The histone-binding affinity of SET is lowered by phosphorylation of H2B S6, as revealed in this study, but also by phosphorylation of H3 at T3, S10, T11, or S28 (Schneider et al., 2004; Kim et al., 2012). Of note, phosphorylation of H2B S6 and H3 T3 are both found at inner centromeres, and together, these modifications may locally accumulate sufficient negative charge to reduce the binding affinity of the negatively charged C-terminal region of SET, which mediates the contact to H2B. It is plausible that an increase in number and local density of phosphorylations will decrease the chromatin affinity of SET, thus enabling these phosphorylations to function as a scalable affinity regulator rather than functioning as a switch-like toggle.

It is also possible that, rather than displacing SET from chromatin, core histone phosphorylation acts to prevent the histone chaperone activity of SET at centromeres. Indeed, SET localization can also be determined by its binding partner Shugoshin, which moves from the inner centromere to kinetochores between prometaphase and metaphase (Liu et al., 2013). Future studies have to reveal the relative contribution of these different mechanisms mediating the progressive eviction of SET from the chromatin between prometaphase and metaphase.

How can H2B S6ph influence chromosome segregation? Sister chromatids are first resolved at the chromosome arms during prophase, while centromeric cohesion is maintained until metaphase. A recent study suggests that SET serves to evict phosphorylated linker histone H1 (H1 T18ph) and the cohesin protector Shugoshin from chromatin to allow sister chromatid separation and chromosome segregation in mitosis (Krishnan et al., 2017). The need to control the activity and localization of SET is also illustrated by the finding that its overexpression can cause a premature loss of sister chromatid cohesion in mouse oocytes (Qi et al., 2013) and SET depletion interferes with meiotic chromosome segregation (Chambon et al., 2013). We speculate that H2B S6ph (and perhaps H3 phosphorylation) impairs the function of SET during mitosis to preserve cohesion at inner centromeres until anaphase. Interestingly, RPE-1 cells lacking SET exhibit a mitotic phenotype similar to cells injected with phosphorylation-specific H2B S6 antibodies, displaying chromosome missegregation and chromosome trapped in cleavage furrows (Krishnan et al., 2017). The function of H2B S6ph for the regulation of mitosis might not be restricted to the limitation of SET binding and involve further mechanisms to be elucidated in future studies.

## Materials and methods

### Antibodies, plasmids, and reagents

All the information on antibodies, plasmids, and reagents is given in Table S3.

### siRNA screen

Hela cells were grown in 384-well clear-bottomed plates and treated with a siRNA kinome library (Dharmacon). Cells were then accumulated in mitosis with 200 nM nocodazole and immunofluorescently stained using H2B S6ph and H3 T3ph (16B2) antibodies. Average fluorescence intensity (integrated) for mitotic cells (defined as cells positive in either H2B S6ph or H3 T3ph) was measured using a wide-field Nikon microscope (Eclipse Ti-E) equipped with high-content analysis software. Excel was used to calculate the standard score for each repeat (*n* = 4), and P values were calculated using one-sample *t* test for each treatment. (∼300 cells measured for each treatment.) Staining for both H2B S6ph and H3 T3ph meant that each could serve as an internal control for the other.

### Generation of phospho-specific antibodies

Anti-H2B S6ph antibodies were generated by immunization of two rabbits with peptide SP120160 (Table S3) by PolyPeptide Laboratories. Phosphorylation-specific antibodies were affinity purified from sera (large and final bleedings), using peptides C-PEPAK (for removal of unspecific antibodies; Table S3) and SP120160 (for collection of phosphorylation-specific antibodies) by Peptide Specialty Laboratories and Eurogentec.

### MS

Samples for MS analysis were prepared with LDS sample buffer (2× NuPAGE LDS Sample Buffer and 2× NuPAGE Sample Reducing Agent in MilliQ water) and SDS-PAGE. Gels were stained with Coomassie brilliant blue (CBB) and cut into small pieces. Gel pieces were washed twice with 50% (vol/vol) ethanol in ABC buffer (50 mM ammonium bicarbonate) and dehydrated with 100% (vol/vol) ethanol. Proteins were reduced with 10 mM DTT at 56°C for 45 min and alkylated with 55 mM iodacetamide at room temperature for 30 min. Gel pieces were repeatedly washed with ABC buffer and dehydrated with 100% (vol/vol) ethanol. Proteins were digested with 48 ng Trypsin in ABC buffer at 37°C for 16 h. Peptides were extracted with 30% (vol/vol) acetonitrile and 3% (vol/vol) trifluoroacetic acid (TFA). Extraction was repeated with 70 and 100% (vol/vol) acetonitrile. Extracts of each gel piece were combined and mixed with one volume buffer C (5% [vol/vol] acetonitrile and 1% [vol/vol] TFA). StageTips were prepared with two layers of C18 resin and washed with methanol, buffer B (80% [vol/vol] acetonitrile and 0.5% [vol/vol] acetic acid) and twice with buffer A (0.5% [vol/vol] acetic acid in MilliQ water). Extracts were loaded on one StageTip per gel piece. StageTips were again washed with buffer A and stored for MS analysis at 4°C.

MS analysis was performed with an LTQ-orbitrap Velos instrument (Thermo Fisher) equipped with a nanoelectrospray source (Proxeon). The mass spectrometer was operated in the data dependent mode to monitor MS and MS/MS spectra. Survey full-scan MS spectra (from m/z 300 to 2,000) were acquired in the Orbitrap with a resolution of R = 60,000 at m/z 400 after accumulation of one million ions. The five most intense ions from the preview survey scan delivered by the Orbitrap were sequenced by collision-induced dissociation in the LTQ. For higher C-trap dissociation with a LTQ-Orbitrap Velos instrument, 30,000 ions were accumulated in the C-trap and MS/MS spectra were detected in the Orbitrap at a resolution of 7,500 (Olsen et al., 2010). Mass spectra were analyzed using MaxQuant software (Version 1.0.14.10) and automated database searching (Matrix Science; Version 2.2; Cox and Mann, 2008). A human UniProt (2015) database with common contaminants was used for peptide and protein identification. A false discovery rate of 1% was used for both the peptide spectrum matches and protein level matches with the implemented target decoy algorithm. The required false positive rate was set to 1% at the protein level, and maximum allowed mass deviation was set to 5 ppm in MS mode and 0.5 D for MS/MS peaks. Cysteine carbamidomethylation was searched as a fixed modification, and N-acetyl protein, oxidized methionine, and acetylation of lysine was searched as variable modifications. A maximum of three missed cleavages were allowed.

### Cell culture and transfections

Human embryonic kidney 293 T cells, HCT116, HeLa, and RPE-1 cells were grown in DMEM or DMEM/F12 (RPE-1) containing 10% FCS and 1% (vol/vol) penicillin/streptomycin at 37°C and 5% CO_2_. Cells were seeded in dishes and transfected using the transfection reagents Rotifect (Roth), Lipofectamine 3000 (Invitrogen), or linear polyethylenimine. After pipetting up and down several times, complex formation occurred in serum- and antibiotic-free DMEM during 30 min at room temperature. After adding the transfection mixture to antibiotic-free DMEM containing FCS, the cells were incubated 4 h before the medium was changed and the cells were further grown.

### Cell lysis

To prepare cell extracts under native conditions, the cells were washed once with 1× PBS, harvested by scraping, and collected by centrifugation for 4 min at 350 *g*. The pellet was resuspended in an appropriate amount of NP-40 buffer (20 mM Tris/HCl, pH 7.5, 150 mM NaCl, 1 mM phenylmethylsulfonylfluoride, 10 mM NaF, 0.5 mM sodium orthovanadate, leupeptine [10 µg/ml], aprotinin [10 µg/ml], 1% NP-40, and 10% glycerol) and incubated on ice for 20 min. The lysate was cleared by centrifugation for 10 min at 16,000 *g*. The supernatant was then used for IP experiments or mixed with 5× SDS sample buffer, boiled at 95°C for 5 min, and analyzed by Western blotting. Alternatively, cells were directly lysed in 1× SDS sample buffer, sonified to shear the genomic DNA, and used for Western blotting.

### Cell cycle synchronization

For interphase synchronization, cells were treated with complete medium containing 2 mM thymidine for 16 h (first thymidine block). Cells were then washed three times with warm PBS and released in medium without thymidine for 8 h. Synchronization was completed by a second thymidine block for 16 h. After three times of washing, cells were released into S phase. Mitotic synchronization was performed either by CDK1 inhibition (RO-3306) or by mitotic spindle disruption (nocodazole and taxol). Treatment for 16 h with 10 µM RO-3306 caused cell cycle arrest at the G_2_/M transition. Cells were washed three times with warm PBS and released into M phase. For mitotic arrest in prometaphase, cells were treated with 0.1 µg/ml nocodazole or 100 nM taxol for 8–16 h. Mitotic cells were collected by shake-off and released with fresh medium or kept in mitosis by addition of 10 µM MG132.

### Immunofluorescence staining

Cells were grown on coverslips in 12-well plates. Attached cells were washed with PBS and fixed with 4% (vol/vol) paraformaldehyde at room temperature for 5 min. After having been washed for three times with PBS, cell membranes were permeabilized with 0.5% (vol/vol) Triton X-100, and unspecific antibody binding was blocked with blocking solution (PBS containing 10% [vol/vol] goat serum and 0.5% [vol/vol] Triton X-100) at room temperature for 1 h. Cells were incubated with primary antibody solutions (PBS containing 1% [vol/vol] goat serum and 0.5% [vol/vol] Triton X-100) at 4°C for 16 h. Cells were washed three times with PBS and incubated with secondary antibodies for 1 h at room temperature. The secondary antibodies conjugated to Alexa Fluor 488, 594, and 647 or Cy3 were purchased from Dianova and Invitrogen Molecular Probes. After incubation, cells were washed three times, and DNA was stained with 1 µg/ml Hoechst 33342. Coverslips were washed again and mounted on microscope slides with Mowiol mounting medium. Slides were stored at 4°C while being protected from light.

### Preparation of mitotic chromosome spreads

Cells were grown on coverslips, and mitotic cells were arrested with nocodazole for 1–6 h. After medium removal, hypotonic buffer (0.8% [wt/vol] sodium citrate/H_2_O; freshly added: 10 mM NaF; 0.5 mM Na_3_VO_4_; 2 µg/ml aprotinin, 1 µg/ml leupeptin; and 1 mM PMSF) was added drop-wise, and cells were swollen for 15 min at room temperature. Cells were centrifuged on coverslips with a CytoSpin (Thermo Fisher) at 250 *g* for 5 min and processed for immunofluorescence staining.

### Microinjections

LLC-PK pig kidney cells were plated on 25-mm round coverslips and cultured to a density of ∼70%. Before microinjection, the coverslips were mounted in stainless steel chambers with Leibovitz’s L-15 medium supplemented with 10% FBS and 1% penicillin and streptomycin and overlaid with light mineral oil (Sigma) to prevent evaporation. Mitotic cells were injected using a Burleigh micromanipulator and microneedles containing 2 mg/ml anti-H2B S6ph antibodies or, for control injections, nonspecific rabbit IgG. Time-lapse phase contrast and fluorescence images were collected at 37°C using a 100×/1.4 NA oil objective and an inverted microscope (AxioObserver Z1; Zeiss), equipped with a stage heater, air curtain, an ORCA-Flash4.0LT camera (Hamamatsu), and Slidebook software (Intelligent Imaging Innovations, Inc.). During microinjection, images were collected every 170 ms and thereafter at 1 min intervals. Individual cells were evaluated for mitotic progression, and image panels and videos were assembled using MetaMorph software (Molecular Devices). Distance measurements were performed using the Metamorph’s caliper tool.

### In vitro kinase and phosphatase assays

In vitro kinase assays were performed with immunoprecipitated kinases or recombinant proteins. Cells were lysed under native conditions and kinases were purified by IPs. Beads were washed three times with NP-40 IP buffer and twice with kinase buffer (60 mM Hepes, pH 7.5, 3 mM MgCl_2_, 3 mM MnCl_2_, and 1.2 mM DTT). Supernatants were removed completely from beads and 30 µl of kinase reaction mix (6 mM Hepes, pH 7.5, 3 mM MgCl_2_, 3 mM MnCl_2_, 1.2 mM DTT, 10 mM NaF, 10 mM β-glycerophosphate, 1 mM Na_3_VO_4_, 5% [vol/vol] glycerol, 20 µM ATP, and 2 µg substrate protein) was added. Samples were incubated at 37°C for 30 min and the reaction was stopped by addition of SDS sample buffer. After heating at 95°C for 5 min, samples were centrifuged (16,000 *g*; 2 min), and supernatants were analyzed by SDS-PAGE and Western blotting. In vitro phosphatase assays were performed by incubation of 0.2 µg PP1α (Novus Biologicals) with 2 µg phosphorylated H2B S6 using kinase buffer lacking ATP and the phosphatase inhibitors NaF, β-glycerophosphate, and Na_3_VO_4_ for 30 min at 37°C.

### IP

Proteins from a cell lysate were precipitated with antibodies bound to protein A/G sepharose beads (Millipore) or with GFP-Trap beads (Chromotek) or anti-Flag M2 affinity gel (Sigma). Native buffer conditions (NP40 buffer: 20 mM Tris/HCl, pH 7.5, 150 mM NaCl, 1% [vol/vol] NP-40, 10% [vol/vol] glycerol, 10 mM NaF, and 0.5 mM Na_3_VO_4_) were used for enzymatic assays and co-IPs. Denaturing conditions (radio-IP assay buffer: 50 mM Tris/HCl [pH 7.4], 150 mM NaCl, 1 mM EDTA, 0.1% [wt/vol] SDS, 0.5% [wt/vol] sodium deoxycholate, 1% [vol/vol] NP-40, 10 mM NaF, and 0.5 mM Na_3_VO_4_) were needed for IP of chromatin proteins. In general, 25 µl beads per sample were equilibrated with IP buffer and mixed with up to 1 µg of antibody. Supernatants from lysis under native or denaturing conditions were added and samples were rotated at 4°C. Up to 10% of the supernatants were collected as input controls. After 3–4 h, beads were washed five times with 1 ml IP buffer at 4°C for 10 min. Bound proteins were subjected for further use or eluted by addition of 30 µl 1.5× SDS sample buffer followed by incubation at 95°C for 5 min. Samples were centrifuged at 16,000 *g* for 2 min, and supernatants were analyzed by SDS-PAGE and Western blotting.

### Peptide pulldown assays

Peptide pulldown assays were performed with native cell lysates and biotinylated peptides. 2.5 µg peptides were dissolved in 100 µl PBS and incubated with Strep-Tactin magnetic beads (Qiagen) at 4°C for 1 h. Unbound peptides were removed by washing three times with PBS. Beads were added to cell lysates and rotated at 4°C for 3 h. Beads were washed five times with cold PBS, and proteins were eluted with 30 µl 2× LDS sample buffer and incubated at 95°C for 5 min. Eluates were centrifuged at 16,000 *g* for 2 min, and supernatants were collected for further analysis.

### ELISA

ELISA was performed with NeutrAvidin coated 96-well plates (Pierce) and biotinylated peptides. Pre-blocked plates were washed three times with ELISA wash buffer (TBS-T with 0.1% [wt/vol] BSA). Peptides were dissolved in PBS, and 2.5 µg peptide was added per well. Peptide binding was achieved at room temperature after 2 h. Unbound peptides were removed by washing three times with ELISA wash buffer, and decreasing dilutions of test antibodies were transferred to the wells for 20 min. Wells were washed three times, and secondary HRP-conjugated antibodies were added for 20 min. Wells were washed again three times, and substrate solution (110 mM sodium acetate, pH 5.5; 0.1% [vol/vol] H_2_O_2_; and 0.1 µg/ml TMB) was added for 5 min. Reaction was stopped by addition of one volume stop solution (10% [vol/vol] H_2_SO_4_), and antibody binding was quantified by emission at 450 nm.

### Microscope image acquisition

3D-SIM (for Figs. 1 E; 2, A–C; 3, A and B; and 4, A and C) was performed with a Zeiss Elyra PS1 microscope fitted with a Plan Apochromat differential interference contrast (DIC), 63×/1.4 NA oil lens. Images were acquired at room temperature using an EM charge-coupled device Andor iXon DU 885 camera, using five rotations and five translations of the illumination pattern. High-power diode lasers of 405-, 488-, 561-, and 642-nm wavelengths were used in combination with BP 420–480, BP 495–575, BP 570–650, and LP 655 emission filters, respectively. Raw images were reconstructed into high resolution images using Zeiss ZEN 2012 software. Confocal images for Fig. 4 (B and D) were acquired at room temperature with a Leica TCS SP8X White Light confocal microscope equipped with a 63×/1.4 NA oil lens and HyD detector (Leica Microsystems), using a 0.13-µm z-step size (∼15–30 z planes) and Leica Application Suite X (LAS X) software. Representative images were deconvolved using Huygens Software (Scientific Volume Imaging b.v.) and shown as maximum intensity projections. Confocal images for Fig. 7 B, Fig. 9 F, Fig. 10 A, and Fig. S2 were acquired at room temperature with a Leica TCS SP2 AOBS microscope using a Plan-Apochromat 63×/1.4 NA oil lens (Leica Microsystems) and Leica LCS acquisition software. Samples were illuminated using a diode 405-nm laser, a HeNe 543-nm laser, and an Argon laser for 488-nm excitation. Immunofluorescence images for figure for Fig. 6 D, Fig. 7 A, Fig. S3 A, and Fig. S5 A were acquired at room temperature with an Eclipse TE2000-E inverted microscope (Nikon) equipped with an X-Cite Series 120 fluorescence microscope light source (EXFO), a T-RCP remote control (Nikon), an ORCA-spark Digital CMOS camera C11440-36U (Hamamatsu), and a Nikon Plan-Apochromat 100×/1.4 NA oil lens using NIS Elements AR 3.00 software (Nikon). The open source image processing program ImageJ was used for image projection (maximum intensity) and calculation of scale bars. Brightness and contrast were adjusted with ImageJ or Adobe Photoshop. Fluorescence intensities were quantified with ImageJ and analyzed using GraphPad Prism.

### Real-time quantitative PCR (qPCR)

RNA was extracted with RNeasy Mini kit and QIAshredder (Qiagen) according to the manufacturer’s instructions. Messenger RNA was converted to cDNA by reverse transcription using the SuperScript II RT (Thermo Fisher). Relative expression of target genes was analyzed by real-time qPCR. SYBR green (Thermo Fisher) was used as a fluorescence reporter for cDNA levels during amplification by PCR. Real-time qPCR was performed in StepOnePlus Real-Time PCR system (Applied Biosystems). Every reaction was performed as triplicates and quantified with the ΔΔC_T_-method. Therefore, threshold cycles (C_T_) of target genes were normalized to a housekeeping gene (*ACTB*). The resulting ΔC_T_ were compared with control samples and relative mRNA expression was calculated by R = 2^−ΔΔC^_T_.

### Online supplemental material

Fig. S1 shows results from the characterization of the phospho-specific antibodies. Fig. S2 shows the effects of kinase inhibitors on H2B S6ph. Fig. S3 shows the effects of phosphatase inhibition on H2B S6ph. Fig. S4 relates to Fig. 8 and displays further details for the antibody injection experiments. Fig. S5 shows the localization of 14-3-3 proteins, the inducible expression of GFP-SET, and in vitro kinase assays with recombinant CDK complexes and histone octamers as substrates. Table S1 shows the results from the siRNA kinase screen. Table S2 displays MS results for the identification of phosphorylation-dependent H2B(1–20) interactors. Table S3 lists reagents and materials. Video 1 shows the mitotic effects caused by the injection of IgG control antibodies. Video 2 shows the mitotic effects caused by the injection of anti-H2B S6ph antibodies.

## Supplementary Material

Supplemental Materials (PDF)

Tables S1-S3 (ZIP)

Video 1

Video 2
