# Supplementary material for: CDK1-mediated phosphorylation at H2B serine 6 is required for mitotic chromosome segregation
Source: J Cell Biol. 2019 Feb 14;218(4):1164–81. doi: 10.1083/jcb.201806057 (PMC6446833; doi:10.1083/jcb.201806057)
Supplement: Supplemental Materials (PDF) [file JCB_201806057_sm.pdf]

Supplemental material

Seibert et al., https://doi.org/10.1083/jcb.201806057

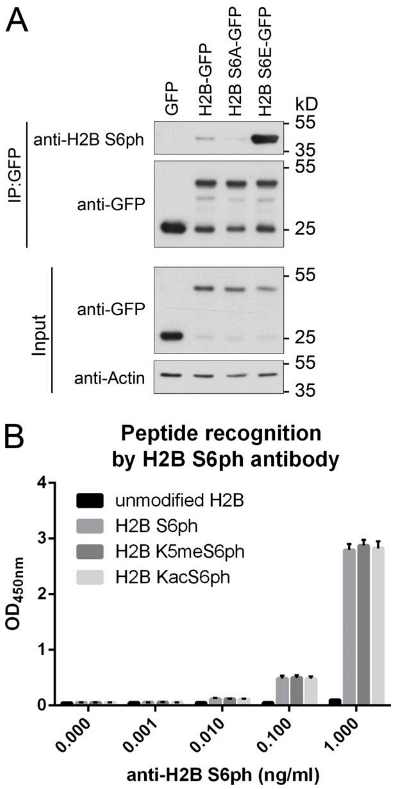

Figure S1. **Results from the characterization of the phospho-specific antibodies. (A)** H2B-GFP or phosphorylation-deficient (H2B S6A-GFP) or phospho-mimicking (H2B S6E-GFP) variants thereof were expressed in 293T cells together with a GFP control. Cells were lysed with radio-IP assay buffer and sonicated for chromatin solubilization. H2B-GFP was immunoprecipitated from extracts with the GFP-Trap, and samples were analyzed by SDS-PAGE and Western blotting using the indicated antibodies. **(B)** Increasing concentrations of anti-H2B S6ph antibodies were incubated with 2.5 µg immobilized peptides consisting of the first 20 amino acids of H2B. Peptides were either unmodified, phosphorylated on S6 (S6ph) or comodified by K5 mono-methylation (K5meS6ph) or acetylation (K5acS6ph). Antibody binding to peptides was quantified by ELISA; error bars show standard deviations between three technical replicates.

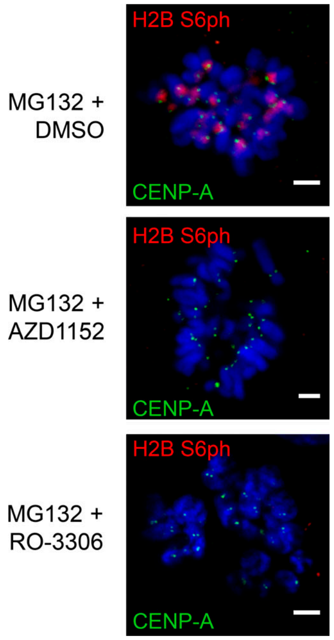

Figure S2. **Effects of kinase inhibitors on H2B S6ph.** RPE-1 cells were arrested in prometaphase by treatment with nocodazole for 6 h and released in the presence of 10 µM MG132, together with the indicated kinase inhibitors. DNA was stained using Hoechst 33342. Bars, 2 µm.

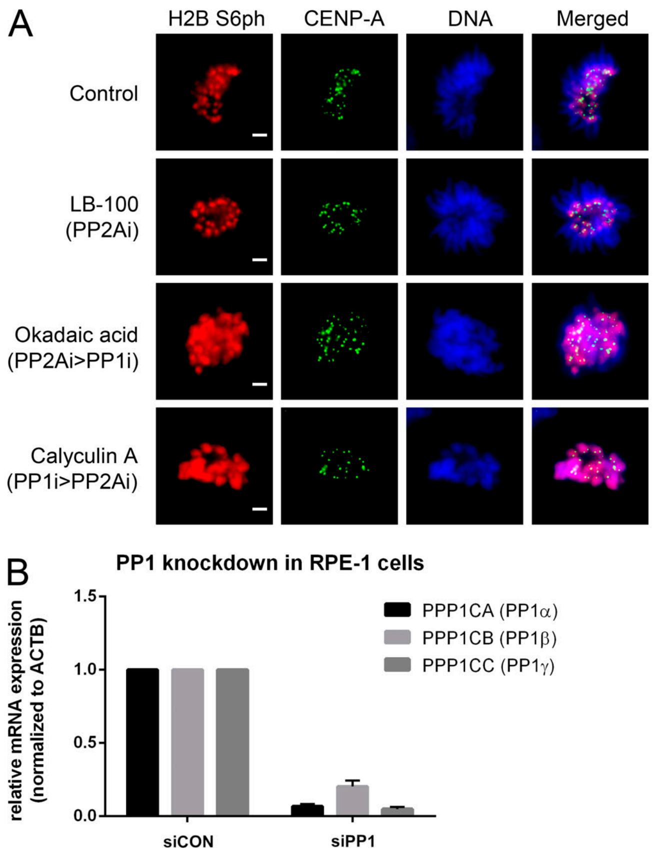

Figure S3. **Effects of phosphatase inhibition on H2B S6ph. (A)** Unsynchronized RPE-1 cells were treated with LB-100 (10 µM), okadaic acid (1 µM), calyculin A (10 nM), or DMSO (control) 1 h before fixation. The specificity for phosphatase inhibition is also indicated in parentheses. Mitotic cells were stained with the indicated antibodies. DNA was stained using Hoechst 33342. Bars, 2 µm. **(B)** RPE-1 cells were transfected with siRNAs targeting PP1α, β, and γ (siPP1) or scrambled control siRNAs (siCON). One aliquot of the cells was tested for efficient knockdown by qPCR with specific primers. To facilitate comparison, gene expression measured with control siRNAs was arbitrarily set as 1; error bars show standard deviations of three biological replicates.

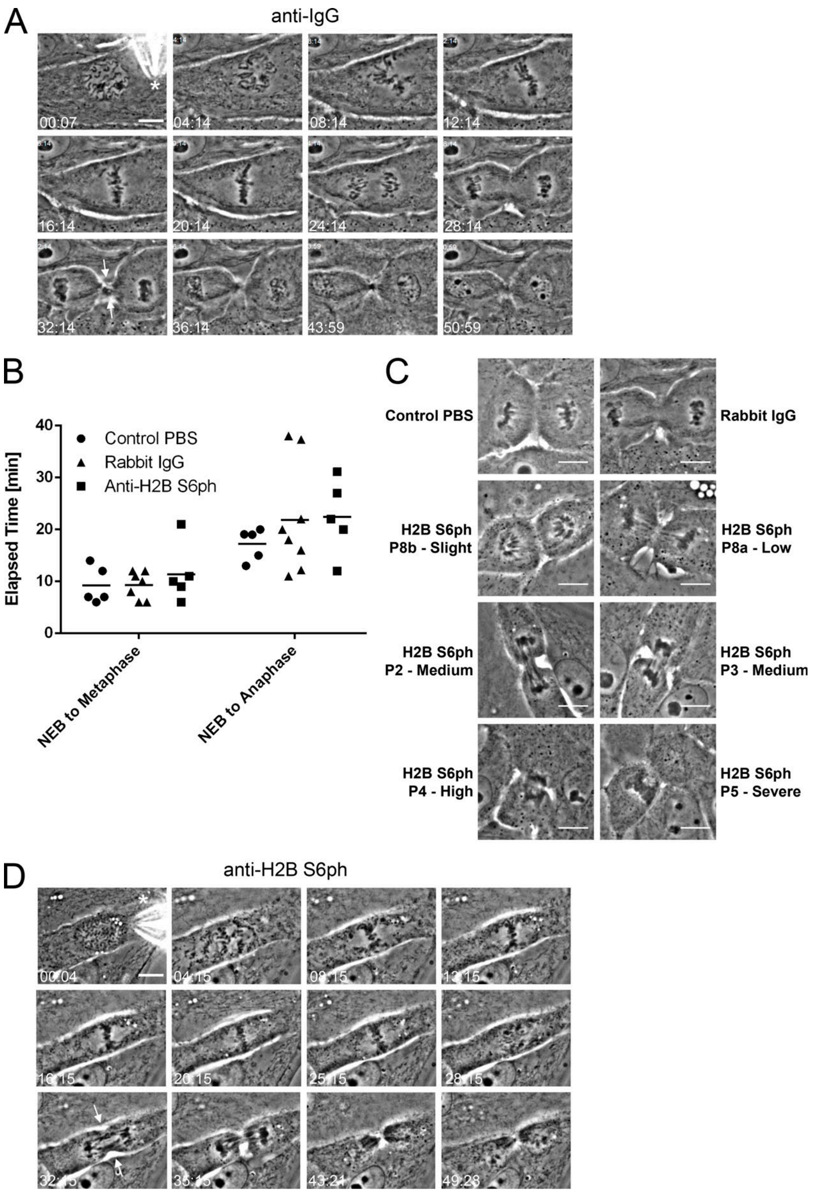

Figure S4. **Further details for the antibody injection experiments (related to Fig. 8). (A)** Prophase LLC-PK cells were injected with rabbit IgG control antibodies at the onset of mitosis and further traced by live cell imaging. Selected time points of this experiment are already displayed in Fig. 8 A. Pictures show representative phenotypes after injection of rabbit IgG. Area of microinjection is marked with an asterisk (*), the position of the cleavage furrow is indicated by arrows, and time points after antibody injection are given (min:s). **(B)** Elapsed time (in minutes) between nuclear envelope breakdown (NEB) and start of metaphase or anaphase was measured for each injected cell. Lines represent mean values for each condition. **(C)** The experiment was done as in Fig. S3 (A and D), different phenotypes with an increasing severity of the mitotic defect are shown. Phenotypes with slight or low severity were categorized by slightly compromised poleward chromosome movements and some chromosomes caught in the cleavage furrow. Medium phenotypes showed compromised chromosome movements resulting in chromosomal bridges between daughter cells, whereas in cells with high and severe phenotypes, chromosomes completely failed to segregate before cleavage furrow ingression. **(D)** The experiment was done as in Fig. S3 A, with the difference that anti-H2B S6ph antibodies were injected. Selected time points of this experiment are already displayed in Fig. 8 A. Area of microinjection is marked with an asterisk (*), the position of the cleavage furrow is indicated by arrows, and time points after antibody injection are given (min:s). Bars: 10 µm (A, C, and D).

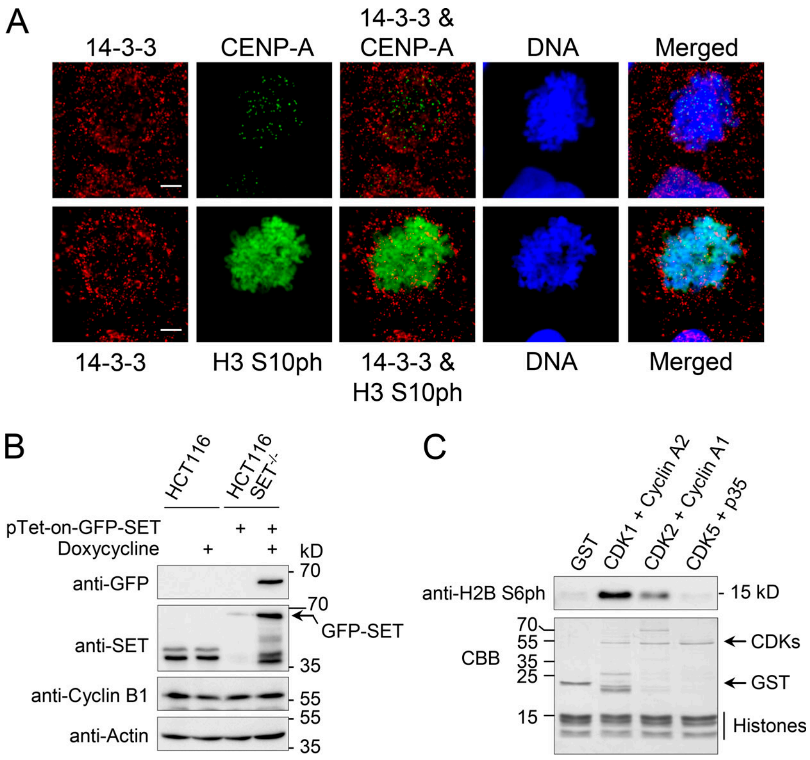

Figure S5. **Localization of 14-3-3 proteins, the inducible expression of GFP-SET, and in vitro kinase assays with recombinant CDK complexes and histone octamers as substrates. (A)** HeLa cells were arrested in prometaphase with nocodazole for 4 h; chromosome spreads were produced for immunostaining of 14-3-3 proteins and CENP-A or H3 S10ph. DNA was stained using Hoechst 33342. Bars, 5 µm. **(B)** HCT SET-deficient cells were stably transfected with a plasmid encoding tet-inducible GFP-SET. These cells or HCT116 control cells were treated for 24 h with 1 µg/ml doxycycline and analyzed by Western blotting as shown. The two lower bands in GFP-SET–expressing cells likely represent proteolytic cleavage products. **(C)** Recombinant and purified GST-tagged CDK1-cyclin A2, CDK2-cyclin A1 and CDK5-p35 (1 µg) were incubated together for in vitro kinase assay with purified histone octamers (8 µg) as substrate. The reaction was analyzed for histone phosphorylation by Western blotting (upper) and protein integrity by SDS-PAGE and CBB staining. The positions of molecular weight markers are shown.

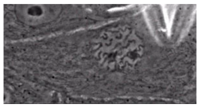

Video 1. **Mitotic effects caused by the injection of IgG control antibodies.** Prophase LLC-PK cells were injected with rabbit IgG control antibodies and further traced by live cell imaging. Video shows representative phenotype after injection of rabbit IgG (normal mitotic progression). Time stamps indicate hours:minutes:seconds.

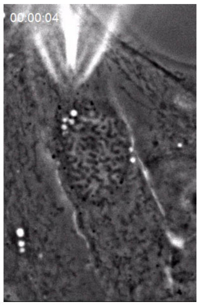

Video 2. **The mitotic effects caused by the injection of anti-H2B S6ph antibodies.** Prophase LLC-PK cells were injected with anti-H2B S6ph antibodies and further traced by live cell imaging. Video shows a representative phenotype with medium severity after injection of anti-H2B S6ph microinjection (CUT phenotype). Time stamps indicate hours:minutes:seconds.

Tables S1, S2, and S3 are included as separate Microsoft Word files. Table S1 shows the results from the siRNA kinase screen. Table S2 displays MS results for the identification of phosphorylation-dependent H2B(1–20) interactors. Table S3 lists reagents and materials.

https://doi.org/10.1083/jcb.201806057
