# Supplementary material for: CDK1-mediated phosphorylation at H2B serine 6 is required for mitotic chromosome segregation
Source: J Cell Biol. 2019 Feb 14;218(4):1164–81. doi: 10.1083/jcb.201806057 (PMC6446833; doi:10.1083/jcb.201806057)
Supplement: Tables S1-S3 (ZIP) [file JCB_201806057_TablesS1-S3.zip › JCB_201806057_TableS1.doc]

**H2BS6 kinome screen standardscores_pvalues per kinase**

| **Target** | **mean standard score** | **stdev of standard score** | **p-value** | **log p value** |
| --- | --- | --- | --- | --- |
| AURKB | -5.268813167 | 1.303238567 | 0.004 | 2.39794 |
| BUB1B | -4.458638566 | 0.503707305 | 0.0042 | 2.376751 |
| IGF1R | -2.547154885 | 0.449285504 | 0.0015 | 2.823909 |
| GPR123 | -2.290061919 | 0.699042259 | 0.0297 | 1.527244 |
| CDKL4 | -2.191658086 | 1.105547211 | 0.0287 | 1.542118 |
| MATK | -2.152558726 | 0.248763146 | 0.0004 | 3.39794 |
| ADRA2B | -2.118497055 | 0.262338809 | 0.0051 | 2.29243 |
| PCK2 | -2.046759795 | 0.36427221 | 0.0015 | 2.823909 |
| TACR2 | -2.039485445 | 0.381157065 | 0.0017 | 2.769551 |
| GPR142 | -2.035907606 | 0.462740395 | 0.1014 | 0.993962 |
| EMR3 | -2.011783991 | 0.591214333 | 0.0276 | 1.559091 |
| CDK11 | -1.998728369 | 0.219919398 | 0.0004 | 3.39794 |
| DTYMK | -1.987575609 | 0.705263276 | 0.011 | 1.958607 |
| CDC2 | -1.905966606 | 0.380526881 | 0.013 | 1.886057 |
| PI4K2B | -1.881617216 | 0.700070614 | 0.0126 | 1.899629 |
| WEE1 | -1.79274624 | 0.549908668 | 0.0073 | 2.136677 |
| DLG1 | -1.747045434 | 0.238577019 | 0.0007 | 3.154902 |
| TJP2 | -1.731105264 | 0.586808701 | 0.0362 | 1.441291 |
| RXFP3 | -1.725247378 | 0.810982794 | 0.0664 | 1.177832 |
| CDKN2C | -1.689175209 | 0.601151234 | 0.0111 | 1.954677 |
| TRIB3 | -1.688900275 | 1.351563014 | 0.0878 | 1.056505 |
| EDG2 | -1.646490332 |  |  |  |
| GPR24 | -1.624229527 | 0.010454056 |  |  |
| STK32A | -1.619939085 | 0.326346588 | 0.0022 | 2.657577 |
| CDK10 | -1.617968511 | 0.245864384 | 0.0009 | 3.045757 |
| NTRK1 | -1.608863705 | 0.657098185 | 0.0163 | 1.787812 |
| RPS6KB1 | -1.608041294 | 1.100857304 | 0.0614 | 1.211832 |
| MST1R | -1.603130244 | 0.594974489 | 0.0004 | 3.39794 |
| NRBP2 | -1.594107928 | 0.895025416 | 0.091 | 1.040959 |
| DUSP21 | -1.559309476 | 0.174277473 | 0.0004 | 3.39794 |
| DGKI | -1.519390244 | 0.874724201 | 0.0402 | 1.395774 |
| PINK1 | -1.510479271 | 1.221322367 | 0.0898 | 1.046724 |
| IHPK2 | -1.498761393 | 0.241914579 | 0.0011 | 2.958607 |
| EDG1 | -1.495332198 | 0.870925874 | 0.0414 | 1.383 |
| EEF2K | -1.456948744 | 0.198464592 | 0.0611 | 1.213959 |
| OSR1 | -1.436055437 | 0.703629588 | 0.0715 | 1.145694 |
| HTR4 | -1.428012795 | 0.693455259 | 0.0704 | 1.152427 |
| PSKH2 | -1.416212701 | 0.692549595 | 0.0264 | 1.578396 |
| KIAA1361 | -1.395972885 | 0.694458195 | 0.0735 | 1.133713 |
| FLJ32685 | -1.395401474 | 0.136717199 | 0.044 | 1.356547 |
| CNR2 | -1.365503522 | 0.253865332 | 0.0113 | 1.946922 |
| GPR75 | -1.356444394 | 0.309060235 | 0.0031 | 2.508638 |
| ADRBK2 | -1.327861175 | 0.900273373 | 0.06 | 1.221849 |
| STK38L | -1.317506451 | 0.240457206 | 0.0109 | 1.962574 |
| PIK3CA | -1.311176282 | 0.302725679 | 0.103 | 0.987163 |
| FGFR4 | -1.2924893 | 1.572547968 | 0.1988 | 0.701584 |
| ADRA1D | -1.287028949 | 0.858281549 | 0.2805 | 0.552067 |
| MAPK14 | -1.27932169 | 0.528862355 | 0.0525 | 1.279841 |
| CSNK1E | -1.276757763 | 0.40344894 | 0.008 | 2.09691 |
| NME3 | -1.266980356 | 0.738619098 | 0.0971 | 1.012781 |
| RPS6KB2 | -1.26192677 | 0.284001993 | 0.0165 | 1.782516 |
| VRK3 | -1.261506807 | 0.641671266 | 0.0293 | 1.533132 |
| EDG5 | -1.255831211 | 0.699745864 | 0.0898 | 1.046724 |
| PDPK1 | -1.255365055 | 0.53705386 | 0.0023 | 2.638272 |
| IHPK1 | -1.238284831 | 0.397676461 | 0.0083 | 2.080922 |
| TAF1 | -1.232420733 | 0.580702058 | 0.024 | 1.619789 |
| PMVK | -1.231088748 | 1.514615948 | 0.2945 | 0.530915 |
| MAPK11 | -1.226959711 | 0.029636296 | 0.0002 | 3.69897 |
| PLK4 | -1.223131048 | 0.454472371 | 0.0126 | 1.899629 |
| BAI1 | -1.21133986 | 0.488361772 | 0.0501 | 1.300162 |
| BRS3 | -1.202252441 | 0.59789745 | 0.0735 | 1.133713 |
| CDK6 | -1.19828852 | 0.506228167 | 0.0179 | 1.747147 |
| CSNK1G2 | -1.180938461 | 0.665104266 | 0.2413 | 0.617443 |
| RAGE | -1.158645958 | 0.327534791 | 0.0256 | 1.59176 |
| ERN2 | -1.149965611 | 0.564459476 | 0.0718 | 1.143876 |
| CTDSP1 | -1.14687595 | 0.635777575 | 0.0366 | 1.436519 |
| GPR37L1 | -1.140992141 | 0.41358992 | 0.0117 | 1.931814 |
| PIK3CG | -1.117782274 | 0.956394102 | 0.1015 | 0.993534 |
| ULK4 | -1.113898188 | 1.600336766 | 0.2581 | 0.588212 |
| PRKR | -1.106904156 | 0.172511884 | 0.001 | 3 |
| CDKN1B | -1.091477036 | 1.175747092 | 0.1604 | 0.794796 |
| FLT3 | -1.090504273 | 0.347572547 | 0.0082 | 2.086186 |
| AMHR2 | -1.090050118 | 0.28882847 | 0.0048 | 2.318759 |
| MELK | -1.08828803 | 0.362043487 | 0.0092 | 2.036212 |
| MC5R | -1.078417975 | 0.540412425 | 0.0745 | 1.127844 |
| CHRM4 | -1.053589593 | 0.466674163 | 0.0027 | 2.568636 |
| PTK9 | -1.052594103 | 0.787922055 | 0.1467 | 0.83357 |
| NEK9 | -1.045648372 | 0.353732378 | 0.0097 | 2.013228 |
| EFNA4 | -1.040012147 | 0.54189978 | 0.0312 | 1.505845 |
| PTGFR | -1.039244792 | 0.393302273 | 0.0132 | 1.879426 |
| RPS6KC1 | -1.031219027 | 0.568768526 | 0.0361 | 1.442493 |
| IKBKAP | -1.026192859 | 0.263065923 | 0.0044 | 2.356547 |
| ICK | -1.020886356 | 0.317272299 | 0.0076 | 2.119186 |
| LAK | -1.017397743 | 1.334031644 | 0.3174 | 0.498393 |
| FLJ25006 | -1.015878661 | 0.374507879 | 0.0424 | 1.372634 |
| EGFR | -1.010871221 | 0.412329576 | 0.0162 | 1.790485 |
| BRD4 | -1.010686028 | 0.500694224 | 0.0273 | 1.563837 |
| KIAA0999 | -1.006352512 | 0.298083551 | 0.0066 | 2.180456 |
| PIP5K3 | -1.005953138 | 1.311930512 | 0.2227 | 0.65228 |
| SYK | -1.00040445 | 0.62921358 | 0.2664 | 0.574466 |
| TRPM6 | -1.000366645 | 0.791943085 | 0.3249 | 0.48825 |
| NEK1 | -0.992821294 | 0.483687527 | 0.0262 | 1.581699 |
| FLT1 | -0.990482172 | 0.242361236 | 0.0038 | 2.420216 |
| PTGIR | -0.988132427 | 0.885027164 | 0.1928 | 0.714893 |
| PHKA1 | -0.985535463 | 0.89951224 | 0.1982 | 0.702896 |
| NEK5 | -0.984955059 | 0.694888719 | 0.1335 | 0.874519 |
| IKBKG | -0.982558699 | 0.318722456 | 0.0086 | 2.065502 |
| ELTD1 | -0.981999199 | 0.331220781 | 0.0359 | 1.444906 |
| GALK2 | -0.981396828 | 0.806012486 | 0.0929 | 1.031984 |
| TAAR8 | -0.97607075 | 0.608012093 | 0.1087 | 0.96377 |
| PHKB | -0.975329759 | 0.051605239 | 0.0238 | 1.623423 |
| ILK | -0.964706336 | 0.672729793 | 0.0642 | 1.192465 |
| TESK1 | -0.956446556 | 1.704840715 | 0.3436 | 0.463947 |
| PDK1 | -0.951899147 | 0.691189637 | 0.0705 | 1.151811 |
| TYRO3 | -0.947269565 | 0.60607601 | 0.0522 | 1.282329 |
| PIK4CB | -0.941676119 | 0.362462928 | 0.0138 | 1.860121 |
| MAP4K2 | -0.938436875 | 0.609949173 | 0.0543 | 1.2652 |
| BTK | -0.937675645 | 0.695321223 | 0.074 | 1.130768 |
| CDKN2B | -0.937606246 | 0.862413439 | 0.118 | 0.928118 |
| PAPSS1 | -0.936645548 | 0.593137 | 0.0242 | 1.616185 |
| OPRM1 | -0.932640702 | 0.385978687 | 0.0057 | 2.244125 |
| PGK1 | -0.932198343 | 0.208060785 | 0.0029 | 2.537602 |
| TTBK2 | -0.92636825 | 1.267835558 | 0.2401 | 0.619608 |
| PRKG1 | -0.914276821 | 0.540535265 | 0.0994 | 1.002614 |
| AVPR1A | -0.911974309 | 0.902505928 | 0.2222 | 0.653256 |
| CRIM1 | -0.906419424 | 0.205288894 | 0.1011 | 0.995249 |
| BMPR2 | -0.905158826 | 0.31752156 | 0.0387 | 1.412289 |
| ZAK | -0.892309149 | 0.748839047 | 0.0973 | 1.011887 |
| GPR143 | -0.88709953 | 1.019782954 | 0.2709 | 0.567191 |
| NPY2R | -0.883504003 | 0.3983722 | 0.0616 | 1.210419 |
| PAPSS2 | -0.877417737 | 1.199684426 | 0.3328 | 0.477817 |
| EMR4 | -0.877017776 | 0.756870442 | 0.3488 | 0.457424 |
| CSK | -0.876995078 | 0.622714873 | 0.0669 | 1.174574 |
| RFK | -0.875251061 | 0.077309498 | 0.0002 | 3.69897 |
| PIM2 | -0.873214335 | 0.171409052 | 0.0126 | 1.899629 |
| RPS6KA4 | -0.872550461 | 0.351411261 | 0.05 | 1.30103 |
| NME4 | -0.871332063 | 0.344453184 | 0.0016 | 2.79588 |
| ADCK5 | -0.867643106 | 1.302883985 | 0.275 | 0.560667 |
| DLG2 | -0.860744293 | 0.68054173 | 0.0855 | 1.068034 |
| CALM2 | -0.859148889 | 0.357692675 | 0.0172 | 1.764472 |
| PANK3 | -0.857561913 | 0.597339349 | 0.064 | 1.19382 |
| PKM2 | -0.852923896 | 0.534006067 | 0.1096 | 0.960189 |
| DAPK3 | -0.852654175 | 0.617913845 | 0.0702 | 1.153663 |
| MAP2K6 | -0.852347128 | 0.768183239 | 0.1131 | 0.946537 |
| CHEK1 | -0.846214503 | 0.68107718 | 0.0889 | 1.051098 |
| LTK | -0.830363195 | 1.196453591 | 0.3524 | 0.452964 |
| RFP | -0.827174201 | 0.406488165 | 0.0719 | 1.143271 |
| MAGI-3 | -0.823896312 | 0.197409062 | 0.0036 | 2.443697 |
| SGKL | -0.816808169 | 0.726214096 | 0.11 | 0.958607 |
| CHUK | -0.80990184 | 0.616925871 | 0.0786 | 1.104577 |
| INSRR | -0.802187687 | 0.423143834 | 0.0322 | 1.492144 |
| SCYL1 | -0.800776939 | 0.524442137 | 0.0553 | 1.257275 |
| FN3K | -0.80062756 | 0.89080433 | 0.2598 | 0.585361 |
| NTSR1 | -0.799306462 | 0.411932154 | 0.0783 | 1.106238 |
| SMG1 | -0.798318682 | 0.412670391 | 0.0305 | 1.5157 |
| AGTR2 | -0.795619502 | 0.561855177 | 0.0661 | 1.179799 |
| GPR113 | -0.786513284 | 0.574446596 | 0.1411 | 0.850473 |
| SRMS | -0.779139584 | 0.634694531 | 0.3327 | 0.477947 |
| GSK3B | -0.778005504 | 0.567477667 | 0.0712 | 1.14752 |
| PDGFRL | -0.746298775 | 0.336198576 | 0.0615 | 1.211125 |
| GPR74 | -0.737334166 | 1.240580729 | 0.4115 | 0.38563 |
| FSHR | -0.735572784 | 0.761432559 | 0.2363 | 0.626536 |
| NME6 | -0.735176736 | 0.311168648 | 0.018 | 1.744727 |
| GCK | -0.733652657 | 1.323029197 | 0.3483 | 0.458047 |
| STK23 | -0.724162714 | 0.847500822 | 0.186 | 0.730487 |
| PDK3 | -0.721837634 | 0.464934133 | 0.0531 | 1.274905 |
| NME7 | -0.716180273 | 0.629963033 | 0.1878 | 0.726304 |
| PRKY | -0.703520843 | 0.559387217 | 0.0865 | 1.062984 |
| PLK3 | -0.693645465 | 0.475065173 | 0.0309 | 1.510042 |
| PIP5K2A | -0.685539307 | 1.067932931 | 0.2894 | 0.538501 |
| LOC91461 | -0.678442388 | 1.178563229 | 0.333 | 0.477556 |
| TEC | -0.677499704 | 0.317011138 | 0.0235 | 1.628932 |
| PKMYT1 | -0.672419013 | 0.47284107 | 0.0654 | 1.184422 |
| SNARK | -0.669033141 | 0.6399686 | 0.1277 | 0.893809 |
| C9ORF96 | -0.664958838 | 0.316078815 | 0.0245 | 1.610834 |
| PVR | -0.662247872 | 0.660740869 | 0.2247 | 0.648397 |
| PIP5KL1 | -0.65939617 | 0.06761831 | 0.0461 | 1.336299 |
| DDR1 | -0.65786827 | 0.391782583 | 0.0438 | 1.358526 |
| PRKAB2 | -0.656195623 | 0.661980038 | 0.1417 | 0.84863 |
| MAP2K5 | -0.653510198 | 0.365070874 | 0.0902 | 1.044793 |
| CKM | -0.651678846 | 0.352265195 | 0.0851 | 1.07007 |
| LIMK1 | -0.641318805 | 0.177486164 | 0.123 | 0.910095 |
| ROR1 | -0.641189639 | 1.167858274 | 0.3524 | 0.452964 |
| HIPK1 | -0.638914094 | 0.244202717 | 0.0136 | 1.866461 |
| FLJ23074 | -0.636352613 | 0.696864859 | 0.1653 | 0.781727 |
| FRDA | -0.633056469 | 0.310743407 | 0.0267 | 1.573489 |
| GK | -0.628329108 | 0.469806328 | 0.0754 | 1.122629 |
| GPR20 | -0.627157016 | 0.225322959 | 0.0404 | 1.393619 |
| DKFZP761P0423 | -0.626837538 | 0.622169227 | 0.1373 | 0.862329 |
| MOS | -0.621796479 | 1.152253199 | 0.3595 | 0.444301 |
| CAMK4 | -0.621483264 | 1.170813506 | 0.3663 | 0.436163 |
| FPR1 | -0.613923169 | 1.075338481 | 0.3364 | 0.473144 |
| EPHB3 | -0.606724193 | 0.761444879 | 0.0796 | 1.099087 |
| PRKAR2A | -0.606092614 | 0.366636133 | 0.0455 | 1.341989 |
| ERK8 | -0.605076879 | 1.044944055 | 0.3306 | 0.480697 |
| GRM3 | -0.60316409 | 1.46373247 | 0.6641 | 0.177767 |
| CALCR | -0.598201526 | 0.689128444 | 0.2716 | 0.56607 |
| FASTK | -0.597008405 | 1.040104758 | 0.3342 | 0.475994 |
| C10ORF89 | -0.592936571 | 0.098423795 | 0.0012 | 2.920819 |
| GPR10 | -0.592302138 | 0.072052572 | 0.0546 | 1.262807 |
| RIOK2 | -0.586411822 | 0.623076944 | 0.2446 | 0.611544 |
| PFKFB3 | -0.57855737 | 0.571691421 | 0.1361 | 0.866142 |
| VN1R4 | -0.577350132 | 1.056174873 | 0.4437 | 0.352911 |
| FGFR1 | -0.572594587 | 0.80166908 | 0.2485 | 0.604674 |
| SRP72 | -0.55714851 | 0.29998037 | 0.0339 | 1.4698 |
| DMPK | -0.549890377 | 3.446822247 | 0.8082 | 0.092481 |
| FLJ34389 | -0.548666925 | 0.32690536 | 0.2538 | 0.595508 |
| NEK11 | -0.544105564 | 0.301665009 | 0.2379 | 0.623606 |
| HUS1 | -0.542264928 | 0.684505165 | 0.2113 | 0.675101 |
| PRKACG | -0.541513504 | 0.761169497 | 0.2499 | 0.602234 |
| DYRK1B | -0.535128894 | 0.673853042 | 0.3028 | 0.518844 |
| MRGPRX2 | -0.532835591 | 0.832676144 | 0.3831 | 0.416688 |
| LIMK2 | -0.531751657 | 0.552286445 | 0.1498 | 0.824488 |
| PFKFB1 | -0.531433537 | 0.31767468 | 0.0442 | 1.354578 |
| PDK2 | -0.522909256 | 0.719788698 | 0.4914 | 0.308565 |
| EPHA2 | -0.521027942 | 0.630812365 | 0.1971 | 0.705313 |
| CAMK2A | -0.518826634 | 1.191819689 | 0.5295 | 0.276134 |
| DGKB | -0.517322385 | 0.670051823 | 0.4721 | 0.325966 |
| PFTK1 | -0.513479025 | 0.153158537 | 0.0284 | 1.546682 |
| BUB1 | -0.510701201 | 0.947607332 | 0.36 | 0.443697 |
| TYK2 | -0.509315903 | 0.427549585 | 0.0974 | 1.011441 |
| STK17B | -0.508636312 | 0.171568513 | 0.0359 | 1.444906 |
| PRKCI | -0.506519234 | 1.328550726 | 0.5769 | 0.238899 |
| STK32C | -0.502481335 | 0.915897842 | 0.3527 | 0.452595 |
| PRKAG1 | -0.500708924 | 0.335144198 | 0.0582 | 1.235077 |
| FYN | -0.499159682 | 0.55513215 | 0.17 | 0.769551 |
| JIK | -0.499049302 | 1.398843618 | 0.527 | 0.278189 |
| FN3KRP | -0.493686018 | 0.185094729 | 0.0129 | 1.88941 |
| FES | -0.492882449 | 0.872020781 | 0.2248 | 0.648204 |
| MLCK | -0.470200145 | 0.313633715 | 0.1218 | 0.914353 |
| ROCK2 | -0.46904106 | 0.880442543 | 0.3648 | 0.437945 |
| TTK | -0.468776936 | 0.382277328 | 0.1676 | 0.775726 |
| KIAA1804 | -0.46777298 | 0.597380176 | 0.3078 | 0.511731 |
| GPR146 | -0.46731451 | 0.629585459 | 0.3273 | 0.485054 |
| MRGPRE | -0.463878872 | 0.686114919 | 0.3622 | 0.441052 |
| LMTK2 | -0.46219665 | 1.137806167 | 0.476 | 0.322393 |
| PRPS1L1 | -0.460743426 | 0.613817363 | 0.2303 | 0.637706 |
| FER | -0.458668065 | 0.761575649 | 0.5509 | 0.258927 |
| ADCK2 | -0.451638436 | 0.607896986 | 0.234 | 0.630784 |
| LOC390226 | -0.447813121 | 0.858730812 | 0.3736 | 0.427593 |
| URKL1 | -0.444371298 | 2.323209047 | 0.8318 | 0.079981 |
| CAMKK2 | -0.443094363 | 0.535299234 | 0.4501 | 0.346691 |
| EFNA5 | -0.44115011 | 0.379633752 | 0.1027 | 0.98843 |
| PIK3C2A | -0.440511899 | 0.755717567 | 0.328 | 0.484126 |
| KIAA1811 | -0.440391131 | 1.084531123 | 0.6681 | 0.175159 |
| EPHB2 | -0.437877276 | 0.555531724 | 0.213 | 0.67162 |
| CDK4 | -0.437052664 | 0.889901759 | 0.3984 | 0.399681 |
| MERTK | -0.428678546 | 0.305892282 | 0.0677 | 1.169411 |
| DLG3 | -0.420669605 | 0.641333996 | 0.2809 | 0.551448 |
| CDK9 | -0.41846473 | 0.306258387 | 0.1416 | 0.848937 |
| PAK3 | -0.416565619 | 1.158364941 | 0.3781 | 0.422393 |
| PHKG1 | -0.412968437 | 0.932527682 | 0.441 | 0.355561 |
| GNRHR | -0.41059957 | 0.751371814 | 0.4438 | 0.352813 |
| STK31 | -0.403122829 | 0.534722421 | 0.2287 | 0.640734 |
| LYK5 | -0.397273761 | 1.159119266 | 0.5423 | 0.26576 |
| HIPK2 | -0.395842441 | 0.847054527 | 0.4189 | 0.37789 |
| STK22C | -0.389916839 | 0.608526127 | 0.2901 | 0.537452 |
| PANK4 | -0.378877219 | 0.524692336 | 0.3375 | 0.471726 |
| FLJ23356 | -0.378112846 | 0.462793805 | 0.2008 | 0.697236 |
| GPR32 | -0.37568893 | 1.094188264 | 0.6124 | 0.212965 |
| ILK-2 | -0.373535908 | 0.584940763 | 0.2914 | 0.53551 |
| GPR56 | -0.370288272 | 0.918631852 | 0.4791 | 0.319574 |
| LRRK1 | -0.367261664 | 0.504538555 | 0.3345 | 0.475604 |
| PANK1 | -0.366925534 | 0.711671582 | 0.4661 | 0.331521 |
| PIK3R1 | -0.366121632 | 0.465832815 | 0.214 | 0.669586 |
| PDK4 | -0.365131471 | 1.298767303 | 0.6744 | 0.171082 |
| EFNB3 | -0.364231735 | 0.55350473 | 0.2797 | 0.553308 |
| MAP3K11 | -0.354825612 | 0.564946643 | 0.298 | 0.525784 |
| GPR | -0.353863428 | 0.440897645 | 0.2068 | 0.684449 |
| CKS2 | -0.352409758 | 0.363457918 | 0.2351 | 0.628747 |
| GSG2 | -0.350642009 | 0.527293362 | 0.2756 | 0.559721 |
| PGK2 | -0.349705273 | 0.604545826 | 0.4219 | 0.37479 |
| GPR77 | -0.348762133 | 0.243394154 | 0.1312 | 0.882066 |
| PRKCG | -0.347531179 | 1.178410607 | 0.5968 | 0.224171 |
| TGFBR1 | -0.344373714 | 0.537659968 | 0.2902 | 0.537303 |
| NAGK | -0.338801139 | 0.058506827 | 0.0098 | 2.008774 |
| P2RY5 | -0.338553318 | 0.836458748 | 0.5559 | 0.255003 |
| ACVR2 | -0.334785508 | 0.281330056 | 0.0976 | 1.01055 |
| TK2 | -0.32674384 | 0.231177102 | 0.0664 | 1.177832 |
| GTF2H1 | -0.325223988 | 0.757775431 | 0.4538 | 0.343136 |
| FRK | -0.324870765 | 0.583286964 | 0.3465 | 0.460297 |
| MYLK2 | -0.3157637 | 0.630152957 | 0.4769 | 0.321573 |
| GPR55 | -0.313261639 | 0.45880353 | 0.3585 | 0.445511 |
| MGC42105 | -0.311346632 | 0.985135124 | 0.5722 | 0.242452 |
| MYLK | -0.308531098 | 1.340147592 | 0.5648 | 0.248105 |
| GPR116 | -0.307198461 | 0.906788421 | 0.5466 | 0.26233 |
| LATS2 | -0.306374457 | 0.722652323 | 0.4588 | 0.338377 |
| MAP3K7IP1 | -0.305000455 | 0.524650884 | 0.329 | 0.482804 |
| LRRK2 | -0.301188638 | 0.426960819 | 0.1112 | 0.953895 |
| AK5 | -0.295559092 | 0.681594291 | 0.4496 | 0.347174 |
| DYRK4 | -0.293643238 | 0.82948375 | 0.53 | 0.275724 |
| NEK7 | -0.290288632 | 0.482373929 | 0.3151 | 0.501552 |
| CKS1B | -0.285542111 | 1.197366203 | 0.7196 | 0.142909 |
| HK3 | -0.277037194 | 0.334937442 | 0.1966 | 0.706416 |
| LGR5 | -0.274697143 | 0.920221409 | 0.5925 | 0.227312 |
| CDADC1 | -0.27320385 | 0.701871969 | 0.493 | 0.307153 |
| TAAR2 | -0.266729786 | 0.961086736 | 0.6782 | 0.168642 |
| FLJ13052 | -0.265597516 | 0.911660118 | 0.6639 | 0.177897 |
| MAST3 | -0.264510384 | 0.609979732 | 0.4496 | 0.347174 |
| ADP-GK | -0.264035056 | 0.369084163 | 0.2479 | 0.605723 |
| DGKD | -0.263716242 | 0.776687089 | 0.5458 | 0.262966 |
| CIB2 | -0.263541495 | 0.366145783 | 0.2456 | 0.609772 |
| OMG | -0.260687494 | 0.194340629 | 0.1458 | 0.836242 |
| RIPK3 | -0.256127619 | 0.482925175 | 0.3666 | 0.435808 |
| ULK2 | -0.252869001 | 1.002372576 | 0.6486 | 0.188023 |
| PIP5K1B | -0.251431945 | 0.868204491 | 0.6031 | 0.219611 |
| GPR110 | -0.250657441 | 1.015211447 | 0.7106 | 0.148375 |
| MARK2 | -0.246966889 | 0.909829795 | 0.6845 | 0.164627 |
| HIPK3 | -0.235006588 | 0.174132299 | 0.0738 | 1.131944 |
| COMMD3 | -0.232887423 | 0.69421439 | 0.5503 | 0.2594 |
| TLK2 | -0.231815991 | 0.543979795 | 0.5373 | 0.269783 |
| CARKL | -0.221280391 | 0.767397958 | 0.667 | 0.175874 |
| MAST4 | -0.220252851 | 1.330237441 | 0.7623 | 0.117874 |
| MAP3K13 | -0.218027653 | 0.659597377 | 0.5558 | 0.255081 |
| PTHR2 | -0.215570661 | 0.269781781 | 0.3006 | 0.522011 |
| MAP2K4 | -0.213150605 | 0.967276852 | 0.7394 | 0.131121 |
| HSMDPKIN | -0.210742329 | 0.678695914 | 0.5786 | 0.237622 |
| PRKD2 | -0.207945651 | 0.565940041 | 0.5157 | 0.287603 |
| STK25 | -0.20641596 | 0.398020617 | 0.4638 | 0.333669 |
| EPHA4 | -0.205339127 | 1.13729688 | 0.742 | 0.129596 |
| MAPK9 | -0.200283566 | 0.993591089 | 0.7138 | 0.146423 |
| DUSP10 | -0.196776375 | 0.735902757 | 0.6299 | 0.200728 |
| PIP5K1C | -0.196151561 | 0.361378964 | 0.3571 | 0.44721 |
| HIPK4 | -0.191722064 | 0.555186991 | 0.5394 | 0.268089 |
| CAMK2B | -0.190282743 | 0.667581947 | 0.6704 | 0.173666 |
| MUSK | -0.182614331 | 0.151220719 | 0.0946 | 1.024109 |
| XPR1 | -0.181366146 | 0.197221089 | 0.2522 | 0.598255 |
| AK2 | -0.179411431 | 0.262080815 | 0.3575 | 0.446724 |
| CDC42BPA | -0.17746848 | 1.859711163 | 0.8608 | 0.065098 |
| SNF1LK | -0.175744105 | 0.532450481 | 0.6252 | 0.203981 |
| PIK3R2 | -0.175059748 | 0.841181272 | 0.7052 | 0.151688 |
| EPHA7 | -0.174457972 | 0.391485276 | 0.4384 | 0.358129 |
| RP6-213H19.1 | -0.174193699 | 0.728625372 | 0.6652 | 0.177048 |
| HRI | -0.173147516 | 0.390092134 | 0.4401 | 0.356449 |
| TAO1 | -0.172783862 | 0.148796525 | 0.182 | 0.739929 |
| MAP2K1 | -0.170974367 | 0.882942517 | 0.6553 | 0.18356 |
| EDG7 | -0.16666296 | 0.221312908 | 0.322 | 0.492144 |
| MET | -0.164833291 | 0.053152581 | 0.0084 | 2.075721 |
| GNE | -0.159359676 | 0.234119368 | 0.2666 | 0.57414 |
| PFKM | -0.156751984 | 0.255491538 | 0.3073 | 0.512437 |
| ADRB2 | -0.154026375 | 1.615952275 | 0.884 | 0.053548 |
| GIPR | -0.150681906 | 1.137127806 | 0.8082 | 0.092481 |
| RAF1 | -0.148173097 | 0.826779777 | 0.7438 | 0.128544 |
| BMPR1A | -0.144583375 | 0.41243458 | 0.707 | 0.150581 |
| CYSLTR1 | -0.143492589 | 0.796742718 | 0.6775 | 0.169091 |
| ROCK1 | -0.141450129 | 0.148622062 | 0.241 | 0.617983 |
| PRKAR2B | -0.141083111 | 0.982407587 | 0.7642 | 0.116793 |
| ADK | -0.140559111 | 0.740072315 | 0.7294 | 0.137034 |
| FZD3 | -0.139182894 | 0.357118389 | 0.5692 | 0.244735 |
| LATS1 | -0.129010189 | 0.589894677 | 0.7413 | 0.130006 |
| MAP3K14 | -0.127744862 | 0.750755589 | 0.796 | 0.099087 |
| LOC340156 | -0.123272326 | 1.125019389 | 0.8406 | 0.075411 |
| ZAP70 | -0.121503722 | 1.215782219 | 0.8544 | 0.068339 |
| MAP3K2 | -0.120882782 | 0.660307131 | 0.7386 | 0.131591 |
| CDK2 | -0.120721958 | 0.587535132 | 0.7087 | 0.149538 |
| MAPKAPK2 | -0.116506864 | 0.197899732 | 0.4151 | 0.381847 |
| ADRB3 | -0.111217628 | 0.313201896 | 0.5288 | 0.276709 |
| UMP-CMPK | -0.097053947 | 0.794694167 | 0.8228 | 0.084706 |
| NYD-SP25 | -0.094927692 | 0.885491065 | 0.844 | 0.073658 |
| NEK6 | -0.094300204 | 0.36573463 | 0.6417 | 0.192668 |
| MULK | -0.091933931 | 0.387105702 | 0.7937 | 0.100344 |
| ATR | -0.091713354 | 0.846998494 | 0.8685 | 0.06123 |
| IKBKE | -0.090930129 | 0.257744445 | 0.6034 | 0.219395 |
| MAPKAPK3 | -0.090332959 | 0.277592595 | 0.5616 | 0.250573 |
| MAPK4 | -0.089310913 | 1.151285891 | 0.8568 | 0.067121 |
| PRKAG3 | -0.086321212 | 1.116571184 | 0.9057 | 0.043016 |
| HK2 | -0.0793145 | 0.813727237 | 0.8815 | 0.054778 |
| LCK | -0.075349356 | 0.628365135 | 0.8259 | 0.083073 |
| CSNK1A1 | -0.072430805 | 0.448940613 | 0.7681 | 0.114582 |
| NUCKS | -0.072115987 | 0.70630348 | 0.8513 | 0.069917 |
| PTK9L | -0.071414818 | 1.007823258 | 0.869 | 0.06098 |
| TRIO | -0.07053732 | 1.27324863 | 0.9323 | 0.030444 |
| GPR107 | -0.067765357 | 0.520424854 | 0.8425 | 0.07443 |
| PASK | -0.066108606 | 1.349627634 | 0.9281 | 0.032405 |
| GPBAR1 | -0.064536129 | 0.05286562 | 0.3343 | 0.475864 |
| CRK7 | -0.064384876 | 1.182732863 | 0.9202 | 0.036118 |
| PRKCSH | -0.063567456 | 0.788963941 | 0.8822 | 0.054433 |
| NRBP | -0.062980389 | 0.253027623 | 0.6528 | 0.18522 |
| PXK | -0.055934447 | 0.813505817 | 0.8729 | 0.059036 |
| CHEK2 | -0.054436017 | 1.02536001 | 0.9221 | 0.035222 |
| PKN3 | -0.053504229 | 1.051128019 | 0.9378 | 0.02789 |
| EDG3 | -0.052908218 | 0.422365332 | 0.8484 | 0.071399 |
| PRKG2 | -0.051097656 | 0.335421617 | 0.7805 | 0.107627 |
| PRPS1 | -0.050511367 | 1.45393873 | 0.949 | 0.022734 |
| STK32B | -0.050508115 | 0.577769537 | 0.8723 | 0.059334 |
| BMX | -0.038504508 | 1.12284315 | 0.9496 | 0.022459 |
| SPHK2 | -0.036823527 | 1.165738099 | 0.9536 | 0.020634 |
| GPR156 | -0.031574945 | 0.664015372 | 0.9419 | 0.025995 |
| PIM3 | -0.031521428 | 0.759093917 | 0.9229 | 0.034845 |
| PIP5K1A | -0.030905133 | 0.212139062 | 0.8244 | 0.083862 |
| CERK | -0.023845666 | 0.366837351 | 0.9048 | 0.043447 |
| HTR2A | -0.021084125 | 0.921126875 | 0.9575 | 0.018861 |
| BRD3 | -0.018729192 | 0.622232047 | 0.9729 | 0.011932 |
| CDK5R1 | -0.016476464 | 0.741083538 | 0.9673 | 0.014439 |
| MAP2K2 | -0.008009735 | 0.510058941 | 0.9769 | 0.01015 |
| PACE-1 | -0.001414902 | 0.233710494 | 0.9911 | 0.003883 |
| BAIAP1 | -0.0009571 | 0.404008816 | 0.9965 | 0.001523 |
| DYRK1A | 0.000919121 | 0.89437909 | 0.9985 | 0.000652 |
| ERBB2 | 0.005654034 | 0.537527828 | 0.9845 | 0.006784 |
| MAP3K6 | 0.008251805 | 1.116541843 | 0.9891 | 0.00476 |
| PIK3C2B | 0.008595612 | 0.500372849 | 0.9748 | 0.011084 |
| IPMK | 0.013391917 | 0.622557513 | 0.96 | 0.017729 |
| PRKCD | 0.01346515 | 0.100162054 | 0.8055 | 0.093934 |
| PDIK1L | 0.016517788 | 0.577739042 | 0.958 | 0.018634 |
| P2RY14 | 0.019578777 | 0.654671383 | 0.9634 | 0.016193 |
| PCK1 | 0.021776495 | 1.337851451 | 0.9761 | 0.010506 |
| PIK3R4 | 0.024753411 | 0.934537647 | 0.9611 | 0.017231 |
| ADCK4 | 0.025322547 | 0.802405922 | 0.9536 | 0.020634 |
| EPHA10 | 0.030163132 | 0.478894076 | 0.9231 | 0.034751 |
| CDC7 | 0.03019609 | 0.566034468 | 0.9348 | 0.029281 |
| EPHB4 | 0.032697299 | 0.131893078 | 0.6541 | 0.184356 |
| FLJ10761 | 0.036095236 | 0.916158012 | 0.9422 | 0.025857 |
| ABL1 | 0.036558757 | 0.370678334 | 0.8562 | 0.067425 |
| HCK | 0.03904919 | 0.732007729 | 0.9348 | 0.029281 |
| KIAA2002 | 0.040120131 | 1.636615386 | 0.97 | 0.013228 |
| TESK2 | 0.040543979 | 0.995117168 | 0.9402 | 0.02678 |
| FGR | 0.041274803 | 0.600890372 | 0.8994 | 0.046047 |
| TGFBR3 | 0.041368344 | 1.287215323 | 0.9528 | 0.020998 |
| ULK1 | 0.042038541 | 0.827982063 | 0.9255 | 0.033624 |
| DOT1L | 0.048666906 | 0.154186699 | 0.6394 | 0.194227 |
| OPN1MW | 0.050717973 | 1.008437873 | 0.9385 | 0.027566 |
| CCR7 | 0.058347868 | 0.852175947 | 0.8998 | 0.045854 |
| NT5M | 0.059846574 | 1.592314505 | 0.9448 | 0.02466 |
| RPS6KA5 | 0.061509719 | 0.842101081 | 0.8931 | 0.0491 |
| CHKA | 0.064646693 | 1.132602114 | 0.9046 | 0.043543 |
| MAP4K4 | 0.065257948 | 0.815093899 | 0.9024 | 0.044601 |
| PIK3CB | 0.067472148 | 0.474608893 | 0.7947 | 0.099797 |
| SRPK1 | 0.068188698 | 1.367448296 | 0.9268 | 0.033014 |
| GUCY2D | 0.068201913 | 1.383601109 | 0.9397 | 0.027011 |
| PTGDR | 0.069768717 | 0.521681796 | 0.8384 | 0.076549 |
| PRKCE | 0.070098518 | 0.634602584 | 0.8659 | 0.062532 |
| DYRK2 | 0.073541036 | 0.4307033 | 0.7553 | 0.121881 |
| PDGFRA | 0.074084871 | 0.759038974 | 0.8577 | 0.066665 |
| CSNK1G3 | 0.075789295 | 0.045771371 | 0.1031 | 0.986741 |
| SBK1 | 0.07713259 | 1.43988548 | 0.9345 | 0.029421 |
| PNCK | 0.087703679 | 1.02004609 | 0.8953 | 0.048031 |
| GABBR1 | 0.096503635 | 0.730199297 | 0.8824 | 0.054335 |
| MAPK3 | 0.098912935 | 0.428657772 | 0.6758 | 0.170182 |
| IRAK3 | 0.103336817 | 0.605088034 | 0.7552 | 0.121938 |
| YES1 | 0.104111029 | 0.886839129 | 0.8295 | 0.081184 |
| STYK1 | 0.104632098 | 0.924944698 | 0.8628 | 0.06409 |
| INSR | 0.105033463 | 0.562699901 | 0.7337 | 0.134481 |
| TPK1 | 0.10622858 | 0.827858975 | 0.8141 | 0.089322 |
| LYN | 0.107704931 | 1.06546577 | 0.8771 | 0.056951 |
| AAK1 | 0.114663426 | 1.264506788 | 0.8676 | 0.06168 |
| C7ORF2 | 0.117140006 | 0.766462497 | 0.816 | 0.08831 |
| MAP3K4 | 0.117257611 | 0.581221472 | 0.7601 | 0.119129 |
| PIK3C3 | 0.118277227 | 0.716908346 | 0.7631 | 0.117419 |
| ACVR2B | 0.118417344 | 0.885065659 | 0.8064 | 0.093449 |
| OPN3 | 0.127203229 | 0.910361661 | 0.8313 | 0.080242 |
| DGKK | 0.127759732 | 0.97688161 | 0.8106 | 0.091193 |
| PTK2B | 0.128884135 | 0.58685548 | 0.6902 | 0.161025 |
| LTB4R | 0.129346029 | 0.624200649 | 0.7064 | 0.150949 |
| PRKAR1B | 0.132559188 | 0.413721862 | 0.5672 | 0.246264 |
| TGFBR2 | 0.133655197 | 0.769548197 | 0.7513 | 0.124187 |
| BLK | 0.137153836 | 1.930418582 | 0.896 | 0.047692 |
| TNIK | 0.138043016 | 0.877732983 | 0.8109 | 0.091033 |
| RBKS | 0.139658663 | 1.169572267 | 0.8553 | 0.067882 |
| FFAR1 | 0.141179251 | 0.9238229 | 0.8645 | 0.063235 |
| STK3 | 0.142317059 | 0.596387344 | 0.6658 | 0.176656 |
| ITPKA | 0.142416915 | 0.939765018 | 0.7816 | 0.107015 |
| MAPK1 | 0.147789634 | 1.069823961 | 0.8003 | 0.096747 |
| BRAF | 0.15044421 | 0.805393805 | 0.7335 | 0.1346 |
| RPS6KA3 | 0.154922176 | 0.266792956 | 0.3295 | 0.482145 |
| DUSTYPK | 0.160005328 | 0.630923034 | 0.6469 | 0.189163 |
| STK29 | 0.16537964 | 0.782175665 | 0.7009 | 0.154344 |
| STK4 | 0.167986674 | 0.083971028 | 0.028 | 1.552842 |
| BMP2K | 0.17069742 | 1.172915861 | 0.8245 | 0.083809 |
| MYO3B | 0.177142705 | 0.448870151 | 0.5648 | 0.248105 |
| PKIA | 0.179958305 | 0.495647601 | 0.5937 | 0.226433 |
| CDK3 | 0.180242251 | 1.321708136 | 0.8027 | 0.095447 |
| SPEG | 0.18032646 | 0.20019372 | 0.4237 | 0.372942 |
| BMPR1B | 0.1889087 | 0.964113042 | 0.7666 | 0.115431 |
| N4BP2 | 0.193024409 | 1.492563632 | 0.7439 | 0.128485 |
| PFKP | 0.198614477 |  |  |  |
| ETNK1 | 0.200694132 | 0.688459272 | 0.7511 | 0.124302 |
| PFKFB2 | 0.20234953 | 0.64939651 | 0.5773 | 0.238598 |
| CDK7 | 0.203413341 | 1.111675876 | 0.7813 | 0.107182 |
| PNKP | 0.208450003 | 0.522106077 | 0.483 | 0.316053 |
| MAPK10 | 0.208457073 | 0.888166351 | 0.6708 | 0.173407 |
| ADRA1B | 0.211836652 | 0.491587185 | 0.4522 | 0.344669 |
| DGKG | 0.211994732 | 0.508912428 | 0.4659 | 0.331707 |
| CDK5 | 0.215997034 | 0.757413093 | 0.6084 | 0.215811 |
| PRKCA | 0.218494121 | 0.61986292 | 0.4274 | 0.369165 |
| EPHA1 | 0.219098401 | 1.141636989 | 0.7267 | 0.138645 |
| PSKH1 | 0.223738851 | 0.918287451 | 0.7888 | 0.103033 |
| MYO3A | 0.227068384 | 0.648588863 | 0.5342 | 0.272296 |
| MAP4K3 | 0.232037003 | 0.796109327 | 0.5072 | 0.294821 |
| MKNK1 | 0.232136397 | 0.859948077 | 0.6268 | 0.202871 |
| STK38 | 0.232308162 | 1.409731053 | 0.7634 | 0.117248 |
| CLK4 | 0.244852916 | 0.799000498 | 0.6486 | 0.188023 |
| GPR42 | 0.248682498 | 0.581257219 | 0.6536 | 0.184688 |
| GALK1 | 0.252375823 | 0.758274751 | 0.5532 | 0.257118 |
| TRIB1 | 0.252517535 | 0.796082925 | 0.6379 | 0.195247 |
| KIAA1765 | 0.254043864 | 0.648653241 | 0.4906 | 0.309272 |
| RNASEL | 0.255086161 | 0.960001843 | 0.6905 | 0.160836 |
| CDC2L2 | 0.258845133 | 0.538029626 | 0.4069 | 0.390512 |
| CDC2L5 | 0.262201582 | 0.642461911 | 0.4741 | 0.32413 |
| PRKAG2 | 0.265271412 | 0.123636131 | 0.0233 | 1.632644 |
| ADRBK1 | 0.26563861 | 0.578848643 | 0.4264 | 0.370183 |
| FLJ32332 | 0.267199812 | 0.384670269 | 0.2589 | 0.586868 |
| MAPK6 | 0.274707806 | 0.628384386 | 0.4463 | 0.350373 |
| DDR2 | 0.27645253 | 0.652023167 | 0.4587 | 0.338471 |
| RET | 0.285346064 | 1.366974319 | 0.7523 | 0.123609 |
| HSPB8 | 0.289122695 | 0.766516156 | 0.5054 | 0.296365 |
| PIK3R3 | 0.292642295 | 0.54058996 | 0.3582 | 0.445874 |
| CDKL5 | 0.293144088 | 0.627174272 | 0.5032 | 0.298259 |
| AKT2 | 0.301213023 | 0.585194556 | 0.379 | 0.421361 |
| VRK2 | 0.301219009 | 0.591786651 | 0.3836 | 0.416121 |
| KIAA1639 | 0.302017017 | 0.33073038 | 0.4195 | 0.377268 |
| WNK4 | 0.308793724 | 1.147503927 | 0.6278 | 0.202179 |
| PRPF4B | 0.310686247 | 0.91890343 | 0.5474 | 0.261695 |
| EFNA3 | 0.311199128 | 0.837050077 | 0.5111 | 0.291494 |
| FUK | 0.311929923 | 1.20546143 | 0.6405 | 0.193481 |
| DCK | 0.312898365 | 0.446244773 | 0.2554 | 0.592779 |
| VRK1 | 0.319186072 | 0.103239139 | 0.008500001 | 2.070581 |
| PAK2 | 0.320491681 | 0.753976377 | 0.4577 | 0.339419 |
| TLK1 | 0.322480774 | 1.132781199 | 0.609 | 0.215383 |
| CAMK1D | 0.325231212 | 0.749168911 | 0.4491 | 0.347657 |
| IRAK4 | 0.326069111 | 0.932875332 | 0.5348 | 0.271809 |
| CKMT2 | 0.328164291 | 0.744548362 | 0.443 | 0.353596 |
| CDKL1 | 0.328352827 | 1.144405904 | 0.6685 | 0.174899 |
| SGK | 0.328496547 | 0.493445854 | 0.2752 | 0.560352 |
| AKT3 | 0.331938679 | 1.001652075 | 0.5548 | 0.255864 |
| SPHK1 | 0.332428661 | 0.66069812 | 0.3884 | 0.410721 |
| MASTL | 0.334605462 | 0.580262061 | 0.4231 | 0.373557 |
| TNNI3K | 0.338202528 | 1.51775924 | 0.6444 | 0.190844 |
| SGK2 | 0.342815287 | 0.67851207 | 0.3867 | 0.412626 |
| CKB | 0.344834575 | 0.379119601 | 0.2558 | 0.592099 |
| MAP3K12 | 0.345399463 | 0.700095092 | 0.3965 | 0.401757 |
| PCTK3 | 0.347171031 | 0.541096935 | 0.2896 | 0.538201 |
| JAK1 | 0.356960748 | 0.618338248 | 0.3319 | 0.478993 |
| RIOK1 | 0.357028109 | 0.740173199 | 0.4914 | 0.308565 |
| BCKDK | 0.358381055 | 0.991234999 | 0.5219 | 0.282413 |
| COASY | 0.360372954 | 0.130121638 | 0.0408 | 1.38934 |
| LHCGR | 0.368380412 | 0.939960061 | 0.4903 | 0.309538 |
| CALM1 | 0.369552855 | 0.423742646 | 0.27 | 0.568636 |
| CDKL3 | 0.372232203 | 0.307455573 | 0.1709 | 0.767258 |
| PPP1R3C | 0.374627269 | 0.703435145 | 0.3649 | 0.437826 |
| ERBB4 | 0.375115061 | 0.864219044 | 0.4492 | 0.34756 |
| PIK3CD | 0.376129097 | 0.627495927 | 0.3167 | 0.499352 |
| MAPK8 | 0.380267946 | 0.675629595 | 0.3422 | 0.46572 |
| PTK7 | 0.387117546 | 1.430408546 | 0.6259 | 0.203495 |
| ITPK1 | 0.387598385 | 0.71901 | 0.3599 | 0.443818 |
| STK33 | 0.389542333 | 0.48707763 | 0.3003 | 0.522445 |
| PACSIN1 | 0.389704793 | 1.103285061 | 0.5308 | 0.275069 |
| ASK | 0.39795453 | 0.763367803 | 0.3738 | 0.427361 |
| ANKK1 | 0.400125275 | 0.756537466 | 0.4563 | 0.34075 |
| CDKL2 | 0.400361842 | 0.683794683 | 0.3261 | 0.486649 |
| MPP1 | 0.404709061 | 0.927282954 | 0.447 | 0.349692 |
| MKNK2 | 0.410019766 | 0.630092915 | 0.2193 | 0.658961 |
| CXCR6 | 0.41064385 | 1.996939717 | 0.8198 | 0.086292 |
| PHKA2 | 0.420019157 | 0.579005319 | 0.3358 | 0.473919 |
| GPR133 | 0.420393453 | 1.905152509 | 0.8074 | 0.092911 |
| ALS2CR7 | 0.422140332 | 1.477718137 | 0.6698 | 0.174055 |
| CSNK1A1L | 0.423519463 | 0.48091321 | 0.2667 | 0.573977 |
| SSTK | 0.423927157 | 0.95914635 | 0.3789 | 0.421475 |
| GOLGA5 | 0.426261787 | 1.393080716 | 0.6491 | 0.187688 |
| DUSP11 | 0.427627244 | 0.671376835 | 0.2924 | 0.534023 |
| PRKCL1 | 0.428423068 | 1.32699685 | 0.6323 | 0.199077 |
| TAF1L | 0.431581356 | 0.909702069 | 0.4127 | 0.384366 |
| TEK | 0.435899641 | 0.899496605 | 0.4896 | 0.310159 |
| ALK | 0.439175402 | 0.761645763 | 0.3324 | 0.478339 |
| HAK | 0.445942943 | 0.505292277 | 0.1757 | 0.755228 |
| MAP3K9 | 0.447871917 | 0.038087573 | 0.0024 | 2.619789 |
| PTPRJ | 0.457770496 | 0.669135242 | 0.2647 | 0.577246 |
| RIPK2 | 0.462944405 | 1.759144098 | 0.6932 | 0.159141 |
| MPP3 | 0.469103546 | 0.806240294 | 0.3287 | 0.4832 |
| ACVRL1 | 0.471466571 | 0.652594367 | 0.3373 | 0.471984 |
| TSKS | 0.475368732 | 0.730569153 | 0.2841 | 0.546529 |
| MAPK7 | 0.477744241 | 0.94097825 | 0.4719 | 0.32615 |
| BDKRB2 | 0.479388261 | 1.360976685 | 0.475 | 0.323306 |
| PRKCH | 0.480792628 | 0.510899885 | 0.1564 | 0.805763 |
| ITPKB | 0.482828883 | 0.864775894 | 0.3455 | 0.461552 |
| EXOSC10 | 0.483617339 | 0.859517421 | 0.4326 | 0.363913 |
| OR1A1 | 0.487266503 | 0.186908912 | 0.0457 | 1.340084 |
| DAPK1 | 0.487582329 | 0.77591921 | 0.2978 | 0.526075 |
| CSNK1D | 0.48819232 | 1.060057246 | 0.4249 | 0.371713 |
| NME1 | 0.489099146 | 1.018785364 | 0.4078 | 0.389553 |
| PRPS2 | 0.490064817 | 0.648023126 | 0.2276 | 0.642828 |
| DYRK3 | 0.49443048 | 0.448433357 | 0.1146 | 0.940815 |
| EPHA5 | 0.496457973 | 0.548901593 | 0.2577 | 0.588886 |
| FGFR3 | 0.49767795 | 0.726187711 | 0.264 | 0.578396 |
| KALRN | 0.497706873 | 0.634330231 | 0.2146 | 0.66837 |
| ITPKC | 0.497956156 | 1.419756853 | 0.7069 | 0.150642 |
| CCRL1 | 0.500980358 | 0.167956923 | 0.1482 | 0.829152 |
| NEK2 | 0.502660799 | 0.50715593 | 0.1417 | 0.84863 |
| NPY | 0.503282241 | 0.286716152 | 0.0933 | 1.030118 |
| MAK | 0.513437753 | 1.113623771 | 0.31 | 0.508638 |
| PANK2 | 0.513560982 | 0.298542592 | 0.0412 | 1.385103 |
| PIP5K2B | 0.514695656 | 0.740873837 | 0.2589 | 0.586868 |
| STK36 | 0.516640109 | 1.246640923 | 0.468 | 0.329754 |
| PAK7 | 0.517142865 | 0.504351837 | 0.0537 | 1.270026 |
| EPHB1 | 0.517515512 | 1.787361865 | 0.6031 | 0.219611 |
| PRKWNK2 | 0.520421507 | 0.243624932 | 0.2035 | 0.691436 |
| CIT | 0.521155087 | 0.79576069 | 0.2815 | 0.550522 |
| RPS6KA1 | 0.52172583 | 0.871234205 | 0.317 | 0.498941 |
| BRDT | 0.522244372 | 0.613282744 | 0.1871 | 0.727926 |
| KUB3 | 0.524006227 | 0.69243582 | 0.2274 | 0.64321 |
| THNSL1 | 0.524234394 | 1.190439788 | 0.5253 | 0.279593 |
| ACVR1B | 0.527457765 | 0.174374399 | 0.0091 | 2.040959 |
| CAMK2G | 0.529549479 | 1.076359991 | 0.3977 | 0.400444 |
| EDNRB | 0.538702289 | 0.890756213 | 0.5496 | 0.259953 |
| GUCY2C | 0.541047183 | 1.513308912 | 0.5262 | 0.278849 |
| IKBKB | 0.542410618 | 1.239283391 | 0.4458 | 0.35086 |
| EPHB6 | 0.543996825 | 0.855573393 | 0.2931 | 0.532984 |
| PHKG2 | 0.545331031 | 1.348943949 | 0.5563 | 0.254691 |
| PTPN5 | 0.547815153 | 0.966036581 | 0.3392 | 0.469544 |
| CSNK2A2 | 0.554290782 | 0.726010675 | 0.2242 | 0.649364 |
| P101-PI3K | 0.554875323 | 0.779610273 | 0.2498 | 0.602408 |
| RPS6KA2 | 0.558329748 | 0.755647634 | 0.236 | 0.627088 |
| BRD2 | 0.558738412 | 1.061308385 | 0.3697 | 0.432151 |
| GPR61 | 0.560453368 | 1.773204069 | 0.639 | 0.194499 |
| PLK2 | 0.564033019 | 1.015207199 | 0.576 | 0.239578 |
| CDKN1C | 0.565357453 | 0.15916501 | 0.0057 | 2.244125 |
| CAMK2D | 0.565662341 | 0.932926059 | 0.5488 | 0.260586 |
| GAK | 0.569172061 | 1.436123184 | 0.4859 | 0.313453 |
| GPR62 | 0.569555721 | 0.654856372 | 0.2709 | 0.567191 |
| EPHA8 | 0.573211763 | 0.295581078 | 0.2226 | 0.652475 |
| SGPP1 | 0.579960564 | 0.410705719 | 0.0665 | 1.177178 |
| ROS1 | 0.580581024 | 0.942879818 | 0.3059 | 0.514421 |
| KSR | 0.581227098 | 0.254521924 | 0.0584 | 1.233587 |
| OPRK1 | 0.581285486 | 2.065259782 | 0.6741 | 0.171276 |
| KIT | 0.582229329 | 0.791121676 | 0.1752 | 0.756466 |
| C14ORF20 | 0.584683836 | 0.413893142 | 0.0664 | 1.177832 |
| GPR31 | 0.590099588 | 0.714944802 | 0.1974 | 0.704653 |
| TP53RK | 0.593929407 | 1.006637276 | 0.323 | 0.490797 |
| PAK4 | 0.597893922 | 0.90594004 | 0.2785 | 0.555175 |
| SIK2 | 0.601139141 | 0.857732389 | 0.2556 | 0.592439 |
| GUK1 | 0.601507896 | 0.741818206 | 0.1038 | 0.983803 |
| MARK3 | 0.604276808 | 1.049974942 | 0.3331 | 0.477425 |
| GPR35 | 0.604325776 | 0.677914331 | 0.1726 | 0.762959 |
| ATM | 0.610671982 | 0.223068323 | 0.0417 | 1.379864 |
| C9ORF12 | 0.611051834 | 0.624127656 | 0.1451 | 0.838333 |
| EIF2AK3 | 0.615078846 | 0.368779917 | 0.0445 | 1.35164 |
| DLG4 | 0.615662676 | 0.892082108 | 0.2614 | 0.582694 |
| SCAP1 | 0.6197627 | 1.486545251 | 0.4656 | 0.331987 |
| GRK6 | 0.619794957 | 1.117017787 | 0.4379 | 0.358625 |
| MPP2 | 0.621183023 | 0.214104488 | 0.0102 | 1.9914 |
| MAST2 | 0.623867738 | 1.198373753 | 0.3743 | 0.42678 |
| SRPK2 | 0.624298208 | 0.833015043 | 0.2309 | 0.636576 |
| RPS6KL1 | 0.629604558 | 1.305432467 | 0.4059 | 0.391581 |
| PCTK1 | 0.63173946 | 0.675637784 | 0.2468 | 0.607655 |
| ITK | 0.640878483 | 1.152923935 | 0.3473 | 0.459295 |
| TBK1 | 0.642009734 | 1.197223164 | 0.5869 | 0.231436 |
| PAK1 | 0.644151179 | 0.860212852 | 0.2311 | 0.6362 |
| ADMR | 0.644704759 | 0.545541609 | 0.1773 | 0.751291 |
| SNRK | 0.658267154 | 0.933580844 | 0.3464 | 0.460422 |
| HK1 | 0.663649366 | 0.931324185 | 0.4975 | 0.303207 |
| PI4KII | 0.6671388 | 0.961379875 | 0.2593 | 0.586197 |
| CPNE3 | 0.668160432 | 0.722191506 | 0.1614 | 0.792096 |
| JAK2 | 0.672020327 | 1.054897674 | 0.2924 | 0.534023 |
| TAAR9 | 0.672387864 | 0.903353537 | 0.3263 | 0.486383 |
| IRAK2 | 0.675305957 | 0.860132847 | 0.2144 | 0.668775 |
| MGC4796 | 0.680656542 | 0.607698252 | 0.1919 | 0.716925 |
| AK1 | 0.684609176 | 0.791084709 | 0.2726 | 0.564474 |
| MAP2K3 | 0.68969262 | 1.198890529 | 0.3333 | 0.477165 |
| RYK | 0.692168707 | 0.392106402 | 0.0386 | 1.413413 |
| MAPK12 | 0.693060179 | 0.767059063 | 0.1685 | 0.7734 |
| PTK2 | 0.699271659 | 0.747314711 | 0.2465 | 0.608183 |
| PLK1 | 0.699623796 | 1.003937377 | 0.3508 | 0.45494 |
| NEK4 | 0.70182287 | 0.395365927 | 0.0915 | 1.038579 |
| SAST | 0.701833179 | 1.278882467 | 0.2366 | 0.625985 |
| MAPKAPK5 | 0.704044828 | 0.584308489 | 0.095 | 1.022276 |
| TNK1 | 0.709264828 | 0.931121552 | 0.225 | 0.647817 |
| MRGPRX4 | 0.709548859 | 0.820818066 | 0.273 | 0.563837 |
| TOPK | 0.71558792 | 0.615821252 | 0.1027 | 0.98843 |
| PRKACA | 0.723213025 | 1.232666946 | 0.3253 | 0.487716 |
| PRKAA2 | 0.724168629 | 0.756576161 | 0.1515 | 0.819587 |
| AURKC | 0.725513136 | 0.59244689 | 0.168 | 0.774691 |
| GPR78 | 0.726104722 | 0.283342985 | 0.0472 | 1.326058 |
| PPP3CA | 0.735319487 | 0.361008261 | 0.0267 | 1.573489 |
| PRKAB1 | 0.740089295 | 0.22403008 | 0.0071 | 2.148742 |
| GSK3A | 0.740753483 | 1.372002422 | 0.3593 | 0.444543 |
| MAP3K10 | 0.743522414 | 0.654449248 | 0.1077 | 0.967784 |
| CLK3 | 0.749371668 | 0.328413017 | 0.0197 | 1.705534 |
| KHK | 0.749847459 | 1.60216375 | 0.4183 | 0.378512 |
| ALPPL2 | 0.750957679 | 0.32736813 | 0.0195 | 1.709965 |
| PRKACB | 0.755524597 | 1.762185248 | 0.4542 | 0.342753 |
| CALM3 | 0.757337639 | 0.71460659 | 0.0768 | 1.114639 |
| MGC16169 | 0.765522799 | 1.182772846 | 0.2861 | 0.543482 |
| AK3L1 | 0.767000049 | 0.509716048 | 0.0572 | 1.242604 |
| DGKQ | 0.769175251 | 0.308764656 | 0.0155 | 1.809668 |
| CHKB | 0.769430243 | 0.432375112 | 0.0378 | 1.422508 |
| IHPK3 | 0.769741762 | 1.069040011 | 0.3386 | 0.470313 |
| NUP62 | 0.776409273 | 1.131145622 | 0.2634 | 0.579384 |
| GK2 | 0.778450344 | 0.305518922 | 0.0146 | 1.835647 |
| STK24 | 0.782286907 | 0.094107711 | 0.0048 | 2.318759 |
| GPRC6A | 0.788854724 | 0.718231404 | 0.1975 | 0.704433 |
| MGC8407 | 0.789828272 | 1.133456753 | 0.2577 | 0.588886 |
| NME2 | 0.80014333 | 0.5379521 | 0.0588 | 1.230623 |
| STK19 | 0.80512315 | 1.029499256 | 0.2157 | 0.66615 |
| NME5 | 0.808612926 | 0.610809515 | 0.3123 | 0.505428 |
| PDGFRB | 0.809045832 | 0.735961934 | 0.1153 | 0.938171 |
| MAP4K5 | 0.82306048 | 0.349769583 | 0.0553 | 1.257275 |
| NLK | 0.825004688 | 0.487559943 | 0.043 | 1.366532 |
| TRPM7 | 0.826937734 | 1.178629567 | 0.2551 | 0.59329 |
| AKT1 | 0.82715158 | 2.036302988 | 0.668 | 0.175224 |
| SLK | 0.827233856 | 0.620237227 | 0.0759 | 1.119758 |
| DAPK2 | 0.833976892 | 0.65353236 | 0.0838 | 1.076756 |
| AURKA | 0.840439229 | 0.165001174 | 0.002 | 2.69897 |
| XYLB | 0.847692784 | 0.714317044 | 0.0335 | 1.474955 |
| DGUOK | 0.851073284 | 1.266376059 | 0.2715 | 0.56623 |
| GUCY2F | 0.854674151 | 0.155942854 | 0.0109 | 1.962574 |
| GLP2R | 0.855496107 | 0.28910361 | 0.036 | 1.443697 |
| IGF2R | 0.85903578 | 1.000963588 | 0.1846 | 0.733768 |
| ABL2 | 0.862210072 | 0.436883707 | 0.0116 | 1.935542 |
| CASK | 0.863619929 | 1.797803139 | 0.6201 | 0.207538 |
| ADCK1 | 0.86696377 | 0.678142802 | 0.0834 | 1.078834 |
| NRK | 0.871433089 | 0.848211108 | 0.1322 | 0.878769 |
| STK35 | 0.873160645 | 0.610729859 | 0.0646 | 1.189767 |
| MAPK13 | 0.877639856 | 0.543706121 | 0.0483 | 1.316053 |
| PFKFB4 | 0.879301729 | 0.76372506 | 0.1047 | 0.980053 |
| PAK6 | 0.888630717 | 0.633682636 | 0.0676 | 1.170053 |
| PYCS | 0.890379712 | 0.782956181 | 0.1075 | 0.968592 |
| ERN1 | 0.890517057 | 0.856968237 | 0.1292 | 0.888737 |
| PRKCM | 0.899834109 | 0.591283027 | 0.0557 | 1.254145 |
| CDKN1A | 0.910249073 | 0.277442662 | 0.0072 | 2.142668 |
| PIP5K2C | 0.911627327 | 0.345119148 | 0.0132 | 1.879426 |
| CLK2 | 0.914974702 | 1.002076022 | 0.1653 | 0.781727 |
| PRKCZ | 0.923564953 | 1.217368905 | 0.2265 | 0.644932 |
| NTSR2 | 0.92876247 | 0.615531067 | 0.1205 | 0.919013 |
| ARAF1 | 0.930009072 | 0.629214552 | 0.0597 | 1.224026 |
| CAMKK1 | 0.933644947 | 1.090916128 | 0.1855 | 0.731656 |
| OPN1SW | 0.93518076 | 1.179672 | 0.3034 | 0.517984 |
| PIM1 | 0.940333324 | 0.606152466 | 0.0532 | 1.274088 |
| MAP3K5 | 0.950610971 | 1.335836504 | 0.343 | 0.464706 |
| PIK4CA | 0.952029199 | 1.156226058 | 0.2899 | 0.537752 |
| AK7 | 0.954356552 | 0.790172819 | 0.0316 | 1.500313 |
| KCNH2 | 0.956843941 | 0.325833443 | 0.0098 | 2.008774 |
| FRAP1 | 0.964477355 | 0.388127018 | 0.1765 | 0.753255 |
| ANKRD3 | 0.966621851 | 1.003260604 | 0.1496 | 0.825068 |
| UCK1 | 0.969278454 | 1.147156231 | 0.1896 | 0.722162 |
| STK17A | 0.991123615 | 0.673808989 | 0.1257 | 0.900665 |
| CCRK | 0.992294659 | 0.844626544 | 0.1788 | 0.747632 |
| MVK | 0.99772072 | 1.21066668 | 0.1979 | 0.703554 |
| STK22B | 1.000940695 | 0.881313133 | 0.1078 | 0.967381 |
| NTRK2 | 1.00203193 | 0.332155707 | 0.1466 | 0.833866 |
| AXL | 1.003616219 | 0.760293583 | 0.0777 | 1.109579 |
| CAMKIINALPHA | 1.008507306 | 0.714387413 | 0.0097 | 2.013228 |
| GPR109B | 1.012019082 | 2.312926666 | 0.5277 | 0.277613 |
| ADRA2A | 1.013406358 | 1.883569463 | 0.4498 | 0.346981 |
| MAP3K15 | 1.014257478 | 0.785413055 | 0.0816 | 1.08831 |
| DCAMKL1 | 1.014911413 | 0.799354565 | 0.0847 | 1.072117 |
| PRKWNK1 | 1.025974274 | 1.440453334 | 0.2495 | 0.602929 |
| PRKX | 1.027958201 | 0.67128356 | 0.0549 | 1.260428 |
| F2RL3 | 1.03451779 | 0.251366566 | 0.0191 | 1.718967 |
| CDK5R2 | 1.036055255 | 0.872390496 | 0.098 | 1.008774 |
| PRKAA1 | 1.045339006 | 0.10914145 | 0.0036 | 2.443697 |
| CKMT1B | 1.047033904 | 1.470095113 | 0.1415 | 0.849244 |
| FY | 1.050252754 | 0.693388862 | 0.1197 | 0.921906 |
| KDR | 1.052374416 | 1.134947495 | 0.2495 | 0.602929 |
| ERBB3 | 1.05492021 | 0.495631063 | 0.0238 | 1.623423 |
| CAMK1 | 1.056395752 | 0.894661762 | 0.0993 | 1.003051 |
| MAP3K7 | 1.061235035 | 0.023415809 | 0.0099 | 2.004365 |
| TRIB2 | 1.064440074 | 0.723047489 | 0.0603 | 1.219683 |
| PRKDC | 1.065720812 | 0.697676619 | 0.0552 | 1.258061 |
| CCR4 | 1.072187101 | 0.615231504 | 0.0945 | 1.024568 |
| CSNK2A1 | 1.072786189 | 1.082383351 | 0.1417 | 0.84863 |
| PRKAR1A | 1.073691265 | 0.60849391 | 0.0387 | 1.412289 |
| IRAK1 | 1.075329044 | 0.24896216 | 0.0174 | 1.759451 |
| DKFZP434C131 | 1.077263554 | 0.841102576 | 0.0831 | 1.080399 |
| JAK3 | 1.077828393 | 0.381101876 | 0.0109 | 1.962574 |
| UHMK1 | 1.091842549 | 0.411742541 | 0.0131 | 1.882729 |
| DGKH | 1.100781243 | 0.784893896 | 0.0676 | 1.170053 |
| GRK5 | 1.102228978 | 0.54284338 | 0.0722 | 1.141463 |
| MTMR4 | 1.110562011 | 0.413347516 | 0.0126 | 1.899629 |
| PRKCN | 1.122161884 | 0.64055939 | 0.2442 | 0.612254 |
| PRKWNK3 | 1.128743868 | 1.069311642 | 0.1252 | 0.902396 |
| MAP3K1 | 1.137147145 | 0.60475611 | 0.0329 | 1.482804 |
| TTBK1 | 1.138668616 | 0.976724923 | 0.1809 | 0.742561 |
| PKLR | 1.139303108 | 1.38261512 | 0.1979 | 0.703554 |
| EPHA6 | 1.14411241 | 0.316621024 | 0.0055 | 2.259637 |
| MAP3K3 | 1.146041859 | 0.469434583 | 0.0164 | 1.785156 |
| MIDORI | 1.150324059 | 0.329736766 | 0.0263 | 1.580044 |
| CDC25A | 1.167987927 | 0.971689655 | 0.0955 | 1.019997 |
| MGC45428 | 1.168736212 | 1.353932376 | 0.1827 | 0.738261 |
| PCTK2 | 1.180663661 | 0.288127103 | 0.0038 | 2.420216 |
| PTPDC1 | 1.185305086 | 0.725539205 | 0.0469 | 1.328827 |
| RPS6KA6 | 1.19009833 | 1.24665528 | 0.1522 | 0.817585 |
| HTR3A | 1.193664767 | 0.850926925 | 0.1357 | 0.86742 |
| NTRK3 | 1.223128661 | 0.804594276 | 0.0559 | 1.252588 |
| MAP3K8 | 1.226253286 | 0.685460634 | 0.0373 | 1.428291 |
| KSR2 | 1.230029393 | 0.893053515 | 0.0705 | 1.151811 |
| CRKL | 1.231673482 | 0.616929759 | 0.0281 | 1.551294 |
| STK10 | 1.240578855 | 0.626673584 | 0.0288 | 1.540608 |
| FGFRL1 | 1.245319007 | 1.457411951 | 0.186 | 0.730487 |
| MARK4 | 1.259499749 | 1.957414818 | 0.2884 | 0.540005 |
| SMO | 1.261715612 | 0.152154375 | 0.0542 | 1.266001 |
| TNK2 | 1.264330985 | 0.943414074 | 0.075 | 1.124939 |
| AK3 | 1.276101923 | 0.658819266 | 0.0304 | 1.517126 |
| TEX14 | 1.282156776 | 1.70559009 | 0.3227 | 0.491201 |
| ROR2 | 1.287017218 | 0.677748661 | 0.032 | 1.49485 |
| ACVR1 | 1.300129666 | 0.535888626 | 0.0167 | 1.777284 |
| STK16 | 1.326231626 | 0.951568017 | 0.1372 | 0.862646 |
| MAP2K7 | 1.33689074 | 1.044491061 | 0.0832 | 1.079877 |
| COL4A3BP | 1.345803793 | 0.734546665 | 0.0866 | 1.062482 |
| PRKCB1 | 1.355939387 | 1.451695246 | 0.1586 | 0.799697 |
| PKIB | 1.364422818 | 0.8346722 | 0.0468 | 1.329754 |
| CLK1 | 1.364556261 | 1.06246023 | 0.1561 | 0.806597 |
| CDC42BPB | 1.382948862 | 1.372411138 | 0.1373 | 0.862329 |
| CMKLR1 | 1.390452207 | 0.782231743 | 0.0913 | 1.039529 |
| RIOK3 | 1.393539924 | 0.371986329 | 0.0049 | 2.309804 |
| PDXK | 1.397986011 | 1.180545105 | 0.0986 | 1.006123 |
| PTK6 | 1.417812829 | 1.063095328 | 0.0759 | 1.119758 |
| ARK5 | 1.434413097 | 0.976027155 | 0.0605 | 1.218245 |
| BCR | 1.439040093 | 1.144752267 | 0.0866 | 1.062482 |
| FLT4 | 1.444941319 | 1.049360896 | 0.0705 | 1.151811 |
| AIP1 | 1.450163231 | 0.394481895 | 0.0052 | 2.283997 |
| CDKN2D | 1.467961858 | 1.289448346 | 0.1072 | 0.969805 |
| NEK3 | 1.477908367 | 1.237601931 | 0.3403 | 0.468138 |
| CDC2L1 | 1.482290962 | 1.468667597 | 0.1368 | 0.863914 |
| AATK | 1.494354247 | 1.269085586 | 0.0999 | 1.000435 |
| PIK3C2G | 1.525085131 | 0.590187956 | 0.0141 | 1.850781 |
| STK22D | 1.528706376 | 1.188915681 | 0.0824 | 1.084073 |
| ALS2CR2 | 1.532070555 | 1.095990537 | 0.1365 | 0.864867 |
| MARK1 | 1.534859651 | 1.27452623 | 0.0951 | 1.021819 |
| ADRA2C | 1.564113694 | 0.968132938 | 0.0482 | 1.316953 |
| PRKCQ | 1.577176258 | 0.382041723 | 0.0037 | 2.431798 |
| NEK8 | 1.581693409 | 0.85624801 | 0.0344 | 1.463442 |
| MAP4K1 | 1.602189525 | 0.490854747 | 0.0073 | 2.136677 |
| CAMK1G | 1.653880084 | 0.725577627 | 0.0198 | 1.703335 |
| EPHA3 | 1.663292425 | 1.463676402 | 0.1077 | 0.967784 |
| GRK4 | 1.681877369 | 0.410164829 | 0.0038 | 2.420216 |
| KCNH8 | 1.682880782 | 0.517062808 | 0.0301 | 1.521434 |
| FGFR2 | 1.685719623 | 0.734685427 | 0.0194 | 1.712198 |
| EIF2AK4 | 1.688060182 | 0.506010633 | 0.0287 | 1.542118 |
| CSNK2B | 1.697682214 | 0.865910927 | 0.0295 | 1.530178 |
| GRK7 | 1.713081152 | 0.863966367 | 0.0286 | 1.543634 |
| RIPK1 | 1.740152064 | 0.915381459 | 0.032 | 1.49485 |
| PPP1R1B | 1.751777184 | 0.446460741 | 0.0043 | 2.366532 |
| UMPK | 1.752200042 | 0.184659307 | 0.0037 | 2.431798 |
| HTR1A | 1.792812076 | 2.076772785 | 0.1827 | 0.738261 |
| LMTK3 | 1.817987765 | 0.97167384 | 0.0333 | 1.477556 |
| PFKL | 1.821863901 | 0.644084648 | 0.0109 | 1.962574 |
| SSTR5 | 1.841978669 | 0.451672764 | 0.0195 | 1.709965 |
| PRKCL2 | 1.883380864 | 1.684429346 | 0.1114 | 0.953115 |
| SRC | 1.912274332 | 0.137441984 | 0.0323 | 1.490797 |
| STK39 | 1.935633753 | 1.479206921 | 0.0792 | 1.101275 |
| PPP1R3A | 1.943957702 | 0.838452155 | 0.0189 | 1.723538 |
| HUNK | 1.983025049 | 1.197809767 | 0.0454 | 1.342944 |
| CDK8 | 1.986730363 | 0.129513277 |  |  |
| STK11 | 2.159867807 | 0.345793243 | 0.0011 | 2.958607 |
| G6PC3 | 2.163657664 | 1.838103031 | 0.0999 | 1.000435 |
| RELA | 2.207408969 | 0.946391554 | 0.0186 | 1.730487 |
| CSNK1G1 | 2.237090389 | 0.528985992 | 0.0181 | 1.742321 |
| ACVR1C | 2.375574949 | 1.871576571 | 0.0848 | 1.071604 |
| DGKA | 2.391031271 | 2.412319123 | 0.1417 | 0.84863 |
| MINK | 2.767894316 | 0.387899313 | 0.0629 | 1.201349 |
| CSF1R | 3.198353963 | 1.399490566 | 0.0196 | 1.707744 |
