# Supplementary material for: CDK1-mediated phosphorylation at H2B serine 6 is required for mitotic chromosome segregation
Source: J Cell Biol. 2019 Feb 14;218(4):1164–81. doi: 10.1083/jcb.201806057 (PMC6446833; doi:10.1083/jcb.201806057)
Supplement: Tables S1-S3 (ZIP) [file JCB_201806057_TablesS1-S3.zip › JCB_201806057_TableS2.doc]

| **LFQ intensity neg_Ctrl** | **LFQ intensity unmod_pep** | **LFQ intensity phos** | **LFQ intensity meth-phos** | **Peptides** | **Razor + unique peptides** | **Unique peptides** | **Sequence coverage [%]** | **Unique + razor sequence coverage [%]** | **Unique sequence coverage [%]** | **Mol. weight [kDa]** | **Score** | **Intensity** | **MS/ MS Count** | **Protein names** | **Gene names** |
| --- | --- | --- | --- | --- | --- | --- | --- | --- | --- | --- | --- | --- | --- | --- | --- |
| 33.87014 | 35.15689 | 35.26458 | 34.98388 | 182 | 182 | 1 | 79.9 | 79.9 | 0.8 | 265.55 | 323.31 | 1.4129E+12 | 3109 | Acetyl-CoA carboxylase 1;Biotin carboxylase | ACACA |
| 34.39567 | 34.47066 | 34.88921 | 34.66792 | 85 | 85 | 85 | 79.8 | 79.8 | 79.8 | 129.63 | 323.31 | 1.0661E+12 | 1549 | Pyruvate carboxylase, mitochondrial | PC |
| 35.62206 | 32.81387 | 33.83686 | 33.00149 | 37 | 37 | 0 | 91.5 | 91.5 | 0 | 41.792 | 323.31 | 1.1324E+12 | 1504 | Actin, cytoplasmic 2;Actin, cytoplasmic 2, N-terminally processed | ACTG1 |
| 23.48922 | 24.92976 | 32.70366 | 30.05155 | 24 | 21 | 20 | 77 | 69.8 | 66.9 | 27.774 | 323.31 | 3.5399E+10 | 269 | 14-3-3 protein sigma | SFN |
| 26.98522 | 27.4219 | 32.41304 | 29.88807 | 26 | 26 | 23 | 77.6 | 77.6 | 70.6 | 29.174 | 323.31 | 3.0026E+10 | 215 | 14-3-3 protein epsilon | YWHAE |
| 32.13687 | 30.49734 | 31.28675 | 30.82646 | 194 | 194 | 183 | 71.5 | 71.5 | 68.4 | 226.53 | 323.31 | 3.6033E+11 | 1200 | Myosin-9 | MYH9 |
| 29.82733 | 31.04225 | 30.95518 | 30.79071 | 54 | 54 | 54 | 69 | 69 | 69 | 73.62 | 323.31 | 6.3331E+10 | 693 | Heterogeneous nuclear ribonucleoprotein M | HNRNPM |
| 29.82498 | 30.59272 | 30.92309 | 31.12995 | 48 | 48 | 27 | 84.1 | 84.1 | 50.8 | 61.054 | 323.31 | 4.6409E+10 | 353 | 60 kDa heat shock protein, mitochondrial | HSPD1 |
| 29.45093 | 30.82866 | 30.79668 | 30.98638 | 89 | 89 | 86 | 63.6 | 63.6 | 62.1 | 165.93 | 323.31 | 2.8652E+10 | 385 | Carbamoyl-phosphate synthase [ammonia], mitochondrial | CPS1 |
| 25.9061 | 25.64645 | 30.69544 | 30.52673 | 18 | 14 | 12 | 62.3 | 52.2 | 45.7 | 28.302 | 186.76 | 1.0352E+10 | 153 | 14-3-3 protein gamma;14-3-3 protein gamma, N-terminally processed | YWHAG |
| 27.42254 | 27.14209 | 30.69544 | 30.2589 | 22 | 18 | 17 | 79.2 | 69 | 65.3 | 27.745 | 323.31 | 1.1275E+10 | 160 | 14-3-3 protein zeta/delta | YWHAZ |
| 31.16549 | 30.81406 | 30.67901 | 28.62041 | 88 | 88 | 68 | 56.7 | 56.7 | 45.8 | 187.89 | 323.31 | 5.7568E+10 | 362 | Clathrin heavy chain 1 | CLTC |
| 30.7332 | 30.51277 | 30.47875 | 30.80724 | 24 | 24 | 13 | 60 | 60 | 35.1 | 50.184 | 323.31 | 6.0691E+10 | 236 | Putative elongation factor 1-alpha-like 3;Elongation factor 1-alpha 1 | EEF1A1P5;EEF1A1 |
| 30.23603 | 30.56174 | 30.43966 | 30.6801 | 48 | 48 | 22 | 61.3 | 61.3 | 35.2 | 83.263 | 323.31 | 6.895E+10 | 303 | Heat shock protein HSP 90-beta | HSP90AB1 |
| 30.64882 | 30.19613 | 30.32313 | 30.4826 | 26 | 26 | 0 | 65.6 | 65.6 | 0 | 50.151 | 323.31 | 4.4973E+10 | 250 | Tubulin alpha-1B chain | TUBA1B |
| 30.01969 | 30.11371 | 30.19155 | 30.43549 | 25 | 25 | 3 | 55.1 | 55.1 | 7 | 49.83 | 323.31 | 4.5155E+10 | 264 | Tubulin beta-4B chain;Tubulin beta-4A chain | TUBB4B;TUBB4A |
| 28.37302 | 30.09723 | 30.17712 | 30.17057 | 17 | 17 | 17 | 54.3 | 54.3 | 54.3 | 36.053 | 323.31 | 1.8643E+10 | 165 | Glyceraldehyde-3-phosphate dehydrogenase | GAPDH |
| 30.87811 | 30.79289 | 30.06638 | 30.9789 | 39 | 36 | 23 | 62.1 | 61 | 45.4 | 70.897 | 323.31 | 5.7533E+10 | 404 | Heat shock cognate 71 kDa protein | HSPA8 |
| 33.78858 | 30.74262 | 29.96733 | 30.39632 | 171 | 171 | 18 | 73.8 | 73.8 | 6.4 | 280.01 | 323.31 | 7.3607E+11 | 1045 | Filamin-A | FLNA |
| 28.87646 | 30.40862 | 29.8649 | 30.81306 | 33 | 33 | 30 | 70.3 | 70.3 | 61.8 | 47.168 | 323.31 | 2.7788E+10 | 202 | Alpha-enolase | ENO1 |
| 26.79625 | 28.48631 | 29.61942 | 29.09194 | 9 | 9 | 9 | 49.4 | 49.4 | 49.4 | 19.25 | 127.95 | 9128900000 | 173 | Lipocalin-1 | LCN1 |
| 29.48656 | 29.65605 | 29.56828 | 29.32937 | 48 | 48 | 47 | 61.1 | 61.1 | 61.1 | 72.4 | 323.31 | 2.4395E+10 | 308 | Stress-70 protein, mitochondrial | HSPA9 |
| 26.84518 | 28.44896 | 29.55768 | 28.48919 | 6 | 6 | 6 | 42.7 | 42.7 | 42.7 | 11.284 | 49.592 | 4955100000 | 91 | Dermcidin;Survival-promoting peptide;DCD-1 | DCD |
| 27.7513 | 30.82442 | 29.50099 | 28.25061 | 15 | 15 | 15 | 58 | 58 | 58 | 34.273 | 230.87 | 1.3497E+10 | 80 | 60S acidic ribosomal protein P0;60S acidic ribosomal protein P0-like | RPLP0;RPLP0P6 |
| 28.16265 | 29.33599 | 29.49035 | 29.18997 | 37 | 37 | 37 | 66.3 | 66.3 | 66.3 | 57.936 | 323.31 | 1.5804E+10 | 171 | Pyruvate kinase PKM;Pyruvate kinase | PKM |
| 29.39147 | 28.16399 | 29.46935 | 29.43526 | 23 | 5 | 4 | 53.1 | 14.1 | 10.6 | 47.766 | 155.08 | 1.9757E+10 | 51 | Tubulin beta chain | TUBB |
| 24.08699 | 23.51424 | 29.45433 | 29.36072 | 18 | 14 | 14 | 67.9 | 58.1 | 58.1 | 28.218 | 180.8 | 3721200000 | 82 | 14-3-3 protein eta | YWHAH |
| 28.12302 | 28.89663 | 29.35785 | 28.41422 | 50 | 50 | 50 | 54.2 | 54.2 | 54.2 | 107.97 | 323.31 | 1.2004E+10 | 152 | General transcription factor II-I | GTF2I |
| 28.3173 | 29.91056 | 29.3554 | 28.97828 | 14 | 14 | 14 | 53.2 | 53.2 | 53.2 | 29.464 | 323.31 | 1.4393E+10 | 147 | Nucleophosmin | NPM1 |
| 29.4582 | 28.15316 | 29.3421 | 27.56427 | 56 | 56 | 52 | 54.3 | 54.3 | 51.5 | 124.95 | 323.31 | 2.7104E+10 | 238 | Unconventional myosin-Ib | MYO1B |
| 30.48587 | 29.17074 | 29.32631 | 28.8938 | 32 | 32 | 32 | 70.5 | 70.5 | 70.5 | 59.403 | 323.31 | 3.7094E+10 | 159 | Propionyl-CoA carboxylase beta chain, mitochondrial | PCCB |
| 28.27244 | 31.18287 | 29.28058 | 28.87423 | 43 | 43 | 43 | 41.5 | 41.5 | 41.5 | 76.613 | 323.31 | 4.1121E+10 | 217 | Nucleolin | NCL |
| 27.75385 | 29.13428 | 29.21099 | 29.23302 | 16 | 16 | 16 | 28.4 | 28.4 | 28.4 | 58.656 | 204.33 | 4315500000 | 92 | RNA-binding protein 39 | RBM39;RNPC2 |
| 27.28595 | 28.79424 | 29.17743 | 29.35208 | 30 | 30 | 26 | 65.1 | 65.1 | 60.7 | 39.42 | 323.31 | 8740200000 | 114 | Fructose-bisphosphate aldolase A | ALDOA |
| 28.98395 | 29.82043 | 29.08803 | 29.24091 | 40 | 40 | 38 | 55.5 | 55.5 | 54.4 | 72.332 | 323.31 | 2.6698E+10 | 310 | 78 kDa glucose-regulated protein | HSPA5 |
| 28.68164 | 28.98661 | 28.96428 | 29.04573 | 46 | 28 | 27 | 56 | 37.8 | 36.9 | 84.659 | 323.31 | 2.2169E+10 | 135 | Heat shock protein HSP 90-alpha | HSP90AA1 |
| 27.68833 | 28.75821 | 28.9569 | 29.274 | 46 | 46 | 45 | 57.8 | 57.8 | 56.6 | 95.337 | 323.31 | 1.8245E+10 | 198 | Elongation factor 2 | EEF2 |
| 26.81714 | 27.64203 | 28.79197 | 28.14754 | 25 | 25 | 25 | 67.1 | 67.1 | 67.1 | 44.614 | 323.31 | 5458000000 | 120 | Phosphoglycerate kinase 1;Phosphoglycerate kinase | PGK1 |
| 29.83895 | 29.36123 | 28.72618 | 28.79999 | 44 | 44 | 44 | 64.8 | 64.8 | 64.8 | 80.058 | 323.31 | 2.6399E+10 | 304 | Propionyl-CoA carboxylase alpha chain, mitochondrial | PCCA |
| 28.74336 | 27.31915 | 28.57715 | 27.97645 | 52 | 52 | 2 | 47.8 | 47.8 | 1.9 | 119.52 | 323.31 | 1.8504E+10 | 182 | Unconventional myosin-Ic | MYO1C |
| 28.93955 | 28.38653 | 28.46918 | 28.79659 | 10 | 10 | 7 | 49.2 | 49.2 | 36.2 | 22.11 | 70.412 | 2612300000 | 95 | Peroxiredoxin-1 | PRDX1 |
| 26.83641 | 28.64048 | 28.45772 | 28.29013 | 100 | 78 | 76 | 44.3 | 36.8 | 36 | 276.54 | 323.31 | 1.7524E+10 | 278 | Acetyl-CoA carboxylase 2;Biotin carboxylase | ACACB |
| 27.42614 | 28.95424 | 28.37675 | 27.99108 | 6 | 6 | 6 | 42.2 | 42.2 | 42.2 | 14.787 | 76.968 | 2019000000 | 20 | 60S ribosomal protein L22 | RPL22 |
| 29.45321 | 28.17798 | 28.35288 | 28.0239 | 8 | 8 | 8 | 51.9 | 51.9 | 51.9 | 17.965 | 109.83 | 2.0499E+10 | 146 | Ubiquitin-40S ribosomal protein S27a;Ubiquitin;40S ribosomal protein S27a;Ubiquitin-60S ribosomal protein L40;Ubiquitin;60S ribosomal protein L40;Polyubiquitin-B;Ubiquitin;Polyubiquitin-C;Ubiquitin | RPS27A;UBC;UBB;UBA52 |
| 30.68361 | 28.06856 | 28.30921 | 27.9391 | 181 | 181 | 4 | 73.7 | 73.7 | 1.8 | 284.54 | 323.31 | 1.1747E+11 | 560 | Spectrin alpha chain, non-erythrocytic 1 | SPTAN1 |
| 25.05014 | 31.01664 | 28.29281 | 26.59705 | 9 | 9 | 1 | 36.6 | 36.6 | 7.2 | 33.488 | 256.46 | 9445300000 | 41 | Protein SET | SET |
| 27.26329 | 27.94201 | 28.24794 | 28.34168 | 71 | 71 | 71 | 24.6 | 24.6 | 24.6 | 331.77 | 323.31 | 4096300000 | 372 | Desmoplakin | DSP |
| 29.91014 | 26.6345 | 28.23562 | 26.28152 | 23 | 4 | 0 | 52.5 | 19.6 | 0 | 42.019 | 141.92 | 4.1584E+10 | 74 | Actin, alpha cardiac muscle 1;Actin, gamma-enteric smooth muscle;Actin, alpha skeletal muscle;Actin, aortic smooth muscle | ACTC1;ACTG2;ACTA1;ACTA2 |
| 28.7742 | 31.55193 | 28.18976 | 26.8447 | 13 | 13 | 13 | 43.3 | 43.3 | 43.3 | 23.577 | 89.688 | 5696600000 | 58 | 60S ribosomal protein L13a | RPL13A |
| 26.53069 | 27.66119 | 28.17007 | 27.87215 | 13 | 13 | 13 | 42 | 42 | 42 | 42.644 | 315.46 | 3592800000 | 56 | Creatine kinase B-type | CKB |
| 26.4548 | 28.1985 | 28.16289 | 28.67728 | 87 | 87 | 87 | 40.2 | 40.2 | 40.2 | 273.42 | 323.31 | 1.2785E+10 | 269 | Fatty acid synthase;[Acyl-carrier-protein] S-acetyltransferase;[Acyl-carrier-protein] S-malonyltransferase;3-oxoacyl-[acyl-carrier-protein] synthase;3-oxoacyl-[acyl-carrier-protein] reductase;3-hydroxyacyl-[acyl-carrier-protein] dehydratase;Enoyl-[acyl-carrier-protein] reductase;Oleoyl-[acyl-carrier-protein] hydrolase | FASN |
| 28.73467 | 29.73589 | 28.13624 | 27.19929 | 7 | 7 | 7 | 41.6 | 41.6 | 41.6 | 18.11 | 85.134 | 2488100000 | 54 | 60S ribosomal protein L18a | RPL18A |
| 27.92824 | 28.07704 | 28.09607 | 28.59676 | 34 | 28 | 16 | 52.4 | 48.3 | 27.3 | 77.405 | 323.31 | 9983400000 | 129 | Heat shock 70 kDa protein 1A/1B | HSPA1A |
| 27.4753 | 27.49221 | 28.07373 | 26.22869 | 22 | 22 | 21 | 61.4 | 61.4 | 57.8 | 36.688 | 323.31 | 4848800000 | 85 | L-lactate dehydrogenase A chain | LDHA |
| 28.5339 | 29.34742 | 28.07025 | 28.03491 | 38 | 38 | 38 | 38.6 | 38.6 | 38.6 | 88.979 | 323.31 | 2.2734E+10 | 169 | Heterogeneous nuclear ribonucleoprotein U | HNRNPU |
| 26.97027 | 25.47695 | 28.05315 | 26.94257 | 10 | 10 | 10 | 40.3 | 40.3 | 40.3 | 23.742 | 66.993 | 800190000 | 38 | Peptidyl-prolyl cis-trans isomerase B | PPIB |
| 26.7893 | 29.03724 | 28.04375 | 24.50331 | 14 | 14 | 14 | 44.4 | 44.4 | 44.4 | 28.024 | 252.41 | 4072200000 | 84 | 60S ribosomal protein L8 | RPL8 |
| 29.25636 | 27.02136 | 28.01988 | 27.99556 | 10 | 10 | 8 | 63.3 | 63.3 | 52.4 | 18.502 | 123.63 | 2460200000 | 51 | Cofilin-1 | CFL1 |
| 24.63134 | 23.86618 | 28.01728 | 28.73159 | 15 | 9 | 2 | 56.9 | 39.4 | 4.5 | 28.082 | 256.52 | 1884600000 | 42 | 14-3-3 protein beta/alpha;14-3-3 protein beta/alpha, N-terminally processed | YWHAB |
| 26.78169 | 28.18546 | 28.01319 | 28.51473 | 21 | 21 | 21 | 54.6 | 54.6 | 54.6 | 56.559 | 312.36 | 7169300000 | 117 | ATP synthase subunit beta, mitochondrial;ATP synthase subunit beta | ATP5B |
| 30.42982 | 26.57083 | 28.0127 | 27.22481 | 9 | 9 | 7 | 62.8 | 62.8 | 46.2 | 16.29 | 110.16 | 5514400000 | 49 | Myosin light polypeptide 6 | PDE6H;MYL6 |
| 26.35424 | 27.45842 | 27.99776 | 28.18688 | 17 | 17 | 17 | 49.6 | 49.6 | 49.6 | 48.141 | 323.31 | 4953500000 | 70 | Calreticulin | CALR |
| 27.98055 | 28.15822 | 27.99674 | 28.65513 | 23 | 23 | 23 | 51 | 51 | 51 | 46.53 | 317.27 | 8572700000 | 125 | Argininosuccinate synthase | ASS1 |
| 29.98631 | 27.23736 | 27.98191 | 27.30295 | 151 | 151 | 10 | 65.9 | 65.9 | 6.4 | 274.61 | 323.31 | 8.9757E+10 | 448 | Spectrin beta chain, non-erythrocytic 1 | SPTBN1 |
| 29.40912 | 27.9651 | 27.96312 | 28.68398 | 49 | 49 | 47 | 61.7 | 61.7 | 61.6 | 74.139 | 323.31 | 1.9667E+10 | 276 | Prelamin-A/C;Lamin-A/C | LMNA |
| 26.84338 | 28.22265 | 27.95255 | 28.25701 | 43 | 41 | 41 | 48.2 | 46.5 | 46.5 | 92.468 | 323.31 | 1.0201E+10 | 111 | Endoplasmin | HSP90B1 |
| 29.21357 | 30.16651 | 27.95088 | 28.03496 | 11 | 11 | 2 | 51.7 | 51.7 | 8.4 | 20.252 | 92.058 | 3535900000 | 78 | 60S ribosomal protein L11 | RPL11 |
| 29.10112 | 27.76009 | 27.91547 | 28.26441 | 19 | 19 | 19 | 64 | 64 | 64 | 37.429 | 323.31 | 1.3724E+10 | 87 | Heterogeneous nuclear ribonucleoproteins A2/B1 | HNRNPA2B1 |
| 26.62993 | 27.19629 | 27.90971 | 26.72559 | 47 | 47 | 47 | 41.4 | 41.4 | 41.4 | 144.5 | 323.31 | 3436900000 | 106 | Isoleucine--tRNA ligase, cytoplasmic | IARS |
| 26.68606 | 27.47792 | 27.89828 | 28.41159 | 31 | 31 | 31 | 37.3 | 37.3 | 37.3 | 117.85 | 323.31 | 8312900000 | 103 | Ubiquitin-like modifier-activating enzyme 1 | UBA1 |
| 27.70088 | 27.79922 | 27.88995 | 28.28282 | 28 | 28 | 28 | 61.3 | 61.3 | 61.3 | 57.488 | 323.31 | 7926200000 | 121 | T-complex protein 1 subunit beta | CCT2 |
| 27.01531 | 27.59676 | 27.88826 | 27.66838 | 11 | 11 | 11 | 50.4 | 50.4 | 50.4 | 29.505 | 278.06 | 5315500000 | 48 | 40S ribosomal protein SA | RPSA;RPSAP58 |
| 27.57376 | 28.09999 | 27.8685 | 28.38084 | 20 | 20 | 20 | 34.3 | 34.3 | 34.3 | 50.118 | 294.77 | 7139900000 | 103 | Elongation factor 1-gamma | EEF1G |
| 28.28154 | 28.02944 | 27.86844 | 27.58284 | 24 | 24 | 24 | 57.5 | 57.5 | 57.5 | 48.562 | 323.31 | 9250000000 | 119 | Heterogeneous nuclear ribonucleoprotein K | HNRNPK |
| 26.70596 | 30.29406 | 27.86437 | 27.18281 | 26 | 26 | 26 | 52.7 | 52.7 | 52.7 | 45.498 | 292.6 | 1.0599E+10 | 197 | 60S ribosomal protein L4 | RPL4 |
| 27.98495 | 29.44237 | 27.84751 | 24.79927 | 19 | 19 | 19 | 55.6 | 55.6 | 55.6 | 29.225 | 237.06 | 7131200000 | 78 | 60S ribosomal protein L7 | RPL7 |
| 27.36503 | 28.46147 | 27.83551 | 26.55013 | 10 | 10 | 10 | 38.2 | 38.2 | 38.2 | 24.831 | 74.958 | 1128200000 | 33 | 60S ribosomal protein L10a | RPL10A |
| 27.23444 | 28.19357 | 27.8302 | 28.99825 | 30 | 30 | 30 | 53.6 | 53.6 | 53.6 | 67.877 | 323.31 | 7652100000 | 151 | Transketolase | TKT |
| 26.67909 | 27.96345 | 27.80686 | 28.27915 | 28 | 28 | 28 | 51 | 51 | 51 | 59.75 | 323.31 | 5543100000 | 102 | ATP synthase subunit alpha, mitochondrial | ATP5A1 |
| 27.50256 | 28.00475 | 27.79928 | 27.74323 | 29 | 29 | 29 | 62.7 | 62.7 | 62.7 | 57.924 | 323.31 | 7133500000 | 126 | T-complex protein 1 subunit delta | CCT4 |
| 28.0879 | 29.25818 | 27.75462 | 26.40938 | 12 | 12 | 12 | 49 | 49 | 49 | 22.591 | 75.462 | 1996600000 | 58 | 40S ribosomal protein S9 | RPS9 |
| 27.106 | 27.7267 | 27.75411 | 27.70813 | 29 | 29 | 29 | 53.5 | 53.5 | 53.5 | 57.116 | 323.31 | 5620000000 | 106 | Protein disulfide-isomerase | P4HB |
| 27.49724 | 28.08654 | 27.72956 | 25.47076 | 10 | 10 | 10 | 42.3 | 42.3 | 42.3 | 17.695 | 70.948 | 1023200000 | 41 | 60S ribosomal protein L23a | RPL23A |
| 25.27547 | 27.20658 | 27.72891 | 27.5957 | 8 | 8 | 8 | 62.2 | 62.2 | 62.2 | 16.537 | 119.88 | 2929100000 | 120 | Lysozyme C | LYZ |
| 26.65281 | 26.76243 | 27.72156 | 27.16471 | 28 | 28 | 28 | 56.3 | 56.3 | 56.3 | 60.533 | 292.43 | 4524600000 | 108 | T-complex protein 1 subunit gamma | CCT3 |
| 27.16835 | 27.57029 | 27.70048 | 28.37563 | 64 | 64 | 63 | 47.9 | 47.9 | 47.9 | 170.59 | 323.31 | 5570600000 | 169 | Bifunctional glutamate/proline--tRNA ligase;Glutamate--tRNA ligase;Proline--tRNA ligase | EPRS |
| 27.42085 | 26.98815 | 27.67882 | 27.69012 | 12 | 12 | 2 | 41.7 | 41.7 | 7.2 | 38.221 | 175.04 | 5037900000 | 57 | Poly(rC)-binding protein 2;Poly(rC)-binding protein 3 | PCBP2;PCBP3 |
| 25.55482 | 27.50469 | 27.67357 | 27.2562 | 25 | 25 | 25 | 30.6 | 30.6 | 30.6 | 84.051 | 299.27 | 3971100000 | 125 | Ensconsin | MAP7 |
| 30.66119 | 26.18669 | 27.66465 | 26.88658 | 29 | 29 | 29 | 66.4 | 66.4 | 66.4 | 38.604 | 323.31 | 3.5335E+10 | 154 | Annexin A2;Annexin;Putative annexin A2-like protein | ANXA2;ANXA2P2 |
| 30.18187 | 27.14054 | 27.65785 | 26.11058 | 86 | 86 | 16 | 60.2 | 60.2 | 11.6 | 153.67 | 323.31 | 2.6888E+10 | 279 | LIM domain only protein 7 | LMO7 |
| 27.4729 | 30.5662 | 27.65717 | 26.69498 | 27 | 27 | 27 | 47.9 | 47.9 | 47.9 | 46.108 | 308.54 | 1.0637E+10 | 164 | 60S ribosomal protein L3 | RPL3 |
| 31.10005 | 27.3286 | 27.65219 | 27.84966 | 46 | 43 | 42 | 80.5 | 77 | 75.1 | 53.651 | 323.31 | 4.5679E+10 | 278 | Vimentin | VIM |
| 25.65146 | 27.16145 | 27.62513 | 26.77329 | 29 | 29 | 27 | 54.7 | 54.7 | 51.7 | 76.625 | 323.31 | 3738400000 | 159 | Lactotransferrin;Lactoferricin-H;Kaliocin-1;Lactoferroxin-A;Lactoferroxin-B;Lactoferroxin-C | LTF |
| 26.74868 | 26.93731 | 27.62101 | 27.06013 | 25 | 25 | 25 | 46.9 | 46.9 | 46.9 | 58.024 | 323.31 | 4129100000 | 74 | T-complex protein 1 subunit zeta | CCT6A |
| 28.47104 | 26.94123 | 27.60166 | 27.35632 | 25 | 24 | 16 | 56.5 | 54.9 | 41.4 | 48.057 | 323.31 | 8029000000 | 130 | Keratin, type I cytoskeletal 18 | KRT18 |
| 26.88145 | 27.46076 | 27.55735 | 27.21549 | 40 | 40 | 36 | 55.6 | 55.6 | 50 | 94.33 | 323.31 | 6959100000 | 135 | Heat shock 70 kDa protein 4 | HSPA4 |
| 26.68271 | 28.59399 | 27.55706 | 27.57051 | 12 | 12 | 12 | 49.3 | 49.3 | 49.3 | 17.718 | 93.274 | 1388900000 | 51 | 40S ribosomal protein S18 | RPS18 |
| 27.13536 | 27.7386 | 27.55128 | 27.32122 | 21 | 21 | 21 | 35.4 | 35.4 | 35.4 | 64.872 | 323.31 | 6794900000 | 86 | 4F2 cell-surface antigen heavy chain | SLC3A2 |
| 25.08756 | 26.55539 | 27.53742 | 26.09279 | 20 | 20 | 20 | 24.1 | 24.1 | 24.1 | 113.75 | 185.56 | 1447800000 | 141 | Desmoglein-1 | DSG1 |
| 27.02602 | 27.68311 | 27.53133 | 27.92954 | 120 | 120 | 120 | 31.9 | 31.9 | 31.9 | 469.08 | 323.31 | 1.5556E+10 | 326 | DNA-dependent protein kinase catalytic subunit | PRKDC |
| 19.41394 | 30.02868 | 27.52238 | 27.17997 | 49 | 49 | 49 | 47.9 | 47.9 | 47.9 | 125.5 | 323.31 | 5105800000 | 131 | Arf-GAP with SH3 domain, ANK repeat and PH domain-containing protein 1 | ASAP1 |
| 27.00174 | 27.87367 | 27.52103 | 28.00367 | 18 | 18 | 18 | 37.5 | 37.5 | 37.5 | 63.146 | 244.38 | 4984100000 | 65 | Glucose-6-phosphate isomerase | GPI |
| 28.57723 | 29.69855 | 27.5142 | 27.35481 | 11 | 11 | 11 | 50 | 50 | 50 | 21.879 | 121.32 | 2365000000 | 57 | 40S ribosomal protein S8 | RPS8 |
| 27.01871 | 27.26857 | 27.51194 | 27.32629 | 28 | 28 | 28 | 59.7 | 59.7 | 59.7 | 60.343 | 323.31 | 5041800000 | 104 | T-complex protein 1 subunit alpha | TCP1 |
| 26.9969 | 27.37302 | 27.50506 | 27.62533 | 34 | 34 | 34 | 63.1 | 63.1 | 63.1 | 59.478 | 292.86 | 5493200000 | 128 | T-complex protein 1 subunit theta | CCT8 |
| 27.03139 | 28.92824 | 27.47816 | 27.43094 | 9 | 9 | 9 | 69.1 | 69.1 | 69.1 | 17.818 | 114.07 | 1554300000 | 42 | 60S ribosomal protein L12 | RPL12 |
| 28.05941 | 25.6093 | 27.46995 | 26.01404 | 8 | 8 | 8 | 46 | 46 | 46 | 20.762 | 112.38 | 1437600000 | 44 | Calmodulin | CALM2;CALM1;CALM3 |
| 28.94193 | 28.90822 | 27.46037 | 25.78796 | 17 | 17 | 16 | 54.9 | 54.9 | 52.6 | 32.679 | 278.83 | 1.3068E+10 | 111 | Heterogeneous nuclear ribonucleoprotein A1;Heterogeneous nuclear ribonucleoprotein A1, N-terminally processed;Heterogeneous nuclear ribonucleoprotein A1-like 2 | HNRNPA1;HNRNPA1L2 |
| 27.25278 | 28.78079 | 27.44103 | 27.36761 | 13 | 13 | 13 | 57.6 | 57.6 | 57.6 | 18.431 | 92.658 | 1512900000 | 66 | 40S ribosomal protein S11 | RPS11 |
| 26.43997 | 26.84733 | 27.43976 | 27.45521 | 34 | 34 | 34 | 56.4 | 56.4 | 56.4 | 62.639 | 316.62 | 4203700000 | 102 | Stress-induced-phosphoprotein 1 | STIP1 |
| 27.20955 | 27.19582 | 27.42222 | 27.62478 | 22 | 22 | 22 | 43.7 | 43.7 | 43.7 | 56.65 | 323.31 | 5791000000 | 83 | D-3-phosphoglycerate dehydrogenase | PHGDH |
| 26.86726 | 28.03868 | 27.42013 | 28.46618 | 28 | 28 | 22 | 52.3 | 52.3 | 42.7 | 54.102 | 297.78 | 5477300000 | 87 | Protein disulfide-isomerase;Protein disulfide-isomerase A3 | PDIA3 |
| 26.71581 | 26.56597 | 27.41587 | 27.70655 | 11 | 11 | 11 | 66.1 | 66.1 | 66.1 | 18.012 | 149.41 | 1205500000 | 49 | Peptidyl-prolyl cis-trans isomerase A;Peptidyl-prolyl cis-trans isomerase A, N-terminally processed;Peptidyl-prolyl cis-trans isomerase | PPIA |
| 26.96818 | 27.15981 | 27.40194 | 27.3616 | 28 | 28 | 28 | 61.7 | 61.7 | 61.7 | 59.366 | 323.31 | 4886300000 | 108 | T-complex protein 1 subunit eta | CCT7 |
| 26.57888 | 26.30023 | 27.39713 | 26.76319 | 31 | 31 | 31 | 63.4 | 63.4 | 63.4 | 59.67 | 323.31 | 3992200000 | 102 | T-complex protein 1 subunit epsilon | CCT5 |
| 28.37642 | 28.56322 | 27.37782 | 28.41728 | 82 | 82 | 82 | 60.9 | 60.9 | 60.9 | 157.9 | 323.31 | 9943000000 | 215 | Leucine-rich PPR motif-containing protein, mitochondrial | LRPPRC |
| 26.50019 | 27.44 | 27.34908 | 27.72195 | 39 | 39 | 39 | 51.6 | 51.6 | 51.6 | 89.321 | 323.31 | 6626100000 | 126 | Transitional endoplasmic reticulum ATPase | VCP |
| 26.88809 | 28.74967 | 27.30722 | 26.05676 | 9 | 9 | 9 | 48.5 | 48.5 | 48.5 | 18.592 | 107.71 | 1159300000 | 41 | 60S ribosomal protein L10 | RPL10 |
| 26.60752 | 30.92479 | 27.28463 | 26.76812 | 50 | 50 | 50 | 66.7 | 66.7 | 66.7 | 82.999 | 323.31 | 2.9329E+10 | 136 | Trifunctional enzyme subunit alpha, mitochondrial;Long-chain enoyl-CoA hydratase;Long chain 3-hydroxyacyl-CoA dehydrogenase | HADHA |
| 29.44924 | 26.99421 | 27.27169 | 26.04195 | 76 | 76 | 1 | 56.5 | 56.5 | 1.3 | 148.83 | 323.31 | 1.3482E+10 | 169 | Unconventional myosin-VI | MYO6 |
| 26.49121 | 27.18054 | 27.26839 | 27.62492 | 31 | 31 | 31 | 34.1 | 34.1 | 34.1 | 107.78 | 323.31 | 4543000000 | 89 | Exportin-2 | CSE1L |
| 25.19787 | 26.77706 | 27.26222 | 27.72215 | 49 | 49 | 49 | 52.8 | 52.8 | 52.8 | 116.72 | 323.31 | 4162000000 | 122 | Vinculin | VCL |
| 24.61015 | 25.11688 | 27.23863 | 26.58864 | 5 | 5 | 5 | 48.8 | 48.8 | 48.8 | 14.395 | 34.757 | 396810000 | 15 | Activated RNA polymerase II transcriptional coactivator p15 | SUB1 |
| 27.91974 | 26.93101 | 27.23571 | 26.77128 | 42 | 42 | 42 | 43.5 | 43.5 | 43.5 | 135.58 | 323.31 | 1.0916E+10 | 135 | Splicing factor 3B subunit 3 | SF3B3 |
| 29.16075 | 27.35624 | 27.22812 | 27.77304 | 25 | 25 | 22 | 62.6 | 62.6 | 56.7 | 46.153 | 323.31 | 1.6151E+10 | 168 | Eukaryotic initiation factor 4A-I | EIF4A1 |
| 25.46445 | 27.08132 | 27.19131 | 27.56485 | 19 | 19 | 19 | 60.4 | 60.4 | 60.4 | 38.714 | 323.31 | 1950100000 | 51 | Annexin A1 | ANXA1 |
| 27.26114 | 26.77744 | 27.17083 | 25.89368 | 22 | 22 | 22 | 48.6 | 48.6 | 48.6 | 42.331 | 198.46 | 4525400000 | 82 | RNA-binding motif protein, X chromosome;RNA-binding motif protein, X chromosome, N-terminally processed;RNA binding motif protein, X-linked-like-1 | RBMX;RBMXL1 |
| 25.85265 | 28.70048 | 27.16 | 26.68819 | 5 | 5 | 5 | 60 | 60 | 60 | 11.665 | 80.077 | 1075300000 | 24 | 60S acidic ribosomal protein P2 | RPLP2 |
| 27.50529 | 28.76604 | 27.15567 | 26.51336 | 20 | 20 | 20 | 75.3 | 75.3 | 75.3 | 26.688 | 194.71 | 5623300000 | 79 | 40S ribosomal protein S3 | RPS3 |
| 28.35531 | 27.70088 | 27.13731 | 28.189 | 15 | 15 | 9 | 43.6 | 43.6 | 26.7 | 51.229 | 323.31 | 9804100000 | 116 | Heterogeneous nuclear ribonucleoprotein H;Heterogeneous nuclear ribonucleoprotein H, N-terminally processed | HNRNPH1 |
| 25.46995 | 26.01663 | 27.11128 | 25.98159 | 9 | 9 | 9 | 26.7 | 26.7 | 26.7 | 48.803 | 174.58 | 1782300000 | 51 | Clusterin;Clusterin beta chain;Clusterin alpha chain;Clusterin | CLU |
| 27.90231 | 26.69551 | 27.09245 | 26.63381 | 17 | 17 | 17 | 52.3 | 52.3 | 52.3 | 28.916 | 216.71 | 6857600000 | 76 | Heterogeneous nuclear ribonucleoproteins C1/C2 | HNRNPC |
| 25.73006 | 26.00127 | 27.07521 | 26.53141 | 4 | 4 | 4 | 30.8 | 30.8 | 30.8 | 15.892 | 38.459 | 910660000 | 44 | Calmodulin-like protein 5 | CALML5 |
| 25.28012 | 28.40892 | 27.06805 | 26.67519 | 6 | 6 | 6 | 36.8 | 36.8 | 36.8 | 15.798 | 39.562 | 953970000 | 18 | 60S ribosomal protein L27 | RPL27 |
| 25.98335 | 28.47448 | 27.05103 | 26.19968 | 7 | 7 | 7 | 48 | 48 | 48 | 14.463 | 50.539 | 1103500000 | 39 | 60S ribosomal protein L31 | RPL31 |
| 25.81333 | 27.41313 | 27.03769 | 26.78856 | 5 | 5 | 5 | 29.4 | 29.4 | 29.4 | 15.807 | 36.46 | 4134200000 | 24 | 40S ribosomal protein S23 | RPS23 |
| 26.32989 | 29.2395 | 27.03181 | 26.5225 | 14 | 14 | 14 | 60.6 | 60.6 | 60.6 | 15.747 | 93.584 | 1501100000 | 55 | 60S ribosomal protein L28 | RPL28 |
| 27.0185 | 26.79203 | 27.02602 | 27.49343 | 36 | 36 | 36 | 44.2 | 44.2 | 44.2 | 102 | 323.31 | 7011200000 | 102 | Staphylococcal nuclease domain-containing protein 1 | SND1 |
| 24.28905 | 25.09362 | 27.01191 | 25.29393 | 11 | 11 | 11 | 15.6 | 15.6 | 15.6 | 93.834 | 116.34 | 780790000 | 63 | Desmocollin-1 | DSC1 |
| 28.68889 | 29.864 | 27.00035 | 25.31406 | 10 | 10 | 10 | 43.1 | 43.1 | 43.1 | 24.261 | 149.09 | 4397100000 | 101 | 60S ribosomal protein L13 | RPL13 |
| 26.88565 | 27.19676 | 26.97596 | 26.00605 | 17 | 16 | 16 | 54.8 | 51.2 | 51.2 | 36.638 | 205.22 | 3745200000 | 70 | L-lactate dehydrogenase B chain;L-lactate dehydrogenase | LDHB |
| 26.10041 | 27.12445 | 26.97005 | 27.18395 | 134 | 134 | 134 | 34.1 | 34.1 | 34.1 | 532.4 | 323.31 | 8955500000 | 261 | Cytoplasmic dynein 1 heavy chain 1 | DYNC1H1 |
| 23.27994 | 25.96389 | 26.9526 | 26.18722 | 8 | 8 | 8 | 31.5 | 31.5 | 31.5 | 34.258 | 76.43 | 1349300000 | 57 | Zinc-alpha-2-glycoprotein | AZGP1 |
| 31.19102 | 27.70378 | 26.94559 | 27.19018 | 166 | 153 | 150 | 75 | 72 | 71.4 | 281.63 | 323.31 | 1.4856E+11 | 482 | Filamin-B | FLNB |
| 29.47373 | 26.69737 | 26.93663 | 26.60088 | 260 | 260 | 2 | 56.2 | 56.2 | 0.4 | 512.6 | 323.31 | 5.098E+10 | 465 | Plectin | PLEC |
| 25.38676 | 26.67277 | 26.93247 | 26.45956 | 12 | 12 | 12 | 26.7 | 26.7 | 26.7 | 33.216 | 76.867 | 1759400000 | 35 | Arginine and glutamate-rich protein 1 | ARGLU1 |
| 26.50109 | 28.77093 | 26.919 | 25.65018 | 20 | 20 | 20 | 50.5 | 50.5 | 50.5 | 34.362 | 199.58 | 5045400000 | 144 | 60S ribosomal protein L5 | RPL5 |
| 28.29535 | 26.46203 | 26.91411 | 27.12672 | 28 | 28 | 25 | 57.6 | 57.6 | 54.3 | 55.804 | 323.31 | 8249700000 | 92 | Inosine-5-monophosphate dehydrogenase 2 | IMPDH2 |
| 25.13128 | 25.4617 | 26.90208 | 25.44297 | 9 | 9 | 9 | 77.6 | 77.6 | 77.6 | 11.006 | 80.754 | 854360000 | 69 | Cystatin-A;Cystatin-A, N-terminally processed | CSTA |
| 25.8625 | 26.67128 | 26.89389 | 26.96895 | 24 | 24 | 24 | 37.2 | 37.2 | 37.2 | 84.87 | 298.66 | 3905600000 | 73 | Transferrin receptor protein 1;Transferrin receptor protein 1, serum form | TFRC |
| 27.04854 | 27.00121 | 26.8896 | 27.39632 | 21 | 21 | 21 | 54.4 | 54.4 | 54.4 | 49.541 | 281.06 | 4856400000 | 86 | Elongation factor Tu, mitochondrial | TUFM |
| 28.1923 | 30.11483 | 26.88937 | 25.48879 | 7 | 7 | 7 | 40.2 | 40.2 | 40.2 | 18.756 | 89.674 | 3604400000 | 101 | 60S ribosomal protein L18 | RPL18 |
| 27.43746 | 26.90449 | 26.88774 | 27.51134 | 44 | 42 | 39 | 61.1 | 59.6 | 56 | 66.408 | 323.31 | 6125000000 | 135 | Lamin-B1 | LMNB1 |
| 26.88204 | 27.9419 | 26.87701 | 24.91907 | 17 | 17 | 17 | 57.8 | 57.8 | 57.8 | 29.597 | 230.55 | 3475100000 | 57 | 40S ribosomal protein S4, X isoform | RPS4X |
| 27.4649 | 27.29325 | 26.84014 | 25.75453 | 9 | 9 | 9 | 41.2 | 41.2 | 41.2 | 22.127 | 67.347 | 775980000 | 39 | 40S ribosomal protein S7 | RPS7 |
| 26.99172 | 26.49097 | 26.82481 | 26.40863 | 19 | 19 | 16 | 51 | 51 | 46.3 | 57.221 | 323.31 | 4253100000 | 64 | Polypyrimidine tract-binding protein 1 | PTBP1 |
| 26.67681 | 26.49361 | 26.8158 | 26.74304 | 26 | 26 | 26 | 65.9 | 65.9 | 65.9 | 51.156 | 288.61 | 3732400000 | 75 | RuvB-like 2 | RUVBL2 |
| 26.20036 | 26.78407 | 26.81433 | 27.38821 | 22 | 22 | 22 | 37.1 | 37.1 | 37.1 | 68.569 | 222.64 | 3094900000 | 76 | Dolichyl-diphosphooligosaccharide--protein glycosyltransferase subunit 1 | RPN1 |
| 26.1787 | 26.44144 | 26.80968 | 26.44077 | 21 | 21 | 21 | 59.2 | 59.2 | 59.2 | 50.227 | 310.16 | 2681800000 | 66 | RuvB-like 1 | RUVBL1 |
| 25.67166 | 26.43302 | 26.80907 | 27.29325 | 29 | 29 | 1 | 29 | 29 | 2.2 | 106.87 | 323.31 | 3785300000 | 81 | Neutral alpha-glucosidase AB | GANAB |
| 27.16337 | 26.56176 | 26.80132 | 27.21085 | 15 | 15 | 15 | 86.3 | 86.3 | 86.3 | 22.693 | 270.06 | 4459400000 | 49 | Brain acid soluble protein 1 | BASP1 |
| 26.98316 | 26.96179 | 26.79749 | 27.18234 | 34 | 34 | 34 | 51.4 | 51.4 | 51.4 | 82.704 | 323.31 | 7043900000 | 93 | X-ray repair cross-complementing protein 5 | XRCC5 |
| 25.84369 | 25.61468 | 26.78357 | 27.11763 | 40 | 10 | 0 | 62.1 | 22.8 | 0 | 66.35 | 88.847 | 1722500000 | 86 |  | KRT17 |
| 30.38587 | 25.87736 | 26.78357 | 27.29105 | 7 | 7 | 1 | 58.6 | 58.6 | 8.6 | 13.936 | 94.21 | 5869100000 | 36 | Histone H2A type 1-J;Histone H2A type 1-H;Histone H2A.J;Histone H2A type 1-D;Histone H2A type 1;Histone H2A;Histone H2A type 2-C;Histone H2A type 2-A | HIST1H2AJ;HIST1H2AH;H2AFJ;HIST1H2AD;HIST1H2AG;HIST2H2AC;HIST2H2AA3 |
| 27.11466 | 27.73512 | 26.78207 | 26.08539 | 5 | 5 | 5 | 33.9 | 33.9 | 33.9 | 14.369 | 35.327 | 868890000 | 38 | 60S ribosomal protein L24 | RPL24 |
| 26.37091 | 26.57044 | 26.77178 | 26.97399 | 12 | 8 | 8 | 49.2 | 36 | 36 | 37.497 | 82.634 | 3091900000 | 33 | Poly(rC)-binding protein 1 | PCBP1 |
| 26.05949 | 25.9558 | 26.76155 | 26.29151 | 10 | 10 | 10 | 52.2 | 52.2 | 52.2 | 25.035 | 81.491 | 454170000 | 32 | Peroxiredoxin-6 | PRDX6 |
| 24.88679 | 26.98022 | 26.74368 | 26.35881 | 15 | 15 | 10 | 35.7 | 35.7 | 22.2 | 46.513 | 201.09 | 1734200000 | 64 | Putative RNA-binding protein Luc7-like 2;UPF0562 protein C7orf55 | LUC7L2;C7orf55 |
| 26.97574 | 27.52036 | 26.74085 | 26.99981 | 7 | 7 | 7 | 38.4 | 38.4 | 38.4 | 16.273 | 76.066 | 1063800000 | 38 | 40S ribosomal protein S14 | RPS14 |
| 26.25211 | 26.56312 | 26.7375 | 27.81372 | 8 | 8 | 8 | 69.3 | 69.3 | 69.3 | 15.054 | 72.199 | 800010000 | 28 | Profilin-1 | PFN1 |
| 25.43838 | 25.81963 | 26.72819 | 27.0425 | 7 | 7 | 1 | 50.7 | 50.7 | 9.2 | 17.149 | 53.68 | 498410000 | 28 | Nucleoside diphosphate kinase A | NME1 |
| 26.83473 | 28.66014 | 26.72754 | 26.3939 | 30 | 30 | 30 | 37.8 | 37.8 | 37.8 | 90.725 | 323.31 | 5600800000 | 85 | DNA topoisomerase 1 | TOP1 |
| 24.13203 | 26.44582 | 26.71437 | 26.50085 | 8 | 8 | 3 | 24.4 | 24.4 | 10.2 | 37.654 | 73.773 | 1908000000 | 74 | Ig alpha-1 chain C region | IGHA1 |
| 24.88981 | 28.22771 | 26.70359 | 26.61751 | 11 | 11 | 8 | 47.2 | 47.2 | 42.6 | 35.924 | 323.31 | 3373000000 | 57 | Nuclease-sensitive element-binding protein 1 | YBX1 |
| 32.27681 | 25.86733 | 26.69763 | 26.41485 | 39 | 39 | 39 | 72.2 | 72.2 | 72.2 | 54.529 | 323.31 | 9.4451E+10 | 189 | Fascin | FSCN1 |
| 28.45513 | 25.23601 | 26.69126 | 25.88835 | 8 | 8 | 4 | 52 | 52 | 29.2 | 19.794 | 84.97 | 1529200000 | 39 | Myosin regulatory light chain 12A;Myosin regulatory light chain 12B | MYL12A;MYL12B |
| 25.73877 | 27.08972 | 26.676 | 26.1148 | 6 | 6 | 6 | 40.8 | 40.8 | 40.8 | 13.742 | 38.339 | 575940000 | 21 | 40S ribosomal protein S25 | RPS25 |
| 25.78179 | 28.97451 | 26.67142 | 25.95746 | 12 | 12 | 4 | 51.7 | 51.7 | 15.2 | 17.258 | 133.95 | 1097500000 | 58 | 60S ribosomal protein L26 | RPL26;RPL26L1 |
| 27.01945 | 27.17322 | 26.66574 | 27.09818 | 16 | 16 | 16 | 40.6 | 40.6 | 40.6 | 57.861 | 323.31 | 4488700000 | 53 | Importin subunit alpha-1 | KPNA2 |
| 25.60648 | 26.55643 | 26.65976 | 26.29749 | 30 | 30 | 26 | 37.1 | 37.1 | 33.4 | 112.89 | 323.31 | 3322800000 | 76 | Sodium/potassium-transporting ATPase subunit alpha-1;Sodium/potassium-transporting ATPase subunit alpha-3 | ATP1A1;ATP1A3 |
| 25.4965 | 26.34259 | 26.6245 | 26.91809 | 20 | 20 | 20 | 33.6 | 33.6 | 33.6 | 67.567 | 219.6 | 3737100000 | 63 | Calnexin | CANX |
| 26.48777 | 27.51991 | 26.61639 | 26.50691 | 8 | 8 | 8 | 47.1 | 47.1 | 47.1 | 14.865 | 81.129 | 825810000 | 29 | 60S ribosomal protein L23 | RPL23 |
| 28.78416 | 26.5527 | 26.59761 | 26.50323 | 39 | 39 | 39 | 47.5 | 47.5 | 47.5 | 88.885 | 323.31 | 1.4761E+10 | 107 | Splicing factor 3A subunit 1 | SF3A1 |
| 27.03633 | 26.89747 | 26.58032 | 26.47686 | 13 | 13 | 13 | 44.8 | 44.8 | 44.8 | 29.724 | 146.46 | 3779900000 | 43 | Heterogeneous nuclear ribonucleoprotein D0 | HNRNPD |
| 26.67533 | 25.99194 | 26.5786 | 26.93405 | 12 | 12 | 6 | 61.5 | 61.5 | 27.8 | 22.677 | 85.451 | 535010000 | 45 | Ras-related protein Rab-1A | RAB1A |
| 27.51315 | 27.29307 | 26.56471 | 27.14063 | 25 | 25 | 25 | 48.8 | 48.8 | 48.8 | 69.842 | 323.31 | 5661000000 | 95 | X-ray repair cross-complementing protein 6 | XRCC6 |
| 26.96521 | 26.48948 | 26.55448 | 27.0238 | 13 | 11 | 11 | 39.3 | 32.8 | 32.8 | 45.671 | 323.31 | 4093800000 | 70 | Heterogeneous nuclear ribonucleoprotein F;Heterogeneous nuclear ribonucleoprotein F, N-terminally processed | HNRNPF |
| 25.82355 | 26.44427 | 26.54007 | 26.26587 | 31 | 31 | 31 | 30.6 | 30.6 | 30.6 | 134.46 | 323.31 | 4004600000 | 81 | Leucine--tRNA ligase, cytoplasmic | LARS |
| 24.81372 | 25.47376 | 26.53936 | 26.71424 | 10 | 10 | 10 | 22.6 | 22.6 | 22.6 | 56.598 | 181.75 | 1578900000 | 35 | Neutral amino acid transporter B(0) | SLC1A5 |
| 25.99008 | 28.24014 | 26.53543 | 26.59548 | 7 | 7 | 7 | 41.1 | 41.1 | 41.1 | 17.222 | 62.193 | 823430000 | 28 | 40S ribosomal protein S13 | RPS13 |
| 24.8931 | 25.76221 | 26.52966 | 25.58279 | 7 | 7 | 7 | 25.5 | 25.5 | 25.5 | 30.358 | 53.602 | 281980000 | 22 | Protein-L-isoaspartate O-methyltransferase;Protein-L-isoaspartate(D-aspartate) O-methyltransferase | PCMT1 |
| 25.84855 | 26.075 | 26.52099 | 27.02739 | 23 | 23 | 23 | 30.6 | 30.6 | 30.6 | 97.169 | 283.08 | 3615700000 | 72 | Importin subunit beta-1 | KPNB1 |
| 26.93607 | 26.4331 | 26.50647 | 26.91878 | 28 | 28 | 28 | 38.9 | 38.9 | 38.9 | 99.966 | 323.31 | 6061800000 | 86 | Matrin-3 | MATR3 |
| 26.96983 | 25.52573 | 26.50605 | 25.7357 | 33 | 33 | 17 | 43.5 | 43.5 | 26.7 | 104.55 | 323.31 | 4606100000 | 74 | AP-2 complex subunit beta | AP2B1 |
| 25.77736 | 27.22371 | 26.48464 | 26.04135 | 9 | 9 | 9 | 43.7 | 43.7 | 43.7 | 28.821 | 157.78 | 1857300000 | 43 | Elongation factor 1-delta | EEF1D |
| 25.32995 | 29.77778 | 26.48219 | 25.08857 | 30 | 30 | 30 | 55.7 | 55.7 | 55.7 | 51.294 | 318.06 | 6275000000 | 78 | Trifunctional enzyme subunit beta, mitochondrial;3-ketoacyl-CoA thiolase | HADHB |
| 24.63057 | 26.18353 | 26.4771 | 26.58778 | 9 | 9 | 9 | 15 | 15 | 15 | 55.01 | 125.68 | 1461600000 | 49 | Large neutral amino acids transporter small subunit 1 | SLC7A5 |
| 27.07235 | 28.97984 | 26.47152 | 24.57265 | 19 | 19 | 19 | 45.9 | 45.9 | 45.9 | 29.995 | 199.41 | 3879300000 | 91 | 60S ribosomal protein L7a | RPL7A |
| 25.39234 | 27.41538 | 26.46925 | 25.15684 | 7 | 7 | 7 | 35.9 | 35.9 | 35.9 | 21.863 | 49.117 | 462260000 | 25 | 60S ribosomal protein L9 | RPL9 |
| 25.97906 | 25.5362 | 26.4619 | 25.67436 | 23 | 23 | 17 | 47.5 | 47.5 | 34.8 | 65.308 | 254.12 | 2026300000 | 51 | Serine/threonine-protein phosphatase 2A 65 kDa regulatory subunit A alpha isoform | PPP2R1A |
| 26.10506 | 26.07553 | 26.45216 | 26.20645 | 30 | 30 | 30 | 37.6 | 37.6 | 37.6 | 123.63 | 323.31 | 3285300000 | 82 | Importin-5 | IPO5 |
| 25.56366 | 25.18888 | 26.43554 | 23.20174 | 15 | 15 | 8 | 37.9 | 37.9 | 19.8 | 32.852 | 122.51 | 1422600000 | 34 | ADP/ATP translocase 2;ADP/ATP translocase 2, N-terminally processed | SLC25A5 |
| 24.08877 | 25.4022 | 26.42921 | 26.74201 | 17 | 17 | 17 | 35.8 | 35.8 | 35.8 | 47.517 | 150.2 | 1128000000 | 49 | Aspartate aminotransferase, mitochondrial | GOT2 |
| 26.48348 | 26.08952 | 26.4259 | 26.71463 | 39 | 39 | 38 | 41.4 | 41.4 | 40.6 | 101.56 | 323.31 | 4386600000 | 91 | C-1-tetrahydrofolate synthase, cytoplasmic;Methylenetetrahydrofolate dehydrogenase;Methenyltetrahydrofolate cyclohydrolase;Formyltetrahydrofolate synthetase;C-1-tetrahydrofolate synthase, cytoplasmic, N-terminally processed | MTHFD1 |
| 25.59126 | 26.50762 | 26.42132 | 27.48884 | 31 | 31 | 31 | 42.6 | 42.6 | 42.6 | 83.434 | 296.23 | 3922800000 | 83 | Threonine--tRNA ligase, cytoplasmic | TARS |
| 26.55535 | 27.99335 | 26.41976 | 24.77067 | 20 | 20 | 20 | 63.3 | 63.3 | 63.3 | 29.945 | 235.91 | 3163900000 | 71 | 40S ribosomal protein S3a | RPS3A |
| 28.21211 | 25.31029 | 26.4195 | 25.39103 | 19 | 19 | 19 | 42.6 | 42.6 | 42.6 | 57.304 | 297.51 | 9476900000 | 67 | Alkaline phosphatase, tissue-nonspecific isozyme | ALPL |
| 25.32536 | 26.26069 | 26.41677 | 26.91764 | 29 | 29 | 29 | 29.1 | 29.1 | 29.1 | 126.22 | 323.31 | 3370800000 | 88 | ATP-citrate synthase | ACLY |
| 26.83629 | 25.60848 | 26.41421 | 25.73319 | 38 | 38 | 29 | 50.2 | 50.2 | 41.4 | 105.36 | 323.31 | 6934000000 | 79 | AP-2 complex subunit alpha-1 | AP2A1 |
| 24.32052 | 25.3508 | 26.38349 | 26.59263 | 15 | 15 | 15 | 45.9 | 45.9 | 45.9 | 38.242 | 112.4 | 997770000 | 43 | Nuclear migration protein nudC | NUDC |
| 31.18104 | 26.96664 | 26.36189 | 27.24309 | 7 | 7 | 0 | 38 | 38 | 0 | 18.041 | 85.837 | 8411100000 | 50 | Histone H2B;Histone H2B type 1-H;Histone H2B type 2-F;Histone H2B type 1-L;Histone H2B type 1-M;Histone H2B type 1-N | HIST1H2BH;HIST2H2BF;HIST1H2BL;HIST1H2BM;HIST1H2BN |
| 25.46173 | 26.83352 | 26.34208 | 25.30689 | 3 | 3 | 3 | 27 | 27 | 27 | 13.015 | 20.114 | 454730000 | 14 | 40S ribosomal protein S26;Putative 40S ribosomal protein S26-like 1 | RPS26;RPS26P11 |
| 25.52465 | 26.42944 | 26.32884 | 26.59392 | 23 | 23 | 23 | 40.3 | 40.3 | 40.3 | 75.378 | 209.21 | 2078600000 | 74 | Arginine--tRNA ligase, cytoplasmic | RARS |
| 32.85313 | 26.50311 | 26.32052 | 24.85343 | 46 | 46 | 5 | 88.7 | 88.7 | 13.3 | 29.032 | 323.31 | 1.5133E+11 | 227 | Tropomyosin alpha-3 chain | TPM3 |
| 23.60727 | 25.45795 | 26.31349 | 25.22534 | 6 | 6 | 6 | 45.9 | 45.9 | 45.9 | 16.572 | 49.703 | 771920000 | 59 | Prolactin-inducible protein | PIP |
| 25.44452 | 26.47831 | 26.31337 | 27.13291 | 22 | 22 | 16 | 55.7 | 55.7 | 40.8 | 51.18 | 285.06 | 2162900000 | 65 | Rab GDP dissociation inhibitor beta | GDI2 |
| 25.77769 | 26.01907 | 26.30201 | 26.89631 | 8 | 8 | 8 | 39 | 39 | 39 | 26.669 | 84.469 | 449850000 | 32 | Triosephosphate isomerase | TPI1 |
| 25.51274 | 25.22468 | 26.29493 | 25.77286 | 18 | 18 | 18 | 35.9 | 35.9 | 35.9 | 47.716 | 174.82 | 1255200000 | 60 | Adenosylhomocysteinase | AHCY |
| 26.19513 | 25.93708 | 26.29005 | 25.94889 | 17 | 17 | 17 | 57.9 | 57.9 | 57.9 | 39.777 | 278.21 | 1997100000 | 45 | Serine-threonine kinase receptor-associated protein | STRAP |
| 27.25115 | 26.8642 | 26.28304 | 27.27817 | 6 | 6 | 6 | 27.5 | 27.5 | 27.5 | 26.224 | 47.204 | 885120000 | 41 | GTP-binding nuclear protein Ran | RAN |
| 26.25981 | 26.44293 | 26.26844 | 26.68552 | 20 | 20 | 20 | 52.8 | 52.8 | 52.8 | 43.786 | 323.31 | 2738300000 | 86 | Proliferation-associated protein 2G4 | PA2G4 |
| 31.76315 | 26.07194 | 26.26309 | 24.2501 | 106 | 106 | 102 | 64.9 | 64.9 | 62.7 | 189.25 | 323.31 | 6.6396E+10 | 267 | Ras GTPase-activating-like protein IQGAP1 | IQGAP1 |
| 26.12873 | 27.5952 | 26.26152 | 25.51422 | 31 | 31 | 28 | 50 | 50 | 46.4 | 75.508 | 323.31 | 5362000000 | 92 | Interleukin enhancer-binding factor 3 | ILF3 |
| 25.21878 | 25.81438 | 26.25314 | 26.49855 | 19 | 19 | 19 | 45.6 | 45.6 | 45.6 | 56.157 | 323.31 | 1679300000 | 49 | Succinyl-CoA:3-ketoacid coenzyme A transferase 1, mitochondrial | OXCT1 |
| 24.94217 | 25.55415 | 26.24294 | 26.41282 | 7 | 7 | 7 | 44.4 | 44.4 | 44.4 | 15.164 | 50.531 | 644810000 | 67 | Fatty acid-binding protein, epidermal | FABP5 |
| 26.04041 | 29.59146 | 26.23688 | 25.22902 | 20 | 20 | 20 | 53.8 | 53.8 | 53.8 | 32.728 | 194.81 | 5445700000 | 119 | 60S ribosomal protein L6 | RPL6 |
| 24.5566 | 26.03309 | 26.23476 | 25.89493 | 50 | 50 | 50 | 27 | 27 | 27 | 244.5 | 323.31 | 3921900000 | 101 | U5 small nuclear ribonucleoprotein 200 kDa helicase | SNRNP200 |
| 25.8043 | 27.51232 | 26.20208 | 24.66904 | 6 | 6 | 6 | 34.4 | 34.4 | 34.4 | 18.565 | 45.426 | 470250000 | 20 | 60S ribosomal protein L21 | RPL21 |
| 27.31562 | 25.23677 | 26.20135 | 25.16469 | 40 | 40 | 35 | 36.4 | 36.4 | 31.4 | 127.06 | 323.31 | 5884000000 | 76 | Unconventional myosin-Ie | MYO1E |
| 28.13702 | 29.62991 | 26.19458 | 27.24391 | 61 | 61 | 61 | 48 | 48 | 48 | 140.96 | 323.31 | 9499200000 | 194 | ATP-dependent RNA helicase A | DHX9 |
| 22.97392 | 25.27245 | 26.17967 | 24.98485 | 7 | 4 | 4 | 7.6 | 4.2 | 4.2 | 95.949 | 27.215 | 785320000 | 32 | Glycogen phosphorylase, muscle form | PYGM |
| 27.10779 | 27.31225 | 26.17413 | 25.70137 | 12 | 12 | 12 | 39.7 | 39.7 | 39.7 | 22.876 | 85.855 | 688390000 | 41 | 40S ribosomal protein S5;40S ribosomal protein S5, N-terminally processed | RPS5 |
| 25.89437 | 28.25539 | 26.16657 | 25.40133 | 9 | 9 | 9 | 40.2 | 40.2 | 40.2 | 13.293 | 58.893 | 775110000 | 28 | 60S ribosomal protein L34 | RPL34 |
| 25.48916 | 28.41776 | 26.16546 | 25.73451 | 6 | 6 | 6 | 40.7 | 40.7 | 40.7 | 12.201 | 43.334 | 808010000 | 27 | 60S ribosomal protein L27a | RPL27A |
| 26.85828 | 24.48573 | 26.15831 | 24.78995 | 44 | 44 | 44 | 47 | 47 | 47 | 109.16 | 323.31 | 4945300000 | 82 | Ankycorbin | RAI14 |
| 25.46392 | 24.26757 | 26.15779 | 26.59463 | 12 | 7 | 7 | 43.7 | 26.9 | 26.9 | 27.764 | 108.32 | 500830000 | 40 | 14-3-3 protein theta | YWHAQ |
| 28.01739 | 26.00676 | 26.1531 | 26.31638 | 28 | 28 | 26 | 43.7 | 43.7 | 42.3 | 76.149 | 323.31 | 9112300000 | 93 | Splicing factor, proline- and glutamine-rich | SFPQ |
| 26.0973 | 25.78992 | 26.15167 | 24.99499 | 13 | 13 | 13 | 34.7 | 34.7 | 34.7 | 43.66 | 178.85 | 2067400000 | 47 | S-adenosylmethionine synthase isoform type-2 | MAT2A |
| 25.16296 | 25.83248 | 26.13899 | 26.13587 | 18 | 18 | 18 | 39.5 | 39.5 | 39.5 | 51.83 | 266.27 | 1519600000 | 59 | Adenylyl cyclase-associated protein 1 | CAP1 |
| 26.70491 | 28.72198 | 26.1344 | 24.47667 | 13 | 13 | 13 | 40.6 | 40.6 | 40.6 | 28.68 | 160.62 | 3144100000 | 67 | 40S ribosomal protein S6 | RPS6 |
| 25.42432 | 26.01952 | 26.12879 | 26.50815 | 17 | 17 | 16 | 35 | 35 | 32.1 | 53.454 | 164.93 | 1768500000 | 58 | Serine hydroxymethyltransferase, mitochondrial;Serine hydroxymethyltransferase | SHMT2 |
| 25.77924 | 25.13418 | 26.12143 | 25.93605 | 8 | 7 | 7 | 39.9 | 34.3 | 34.3 | 21.892 | 42.707 | 333660000 | 33 | Peroxiredoxin-2 | PRDX2 |
| 22.83051 | 25.23422 | 26.09688 | 25.69559 | 5 | 5 | 5 | 80.2 | 80.2 | 80.2 | 11.609 | 98.012 | 808590000 | 46 | Ig kappa chain C region | IGKC |
| 28.10869 | 26.68217 | 26.09132 | 26.1475 | 25 | 23 | 22 | 56.5 | 54.4 | 52.9 | 54.231 | 323.31 | 8415400000 | 110 | Non-POU domain-containing octamer-binding protein | NONO |
| 28.23293 | 26.36052 | 26.09116 | 25.77354 | 33 | 32 | 32 | 52.4 | 52.4 | 52.4 | 73.243 | 323.31 | 7254600000 | 120 | ATP-dependent RNA helicase DDX3X;ATP-dependent RNA helicase DDX3Y | DDX3X;DDX3Y |
| 25.69511 | 27.07143 | 26.04679 | 25.67436 | 7 | 7 | 7 | 46.4 | 46.4 | 46.4 | 12.538 | 42.215 | 479630000 | 21 | 60S ribosomal protein L35a | RPL35A |
| 32.2378 | 25.62207 | 26.02905 | 25.60552 | 74 | 74 | 29 | 77.1 | 77.1 | 31.6 | 104.85 | 323.31 | 1.388E+11 | 204 | Alpha-actinin-4 | ACTN4 |
| 27.92773 | 27.84972 | 26.026 | 27.09356 | 34 | 34 | 25 | 50.5 | 50.5 | 36.5 | 69.147 | 323.31 | 7141400000 | 133 | Probable ATP-dependent RNA helicase DDX5 | DDX5 |
| 23.37159 | 26.20452 | 26.00185 | 26.18554 | 60 | 60 | 60 | 28 | 28 | 28 | 267.29 | 323.31 | 1831500000 | 117 | Nucleoprotein TPR | TPR |
| 25.75093 | 26.72689 | 25.99882 | 25.95755 | 8 | 8 | 8 | 70.5 | 70.5 | 70.5 | 14.515 | 84.356 | 521500000 | 34 | 40S ribosomal protein S12 | RPS12 |
| 26.39013 | 27.34426 | 25.99734 | 24.63959 | 16 | 16 | 16 | 51.5 | 51.5 | 51.5 | 31.324 | 138.21 | 2094500000 | 70 | 40S ribosomal protein S2 | RPS2 |
| 26.60201 | 26.86997 | 25.99298 | 25.86978 | 52 | 52 | 52 | 48.4 | 48.4 | 48.4 | 138.34 | 323.31 | 2648100000 | 93 | Coatomer subunit alpha;Xenin;Proxenin | COPA |
| 25.33361 | 25.55964 | 25.98947 | 26.15219 | 4 | 4 | 4 | 13.7 | 13.7 | 13.7 | 36.085 | 37.197 | 4151300000 | 11 | Lymphokine-activated killer T-cell-originated protein kinase | PBK |
| 26.8889 | 26.89296 | 25.96963 | 26.50711 | 20 | 20 | 1 | 36.3 | 36.3 | 2.6 | 69.602 | 290.85 | 3923500000 | 82 | Heterogeneous nuclear ribonucleoprotein Q | SYNCRIP |
| 21.38843 | 24.63456 | 25.96943 | 23.28785 | 8 | 8 | 8 | 22.2 | 22.2 | 22.2 | 46.276 | 61.942 | 422410000 | 34 | Serpin B12 | SERPINB12 |
| 24.41944 | 25.15198 | 25.96217 | 25.93634 | 14 | 14 | 14 | 35.7 | 35.7 | 35.7 | 51.804 | 147.72 | 1123800000 | 38 | Peptidyl-prolyl cis-trans isomerase FKBP4;Peptidyl-prolyl cis-trans isomerase FKBP4, N-terminally processed | FKBP4 |
| 27.74362 | 25.6531 | 25.95236 | 26.14447 | 14 | 14 | 12 | 66.1 | 66.1 | 60.1 | 32.922 | 287.12 | 5488000000 | 49 | F-actin-capping protein subunit alpha-1 | CAPZA1 |
| 24.24009 | 24.854 | 25.94347 | 25.16507 | 13 | 13 | 12 | 38.2 | 38.2 | 36 | 46.659 | 100.87 | 720810000 | 42 | Isocitrate dehydrogenase [NADP] cytoplasmic | IDH1 |
| 23.56337 | 25.59599 | 25.94306 | 26.34687 | 10 | 6 | 6 | 25.8 | 21.4 | 21.4 | 39.455 | 107.52 | 886480000 | 25 | Fructose-bisphosphate aldolase C | ALDOC |
| 22.59341 | 24.82949 | 25.93991 | 25.03065 | 3 | 3 | 3 | 21.7 | 21.7 | 21.7 | 14.246 | 23.994 | 586810000 | 21 | Extracellular glycoprotein lacritin | LACRT;AXIN2 |
| 25.56124 | 26.46279 | 25.93071 | 25.46613 | 8 | 8 | 8 | 43.8 | 43.8 | 43.8 | 16.445 | 53.364 | 423150000 | 23 | 40S ribosomal protein S16 | RPS16 |
| 28.35775 | 25.08898 | 25.92796 | 26.34299 | 10 | 10 | 10 | 46.2 | 46.2 | 46.2 | 22.391 | 73.587 | 958320000 | 52 | Transgelin-2 | TAGLN2 |
| 24.16231 | 26.29404 | 25.92631 | 25.71345 | 45 | 45 | 45 | 25.6 | 25.6 | 25.6 | 236.51 | 323.31 | 2687200000 | 93 | Nuclear mitotic apparatus protein 1 | NUMA1 |
| 25.70575 | 25.97362 | 25.92567 | 26.09633 | 30 | 30 | 30 | 39.9 | 39.9 | 39.9 | 114.76 | 323.31 | 3036600000 | 77 | Sarcoplasmic/endoplasmic reticulum calcium ATPase 2 | ATP2A2 |
| 25.80033 | 26.03666 | 25.91752 | 26.32098 | 24 | 24 | 24 | 49.2 | 49.2 | 49.2 | 88.549 | 323.31 | 3283600000 | 69 | Transcription intermediary factor 1-beta | TRIM28 |
| 26.00316 | 27.89145 | 25.90424 | 25.54159 | 7 | 7 | 7 | 59.6 | 59.6 | 59.6 | 12.656 | 58.114 | 711080000 | 24 | 60S ribosomal protein L30 | RPL30 |
| 25.82549 | 25.21201 | 25.89567 | 26.12469 | 27 | 27 | 27 | 37.9 | 37.9 | 37.9 | 99.271 | 323.31 | 3011600000 | 60 | 26S proteasome non-ATPase regulatory subunit 2 | PSMD2 |
| 26.4164 | 27.61905 | 25.88837 | 25.12818 | 33 | 33 | 31 | 46.2 | 46.2 | 42.8 | 87.343 | 323.31 | 5516900000 | 74 | Nucleolar RNA helicase 2 | DDX21 |
| 26.18728 | 25.2696 | 25.86391 | 26.56652 | 12 | 12 | 12 | 27.5 | 27.5 | 27.5 | 40.538 | 78.223 | 2027200000 | 59 | Basic leucine zipper and W2 domain-containing protein 1 | BZW1 |
| 25.10821 | 25.7541 | 25.85961 | 26.05101 | 46 | 43 | 43 | 26.3 | 25.2 | 25.2 | 236.02 | 323.31 | 3146600000 | 104 | CAD protein;Glutamine-dependent carbamoyl-phosphate synthase;Aspartate carbamoyltransferase;Dihydroorotase | CAD |
| 22.73453 | 24.92701 | 25.84781 | 24.41532 | 17 | 17 | 17 | 22.8 | 22.8 | 22.8 | 89.466 | 105.06 | 617360000 | 42 | MAP7 domain-containing protein 1 | MAP7D1 |
| 24.23798 | 25.53163 | 25.84515 | 25.97112 | 16 | 16 | 16 | 40.5 | 40.5 | 40.5 | 50.212 | 142.26 | 1120800000 | 33 | Fumarate hydratase, mitochondrial | FH |
| 24.95806 | 25.42624 | 25.83203 | 25.95338 | 10 | 10 | 8 | 30.8 | 30.8 | 28 | 44.162 | 166.55 | 1583500000 | 32 | Nucleosome assembly protein 1-like 4 | NAP1L4 |
| 25.10225 | 24.92769 | 25.81729 | 25.64642 | 18 | 18 | 18 | 35.1 | 35.1 | 35.1 | 66.294 | 174.92 | 1088100000 | 39 | Apoptosis-inducing factor 1, mitochondrial | AIFM1 |
| 26.34382 | 26.55633 | 25.80181 | 26.29731 | 50 | 50 | 49 | 36.2 | 36.2 | 35.7 | 166.57 | 323.31 | 2365400000 | 91 | Eukaryotic translation initiation factor 3 subunit A | EIF3A |
| 25.85474 | 27.9718 | 25.78938 | 25.49622 | 10 | 10 | 10 | 45.1 | 45.1 | 45.1 | 21.397 | 66.043 | 612260000 | 29 | 60S ribosomal protein L17 | RPL17 |
| 26.52477 | 26.23794 | 25.78853 | 26.62909 | 24 | 4 | 4 | 63.5 | 12.5 | 12.5 | 48.328 | 54.67 | 2483200000 | 20 | Tubulin alpha-4A chain | TUBA4A |
| 23.92162 | 25.66221 | 25.78492 | 24.04034 | 8 | 8 | 8 | 18.9 | 18.9 | 18.9 | 50.8 | 101.25 | 663790000 | 20 | Dolichyl-diphosphooligosaccharide--protein glycosyltransferase 48 kDa subunit | DDOST |
| 25.39751 | 26.46482 | 25.78157 | 26.17969 | 18 | 18 | 18 | 56.5 | 56.5 | 56.5 | 36.112 | 162.57 | 1798800000 | 75 | Eukaryotic translation initiation factor 2 subunit 1 | EIF2S1 |
| 24.99287 | 25.48139 | 25.78102 | 26.31897 | 30 | 30 | 30 | 39.2 | 39.2 | 39.2 | 106.81 | 323.31 | 2244700000 | 61 | Alanine--tRNA ligase, cytoplasmic | AARS |
| 25.70928 | 26.95649 | 25.76304 | 26.34189 | 20 | 20 | 20 | 51.5 | 51.5 | 51.5 | 51.109 | 222.55 | 2140900000 | 56 | Eukaryotic translation initiation factor 2 subunit 3;Putative eukaryotic translation initiation factor 2 subunit 3-like protein | EIF2S3;EIF2S3L |
| 26.62171 | 25.7982 | 25.75205 | 25.62282 | 15 | 15 | 15 | 40.6 | 40.6 | 40.6 | 64.132 | 228.13 | 2796700000 | 44 | Heterogeneous nuclear ribonucleoprotein L | HNRNPL |
| 25.06977 | 25.3279 | 25.75037 | 25.62979 | 9 | 9 | 9 | 24.1 | 24.1 | 24.1 | 37.106 | 128.03 | 975130000 | 36 | Calumenin | CALU |
| 27.57514 | 25.52085 | 25.74343 | 25.78234 | 11 | 11 | 11 | 59 | 59 | 59 | 22.782 | 143.38 | 1020400000 | 47 | Heat shock protein beta-1 | HSPB1 |
| 24.47005 | 25.78219 | 25.73379 | 25.8905 | 16 | 16 | 14 | 28.9 | 28.9 | 27.5 | 66.231 | 255.54 | 1811300000 | 52 | Far upstream element-binding protein 1 | FUBP1 |
| 26.91775 | 26.31216 | 25.72416 | 25.92927 | 33 | 33 | 33 | 37.6 | 37.6 | 37.6 | 113.08 | 323.31 | 5346300000 | 77 | Poly [ADP-ribose] polymerase 1 | PARP1 |
| 26.68043 | 26.09826 | 25.72148 | 25.47664 | 29 | 29 | 28 | 38 | 38 | 36.9 | 89.502 | 307.82 | 4261600000 | 72 | Putative pre-mRNA-splicing factor ATP-dependent RNA helicase DHX15 | DHX15 |
| 25.75506 | 25.75672 | 25.71508 | 26.02678 | 18 | 18 | 18 | 41.4 | 41.4 | 41.4 | 47.079 | 191.66 | 1601700000 | 61 | Multifunctional protein ADE2;Phosphoribosylaminoimidazole-succinocarboxamide synthase;Phosphoribosylaminoimidazole carboxylase | PAICS |
| 26.89747 | 25.81538 | 25.70893 | 25.36973 | 33 | 33 | 33 | 44.9 | 44.9 | 44.9 | 100.23 | 323.31 | 5158600000 | 74 | Splicing factor 3B subunit 2 | SF3B2 |
| 24.29796 | 25.31969 | 25.68977 | 25.73319 | 13 | 13 | 13 | 31.9 | 31.9 | 31.9 | 51.872 | 125.78 | 953560000 | 31 | 6-phosphogluconate dehydrogenase, decarboxylating | PGD |
| 24.33067 | 25.32444 | 25.68378 | 26.62422 | 11 | 11 | 11 | 28.1 | 28.1 | 28.1 | 47.837 | 147.72 | 1191100000 | 25 | Protein disulfide-isomerase A6 | PDIA6 |
| 24.65813 | 24.15298 | 25.68024 | 24.81739 | 6 | 6 | 6 | 23.1 | 23.1 | 23.1 | 27.679 | 55.333 | 374960000 | 57 | Caspase-14;Caspase-14 subunit p19;Caspase-14 subunit p10 | CASP14 |
| 24.55028 | 24.87371 | 25.66449 | 25.23951 | 16 | 16 | 16 | 44.8 | 44.8 | 44.8 | 55.399 | 170.17 | 1102300000 | 34 | Poly(U)-binding-splicing factor PUF60 | PUF60 |
| 27.40243 | 30.11855 | 25.65728 | 25.04214 | 6 | 6 | 6 | 25 | 25 | 25 | 24.146 | 44.557 | 2412500000 | 52 | 60S ribosomal protein L15;Ribosomal protein L15 | RPL15 |
| 25.1029 | 24.85424 | 25.6539 | 24.11044 | 12 | 12 | 12 | 30.8 | 30.8 | 30.8 | 36.034 | 99.599 | 840950000 | 46 | Very-long-chain enoyl-CoA reductase | TECR |
| 25.31091 | 25.32838 | 25.65177 | 25.87446 | 17 | 17 | 17 | 35.7 | 35.7 | 35.7 | 59.256 | 177.67 | 1414100000 | 33 | Glucose-6-phosphate 1-dehydrogenase | G6PD |
| 25.37541 | 25.49433 | 25.64787 | 24.47333 | 13 | 13 | 13 | 37.4 | 37.4 | 37.4 | 39.958 | 120.09 | 1191700000 | 41 | Phosphate carrier protein, mitochondrial | SLC25A3 |
| 25.40074 | 26.02102 | 25.64238 | 26.37327 | 10 | 10 | 5 | 23.7 | 23.7 | 13.2 | 46.158 | 197.15 | 1877600000 | 31 | Histone-binding protein RBBP4 | RBBP4 |
| 23.82503 | 25.24481 | 25.63675 | 25.75628 | 13 | 13 | 13 | 41 | 41 | 41 | 45.199 | 208.56 | 792240000 | 33 | Acetyl-CoA acetyltransferase, mitochondrial | ACAT1 |
| 23.41455 | 25.3064 | 25.63229 | 24.39506 | 8 | 8 | 8 | 11.4 | 11.4 | 11.4 | 64.135 | 53.166 | 422520000 | 32 | Keratinocyte proline-rich protein | KPRP |
| 24.55614 | 25.67812 | 25.62639 | 25.38278 | 20 | 20 | 20 | 38.2 | 38.2 | 38.2 | 74.267 | 180.32 | 1175100000 | 59 | Heat shock protein 75 kDa, mitochondrial | TRAP1 |
| 24.90573 | 24.81719 | 25.62439 | 27.01563 | 25 | 15 | 14 | 42.8 | 24.7 | 23.4 | 81.744 | 136.46 | 1201400000 | 85 | Junction plakoglobin | JUP |
| 24.22634 | 25.36085 | 25.62232 | 25.38943 | 14 | 14 | 14 | 37.8 | 37.8 | 37.8 | 54.286 | 124.78 | 939250000 | 42 | Glutamate dehydrogenase 1, mitochondrial;Glutamate dehydrogenase 2, mitochondrial | GLUD1;GLUD2 |
| 25.11279 | 24.92098 | 25.62062 | 25.71952 | 18 | 18 | 18 | 49.9 | 49.9 | 49.9 | 44.172 | 186.46 | 1228800000 | 52 | 26S protease regulatory subunit 10B | PSMC6 |
| 22.589 | 24.85281 | 25.60659 | 24.96336 | 11 | 11 | 11 | 28.9 | 28.9 | 28.9 | 40.675 | 72.493 | 476120000 | 27 | Protein-L-isoaspartate O-methyltransferase domain-containing protein 1 | PCMTD1 |
| 25.2832 | 25.38423 | 25.60374 | 25.38004 | 20 | 20 | 20 | 48.5 | 48.5 | 48.5 | 48.633 | 201.35 | 1385900000 | 49 | 26S protease regulatory subunit 7 | PSMC2 |
| 24.33912 | 26.30454 | 25.60317 | 26.42659 | 27 | 27 | 16 | 44.9 | 44.9 | 28.2 | 67.819 | 298.01 | 2300800000 | 76 | Moesin | MSN |
| 24.56005 | 25.08687 | 25.60315 | 25.26148 | 13 | 13 | 13 | 24.4 | 24.4 | 24.4 | 74.175 | 147.06 | 890940000 | 31 | Calcium-binding mitochondrial carrier protein Aralar2 | SLC25A13 |
| 24.95567 | 25.55596 | 25.59989 | 23.67799 | 18 | 17 | 17 | 28.1 | 28.1 | 28.1 | 82.245 | 323.31 | 1448200000 | 39 | Glutamine--tRNA ligase | QARS |
| 24.29362 | 25.23129 | 25.5977 | 25.17509 | 16 | 16 | 16 | 36.1 | 36.1 | 36.1 | 64.732 | 154.58 | 884300000 | 33 | Polypeptide N-acetylgalactosaminyltransferase 2;Polypeptide N-acetylgalactosaminyltransferase 2 soluble form | GALNT2 |
| 27.20172 | 24.59503 | 25.59255 | 25.43597 | 12 | 12 | 12 | 33.1 | 33.1 | 33.1 | 35.695 | 96.244 | 3448000000 | 45 | Complement decay-accelerating factor | CD55 |
| 24.27739 | 25.65529 | 25.59175 | 26.14672 | 23 | 23 | 23 | 37.5 | 37.5 | 37.5 | 72.932 | 250.34 | 1818600000 | 50 | Protein disulfide-isomerase A4 | PDIA4 |
| 25.34215 | 26.63519 | 25.59055 | 25.29309 | 4 | 4 | 4 | 28.6 | 28.6 | 28.6 | 13.373 | 44.54 | 393030000 | 22 | 40S ribosomal protein S20 | RPS20 |
| 26.43382 | 28.77307 | 25.58875 | 25.26395 | 6 | 6 | 6 | 48.4 | 48.4 | 48.4 | 14.558 | 46.929 | 1109500000 | 45 | 60S ribosomal protein L14 | RPL14 |
| 27.11882 | 23.72733 | 25.58354 | 23.21433 | 36 | 36 | 36 | 35.2 | 35.2 | 35.2 | 115.28 | 323.31 | 3899500000 | 65 | Protein phosphatase 1 regulatory subunit 12A | PPP1R12A |
| 24.47754 | 25.46538 | 25.57335 | 25.66568 | 57 | 57 | 57 | 26.6 | 26.6 | 26.6 | 292.75 | 323.31 | 2902500000 | 103 | Translational activator GCN1 | GCN1L1 |
| 24.28256 | 25.93809 | 25.56877 | 26.43951 | 9 | 9 | 9 | 18.3 | 18.3 | 18.3 | 59.177 | 96.995 | 1494400000 | 60 | Glucosidase 2 subunit beta | PRKCSH |
| 26.6637 | 26.65034 | 25.56005 | 26.29204 | 15 | 15 | 15 | 40.7 | 40.7 | 40.7 | 43.135 | 274.15 | 2789200000 | 60 | Plasminogen activator inhibitor 1 RNA-binding protein | SERBP1 |
| 24.08058 | 24.99451 | 25.5443 | 25.6907 | 6 | 6 | 6 | 14.7 | 14.7 | 14.7 | 54.489 | 61.251 | 620520000 | 20 | 7-dehydrocholesterol reductase | DHCR7 |
| 26.77493 | 27.209 | 25.53985 | 26.80538 | 26 | 26 | 18 | 45.1 | 45.1 | 28.8 | 70.67 | 323.31 | 4178300000 | 109 | Polyadenylate-binding protein 1 | PABPC1 |
| 26.26024 | 26.35984 | 25.53964 | 25.67538 | 7 | 7 | 7 | 22.9 | 22.9 | 22.9 | 30.302 | 79.517 | 1906700000 | 27 | Heterogeneous nuclear ribonucleoprotein A/B | HNRNPAB |
| 25.47914 | 25.9009 | 25.53848 | 25.25908 | 12 | 12 | 12 | 38.3 | 38.3 | 38.3 | 55.18 | 205.49 | 1487300000 | 39 | Pre-mRNA-processing factor 19 | PRPF19 |
| 24.35057 | 25.06534 | 25.53089 | 26.05879 | 19 | 19 | 19 | 29.4 | 29.4 | 29.4 | 85.238 | 323.31 | 1956500000 | 62 | Cytosolic phospholipase A2;Phospholipase A2;Lysophospholipase | PLA2G4A |
| 24.9872 | 25.17877 | 25.52955 | 25.66047 | 18 | 18 | 18 | 28.1 | 28.1 | 28.1 | 101.11 | 264.66 | 1881100000 | 46 | Methionine--tRNA ligase, cytoplasmic | MARS |
| 25.17722 | 27.31907 | 25.51406 | 24.74919 | 5 | 5 | 2 | 37.7 | 37.7 | 9.4 | 12.469 | 36.759 | 437560000 | 23 | 60S ribosomal protein L36a-like | RPL36AL |
| 25.70123 | 24.23863 | 25.51072 | 23.99309 | 17 | 17 | 17 | 42 | 42 | 42 | 49.389 | 114.19 | 1336500000 | 38 | AP-2 complex subunit mu | AP2M1 |
| 24.19099 | 25.58035 | 25.50652 | 25.3887 | 11 | 11 | 11 | 33.8 | 33.8 | 33.8 | 37.54 | 89.33 | 868690000 | 19 | Transaldolase | TALDO1 |
| 24.51075 | 24.6212 | 25.50473 | 24.84871 | 9 | 9 | 9 | 19.5 | 19.5 | 19.5 | 69.283 | 140.12 | 766260000 | 21 | Dolichyl-diphosphooligosaccharide--protein glycosyltransferase subunit 2 | RPN2 |
| 25.04735 | 25.77072 | 25.4887 | 26.35072 | 14 | 14 | 14 | 45.6 | 45.6 | 45.6 | 44.204 | 179.25 | 1601700000 | 41 | Serpin H1 | SERPINH1 |
| 24.50948 | 24.57259 | 25.48735 | 25.57109 | 9 | 9 | 9 | 24.4 | 24.4 | 24.4 | 48.881 | 107.59 | 995480000 | 27 | Dihydrolipoyl dehydrogenase, mitochondrial | DLD |
| 24.77037 | 26.66112 | 25.47754 | 27.08699 | 33 | 33 | 33 | 28.3 | 28.3 | 28.3 | 156.27 | 275.56 | 1321100000 | 66 | Kinectin | KTN1 |
| 26.70121 | 25.52758 | 25.47534 | 25.85626 | 25 | 22 | 22 | 62.3 | 56.4 | 56.4 | 46.871 | 265.23 | 3233200000 | 72 | Eukaryotic initiation factor 4A-III;Eukaryotic initiation factor 4A-III, N-terminally processed | EIF4A3 |
| 26.2703 | 26.016 | 25.46585 | 25.33398 | 20 | 20 | 20 | 41.4 | 41.4 | 41.4 | 61.332 | 309.31 | 2818400000 | 63 | Methylcrotonoyl-CoA carboxylase beta chain, mitochondrial | MCCC2 |
| 23.78157 | 27.39673 | 25.45742 | 24.21134 | 13 | 13 | 13 | 35.2 | 35.2 | 35.2 | 40.684 | 109.57 | 974670000 | 27 | Probable rRNA-processing protein EBP2 | EBNA1BP2 |
| 28.3265 | 26.57427 | 25.45701 | 26.21987 | 59 | 59 | 59 | 51.8 | 51.8 | 51.8 | 145.83 | 323.31 | 6611900000 | 128 | Splicing factor 3B subunit 1 | SF3B1 |
| 23.44101 | 24.46694 | 25.44916 | 25.5613 | 9 | 9 | 9 | 22.4 | 22.4 | 22.4 | 38.89 | 101.56 | 553520000 | 28 | Reticulocalbin-1 | RCN1 |
| 30.88431 | 23.63666 | 25.43304 | 24.37776 | 42 | 31 | 0 | 81.7 | 64.8 | 0 | 32.989 | 323.31 | 4.5891E+10 | 140 | Tropomyosin beta chain | TPM2 |
| 24.39676 | 25.18488 | 25.43087 | 26.03534 | 14 | 14 | 14 | 18.3 | 18.3 | 18.3 | 88.414 | 111.97 | 1388800000 | 34 | Integrin beta-1 | ITGB1 |
| 26.55982 | 25.13387 | 25.42755 | 26.1702 | 8 | 8 | 8 | 71.9 | 71.9 | 71.9 | 14.716 | 73.394 | 483160000 | 36 | Galectin-1 | LGALS1 |
| 25.38169 | 25.11239 | 25.42701 | 25.14663 | 22 | 22 | 22 | 31.4 | 31.4 | 31.4 | 92.888 | 225.94 | 2050300000 | 37 | DNA replication licensing factor MCM6 | MCM6 |
| 23.64724 | 25.13907 | 25.42451 | 24.59269 | 3 | 3 | 3 | 7.5 | 7.5 | 7.5 | 58.375 | 46.72 | 209630000 | 14 | Pre-mRNA 3-end-processing factor FIP1 | FIP1L1 |
| 25.09812 | 24.85709 | 25.42233 | 25.64111 | 8 | 8 | 8 | 14.9 | 14.9 | 14.9 | 52.949 | 51.437 | 1428900000 | 27 | Protein transport protein Sec61 subunit alpha isoform 1;Protein transport protein Sec61 subunit alpha isoform 2 | SEC61A1;SEC61A2 |
| 25.68078 | 25.03246 | 25.41613 | 25.64804 | 25 | 25 | 22 | 36.6 | 36.6 | 31.9 | 85.595 | 323.31 | 2645900000 | 55 | ATP-dependent 6-phosphofructokinase, platelet type | PFKP |
| 24.79774 | 25.64743 | 25.38913 | 25.14814 | 24 | 24 | 24 | 36.2 | 36.2 | 36.2 | 68.047 | 187.38 | 1171000000 | 61 | Lysine--tRNA ligase | KARS |
| 23.78487 | 25.79139 | 25.38686 | 25.76377 | 26 | 16 | 14 | 37 | 23 | 21.7 | 69.412 | 161.91 | 1552200000 | 40 | Ezrin | EZR |
| 23.19093 | 24.88758 | 25.37981 | 24.45883 | 10 | 10 | 10 | 29.2 | 29.2 | 29.2 | 43.476 | 163.03 | 568650000 | 38 | Polymerase I and transcript release factor | PTRF |
| 23.37769 | 25.5466 | 25.36706 | 25.63603 | 48 | 48 | 44 | 26 | 26 | 24.2 | 269.76 | 323.31 | 1411500000 | 80 | Talin-1 | TLN1 |
| 31.13339 | 22.5186 | 25.36593 | 28.34384 | 7 | 7 | 4 | 50 | 50 | 38.2 | 15.388 | 49.086 | 7681400000 | 38 | Histone H3.2;Histone H3.1t;Histone H3.3;Histone H3.1;Histone H3.3C;Histone H3 | HIST2H3A;HIST3H3;H3F3A;HIST1H3A;H3F3C |
| 26.00928 | 24.64933 | 25.36433 | 25.07288 | 18 | 18 | 5 | 35.3 | 35.3 | 10.9 | 66.217 | 248.59 | 1953300000 | 41 | ATPase family AAA domain-containing protein 3A | ATAD3A |
| 22.86756 | 24.97334 | 25.36353 | 20.47971 | 13 | 13 | 13 | 42.2 | 42.2 | 42.2 | 35.936 | 198.71 | 650480000 | 25 | Annexin A5;Annexin | ANXA5 |
| 23.91616 | 24.73609 | 25.36072 | 25.66278 | 15 | 15 | 15 | 68.8 | 68.8 | 68.8 | 26.788 | 242.21 | 777640000 | 38 | Hepatoma-derived growth factor | HDGF |
| 24.70023 | 25.05428 | 25.34868 | 26.11355 | 18 | 18 | 17 | 20.3 | 20.3 | 19.4 | 119.52 | 182.97 | 1740000000 | 46 | Importin-7 | IPO7 |
| 22.85787 | 26.84302 | 25.30921 | 24.78372 | 5 | 5 | 5 | 35.9 | 35.9 | 35.9 | 15.197 | 34.966 | 298920000 | 13 | 40S ribosomal protein S24 | RPS24 |
| 26.90575 | 26.95316 | 25.2856 | 26.00612 | 24 | 24 | 24 | 26.6 | 26.6 | 26.6 | 117.36 | 183.77 | 1912400000 | 43 | Probable ATP-dependent RNA helicase DDX46 | DDX46 |
| 24.99998 | 24.4637 | 25.28429 | 25.64557 | 5 | 5 | 5 | 24.6 | 24.6 | 24.6 | 37.144 | 103.95 | 1190800000 | 25 | Reticulon;Reticulon-4 | RTN4 |
| 25.25958 | 25.12459 | 25.2759 | 25.89851 | 17 | 17 | 17 | 23 | 23 | 23 | 99.045 | 193.21 | 2029800000 | 46 | Coatomer subunit beta | COPB2 |
| 17.37213 | 24.31147 | 25.26864 | 23.5087 | 3 | 3 | 3 | 2.2 | 2.2 | 2.2 | 152.3 | 28.763 | 180920000 | 11 | Treacle protein | TCOF1 |
| 24.27533 | 25.69386 | 25.25933 | 25.17429 | 58 | 58 | 58 | 28.6 | 28.6 | 28.6 | 273.6 | 323.31 | 2810300000 | 106 | Pre-mRNA-processing-splicing factor 8 | PRPF8 |
| 22.60479 | 23.98198 | 25.25562 | 23.73645 | 3 | 3 | 3 | 4 | 4 | 4 | 103.58 | 20.241 | 101130000 | 10 | Transcription initiation factor TFIID subunit 3 | TAF3 |
| 26.03446 | 25.39486 | 25.25328 | 23.68164 | 25 | 25 | 25 | 42.9 | 42.9 | 42.9 | 80.472 | 323.31 | 2800500000 | 53 | Methylcrotonoyl-CoA carboxylase subunit alpha, mitochondrial | MCCC1 |
| 23.54321 | 25.47457 | 25.24934 | 25.20788 | 5 | 5 | 5 | 39.3 | 39.3 | 39.3 | 15.55 | 41.802 | 235400000 | 16 | 40S ribosomal protein S17-like;40S ribosomal protein S17 | RPS17L;RPS17 |
| 25.50606 | 26.86597 | 25.2471 | 22.9786 | 12 | 12 | 12 | 36.9 | 36.9 | 36.9 | 43.062 | 122.71 | 1565600000 | 30 | Interleukin enhancer-binding factor 2 | ILF2 |
| 25.07161 | 24.65075 | 25.24637 | 25.36182 | 14 | 14 | 11 | 46.9 | 46.9 | 39 | 44.323 | 133.04 | 1235500000 | 37 | 26S protease regulatory subunit 6A | PSMC3 |
| 24.74057 | 24.96367 | 25.24016 | 25.27441 | 17 | 17 | 17 | 17.4 | 17.4 | 17.4 | 126.97 | 216.99 | 1576100000 | 36 | DNA damage-binding protein 1 | DDB1 |
| 24.66666 | 24.3841 | 25.23878 | 24.62934 | 17 | 17 | 17 | 36 | 36 | 36 | 59.143 | 128.04 | 785620000 | 43 | Tyrosine--tRNA ligase, cytoplasmic;Tyrosine--tRNA ligase, cytoplasmic, N-terminally processed | YARS |
| 24.89393 | 25.07602 | 25.22498 | 25.08428 | 17 | 17 | 17 | 40.9 | 40.9 | 40.9 | 57.136 | 203.75 | 1062700000 | 37 | Aspartate--tRNA ligase, cytoplasmic | DARS |
| 22.99759 | 22.79509 | 25.2179 | 25.22358 | 179 | 2 | 2 | 79.2 | 0.4 | 0.4 | 257.24 | 12.172 | 1115600000 | 13 | Acetyl-CoA carboxylase 1;Biotin carboxylase | ACACA |
| 24.56238 | 25.48505 | 25.21026 | 26.11981 | 8 | 8 | 8 | 22.9 | 22.9 | 22.9 | 46.4 | 98.296 | 1089300000 | 41 | Basigin | BSG |
| 25.42288 | 25.14818 | 25.20415 | 24.8791 | 24 | 24 | 24 | 35.1 | 35.1 | 35.1 | 90.98 | 323.31 | 1971200000 | 51 | DNA replication licensing factor MCM3 | MCM3 |
| 25.49268 | 25.15815 | 25.18752 | 25.04089 | 27 | 26 | 26 | 37.1 | 36.1 | 36.1 | 105.38 | 323.31 | 2190900000 | 49 | 116 kDa U5 small nuclear ribonucleoprotein component | EFTUD2 |
| 24.43371 | 24.97299 | 25.18552 | 25.11716 | 9 | 9 | 9 | 26.9 | 26.9 | 26.9 | 35.611 | 130.11 | 919820000 | 26 | Eukaryotic translation initiation factor 3 subunit G | EIF3G |
| 24.38502 | 24.77223 | 25.17991 | 25.38768 | 25 | 25 | 25 | 29.9 | 29.9 | 29.9 | 123.38 | 258.37 | 1476400000 | 48 | Exportin-1 | XPO1 |
| 25.68024 | 26.27899 | 25.17562 | 25.14853 | 6 | 6 | 6 | 35.2 | 35.2 | 35.2 | 16.06 | 42.442 | 341120000 | 17 | 40S ribosomal protein S19 | RPS19 |
| 24.5559 | 25.18952 | 25.16227 | 25.6113 | 11 | 11 | 10 | 25.4 | 25.4 | 23.4 | 50.431 | 88.566 | 1105200000 | 36 | Citrate synthase;Citrate synthase, mitochondrial | CS |
| 26.82043 | 24.68785 | 25.15711 | 25.30226 | 9 | 9 | 8 | 54.8 | 54.8 | 54.8 | 14.203 | 62.592 | 434910000 | 24 | Serine/arginine-rich splicing factor 3 | SRSF3 |
| 25.04618 | 26.1025 | 25.15348 | 24.84656 | 7 | 7 | 7 | 57.4 | 57.4 | 57.4 | 14.57 | 51.212 | 272760000 | 23 | Signal recognition particle 14 kDa protein | SRP14 |
| 25.01873 | 25.15333 | 25.14915 | 25.70901 | 17 | 17 | 17 | 24.8 | 24.8 | 24.8 | 85.134 | 238.73 | 1844100000 | 41 | Eukaryotic translation initiation factor 3 subunit B | EIF3B |
| 23.00414 | 23.68271 | 25.14748 | 24.0974 | 8 | 8 | 8 | 20.6 | 20.6 | 20.6 | 59.151 | 75.969 | 510330000 | 20 | Lysophosphatidylcholine acyltransferase 1 | LPCAT1 |
| 24.46569 | 24.25523 | 25.14453 | 24.76966 | 6 | 6 | 6 | 31.5 | 31.5 | 31.5 | 26.489 | 43.959 | 133360000 | 18 | Proteasome subunit beta type-1 | PSMB1 |
| 24.26005 | 24.57404 | 25.14406 | 24.41893 | 18 | 18 | 18 | 23.6 | 23.6 | 23.6 | 110.61 | 147.27 | 1002900000 | 32 | 2-oxoglutarate dehydrogenase, mitochondrial | OGDH |
| 23.1652 | 24.9009 | 25.10893 | 25.89728 | 7 | 7 | 7 | 19.3 | 19.3 | 19.3 | 40.532 | 62.146 | 567280000 | 15 | Farnesyl pyrophosphate synthase | FDPS |
| 28.2936 | 23.89846 | 25.10641 | 23.54569 | 83 | 83 | 8 | 43.9 | 43.9 | 5.1 | 231.24 | 323.31 | 1.4872E+10 | 187 | Unconventional myosin-XVIIIa | MYO18A |
| 23.92895 | 24.8075 | 25.09623 | 23.83499 | 4 | 4 | 4 | 8.2 | 8.2 | 8.2 | 53.944 | 129 | 503710000 | 18 | Monocarboxylate transporter 1 | SLC16A1 |
| 25.77077 | 24.84067 | 25.09499 | 25.39486 | 16 | 16 | 16 | 31 | 31 | 31 | 77.328 | 323.31 | 2283600000 | 41 | Protein-glutamine gamma-glutamyltransferase 2 | TGM2 |
| 27.47127 | 25.21745 | 25.09398 | 24.54769 | 23 | 23 | 15 | 47.8 | 47.8 | 30.5 | 75.491 | 323.31 | 4708900000 | 72 | Lamina-associated polypeptide 2, isoform alpha;Thymopoietin;Thymopentin | TMPO |
| 23.27056 | 24.57415 | 25.09075 | 25.32062 | 11 | 11 | 11 | 31 | 31 | 31 | 44.468 | 142.85 | 635420000 | 22 | Hsp90 co-chaperone Cdc37;Hsp90 co-chaperone Cdc37, N-terminally processed | CDC37 |
| 22.77354 | 24.07366 | 25.07769 | 22.68887 | 11 | 11 | 10 | 36.2 | 36.2 | 34 | 42.621 | 99.924 | 436990000 | 24 | Serpin B6 | SERPINB6 |
| 17.81607 | 16.81439 | 25.0719 | 19.08919 | 9 | 1 | 1 | 36.1 | 5.4 | 5.4 | 32.103 | 8.3246 | 120880000 | 1 | Protein SET | SET |
| 26.16697 | 25.52085 | 25.0712 | 24.87657 | 17 | 17 | 16 | 31.2 | 31.2 | 28.8 | 69.491 | 155.84 | 1913700000 | 55 | RNA-binding protein 14 | RBM14 |
| 23.9228 | 24.60462 | 25.07092 | 25.17972 | 12 | 12 | 12 | 40.5 | 40.5 | 40.5 | 38.274 | 106.94 | 664730000 | 32 | Activator of 90 kDa heat shock protein ATPase homolog 1 | AHSA1 |
| 25.24452 | 24.9832 | 25.06238 | 19.58731 | 7 | 7 | 7 | 21.3 | 21.3 | 21.3 | 42.008 | 64.479 | 565310000 | 16 | Uncharacterized protein C9orf114 | C9orf114 |
| 22.88564 | 24.55245 | 25.05949 | 25.28972 | 11 | 11 | 11 | 33.8 | 33.8 | 33.8 | 40.422 | 74.219 | 392870000 | 20 | Phosphoserine aminotransferase | PSAT1 |
| 24.35448 | 24.33265 | 25.05821 | 24.23936 | 10 | 10 | 10 | 40.8 | 40.8 | 40.8 | 36.94 | 136.18 | 706190000 | 23 | Septin-2 | 02. Sep |
| 24.57525 | 23.74417 | 25.0493 | 23.98285 | 8 | 8 | 8 | 25.8 | 25.8 | 25.8 | 44.739 | 131.49 | 849950000 | 25 | TAR DNA-binding protein 43 | TARDBP |
| 25.09499 | 25.16553 | 25.0466 | 25.59218 | 19 | 19 | 19 | 29.7 | 29.7 | 29.7 | 97.717 | 236.26 | 1968800000 | 41 | Coatomer subunit gamma-1 | COPG1 |
| 23.59195 | 24.28926 | 25.04289 | 25.25714 | 10 | 10 | 10 | 33.5 | 33.5 | 33.5 | 39.93 | 69.248 | 429470000 | 22 | Eukaryotic translation initiation factor 3 subunit H | EIF3H |
| 25.46435 | 24.79793 | 25.03993 | 25.50273 | 11 | 11 | 11 | 37.2 | 37.2 | 37.2 | 36.501 | 140.02 | 1240500000 | 36 | Eukaryotic translation initiation factor 3 subunit I | EIF3I |
| 24.73133 | 24.56517 | 25.03511 | 25.02496 | 16 | 16 | 16 | 41.2 | 41.2 | 41.2 | 52.904 | 140.37 | 1003100000 | 34 | 26S proteasome non-ATPase regulatory subunit 12 | PSMD12 |
| 27.50907 | 25.28521 | 25.02813 | 24.8659 | 22 | 22 | 22 | 36.5 | 36.5 | 36.5 | 58.848 | 323.31 | 4381100000 | 61 | Splicing factor 3A subunit 3 | SF3A3 |
| 25.20848 | 23.93319 | 25.02365 | 24.7681 | 7 | 7 | 7 | 30.4 | 30.4 | 30.4 | 28.48 | 56.753 | 164080000 | 33 | Proteasome subunit beta type-5 | PSMB5 |
| 25.06538 | 24.71555 | 25.02149 | 25.66939 | 18 | 18 | 18 | 41.6 | 41.6 | 41.6 | 64.615 | 214.17 | 1015500000 | 37 | Bifunctional purine biosynthesis protein PURH;Phosphoribosylaminoimidazolecarboxamide formyltransferase;IMP cyclohydrolase | ATIC |
| 23.06248 | 23.80282 | 25.01861 | 23.30522 | 8 | 8 | 8 | 18.6 | 18.6 | 18.6 | 59.755 | 67.172 | 272940000 | 19 | Catalase | CAT |
| 24.9167 | 25.85115 | 25.00979 | 24.77012 | 5 | 5 | 5 | 24.2 | 24.2 | 24.2 | 18.898 | 47.133 | 230130000 | 17 | 40S ribosomal protein S10;Putative 40S ribosomal protein S10-like | RPS10;RPS10P5 |
| 26.19336 | 23.576 | 25.00868 | 23.4501 | 45 | 45 | 45 | 29.2 | 29.2 | 29.2 | 190.68 | 323.31 | 1564900000 | 58 | Phosphatidylinositol 4-phosphate 3-kinase C2 domain-containing subunit alpha | PIK3C2A |
| 22.11709 | 24.16216 | 25.00509 | 24.74371 | 5 | 5 | 5 | 41.1 | 41.1 | 41.1 | 16.214 | 34.523 | 317230000 | 34 | Cystatin-S;Cystatin-SA | CST4;CST2 |
| 23.90463 | 24.15653 | 24.99568 | 24.50924 | 10 | 10 | 10 | 23.8 | 23.8 | 23.8 | 49.03 | 80.459 | 565360000 | 27 | Eukaryotic peptide chain release factor subunit 1 | ETF1 |
| 24.77776 | 25.55447 | 24.98772 | 25.91059 | 26 | 23 | 21 | 37.3 | 33.5 | 31 | 92.115 | 220.27 | 1976300000 | 51 | Heat shock protein 105 kDa | HSPH1 |
| 24.4982 | 23.38916 | 24.98198 | 25.28429 | 6 | 6 | 6 | 26 | 26 | 26 | 25.387 | 68.662 | 134710000 | 16 | N-terminal Xaa-Pro-Lys N-methyltransferase 1;N-terminal Xaa-Pro-Lys N-methyltransferase 1, N-terminally processed | NTMT1 |
| 29.7964 | 23.89486 | 24.97189 | 22.24673 | 47 | 47 | 1 | 56 | 56 | 1.8 | 85.225 | 323.31 | 2.2928E+10 | 99 | LIM domain and actin-binding protein 1 | LIMA1;TRMT1 |
| 24.7455 | 24.58669 | 24.97005 | 24.96759 | 14 | 14 | 14 | 28.7 | 28.7 | 28.7 | 40.188 | 93.685 | 753170000 | 39 | Pyruvate dehydrogenase E1 component subunit alpha, somatic form, mitochondrial | PDHA1 |
| 24.4829 | 24.16039 | 24.96468 | 24.67874 | 11 | 11 | 11 | 31.9 | 31.9 | 31.9 | 42.945 | 91.035 | 795670000 | 27 | 26S proteasome non-ATPase regulatory subunit 13 | PSMD13 |
| 26.58592 | 22.74999 | 24.96358 | 23.05447 | 10 | 10 | 10 | 41.2 | 41.2 | 41.2 | 23.772 | 67.398 | 292260000 | 17 | BAG family molecular chaperone regulator 2 | BAG2 |
| 23.63843 | 24.31852 | 24.95708 | 25.11886 | 8 | 8 | 8 | 18.3 | 18.3 | 18.3 | 49.469 | 59.036 | 394910000 | 19 | Monocarboxylate transporter 4 | SLC16A3 |
| 26.5429 | 28.98112 | 24.9526 | 23.35421 | 6 | 6 | 6 | 21.9 | 21.9 | 21.9 | 23.466 | 83.605 | 1382500000 | 48 | 60S ribosomal protein L19 | RPL19 |
| 25.70496 | 24.91036 | 24.95047 | 24.64014 | 26 | 26 | 26 | 44.3 | 44.3 | 44.3 | 82.729 | 323.31 | 2262400000 | 44 | MICOS complex subunit MIC60 | IMMT |
| 25.02644 | 24.73449 | 24.94432 | 25.0916 | 9 | 9 | 9 | 31.9 | 31.9 | 31.9 | 36.954 | 156.26 | 1013100000 | 22 | Mitotic checkpoint protein BUB3 | BUB3 |
| 24.96996 | 24.73939 | 24.94396 | 25.3432 | 14 | 14 | 6 | 34 | 34 | 16.4 | 49.129 | 164.57 | 1267200000 | 37 | ATP-dependent RNA helicase DDX39A | DDX39A;DDX39 |
| 24.9885 | 24.85029 | 24.92909 | 24.64427 | 19 | 19 | 19 | 22 | 22 | 22 | 106.92 | 177.85 | 1510400000 | 37 | Pre-mRNA-processing factor 6 | PRPF6 |
| 24.6011 | 24.24278 | 24.92339 | 25.18673 | 7 | 7 | 7 | 46.3 | 46.3 | 46.3 | 16.118 | 54.953 | 249940000 | 19 | Eukaryotic translation initiation factor 5A-1;Eukaryotic translation initiation factor 5A-1-like | EIF5A;EIF5AL1 |
| 25.15673 | 23.69636 | 24.90601 | 24.52648 | 6 | 6 | 6 | 40.7 | 40.7 | 40.7 | 13.527 | 43.547 | 212340000 | 18 | Small nuclear ribonucleoprotein Sm D2 | SNRPD2 |
| 24.22692 | 24.55022 | 24.88721 | 24.59172 | 10 | 10 | 10 | 39.4 | 39.4 | 39.4 | 37.709 | 90.656 | 470820000 | 27 | Protein arginine N-methyltransferase 1 | PRMT1 |
| 28.42926 | 23.73686 | 24.87755 | 23.34422 | 20 | 20 | 20 | 63.9 | 63.9 | 63.9 | 39.594 | 323.31 | 7965500000 | 52 | Tropomodulin-3 | TMOD3 |
| 23.74581 | 24.31354 | 24.87065 | 25.39398 | 8 | 8 | 8 | 24.2 | 24.2 | 24.2 | 40.369 | 81.947 | 660110000 | 20 | Thioredoxin domain-containing protein 5 | TXNDC5 |
| 25.07333 | 25.14511 | 24.84656 | 24.64147 | 5 | 5 | 4 | 51.5 | 51.5 | 41.9 | 15.016 | 172.12 | 941100000 | 26 | Nascent polypeptide-associated complex subunit alpha;Nascent polypeptide-associated complex subunit alpha, muscle-specific form | NACA |
| 22.57817 | 23.35864 | 24.8436 | 24.48984 | 6 | 6 | 6 | 13.7 | 13.7 | 13.7 | 56.216 | 71.532 | 345150000 | 15 | Adenylosuccinate lyase | ADSL |
| 24.85875 | 26.43917 | 24.84259 | 24.17058 | 16 | 16 | 16 | 32.2 | 32.2 | 32.2 | 60.54 | 170.98 | 1295300000 | 42 | Guanine nucleotide-binding protein-like 3 | GNL3 |
| 25.73451 | 25.19835 | 24.84149 | 25.03154 | 23 | 14 | 14 | 33.5 | 21.8 | 21.8 | 80.439 | 197.96 | 1754700000 | 51 | Probable ATP-dependent RNA helicase DDX17 | DDX17 |
| 24.64246 | 24.90205 | 24.83783 | 25.81499 | 21 | 21 | 19 | 40.2 | 40.2 | 36.5 | 70.81 | 168.4 | 1193700000 | 51 | Plastin-3 | PLS3 |
| 24.69959 | 24.51509 | 24.83045 | 25.08079 | 21 | 21 | 21 | 29.5 | 29.5 | 29.5 | 87.088 | 212.54 | 1574000000 | 46 | Delta-1-pyrroline-5-carboxylate synthase;Glutamate 5-kinase;Gamma-glutamyl phosphate reductase | ALDH18A1 |
| 22.81591 | 24.06809 | 24.83007 | 24.17972 | 10 | 10 | 10 | 37.5 | 37.5 | 37.5 | 41.924 | 108.9 | 315440000 | 21 | 3-ketoacyl-CoA thiolase, mitochondrial | ACAA2 |
| 23.12417 | 24.41209 | 24.82847 | 23.17749 | 8 | 5 | 0 | 26.5 | 19.3 | 0 | 40.332 | 107.01 | 359480000 | 14 | HLA class I histocompatibility antigen, B-42 alpha chain;HLA class I histocompatibility antigen, B-8 alpha chain;HLA class I histocompatibility antigen, B-14 alpha chain;HLA class I histocompatibility antigen, B-37 alpha chain;HLA class I histocompatibility antigen, B-7 alpha chain;HLA class I histocompatibility antigen, B-40 alpha chain;HLA class I histocompatibility antigen, B-81 alpha chain;HLA class I histocompatibility antigen, B-48 alpha chain;HLA class I histocompatibility antigen, B-27 alpha chain;HLA class I histocompatibility antigen, B-50 alpha chain;HLA class I histocompatibility antigen, B-49 alpha chain;HLA class I histocompatibility antigen, B-45 alpha chain | HLA-B |
| 23.74817 | 24.94342 | 24.8209 | 25.57681 | 23 | 23 | 23 | 20.4 | 20.4 | 20.4 | 136.37 | 237.99 | 1075100000 | 44 | Cullin-associated NEDD8-dissociated protein 1 | CAND1 |
| 24.62684 | 24.65398 | 24.82007 | 25.43797 | 12 | 12 | 12 | 23.7 | 23.7 | 23.7 | 67.299 | 106.82 | 818960000 | 28 | Succinate dehydrogenase [ubiquinone] flavoprotein subunit, mitochondrial | SDHA |
| 24.49845 | 24.74191 | 24.81948 | 25.18703 | 24 | 24 | 19 | 32.3 | 32.3 | 26.7 | 109.68 | 312.8 | 1386700000 | 49 | Kinesin-1 heavy chain | KIF5B |
| 23.99507 | 24.82512 | 24.81763 | 25.05759 | 15 | 13 | 13 | 27.7 | 26.5 | 26.5 | 72.896 | 171.85 | 1156500000 | 29 | Far upstream element-binding protein 2 | KHSRP |
| 24.92972 | 24.3002 | 24.81421 | 19.68918 | 9 | 9 | 9 | 26.5 | 26.5 | 26.5 | 42.502 | 86.358 | 629550000 | 17 | Eukaryotic translation initiation factor 3 subunit M | EIF3M |
| 25.15518 | 25.08099 | 24.81029 | 25.09245 | 10 | 10 | 9 | 26.3 | 26.3 | 23.6 | 38.418 | 72.059 | 1046800000 | 32 | Serine/arginine-rich splicing factor 6 | SRSF6 |
| 23.52181 | 25.47633 | 24.80897 | 24.59889 | 10 | 10 | 10 | 39.1 | 39.1 | 39.1 | 29.172 | 81.352 | 654970000 | 38 | Protein FRG1;Protein FRG1B | FRG1;FRG1B |
| 27.92105 | 24.62416 | 24.80272 | 24.02471 | 54 | 54 | 53 | 59.2 | 59.2 | 58.3 | 118.1 | 323.31 | 5284500000 | 86 | Myosin phosphatase Rho-interacting protein | MPRIP |
| 24.58124 | 24.39139 | 24.79223 | 25.05412 | 18 | 18 | 18 | 32.1 | 32.1 | 32.1 | 75.209 | 187.59 | 729750000 | 36 | Very long-chain specific acyl-CoA dehydrogenase, mitochondrial | ACADVL |
| 24.37511 | 24.17387 | 24.78811 | 24.56895 | 16 | 16 | 16 | 41.2 | 41.2 | 41.2 | 47.463 | 187.39 | 854560000 | 37 | 26S proteasome non-ATPase regulatory subunit 11 | PSMD11 |
| 25.34848 | 27.69617 | 24.78586 | 23.67347 | 8 | 8 | 8 | 48.9 | 48.9 | 48.9 | 15.616 | 58.283 | 511750000 | 24 | 60S ribosomal protein L32 | RPL32 |
| 27.35951 | 24.73733 | 24.78427 | 24.86495 | 21 | 21 | 21 | 44.9 | 44.9 | 44.9 | 66.193 | 323.31 | 3674700000 | 55 | WD repeat-containing protein 1 | WDR1 |
| 23.5316 | 23.83306 | 24.7747 | 23.60919 | 9 | 9 | 9 | 27 | 27 | 27 | 34.735 | 52.421 | 310640000 | 28 | Arginase-1 | ARG1 |
| 27.33577 | 25.09772 | 24.76946 | 23.33715 | 24 | 24 | 24 | 40.7 | 40.7 | 40.7 | 86.121 | 323.31 | 5107500000 | 55 | Heterogeneous nuclear ribonucleoprotein U-like protein 1 | HNRNPUL1 |
| 24.19355 | 24.32704 | 24.76512 | 24.87526 | 8 | 8 | 8 | 46.5 | 46.5 | 46.5 | 20.228 | 108.45 | 946450000 | 27 | CD44 antigen | CD44 |
| 24.58858 | 24.51833 | 24.7637 | 25.07708 | 10 | 5 | 5 | 26.7 | 16.3 | 16.3 | 46.938 | 78.409 | 1197600000 | 21 | Histone-binding protein RBBP7 | RBBP7 |
| 23.31879 | 26.13422 | 24.75037 | 25.37948 | 11 | 11 | 1 | 38.1 | 38.1 | 3.6 | 40.908 | 156.99 | 865320000 | 23 | HLA class I histocompatibility antigen, A-68 alpha chain;HLA class I histocompatibility antigen, A-33 alpha chain;HLA class I histocompatibility antigen, A-74 alpha chain;HLA class I histocompatibility antigen, A-31 alpha chain | HLA-A |
| 24.03859 | 25.00002 | 24.72557 | 24.95983 | 12 | 12 | 12 | 46.6 | 46.6 | 46.6 | 39.157 | 122.93 | 612480000 | 28 | Replication factor C subunit 2 | RFC2 |
| 24.48198 | 24.31465 | 24.72067 | 25.23045 | 11 | 11 | 8 | 33.3 | 33.3 | 24.2 | 37.839 | 84.194 | 960060000 | 29 | Erlin-2 | ERLIN2 |
| 26.18499 | 24.09281 | 24.71743 | 24.38331 | 10 | 10 | 5 | 32.8 | 32.8 | 17.8 | 40.532 | 109.78 | 1864800000 | 30 | Guanine nucleotide-binding protein G(k) subunit alpha | GNAI3 |
| 23.95136 | 24.44436 | 24.70773 | 22.48112 | 19 | 14 | 14 | 26.4 | 19.5 | 19.5 | 97.658 | 137.12 | 1003900000 | 26 | Heat shock 70 kDa protein 4L | HSPA4L |
| 23.55028 | 25.85546 | 24.70345 | 25.18495 | 8 | 6 | 2 | 40.6 | 35.3 | 17.4 | 24.366 | 60.243 | 837800000 | 22 | Nucleosome assembly protein 1-like 1 | NAP1L1 |
| 24.17585 | 24.86372 | 24.68918 | 25.05304 | 12 | 12 | 12 | 29.7 | 29.7 | 29.7 | 55.366 | 109.96 | 712230000 | 20 | Alpha-aminoadipic semialdehyde dehydrogenase | ALDH7A1 |
| 24.6848 | 24.5625 | 24.6871 | 24.82425 | 20 | 20 | 20 | 25.4 | 25.4 | 25.4 | 103.64 | 235.52 | 1376700000 | 40 | Eukaryotic translation initiation factor 3 subunit C;Eukaryotic translation initiation factor 3 subunit C-like protein | EIF3C;EIF3CL |
| 24.58428 | 24.05697 | 24.6826 | 24.40886 | 14 | 14 | 14 | 33.4 | 33.4 | 33.4 | 45.531 | 166.78 | 842300000 | 28 | 26S proteasome non-ATPase regulatory subunit 6 | PSMD6 |
| 23.95225 | 24.34807 | 24.6804 | 25.00325 | 12 | 12 | 12 | 19.7 | 19.7 | 19.7 | 86.238 | 132.64 | 976630000 | 27 | Carnitine O-palmitoyltransferase 1, liver isoform | CPT1A |
| 25.3057 | 25.23458 | 24.67492 | 25.56961 | 44 | 44 | 44 | 33.8 | 33.8 | 33.8 | 153.89 | 323.31 | 1208200000 | 68 | DNA repair protein RAD50 | RAD50 |
| 21.29109 | 23.83345 | 24.67266 | 24.60467 | 11 | 11 | 11 | 18.6 | 18.6 | 18.6 | 76.631 | 73.415 | 291850000 | 30 | Protein-glutamine gamma-glutamyltransferase E;Protein-glutamine gamma-glutamyltransferase E 50 kDa catalytic chain;Protein-glutamine gamma-glutamyltransferase E 27 kDa non-catalytic chain | TGM3 |
| 23.296 | 24.54239 | 24.67109 | 23.86457 | 5 | 5 | 5 | 17.1 | 17.1 | 17.1 | 31.576 | 37.226 | 145670000 | 12 | RNA-binding motif protein, X-linked 2 | RBMX2 |
| 26.17735 | 26.53148 | 24.65862 | 25.36753 | 37 | 37 | 37 | 36 | 36 | 36 | 140.46 | 323.31 | 1811500000 | 78 | Valine--tRNA ligase | VARS |
| 24.68833 | 24.40282 | 24.65464 | 24.45513 | 16 | 16 | 16 | 29.8 | 29.8 | 29.8 | 70.901 | 230.26 | 778490000 | 31 | Eukaryotic translation initiation factor 3 subunit L | EIF3L |
| 17.26807 | 24.32608 | 24.65289 | 24.27448 | 4 | 2 | 2 | 22.4 | 13 | 13 | 18.214 | 13.344 | 91324000 | 8 | Peroxiredoxin-4 | PRDX4 |
| 24.20523 | 23.91287 | 24.63062 | 24.76517 | 8 | 8 | 8 | 22.3 | 22.3 | 22.3 | 42.426 | 59.173 | 534950000 | 17 | Medium-chain specific acyl-CoA dehydrogenase, mitochondrial | ACADM |
| 24.4467 | 25.16951 | 24.63018 | 24.92034 | 10 | 10 | 10 | 31.6 | 31.6 | 31.6 | 41.824 | 107.17 | 770690000 | 27 | Lupus La protein | SSB |
| 25.12959 | 25.3683 | 24.625 | 25.29063 | 21 | 21 | 21 | 37 | 37 | 37 | 86.47 | 323.31 | 1943900000 | 47 | tRNA (cytosine(34)-C(5))-methyltransferase | NSUN2 |
| 24.18782 | 23.89929 | 24.62271 | 24.86863 | 5 | 5 | 5 | 32.8 | 32.8 | 32.8 | 19.48 | 38.339 | 130100000 | 12 | Glutathione S-transferase P | GSTP1 |
| 25.07349 | 24.92583 | 24.6193 | 24.64872 | 11 | 9 | 9 | 27.8 | 23.6 | 23.6 | 50.817 | 97.196 | 914270000 | 18 | Ras GTPase-activating protein-binding protein 2 | G3BP2 |
| 25.23994 | 24.23074 | 24.61217 | 25.49179 | 21 | 21 | 21 | 32.7 | 32.7 | 32.7 | 81.307 | 190.59 | 1814700000 | 36 | DNA replication licensing factor MCM7 | MCM7 |
| 24.4222 | 24.63882 | 24.60727 | 24.23557 | 10 | 10 | 10 | 32.2 | 32.2 | 32.2 | 44.76 | 99.695 | 948610000 | 25 | Actin-related protein 2 | ACTR2 |
| 23.43148 | 24.052 | 24.59929 | 24.73319 | 9 | 9 | 9 | 18 | 18 | 18 | 64.244 | 61.135 | 655650000 | 33 | Peptidyl-prolyl cis-trans isomerase FKBP10 | FKBP10 |
| 24.96534 | 25.5228 | 24.59343 | 24.52385 | 12 | 12 | 12 | 43.2 | 43.2 | 43.2 | 38.388 | 145.01 | 984390000 | 39 | Eukaryotic translation initiation factor 2 subunit 2 | EIF2S2 |
| 23.01018 | 23.9049 | 24.59047 | 22.23748 | 6 | 6 | 6 | 21.1 | 21.1 | 21.1 | 41.92 | 60.97 | 324030000 | 17 | Synaptic vesicle membrane protein VAT-1 homolog | VAT1 |
| 25.29204 | 25.39149 | 24.5852 | 24.29446 | 19 | 14 | 14 | 32.5 | 25.4 | 25.4 | 70.942 | 178.8 | 1634400000 | 34 | Heterogeneous nuclear ribonucleoprotein R | HNRNPR |
| 22.19913 | 23.69381 | 24.57623 | 24.70872 | 5 | 5 | 5 | 34.6 | 34.6 | 34.6 | 12.139 | 30.308 | 100780000 | 11 | Protein FAM32A | FAM32A |
| 23.54451 | 24.30236 | 24.57589 | 24.69088 | 15 | 15 | 15 | 20.7 | 20.7 | 20.7 | 83.165 | 120.31 | 680740000 | 23 | Glycine--tRNA ligase | GARS |
| 24.78182 | 24.45876 | 24.57103 | 24.62979 | 13 | 13 | 13 | 34 | 34 | 34 | 48.55 | 114.95 | 938430000 | 41 | Pachytene checkpoint protein 2 homolog | TRIP13 |
| 23.8148 | 24.95863 | 24.56773 | 25.0678 | 13 | 13 | 13 | 20.9 | 20.9 | 20.9 | 83.267 | 187.92 | 1126700000 | 32 | Aspartyl/asparaginyl beta-hydroxylase | ASPH |
| 23.40367 | 23.99378 | 24.55947 | 24.71492 | 9 | 9 | 9 | 42.2 | 42.2 | 42.2 | 29.062 | 74.244 | 448150000 | 26 | Eukaryotic translation initiation factor 3 subunit J | EIF3J |
| 24.98207 | 24.912 | 24.55549 | 24.83277 | 6 | 6 | 6 | 35.5 | 35.5 | 35.5 | 22.842 | 41.214 | 175750000 | 22 | Proteasome subunit alpha type;Proteasome subunit alpha type-6 | PSMA6 |
| 24.60637 | 24.60428 | 24.54363 | 25.10081 | 14 | 14 | 14 | 38.4 | 38.4 | 38.4 | 49.184 | 204.57 | 891410000 | 31 | 26S protease regulatory subunit 4 | PSMC1 |
| 23.8187 | 24.40373 | 24.53919 | 24.82343 | 10 | 10 | 10 | 17.8 | 17.8 | 17.8 | 85.237 | 121.84 | 638530000 | 30 | Nuclear autoantigenic sperm protein | NASP |
| 23.74509 | 24.07896 | 24.53706 | 24.37126 | 13 | 13 | 13 | 39.3 | 39.3 | 39.3 | 43.507 | 108.66 | 562990000 | 30 | 26S protease regulatory subunit 6B | PSMC4 |
| 26.77329 | 24.34476 | 24.52576 | 23.21016 | 18 | 18 | 18 | 72.6 | 72.6 | 72.6 | 35.076 | 248.64 | 2529100000 | 47 | Guanine nucleotide-binding protein subunit beta-2-like 1;Guanine nucleotide-binding protein subunit beta-2-like 1, N-terminally processed | GNB2L1 |
| 24.45318 | 24.75908 | 24.52474 | 24.24402 | 13 | 13 | 13 | 19.2 | 19.2 | 19.2 | 101.31 | 219.71 | 1203600000 | 30 | Transportin-1 | TNPO1 |
| 23.68014 | 23.55672 | 24.51767 | 23.28828 | 4 | 4 | 4 | 17 | 17 | 17 | 27.978 | 35.947 | 132070000 | 15 | Transmembrane protein 33 | TMEM33 |
| 25.1553 | 26.33147 | 24.51635 | 24.25443 | 6 | 6 | 3 | 51 | 51 | 26 | 11.477 | 35.38 | 299300000 | 18 | 40S ribosomal protein S15a | RPS15A |
| 23.84211 | 23.28574 | 24.51629 | 25.16492 | 6 | 6 | 6 | 33 | 33 | 33 | 23.545 | 39.237 | 117980000 | 15 | Ras-related protein Rab-2A | RAB2A |
| 25.10229 | 24.70303 | 24.50894 | 25.21471 | 24 | 24 | 24 | 35.6 | 35.6 | 35.6 | 102.26 | 260.9 | 1594700000 | 43 | 26S proteasome non-ATPase regulatory subunit 1 | PSMD1 |
| 24.90812 | 24.6584 | 24.50773 | 24.63356 | 28 | 28 | 28 | 41.6 | 41.6 | 41.6 | 96.022 | 313.54 | 1559200000 | 40 | Programmed cell death 6-interacting protein | PDCD6IP |
| 24.61263 | 25.19261 | 24.50628 | 23.71471 | 14 | 14 | 14 | 30 | 30 | 30 | 57.673 | 97.732 | 696610000 | 33 | H/ACA ribonucleoprotein complex subunit 4 | DKC1 |
| 23.45375 | 24.81997 | 24.50154 | 24.58325 | 7 | 7 | 7 | 11.4 | 11.4 | 11.4 | 90.254 | 55.509 | 260840000 | 18 | U4/U6.U5 tri-snRNP-associated protein 1 | SART1 |
| 24.78706 | 24.08253 | 24.47475 | 24.72702 | 12 | 12 | 12 | 25.4 | 25.4 | 25.4 | 52.22 | 85.408 | 812560000 | 31 | Eukaryotic translation initiation factor 3 subunit E | EIF3E |
| 26.36991 | 25.19321 | 24.47451 | 22.43563 | 11 | 10 | 2 | 34.1 | 32.3 | 7.9 | 39.594 | 231.4 | 1932500000 | 35 | Heterogeneous nuclear ribonucleoprotein A3 | HNRNPA3 |
| 23.14194 | 24.0118 | 24.47141 | 25.20049 | 8 | 8 | 8 | 23.8 | 23.8 | 23.8 | 47.726 | 84.593 | 394590000 | 19 | COP9 signalosome complex subunit 4 | COPS4 |
| 23.77274 | 24.25904 | 24.46588 | 25.38083 | 18 | 18 | 18 | 27.7 | 27.7 | 27.7 | 90.069 | 188.84 | 1259900000 | 30 | Ribonucleoside-diphosphate reductase large subunit | RRM1 |
| 23.40171 | 24.27263 | 24.46083 | 24.59298 | 9 | 9 | 5 | 18.6 | 18.6 | 10.2 | 52.719 | 94.57 | 477280000 | 25 | Histidine--tRNA ligase, cytoplasmic | HARS |
| 22.86361 | 22.88775 | 24.45732 | 24.02386 | 6 | 6 | 6 | 14 | 14 | 14 | 46.913 | 65.538 | 288110000 | 22 | Basic leucine zipper and W2 domain-containing protein 2 | BZW2 |
| 23.91816 | 22.70073 | 24.44847 | 23.06853 | 5 | 5 | 1 | 17.2 | 17.2 | 5.6 | 38.549 | 38.852 | 192300000 | 19 | Serpin B3 | SERPINB3 |
| 26.57773 | 23.5128 | 24.44695 | 28.69561 | 6 | 6 | 6 | 57.3 | 57.3 | 57.3 | 11.367 | 64.334 | 2455500000 | 57 | Histone H4 | HIST1H4A |
| 24.06159 | 23.10204 | 24.43695 | 24.64174 | 7 | 7 | 7 | 22.9 | 22.9 | 22.9 | 42.592 | 139.47 | 663300000 | 17 | Flap endonuclease 1 | FEN1 |
| 23.36319 | 24.2981 | 24.40587 | 24.71377 | 8 | 8 | 5 | 11.5 | 11.5 | 8.1 | 94.069 | 55.582 | 713560000 | 19 | Phosphorylase;Glycogen phosphorylase, liver form | PYGL |
| 25.92404 | 23.89236 | 24.39846 | 23.46862 | 26 | 26 | 26 | 35.6 | 35.6 | 35.6 | 122.29 | 323.31 | 1472200000 | 46 | Desmoglein-2 | DSG2 |
| 22.89911 | 23.45073 | 24.38627 | 23.9671 | 5 | 5 | 5 | 13.8 | 13.8 | 13.8 | 53.12 | 43.218 | 131300000 | 12 | Splicing factor U2AF 65 kDa subunit | U2AF2 |
| 24.52666 | 23.81773 | 24.38317 | 24.38212 | 19 | 19 | 19 | 23 | 23 | 23 | 107.77 | 187.9 | 1094400000 | 35 | Trifunctional purine biosynthetic protein adenosine-3;Phosphoribosylamine--glycine ligase;Phosphoribosylformylglycinamidine cyclo-ligase;Phosphoribosylglycinamide formyltransferase | GART |
| 24.19393 | 24.07014 | 24.3812 | 23.11009 | 10 | 10 | 10 | 20.1 | 20.1 | 20.1 | 57.563 | 91.331 | 570250000 | 24 | Phenylalanine--tRNA ligase alpha subunit | FARSA |
| 25.05466 | 24.60518 | 24.38093 | 19.99744 | 11 | 11 | 11 | 44.2 | 44.2 | 44.2 | 35.816 | 159.56 | 1609400000 | 21 | Delta(3,5)-Delta(2,4)-dienoyl-CoA isomerase, mitochondrial | ECH1 |
| 26.1045 | 23.9771 | 24.3808 | 24.47122 | 8 | 8 | 8 | 36.9 | 36.9 | 36.9 | 26.559 | 53.101 | 220640000 | 20 | Glutathione S-transferase Mu 3 | GSTM3 |
| 27.57347 | 25.10486 | 24.36226 | 23.22853 | 55 | 55 | 55 | 53.6 | 53.6 | 53.6 | 120.27 | 323.31 | 4091600000 | 87 | Leucine zipper protein 1 | LUZP1 |
| 24.75567 | 24.27491 | 24.36205 | 24.77067 | 11 | 11 | 10 | 36 | 36 | 33 | 45.626 | 94.472 | 950210000 | 41 | 26S protease regulatory subunit 8 | PSMC5 |
| 26.69192 | 23.89004 | 24.35286 | 23.45926 | 15 | 15 | 5 | 56.7 | 56.7 | 16.1 | 37.512 | 323.31 | 2344100000 | 41 | Serine/threonine-protein phosphatase PP1-alpha catalytic subunit;Serine/threonine-protein phosphatase | PPP1CA |
| 24.69136 | 24.41938 | 24.3509 | 24.82512 | 25 | 25 | 25 | 21.9 | 21.9 | 21.9 | 152.78 | 243.8 | 1117500000 | 47 | DNA mismatch repair protein Msh6 | MSH6 |
| 25.99865 | 23.5242 | 24.34861 | 24.22575 | 5 | 5 | 4 | 13.1 | 13.1 | 13.1 | 44.812 | 60.734 | 1466500000 | 19 | RNA-binding protein FUS | FUS |
| 22.97769 | 24.75654 | 24.33967 | 19.46623 | 11 | 11 | 11 | 49.6 | 49.6 | 49.6 | 29.804 | 96.327 | 384700000 | 22 | Prohibitin | PHB |
| 23.28898 | 23.86476 | 24.3293 | 24.18752 | 10 | 10 | 8 | 25.4 | 25.4 | 21.3 | 43.171 | 63.527 | 437580000 | 18 | UV excision repair protein RAD23 homolog B | RAD23B |
| 24.82546 | 24.35239 | 24.32643 | 25.21812 | 7 | 7 | 7 | 29.8 | 29.8 | 29.8 | 27.647 | 47.37 | 166510000 | 15 | Proteasome subunit alpha type-3 | PSMA3 |
| 23.37372 | 24.37597 | 24.32196 | 23.24416 | 20 | 20 | 20 | 7.2 | 7.2 | 7.2 | 367.76 | 136.81 | 486020000 | 29 | Centromere protein F | CENPF |
| 23.78307 | 24.26935 | 24.31927 | 24.01818 | 25 | 25 | 25 | 22.3 | 22.3 | 22.3 | 135.35 | 250 | 545410000 | 41 | Protein diaphanous homolog 1 | DIAPH1 |
| 21.79241 | 22.81415 | 24.3098 | 24.86273 | 9 | 9 | 9 | 30.4 | 30.4 | 30.4 | 33.384 | 69.99 | 260750000 | 18 | SUMO-activating enzyme subunit 1;SUMO-activating enzyme subunit 1, N-terminally processed | SAE1 |
| 26.01653 | 24.7113 | 24.30166 | 22.02303 | 19 | 19 | 11 | 26.1 | 26.1 | 15.8 | 107.47 | 306.98 | 962640000 | 29 | Scaffold attachment factor B2 | SAFB2 |
| 25.15715 | 23.65905 | 24.28961 | 24.29481 | 11 | 11 | 11 | 26.3 | 26.3 | 26.3 | 55.21 | 98.185 | 1058000000 | 26 | tRNA-splicing ligase RtcB homolog | RTCB |
| 24.83335 | 24.29207 | 24.28778 | 24.50875 | 17 | 17 | 17 | 24.5 | 24.5 | 24.5 | 106.93 | 263.65 | 1314100000 | 36 | DNA replication licensing factor MCM2 | MCM2 |
| 24.00427 | 23.89781 | 24.28771 | 24.61352 | 11 | 11 | 6 | 37.2 | 37.2 | 27.7 | 42.613 | 119.95 | 453620000 | 20 | Alpha-centractin | ACTR1A |
| 24.8102 | 23.99421 | 24.28722 | 24.00642 | 13 | 13 | 13 | 32.6 | 32.6 | 32.6 | 62.168 | 120.12 | 825140000 | 24 | Asparagine synthetase [glutamine-hydrolyzing] | ASNS |
| 24.10988 | 24.84264 | 24.28482 | 24.24336 | 13 | 13 | 13 | 28.4 | 28.4 | 28.4 | 56.084 | 101.26 | 737810000 | 32 | Protein RCC2 | RCC2 |
| 23.36239 | 22.83684 | 24.28461 | 24.10398 | 7 | 7 | 7 | 19.1 | 19.1 | 19.1 | 52.351 | 87.509 | 307560000 | 15 | Kynureninase | KYNU |
| 24.6519 | 24.04327 | 24.27831 | 24.97972 | 22 | 22 | 22 | 32.1 | 32.1 | 32.1 | 96.557 | 323.31 | 1451500000 | 41 | DNA replication licensing factor MCM4 | MCM4 |
| 24.13132 | 24.06143 | 24.27377 | 22.72178 | 14 | 14 | 14 | 7.7 | 7.7 | 7.7 | 299.61 | 91.832 | 1088100000 | 25 | Serine/arginine repetitive matrix protein 2 | SRRM2 |
| 25.95844 | 23.67455 | 24.2571 | 24.0416 | 13 | 13 | 10 | 26.6 | 26.6 | 22.8 | 66.392 | 217.17 | 1434700000 | 22 | Phosphatidylinositol-binding clathrin assembly protein | PICALM |
| 22.5519 | 23.2142 | 24.25379 | 24.16269 | 7 | 7 | 7 | 21.8 | 21.8 | 21.8 | 41.806 | 51.201 | 344740000 | 20 | 45 kDa calcium-binding protein | SDF4 |
| 24.6646 | 23.88567 | 24.24858 | 23.35326 | 11 | 11 | 11 | 21.1 | 21.1 | 21.1 | 59.767 | 82.78 | 723380000 | 25 | 26S proteasome non-ATPase regulatory subunit 3 | PSMD3 |
| 26.27665 | 26.3772 | 24.24685 | 24.7749 | 18 | 18 | 18 | 45.2 | 45.2 | 45.2 | 59.578 | 258.27 | 2196100000 | 45 | Nucleolar protein 58 | NOP58 |
| 24.0974 | 27.18187 | 24.2414 | 22.56605 | 23 | 23 | 23 | 37.4 | 37.4 | 37.4 | 73.964 | 219.97 | 2259100000 | 42 | Nucleolar GTP-binding protein 1 | GTPBP4 |
| 24.06595 | 24.31762 | 24.2382 | 24.82445 | 14 | 14 | 14 | 24.5 | 24.5 | 24.5 | 83.549 | 199.14 | 1205400000 | 32 | Procollagen-lysine,2-oxoglutarate 5-dioxygenase 1 | PLOD1 |
| 22.16825 | 24.23929 | 24.23812 | 24.02977 | 7 | 7 | 7 | 30.4 | 30.4 | 30.4 | 40.513 | 75.528 | 267070000 | 13 | DnaJ homolog subfamily B member 11 | DNAJB11 |
| 23.26799 | 23.82231 | 24.23389 | 23.95837 | 6 | 6 | 6 | 14.1 | 14.1 | 14.1 | 45.745 | 43.393 | 465490000 | 18 | DnaJ homolog subfamily A member 2 | DNAJA2 |
| 22.04634 | 23.69456 | 24.20859 | 23.89966 | 11 | 11 | 11 | 22.8 | 22.8 | 22.8 | 79.685 | 102.29 | 269950000 | 29 | Peroxisomal multifunctional enzyme type 2;(3R)-hydroxyacyl-CoA dehydrogenase;Enoyl-CoA hydratase 2 | HSD17B4 |
| 24.05614 | 23.67174 | 24.20628 | 24.53937 | 10 | 10 | 10 | 28.4 | 28.4 | 28.4 | 36.248 | 73.571 | 475360000 | 29 | Macrophage-capping protein | CAPG |
| 24.38745 | 23.10835 | 24.2062 | 24.25811 | 6 | 6 | 6 | 43 | 43 | 43 | 20.567 | 39.276 | 103360000 | 16 | Cysteine and glycine-rich protein 1 | CSRP1 |
| 24.56098 | 27.1962 | 24.20083 | 24.34266 | 12 | 12 | 12 | 49.5 | 49.5 | 49.5 | 47.687 | 323.31 | 1437900000 | 41 | Sequestosome-1 | SQSTM1 |
| 24.634 | 26.0856 | 24.19723 | 22.60987 | 12 | 12 | 11 | 43.9 | 43.9 | 40.5 | 33.784 | 117.21 | 1218200000 | 34 | rRNA 2-O-methyltransferase fibrillarin | FBL |
| 22.94464 | 23.6221 | 24.19378 | 24.44506 | 3 | 3 | 3 | 9.6 | 9.6 | 9.6 | 42.153 | 37.743 | 281420000 | 11 | Proteasomal ubiquitin receptor ADRM1 | ADRM1 |
| 24.7962 | 24.84532 | 24.16515 | 23.97395 | 13 | 11 | 11 | 28.6 | 24.6 | 24.6 | 63.48 | 132.66 | 1018000000 | 23 | Insulin-like growth factor 2 mRNA-binding protein 1 | IGF2BP1 |
| 23.00051 | 22.71967 | 24.16254 | 18.53533 | 7 | 7 | 6 | 37.3 | 37.3 | 28.2 | 19.997 | 101.32 | 182700000 | 12 | Acidic leucine-rich nuclear phosphoprotein 32 family member A | ANP32A |
| 22.91814 | 22.96598 | 24.16123 | 24.22781 | 3 | 3 | 3 | 22.5 | 22.5 | 22.5 | 21.057 | 18.745 | 110290000 | 10 | Phosphatidylethanolamine-binding protein 1;Hippocampal cholinergic neurostimulating peptide | PEBP1 |
| 25.10849 | 24.53439 | 24.16123 | 24.17699 | 8 | 8 | 8 | 11.5 | 11.5 | 11.5 | 76.861 | 74.313 | 1438200000 | 24 | Caprin-1 | CAPRIN1 |
| 23.55719 | 24.85719 | 24.15854 | 25.54245 | 9 | 9 | 9 | 22.5 | 22.5 | 22.5 | 48.755 | 173.46 | 721790000 | 29 | Dihydrolipoyllysine-residue succinyltransferase component of 2-oxoglutarate dehydrogenase complex, mitochondrial | DLST |
| 25.88166 | 24.93454 | 24.15345 | 22.97141 | 31 | 31 | 31 | 28.5 | 28.5 | 28.5 | 148.09 | 323.31 | 1088200000 | 46 | Nuclear pore complex protein Nup155 | NUP155 |
| 23.02293 | 23.32141 | 24.14608 | 23.96746 | 7 | 7 | 7 | 19.9 | 19.9 | 19.9 | 39.591 | 67.062 | 322320000 | 19 | Isocitrate dehydrogenase [NAD] subunit alpha, mitochondrial | IDH3A |
| 23.9393 | 24.56174 | 24.14562 | 19.66924 | 15 | 15 | 15 | 52.5 | 52.5 | 52.5 | 33.296 | 155.51 | 501780000 | 26 | Prohibitin-2 | PHB2 |
| 23.19334 | 25.12187 | 24.13884 | 23.30939 | 12 | 12 | 12 | 17.3 | 17.3 | 17.3 | 87.435 | 90.876 | 854760000 | 22 | Nucleolar transcription factor 1 | UBTF |
| 22.71869 | 23.6536 | 24.13124 | 23.54722 | 8 | 8 | 8 | 12.7 | 12.7 | 12.7 | 71.349 | 96.303 | 518690000 | 15 | Cytoplasmic dynein 1 intermediate chain 2 | DYNC1I2 |
| 21.92303 | 23.93265 | 24.13093 | 24.70504 | 3 | 3 | 3 | 40 | 40 | 40 | 10.884 | 22.703 | 320230000 | 36 | Mammaglobin-B | SCGB2A1 |
| 23.96208 | 23.99688 | 24.1277 | 24.96345 | 11 | 11 | 10 | 17.3 | 17.3 | 15.6 | 82.968 | 123.41 | 950590000 | 18 | Protein transport protein Sec23A | SEC23A |
| 22.6486 | 23.72889 | 24.12558 | 24.46955 | 8 | 8 | 8 | 27.7 | 27.7 | 27.7 | 38.868 | 72.854 | 316860000 | 16 | Na(+)/H(+) exchange regulatory cofactor NHE-RF1 | SLC9A3R1 |
| 24.99658 | 25.03909 | 24.12518 | 22.78991 | 19 | 19 | 19 | 26.8 | 26.8 | 26.8 | 107.14 | 222.31 | 1478100000 | 34 | Coatomer subunit beta | COPB1 |
| 22.77221 | 23.93517 | 24.12337 | 24.42688 | 12 | 12 | 12 | 23.1 | 23.1 | 23.1 | 66.022 | 94.041 | 382180000 | 20 | Cytoskeleton-associated protein 4 | CKAP4 |
| 24.37571 | 24.44569 | 24.11426 | 25.26792 | 26 | 5 | 4 | 56.6 | 15.6 | 13.1 | 57.73 | 139.78 | 796580000 | 23 | Tubulin alpha-1C chain | TUBA1C |
| 23.58187 | 23.9686 | 24.10973 | 24.16139 | 11 | 11 | 11 | 42.8 | 42.8 | 42.8 | 35.554 | 103.54 | 408480000 | 24 | DNA-(apurinic or apyrimidinic site) lyase;DNA-(apurinic or apyrimidinic site) lyase, mitochondrial | APEX1 |
| 30.51032 | 24.14934 | 24.10925 | 23.24714 | 36 | 20 | 10 | 80.6 | 55.2 | 18.1 | 28.521 | 323.31 | 2.8882E+10 | 92 | Tropomyosin alpha-4 chain | TPM4 |
| 22.11801 | 24.49881 | 24.10574 | 23.59912 | 3 | 3 | 3 | 31.2 | 31.2 | 31.2 | 10.834 | 20.373 | 199790000 | 14 | Protein S100-A8;Protein S100-A8, N-terminally processed | S100A8 |
| 23.06997 | 23.54946 | 24.10534 | 24.40458 | 9 | 9 | 9 | 31.8 | 31.8 | 31.8 | 37.804 | 67.579 | 194530000 | 18 | Suppressor of G2 allele of SKP1 homolog | SUGT1 |
| 23.48689 | 23.94735 | 24.10221 | 24.36259 | 14 | 14 | 14 | 18.6 | 18.6 | 18.6 | 102.9 | 144.46 | 746590000 | 28 | Cell cycle and apoptosis regulator protein 2 | CCAR2 |
| 24.37597 | 17.75681 | 24.09144 | 19.96772 | 13 | 2 | 2 | 5.4 | 1 | 1 | 231.21 | 16.201 | 802650000 | 9 | Myosin-14 | MYH14 |
| 23.63145 | 24.03884 | 24.09136 | 24.71057 | 5 | 5 | 5 | 42.3 | 42.3 | 42.3 | 19.891 | 38.625 | 95271000 | 15 | Protein DJ-1 | PARK7 |
| 21.44627 | 23.78975 | 24.09047 | 24.62823 | 3 | 3 | 3 | 6.7 | 6.7 | 6.7 | 44.882 | 23.275 | 455540000 | 10 | Lysosome-associated membrane glycoprotein 1 | LAMP1 |
| 23.43835 | 23.54769 | 24.08853 | 23.66188 | 8 | 8 | 8 | 19.1 | 19.1 | 19.1 | 60.632 | 56.941 | 410750000 | 19 | Prolyl 4-hydroxylase subunit alpha-2 | P4HA2 |
| 26.84566 | 22.56602 | 24.08472 | 23.07331 | 10 | 10 | 10 | 45 | 45 | 45 | 36.567 | 323.31 | 2298300000 | 27 | PRKC apoptosis WT1 regulator protein | PAWR |
| 24.16546 | 24.16607 | 24.07969 | 25.12597 | 6 | 6 | 6 | 12.1 | 12.1 | 12.1 | 66.112 | 58.382 | 810740000 | 26 | Eukaryotic translation initiation factor 4B | EIF4B |
| 23.99291 | 24.66508 | 24.07455 | 24.52987 | 11 | 11 | 11 | 37.6 | 37.6 | 37.6 | 40.542 | 155.25 | 374390000 | 23 | Developmentally-regulated GTP-binding protein 1 | DRG1 |
| 21.28799 | 23.7318 | 24.05672 | 24.07022 | 12 | 12 | 12 | 19.2 | 19.2 | 19.2 | 83.283 | 83.378 | 389280000 | 31 | Polymeric immunoglobulin receptor;Secretory component | PIGR |
| 27.32397 | 22.80168 | 24.05598 | 22.79321 | 49 | 49 | 49 | 47.3 | 47.3 | 47.3 | 120.18 | 323.31 | 3310500000 | 73 | Cytospin-A | SPECC1L;SPECC1L-ADORA2A |
| 22.58128 | 24.07643 | 24.05175 | 23.44746 | 9 | 9 | 9 | 17.2 | 17.2 | 17.2 | 76.107 | 91.291 | 501090000 | 16 | A-kinase anchor protein 8 | AKAP8 |
| 23.34543 | 23.76991 | 24.04502 | 23.66036 | 9 | 9 | 9 | 27.2 | 27.2 | 27.2 | 39.646 | 99.722 | 388500000 | 24 | Mitochondrial import inner membrane translocase subunit TIM50 | TIMM50 |
| 26.16588 | 25.50133 | 24.03674 | 25.69567 | 12 | 12 | 12 | 37 | 37 | 37 | 44.868 | 149.35 | 2094500000 | 46 | DnaJ homolog subfamily A member 1 | DNAJA1 |
| 25.09704 | 26.61863 | 24.02791 | 26.1387 | 35 | 35 | 35 | 41.7 | 41.7 | 41.7 | 108.63 | 323.31 | 1345200000 | 89 | Ribosome-binding protein 1 | RRBP1 |
| 26.84554 | 24.43892 | 24.02445 | 24.62801 | 5 | 5 | 4 | 23.6 | 23.6 | 20.4 | 25.486 | 34.897 | 340850000 | 12 | U2 small nuclear ribonucleoprotein B | SNRPB2 |
| 25.62667 | 23.3538 | 24.02047 | 24.30334 | 12 | 12 | 12 | 64.6 | 64.6 | 64.6 | 28.994 | 209.4 | 1315900000 | 31 | Emerin | EMD |
| 22.94204 | 23.64658 | 24.01385 | 24.06085 | 10 | 10 | 4 | 36.5 | 36.5 | 18.9 | 34.769 | 95.631 | 342290000 | 23 | Ribose-phosphate pyrophosphokinase 2 | PRPS2 |
| 25.60281 | 24.62349 | 24.01376 | 25.10825 | 8 | 8 | 8 | 37.5 | 37.5 | 37.5 | 27.887 | 56.386 | 280130000 | 33 | Proteasome subunit alpha type-7 | PSMA7 |
| 24.48745 | 24.60818 | 24.01299 | 25.69578 | 4 | 4 | 4 | 35.5 | 35.5 | 35.5 | 11.758 | 34.55 | 169250000 | 14 | Prothymosin alpha;Prothymosin alpha, N-terminally processed;Thymosin alpha-1 | PTMA |
| 22.28062 | 23.16196 | 24.01086 | 23.63334 | 7 | 7 | 7 | 19.2 | 19.2 | 19.2 | 59.747 | 91.782 | 246370000 | 16 | Vang-like protein 1 | VANGL1 |
| 18.46163 | 23.2078 | 24.01078 | 23.79124 | 9 | 9 | 9 | 23.8 | 23.8 | 23.8 | 52.645 | 82.031 | 184200000 | 13 | Cytochrome b-c1 complex subunit 1, mitochondrial | UQCRC1 |
| 24.88595 | 23.59628 | 23.99542 | 23.45023 | 10 | 10 | 10 | 15.8 | 15.8 | 15.8 | 71.635 | 84.108 | 698270000 | 22 | Procollagen galactosyltransferase 1 | COLGALT1 |
| 22.51641 | 26.83978 | 23.98181 | 21.76032 | 13 | 13 | 13 | 28.3 | 28.3 | 28.3 | 41.401 | 145.49 | 667310000 | 27 | Ribosome biogenesis protein BRX1 homolog | BRIX1 |
| 22.66353 | 23.62968 | 23.98042 | 19.64433 | 6 | 6 | 6 | 18.9 | 18.9 | 18.9 | 47.151 | 48.849 | 227050000 | 13 | Saccharopine dehydrogenase-like oxidoreductase | SCCPDH |
| 23.01939 | 23.50312 | 23.97797 | 24.29495 | 8 | 8 | 8 | 23.7 | 23.7 | 23.7 | 45.786 | 60.622 | 278130000 | 18 | Ancient ubiquitous protein 1 | AUP1 |
| 24.70382 | 23.51941 | 23.97684 | 24.26413 | 7 | 7 | 7 | 38.1 | 38.1 | 38.1 | 24.593 | 52.551 | 120540000 | 20 | Vesicle-trafficking protein SEC22b | SEC22B |
| 24.50088 | 24.21985 | 23.9736 | 24.85833 | 3 | 3 | 3 | 19.7 | 19.7 | 19.7 | 18.998 | 22.954 | 198180000 | 17 | Translocon-associated protein subunit delta | SSR4 |
| 24.27569 | 25.14044 | 23.96772 | 25.38745 | 5 | 5 | 5 | 43.8 | 43.8 | 43.8 | 17.031 | 34.543 | 174920000 | 12 | Peroxiredoxin-5, mitochondrial | PRDX5 |
| 23.975 | 23.5412 | 23.955 | 24.3889 | 14 | 14 | 14 | 22.6 | 22.6 | 22.6 | 84.784 | 99.663 | 829520000 | 22 | Procollagen-lysine,2-oxoglutarate 5-dioxygenase 3 | PLOD3 |
| 22.76044 | 23.19502 | 23.95127 | 24.25278 | 9 | 9 | 4 | 23.9 | 23.9 | 13.4 | 62.518 | 87.769 | 270390000 | 12 | Ubiquilin-1 | UBQLN1 |
| 21.50678 | 23.55016 | 23.94949 | 23.44139 | 14 | 14 | 14 | 8 | 8 | 8 | 205.11 | 95.137 | 328090000 | 23 | Nuclear pore membrane glycoprotein 210 | NUP210 |
| 25.64669 | 23.70081 | 23.94074 | 24.64944 | 7 | 6 | 6 | 45.5 | 41.2 | 41.2 | 18.506 | 44.744 | 225550000 | 10 | Destrin | DSTN |
| 24.77726 | 25.86448 | 23.94056 | 22.47264 | 23 | 23 | 23 | 26.4 | 26.4 | 26.4 | 115.73 | 323.31 | 1556000000 | 38 | N-acetyltransferase 10 | NAT10 |
| 26.79154 | 23.96252 | 23.93859 | 21.42132 | 12 | 12 | 11 | 47.2 | 47.2 | 44.4 | 27.744 | 123.15 | 2344700000 | 37 | Serine/arginine-rich splicing factor 1 | SRSF1 |
| 21.57473 | 23.29432 | 23.93643 | 24.49387 | 5 | 5 | 5 | 6.6 | 6.6 | 6.6 | 73.602 | 33.943 | 102380000 | 14 | Nucleolar and coiled-body phosphoprotein 1 | NOLC1 |
| 24.16699 | 23.73293 | 23.92307 | 24.54575 | 11 | 11 | 11 | 22.4 | 22.4 | 22.4 | 50.519 | 79.705 | 616430000 | 26 | ATP-dependent RNA helicase DDX19A;ATP-dependent RNA helicase DDX19B | DDX19A;DDX19B |
| 25.02614 | 23.14066 | 23.91616 | 23.31893 | 15 | 15 | 13 | 26.9 | 26.9 | 22.9 | 80.419 | 129.88 | 1176700000 | 26 | Long-chain-fatty-acid--CoA ligase 3 | ACSL3 |
| 23.04966 | 23.55192 | 23.91187 | 24.70946 | 5 | 4 | 4 | 21.3 | 17.9 | 17.9 | 29.224 | 25.497 | 92614000 | 10 | Protein canopy homolog 2 | CNPY2 |
| 25.56337 | 24.61341 | 23.91013 | 23.81137 | 26 | 26 | 18 | 26.6 | 26.6 | 19.6 | 116.78 | 227.71 | 1875400000 | 44 | SWI/SNF-related matrix-associated actin-dependent regulator of chromatin subfamily A member 5 | SMARCA5 |
| 24.62533 | 24.10374 | 23.898 | 20.14925 | 14 | 14 | 14 | 52.3 | 52.3 | 52.3 | 32.193 | 147.02 | 721080000 | 27 | Four and a half LIM domains protein 2 | FHL2 |
| 21.20497 | 28.07449 | 23.89597 | 23.15106 | 17 | 17 | 17 | 40 | 40 | 40 | 61.68 | 190.7 | 1550700000 | 26 | Glypican-1;Secreted glypican-1 | GPC1 |
| 23.36573 | 23.43974 | 23.89393 | 24.18533 | 9 | 9 | 9 | 34.4 | 34.4 | 34.4 | 33.337 | 77.593 | 332090000 | 20 | Stomatin-like protein 2, mitochondrial | STOML2 |
| 27.00045 | 25.6726 | 23.89245 | 24.50355 | 54 | 54 | 54 | 45.3 | 45.3 | 45.3 | 147.18 | 323.31 | 2638300000 | 81 | Structural maintenance of chromosomes protein 4;Structural maintenance of chromosomes protein | SMC4 |
| 24.64504 | 23.74314 | 23.89162 | 23.76446 | 14 | 14 | 14 | 20.6 | 20.6 | 20.6 | 85.746 | 181.17 | 966920000 | 28 | Cold shock domain-containing protein E1 | CSDE1 |
| 23.31424 | 23.13005 | 23.8812 | 23.28715 | 5 | 5 | 5 | 44.9 | 44.9 | 44.9 | 13.133 | 33.595 | 73158000 | 13 | Transcription elongation factor B polypeptide 2 | TCEB2 |
| 24.24583 | 23.94717 | 23.88101 | 23.88316 | 6 | 6 | 6 | 15.7 | 15.7 | 15.7 | 51.691 | 85.456 | 601200000 | 14 | Serine/threonine-protein phosphatase 2A 55 kDa regulatory subunit B alpha isoform | PPP2R2A |
| 23.42008 | 23.4929 | 23.87764 | 24.16921 | 10 | 10 | 10 | 18.3 | 18.3 | 18.3 | 66.115 | 68.802 | 495680000 | 23 | Phenylalanine--tRNA ligase beta subunit | FARSB |
| 24.51779 | 23.6698 | 23.87586 | 23.77555 | 13 | 13 | 13 | 20.7 | 20.7 | 20.7 | 82.021 | 323.31 | 978710000 | 25 | HIV Tat-specific factor 1 | HTATSF1 |
| 22.36909 | 23.72931 | 23.87408 | 23.85576 | 9 | 9 | 9 | 24.8 | 24.8 | 24.8 | 52.385 | 98.171 | 251760000 | 15 | Ubiquitin carboxyl-terminal hydrolase;Ubiquitin carboxyl-terminal hydrolase 14 | USP14 |
| 26.0618 | 28.02253 | 23.87108 | 25.07092 | 3 | 3 | 3 | 28.6 | 28.6 | 28.6 | 12.254 | 23.578 | 731010000 | 21 | 60S ribosomal protein L36 | RPL36 |
| 23.99869 | 23.87145 | 23.86599 | 23.54498 | 11 | 11 | 11 | 42.9 | 42.9 | 42.9 | 40.968 | 152.77 | 534540000 | 27 | mRNA export factor | RAE1 |
| 25.03871 | 26.08982 | 23.86259 | 23.56291 | 3 | 3 | 3 | 15.5 | 15.5 | 15.5 | 26.599 | 23.826 | 193020000 | 8 | Eukaryotic translation initiation factor 6 | EIF6 |
| 21.65338 | 23.34259 | 23.86174 | 24.41732 | 8 | 8 | 8 | 22 | 22 | 22 | 46.247 | 51.13 | 204590000 | 15 | Aspartate aminotransferase, cytoplasmic;Aspartate aminotransferase | GOT1 |
| 22.90353 | 23.60026 | 23.8551 | 24.38588 | 10 | 10 | 10 | 23.9 | 23.9 | 23.9 | 53.801 | 81.606 | 322490000 | 17 | 4-trimethylaminobutyraldehyde dehydrogenase | ALDH9A1 |
| 22.79337 | 24.84052 | 23.85415 | 23.29936 | 11 | 11 | 11 | 25.6 | 25.6 | 25.6 | 63.836 | 133.79 | 455890000 | 25 | Protein LYRIC | MTDH |
| 24.8998 | 23.57738 | 23.85405 | 23.78257 | 14 | 14 | 14 | 33.4 | 33.4 | 33.4 | 56.44 | 152.2 | 763510000 | 28 | DnaJ homolog subfamily C member 7 | DNAJC7 |
| 21.66939 | 19.59428 | 23.84995 | 26.64554 | 6 | 6 | 6 | 9.8 | 9.8 | 9.8 | 64.365 | 40.022 | 273100000 | 8 | Leucine-rich repeat-containing protein 15 | LRRC15 |
| 22.57665 | 21.90643 | 23.84967 | 22.75853 | 7 | 7 | 7 | 16.2 | 16.2 | 16.2 | 65.459 | 51.929 | 209480000 | 12 | Glutaminase kidney isoform, mitochondrial | GLS |
| 23.14456 | 24.86877 | 23.84728 | 24.2363 | 9 | 9 | 9 | 35 | 35 | 35 | 36.588 | 68.537 | 384350000 | 18 | WD repeat-containing protein 5 | WDR5 |
| 26.62324 | 24.29376 | 23.8447 | 24.51208 | 23 | 23 | 23 | 42.7 | 42.7 | 42.7 | 61.585 | 323.31 | 3158200000 | 48 | Src substrate cortactin | CTTN |
| 22.96072 | 22.19027 | 23.84096 | 23.41067 | 8 | 8 | 8 | 15.4 | 15.4 | 15.4 | 68.137 | 61.066 | 304310000 | 14 | Replication protein A 70 kDa DNA-binding subunit;Replication protein A 70 kDa DNA-binding subunit, N-terminally processed | RPA1 |
| 17.8669 | 22.76095 | 23.83856 | 23.67541 | 7 | 1 | 1 | 40.8 | 3.4 | 3.4 | 32.642 | 6.9145 | 50106000 | 6 | Nucleoside diphosphate kinase;Nucleoside diphosphate kinase B | NME1-NME2;NME2;NME1 |
| 28.52981 | 23.58061 | 23.83759 | 24.53843 | 57 | 35 | 35 | 74.2 | 49.7 | 49.7 | 103.06 | 323.31 | 1.0955E+10 | 54 | Alpha-actinin-1 | ACTN1 |
| 23.23024 | 22.6956 | 23.83567 | 23.88045 | 3 | 3 | 3 | 18.4 | 18.4 | 18.4 | 31.554 | 21.313 | 270580000 | 11 | Myristoylated alanine-rich C-kinase substrate | MARCKS |
| 22.99566 | 23.1559 | 23.82648 | 26.63865 | 7 | 7 | 7 | 10.3 | 10.3 | 10.3 | 80.496 | 61.196 | 334350000 | 28 | Plakophilin-1 | PKP1 |
| 20.85392 | 23.77102 | 23.82357 | 24.34631 | 7 | 7 | 7 | 14.5 | 14.5 | 14.5 | 54.812 | 45.067 | 162920000 | 15 | CAAX prenyl protease 1 homolog | ZMPSTE24 |
| 27.26616 | 24.80774 | 23.80725 | 24.76116 | 61 | 61 | 61 | 25.7 | 25.7 | 25.7 | 629.09 | 323.31 | 6053800000 | 116 | Neuroblast differentiation-associated protein AHNAK | AHNAK |
| 23.8375 | 23.48959 | 23.80656 | 24.28863 | 7 | 7 | 7 | 36.7 | 36.7 | 36.7 | 23.897 | 44.496 | 81506000 | 20 | Ras-related protein Rab-14 | RAB14 |
| 23.90793 | 23.82182 | 23.80587 | 24.37749 | 11 | 11 | 11 | 29.1 | 29.1 | 29.1 | 52.771 | 80.991 | 446240000 | 26 | Cytosol aminopeptidase | LAP3 |
| 21.00696 | 16.8518 | 23.80144 | 25.05217 | 3 | 3 | 3 | 37.7 | 37.7 | 37.7 | 11.237 | 23.599 | 231660000 | 19 | Ig lambda-3 chain C regions;Ig lambda-2 chain C regions;Ig lambda-1 chain C regions;Ig lambda-6 chain C region;Immunoglobulin lambda-like polypeptide 5 | IGLC3;IGLC2;IGLC1;IGLC6;IGLL5 |
| 23.34042 | 23.70229 | 23.80065 | 24.64482 | 11 | 11 | 11 | 14.1 | 14.1 | 14.1 | 107.89 | 220.04 | 658120000 | 24 | General vesicular transport factor p115 | USO1 |
| 23.75583 | 23.22986 | 23.78626 | 23.32691 | 11 | 11 | 11 | 19.9 | 19.9 | 19.9 | 67.314 | 109.55 | 493120000 | 15 | ATP-binding cassette sub-family E member 1 | ABCE1 |
| 23.75093 | 25.0585 | 23.77966 | 23.35272 | 17 | 17 | 17 | 62.1 | 62.1 | 62.1 | 35.503 | 133.13 | 503290000 | 46 | Malate dehydrogenase, mitochondrial;Malate dehydrogenase | MDH2 |
| 22.69746 | 23.68752 | 23.77866 | 23.10019 | 4 | 4 | 4 | 7.5 | 7.5 | 7.5 | 70.702 | 28.738 | 243570000 | 14 | Lamin-B receptor | LBR |
| 22.46184 | 23.52695 | 23.77696 | 24.11227 | 5 | 5 | 5 | 13.9 | 13.9 | 13.9 | 35.853 | 33.308 | 206080000 | 10 | Aldose reductase | AKR1B1 |
| 23.95385 | 24.21208 | 23.77605 | 23.72005 | 12 | 12 | 12 | 20.3 | 20.3 | 20.3 | 91.679 | 227.65 | 872580000 | 20 | ATP-binding cassette sub-family F member 1 | ABCF1 |
| 23.47902 | 23.58199 | 23.77304 | 21.69392 | 6 | 6 | 4 | 30.7 | 30.7 | 20.9 | 24.893 | 52.829 | 191020000 | 16 | High mobility group protein B1;Putative high mobility group protein B1-like 1 | HMGB1;HMGB1P1 |
| 22.83869 | 23.38811 | 23.77102 | 23.92931 | 6 | 6 | 6 | 13.9 | 13.9 | 13.9 | 54.191 | 42.359 | 373710000 | 14 | Mitochondrial-processing peptidase subunit beta | PMPCB |
| 24.78477 | 23.0592 | 23.76931 | 18.50297 | 6 | 6 | 6 | 14.8 | 14.8 | 14.8 | 52.488 | 68.299 | 739880000 | 15 | DnaJ homolog subfamily A member 3, mitochondrial | DNAJA3 |
| 24.16898 | 17.53218 | 23.76709 | 24.50173 | 3 | 3 | 3 | 12.8 | 12.8 | 12.8 | 26.21 | 19.808 | 101560000 | 9 | Transmembrane protein 109 | TMEM109 |
| 25.84814 | 23.9736 | 23.76658 | 24.21475 | 9 | 9 | 9 | 37.9 | 37.9 | 37.9 | 26.694 | 81.813 | 218880000 | 16 | MICOS complex subunit MIC19 | CHCHD3 |
| 24.85119 | 25.70282 | 23.76446 | 23.60264 | 4 | 4 | 4 | 24.4 | 24.4 | 24.4 | 14.551 | 43.762 | 210890000 | 15 | 60S ribosomal protein L35 | RPL35 |
| 22.21741 | 23.79174 | 23.76355 | 23.81294 | 17 | 17 | 17 | 7.7 | 7.7 | 7.7 | 292.26 | 157.3 | 577030000 | 32 | Collagen alpha-1(VII) chain | COL7A1 |
| 24.26449 | 23.38824 | 23.75918 | 24.19001 | 10 | 10 | 10 | 21.4 | 21.4 | 21.4 | 64.735 | 98.133 | 588940000 | 25 | V-type proton ATPase catalytic subunit A | ATP6V1A |
| 23.62667 | 23.49984 | 23.75776 | 24.37365 | 14 | 14 | 14 | 19.1 | 19.1 | 19.1 | 96.221 | 108.39 | 680280000 | 21 | Serrate RNA effector molecule homolog | SRRT |
| 22.98191 | 23.905 | 23.74766 | 23.68891 | 6 | 6 | 6 | 13.7 | 13.7 | 13.7 | 53.879 | 38.202 | 250400000 | 8 | Nucleobindin-1 | NUCB1 |
| 23.53326 | 24.49497 | 23.74725 | 25.27739 | 6 | 6 | 6 | 18.4 | 18.4 | 18.4 | 41.331 | 46.722 | 586780000 | 23 | Hsc70-interacting protein;Putative protein FAM10A5;Putative protein FAM10A4 | ST13;ST13P5;ST13P4 |
| 23.32567 | 22.89787 | 23.74314 | 24.66536 | 9 | 9 | 9 | 36.7 | 36.7 | 36.7 | 37.2 | 79.77 | 434570000 | 21 | Pyruvate dehydrogenase E1 component subunit beta, mitochondrial | PDHB |
| 22.9268 | 23.40976 | 23.74181 | 24.1303 | 9 | 9 | 5 | 24.9 | 24.9 | 14.6 | 43.083 | 73.606 | 296080000 | 18 | Inhibitor of nuclear factor kappa-B kinase-interacting protein | IKBIP |
| 22.14619 | 23.17062 | 23.73903 | 24.67621 | 6 | 6 | 6 | 18 | 18 | 18 | 47.873 | 45.389 | 240450000 | 16 | COP9 signalosome complex subunit 3 | COPS3 |
| 22.69381 | 23.54722 | 23.73892 | 24.13289 | 6 | 6 | 6 | 14.7 | 14.7 | 14.7 | 57.548 | 43.523 | 314550000 | 11 | Protein ERGIC-53 | LMAN1 |
| 23.19825 | 23.96895 | 23.72692 | 23.75562 | 13 | 13 | 13 | 16.2 | 16.2 | 16.2 | 101.27 | 97.19 | 585230000 | 19 | N-alpha-acetyltransferase 15, NatA auxiliary subunit | NAA15 |
| 25.73918 | 23.9604 | 23.7263 | 23.55098 | 13 | 13 | 12 | 40.7 | 40.7 | 38.4 | 39.31 | 99.095 | 1265300000 | 30 | Aurora kinase B | AURKB |
| 23.36159 | 24.26871 | 23.72255 | 23.95793 | 18 | 18 | 13 | 17.5 | 17.5 | 13.5 | 136.18 | 172.52 | 380840000 | 26 | SWI/SNF complex subunit SMARCC2 | SMARCC2 |
| 22.75154 | 23.30633 | 23.72234 | 23.49069 | 7 | 7 | 7 | 20.8 | 20.8 | 20.8 | 54.458 | 93.148 | 503530000 | 12 | Aconitate hydratase, mitochondrial | ACO2 |
| 25.12029 | 24.7417 | 23.71471 | 23.84661 | 19 | 12 | 11 | 34.9 | 20.1 | 18.7 | 69.578 | 85.747 | 1294500000 | 30 | Polyadenylate-binding protein 4 | PABPC4 |
| 23.62355 | 22.95853 | 23.71272 | 23.2535 | 7 | 7 | 6 | 17.1 | 17.1 | 14.6 | 54.847 | 50.171 | 396050000 | 15 | Fatty aldehyde dehydrogenase | ALDH3A2 |
| 22.91138 | 23.29446 | 23.71209 | 23.15277 | 6 | 6 | 6 | 16.9 | 16.9 | 16.9 | 47.569 | 55.36 | 114080000 | 19 | Nucleolar and spindle-associated protein 1 | NUSAP1 |
| 23.38627 | 23.38509 | 23.70893 | 24.27796 | 7 | 7 | 7 | 46.4 | 46.4 | 46.4 | 15.693 | 42.396 | 84489000 | 14 | Cellular retinoic acid-binding protein 2 | CRABP2 |
| 22.48719 | 23.57738 | 23.70704 | 23.53552 | 9 | 9 | 9 | 30.8 | 30.8 | 30.8 | 36.573 | 78.698 | 198390000 | 17 | Alcohol dehydrogenase [NADP(+)] | AKR1A1 |
| 25.55066 | 23.84163 | 23.70039 | 24.0053 | 5 | 5 | 4 | 28.3 | 28.3 | 22.2 | 20.511 | 34.882 | 217870000 | 14 | ADP-ribosylation factor 4 | ARF4 |
| 23.0331 | 23.63533 | 23.69816 | 23.40185 | 9 | 9 | 9 | 24.5 | 24.5 | 24.5 | 36.805 | 66.726 | 332870000 | 23 | 28S ribosomal protein S22, mitochondrial | MRPS22 |
| 23.35689 | 23.67174 | 23.69381 | 23.91862 | 7 | 7 | 7 | 36.3 | 36.3 | 36.3 | 35.54 | 109.42 | 359940000 | 16 | Protein SEC13 homolog | SEC13 |
| 22.15261 | 24.83277 | 23.68827 | 25.26055 | 13 | 13 | 13 | 49.2 | 49.2 | 49.2 | 46.946 | 159.48 | 467140000 | 29 | Perilipin-3 | PLIN3 |
| 23.25114 | 23.44063 | 23.68539 | 23.69922 | 8 | 8 | 8 | 13.5 | 13.5 | 13.5 | 72.683 | 52.399 | 302210000 | 15 | Protein arginine N-methyltransferase 5;Protein arginine N-methyltransferase 5, N-terminally processed;Protein arginine N-methyltransferase 5 | PRMT5 |
| 24.05928 | 23.47754 | 23.66742 | 23.69402 | 7 | 7 | 7 | 47.1 | 47.1 | 47.1 | 17.668 | 41.725 | 68023000 | 17 | Ras-related protein Rab-11A;Ras-related protein Rab-11B | RAB11A;RAB11B |
| 21.37459 | 23.32553 | 23.66395 | 23.40613 | 3 | 3 | 3 | 9.6 | 9.6 | 9.6 | 51.539 | 19.857 | 150880000 | 12 | Corneodesmosin | CDSN |
| 26.49834 | 24.06447 | 23.66145 | 19.24302 | 20 | 20 | 20 | 73.2 | 73.2 | 73.2 | 30.628 | 277.01 | 2153000000 | 41 | F-actin-capping protein subunit beta | CAPZB |
| 23.14838 | 23.22685 | 23.66036 | 18.80259 | 5 | 5 | 5 | 25.4 | 25.4 | 25.4 | 34.93 | 63.915 | 223920000 | 19 | Leucine-rich repeat-containing protein 59 | LRRC59 |
| 21.90014 | 23.13105 | 23.65709 | 23.46389 | 8 | 8 | 8 | 22.3 | 22.3 | 22.3 | 38.685 | 56.004 | 162090000 | 18 | Leukocyte elastase inhibitor | SERPINB1 |
| 23.77082 | 24.26571 | 23.65207 | 24.23885 | 11 | 11 | 11 | 35 | 35 | 35 | 39.681 | 84.427 | 430920000 | 20 | Replication factor C subunit 4 | RFC4 |
| 32.40647 | 24.69652 | 23.64295 | 24.635 | 68 | 68 | 3 | 55 | 55 | 4.4 | 93.23 | 323.31 | 1.3877E+11 | 232 | Caldesmon | CALD1 |
| 23.23488 | 23.85348 | 23.63045 | 24.17805 | 6 | 6 | 3 | 23.9 | 23.9 | 14.5 | 36.105 | 39.779 | 402860000 | 26 | Ig gamma-1 chain C region | IGHG1 |
| 22.94546 | 24.58589 | 23.62701 | 25.93299 | 21 | 21 | 21 | 18.2 | 18.2 | 18.2 | 144.73 | 198.6 | 486130000 | 36 | Phosphoribosylformylglycinamidine synthase | PFAS |
| 26.02737 | 26.08552 | 23.62533 | 23.89328 | 17 | 17 | 17 | 34.7 | 34.7 | 34.7 | 66.049 | 192.85 | 1969300000 | 44 | Nucleolar protein 56 | NOP56 |
| 23.86335 | 23.20247 | 23.62522 | 23.57669 | 8 | 8 | 8 | 26.5 | 26.5 | 26.5 | 37.025 | 66.157 | 367260000 | 21 | 26S proteasome non-ATPase regulatory subunit 7 | PSMD7 |
| 23.42342 | 23.2754 | 23.62433 | 23.13696 | 14 | 4 | 4 | 34.2 | 12.4 | 12.4 | 46.701 | 32.802 | 338010000 | 11 | Tubulin beta-6 chain | TUBB6 |
| 23.56244 | 22.87136 | 23.61616 | 24.32113 | 4 | 4 | 4 | 34.3 | 34.3 | 34.3 | 15.138 | 26.716 | 69150000 | 10 | Ras-related protein Rab-7a | RAB7A |
| 22.58763 | 24.95996 | 23.61583 | 22.22594 | 9 | 9 | 9 | 36.5 | 36.5 | 36.5 | 34.352 | 71.297 | 306870000 | 15 | Aminoacyl tRNA synthase complex-interacting multifunctional protein 1;Endothelial monocyte-activating polypeptide 2 | AIMP1 |
| 23.36933 | 23.28912 | 23.61358 | 23.48603 | 10 | 10 | 10 | 21.7 | 21.7 | 21.7 | 61.448 | 68.172 | 277020000 | 17 | Phosphoglucomutase-1 | PGM1 |
| 24.94512 | 24.8723 | 23.61054 | 24.91018 | 28 | 28 | 1 | 22.6 | 22.6 | 0.6 | 158.52 | 323.31 | 1532500000 | 61 | Eukaryotic translation initiation factor 4 gamma 1 | EIF4G1 |
| 24.69243 | 23.31866 | 23.61009 | 23.59787 | 6 | 6 | 6 | 32.2 | 32.2 | 32.2 | 23.548 | 37.903 | 81315000 | 13 | Ras-related protein Rab-6A;Ras-related protein Rab-6B | RAB6A;RAB6B |
| 22.90193 | 23.31147 | 23.60987 | 24.20023 | 8 | 8 | 8 | 12.1 | 12.1 | 12.1 | 91.706 | 126.53 | 492390000 | 17 | Vacuolar protein sorting-associated protein 35 | VPS35 |
| 23.46712 | 23.22597 | 23.60558 | 24.16592 | 5 | 5 | 5 | 26.5 | 26.5 | 26.5 | 20.63 | 32.264 | 65667000 | 13 | 28 kDa heat- and acid-stable phosphoprotein | PDAP1 |
| 24.52552 | 23.95802 | 23.60298 | 24.49039 | 11 | 11 | 10 | 20.6 | 20.6 | 18.3 | 66.69 | 86.557 | 601680000 | 25 | CTP synthase 1 | CTPS1 |
| 24.35421 | 23.3982 | 23.59035 | 23.74201 | 9 | 9 | 8 | 29.4 | 29.4 | 25.9 | 38.044 | 127.38 | 537820000 | 18 | DnaJ homolog subfamily B member 1 | DNAJB1 |
| 24.60473 | 22.51557 | 23.58738 | 22.13223 | 9 | 9 | 9 | 31.5 | 31.5 | 31.5 | 54.861 | 196.76 | 689930000 | 16 | Target of Myb protein 1 | TOM1 |
| 23.90885 | 22.52571 | 23.58692 | 23.51605 | 12 | 10 | 10 | 26.6 | 25 | 25 | 61.64 | 100.21 | 433690000 | 23 | Far upstream element-binding protein 3 | FUBP3 |
| 22.6299 | 24.29684 | 23.58658 | 23.25407 | 13 | 13 | 13 | 15.3 | 15.3 | 15.3 | 117.8 | 119.16 | 602540000 | 21 | Superkiller viralicidic activity 2-like 2 | SKIV2L2 |
| 23.12734 | 24.12052 | 23.58635 | 24.0269 | 8 | 8 | 8 | 20.6 | 20.6 | 20.6 | 53.165 | 72.386 | 397990000 | 22 | Tryptophan--tRNA ligase, cytoplasmic;T1-TrpRS;T2-TrpRS | WARS |
| 26.07722 | 25.61852 | 23.57888 | 24.07944 | 39 | 39 | 39 | 34.6 | 34.6 | 34.6 | 141.45 | 323.31 | 1579800000 | 58 | Vigilin | HDLBP |
| 22.27093 | 23.33851 | 23.57808 | 18.18052 | 6 | 6 | 6 | 17.9 | 17.9 | 17.9 | 39.679 | 38.22 | 129970000 | 11 | Protein CYR61 | CYR61 |
| 24.56558 | 24.14748 | 23.573 | 25.697 | 21 | 21 | 21 | 35.1 | 35.1 | 35.1 | 82.285 | 216.57 | 1385000000 | 40 | DNA replication licensing factor MCM5 | MCM5 |
| 22.92332 | 23.52193 | 23.5566 | 23.88985 | 7 | 7 | 7 | 14.1 | 14.1 | 14.1 | 63.541 | 63.481 | 440500000 | 16 | Ran GTPase-activating protein 1 | RANGAP1 |
| 24.18033 | 23.01086 | 23.55051 | 23.2498 | 4 | 4 | 4 | 20.7 | 20.7 | 20.7 | 26.411 | 42.621 | 72490000 | 11 | Proteasome subunit alpha type-5 | PSMA5 |
| 23.17696 | 23.32182 | 23.54345 | 23.85852 | 8 | 8 | 8 | 11 | 11 | 11 | 115.96 | 114.17 | 433990000 | 16 | Importin-9 | IPO9 |
| 23.46799 | 23.29586 | 23.54215 | 24.01478 | 13 | 13 | 13 | 16.7 | 16.7 | 16.7 | 93.487 | 84.401 | 601470000 | 22 | Nuclear pore complex protein Nup93 | NUP93 |
| 22.92723 | 23.07593 | 23.53552 | 23.5956 | 5 | 5 | 4 | 26.6 | 26.6 | 23.4 | 24.579 | 30.635 | 54113000 | 10 | Hypoxanthine-guanine phosphoribosyltransferase | HPRT1 |
| 22.08102 | 23.15396 | 23.5354 | 23.00816 | 3 | 3 | 3 | 12.8 | 12.8 | 12.8 | 29.573 | 21.785 | 163080000 | 11 | Translocon-associated protein subunit alpha | SSR1 |
| 24.91825 | 23.47667 | 23.53481 | 24.59884 | 7 | 7 | 7 | 29 | 29 | 29 | 35.238 | 159.8 | 1245200000 | 21 | Heterogeneous nuclear ribonucleoprotein H3 | HNRNPH3 |
| 24.01623 | 22.794 | 23.52898 | 23.47976 | 8 | 8 | 8 | 26.4 | 26.4 | 26.4 | 43.066 | 56.644 | 565240000 | 15 | cAMP-dependent protein kinase type II-alpha regulatory subunit | PRKAR2A |
| 23.64823 | 23.80322 | 23.52444 | 24.22118 | 8 | 8 | 8 | 22.3 | 22.3 | 22.3 | 49.222 | 61.192 | 376440000 | 13 | Eukaryotic translation initiation factor 5 | EIF5 |
| 22.80031 | 21.60589 | 23.52432 | 22.92401 | 3 | 3 | 3 | 16 | 16 | 16 | 23.277 | 21.618 | 39249000 | 11 | ATP synthase subunit O, mitochondrial | ATP5O |
| 22.88241 | 22.96619 | 23.52325 | 23.34652 | 8 | 8 | 8 | 17.1 | 17.1 | 17.1 | 60.284 | 52.513 | 265080000 | 13 | Signal recognition particle subunit SRP68 | SRP68 |
| 22.13759 | 23.11375 | 23.51689 | 23.38653 | 9 | 9 | 9 | 19.4 | 19.4 | 19.4 | 45.348 | 53.993 | 157400000 | 20 | Dimethyladenosine transferase 2, mitochondrial | TFB2M |
| 22.77072 | 23.25969 | 23.5122 | 23.43148 | 9 | 9 | 9 | 19.5 | 19.5 | 19.5 | 60.966 | 62.79 | 289210000 | 16 | Prolyl 4-hydroxylase subunit alpha-1 | P4HA1 |
| 22.72509 | 23.24947 | 23.51196 | 23.36586 | 7 | 7 | 7 | 16.5 | 16.5 | 16.5 | 46.971 | 62.477 | 219780000 | 20 | Endoplasmic reticulum resident protein 44 | ERP44 |
| 23.22059 | 23.32594 | 23.51159 | 23.86486 | 3 | 3 | 3 | 21.7 | 21.7 | 21.7 | 22.34 | 20.293 | 81850000 | 6 | Coatomer subunit zeta-1 | COPZ1 |
| 23.0293 | 23.0268 | 23.5087 | 22.78766 | 7 | 7 | 7 | 27.2 | 27.2 | 27.2 | 42.5 | 63.743 | 296060000 | 17 | Armadillo repeat-containing X-linked protein 3 | ARMCX3 |
| 17.66582 | 18.95043 | 23.50797 | 20.6497 | 5 | 5 | 5 | 26.8 | 26.8 | 26.8 | 22.255 | 63.48 | 74517000 | 6 | Dual specificity protein phosphatase 14 | DUSP14 |
| 22.74109 | 21.84479 | 23.50531 | 23.30271 | 5 | 5 | 5 | 12.4 | 12.4 | 12.4 | 49.364 | 29.772 | 82151000 | 23 | Gasdermin-A | GSDMA |
| 23.00352 | 23.57715 | 23.50179 | 24.29474 | 16 | 5 | 5 | 43.4 | 16.8 | 16.8 | 50.47 | 87.834 | 648800000 | 14 | Elongation factor 1-alpha 2 | EEF1A2 |
| 22.51429 | 24.53742 | 23.49838 | 23.45098 | 7 | 7 | 7 | 18.3 | 18.3 | 18.3 | 51.466 | 103.19 | 320590000 | 20 | Luc7-like protein 3 | LUC7L3 |
| 24.77706 | 22.70847 | 23.49558 | 24.00881 | 9 | 7 | 7 | 39.9 | 33.9 | 33.9 | 32.949 | 151.34 | 807190000 | 21 | F-actin-capping protein subunit alpha-2 | CAPZA2 |
| 23.26714 | 24.826 | 23.48308 | 23.48517 | 10 | 10 | 10 | 24.8 | 24.8 | 24.8 | 53.126 | 87.547 | 427170000 | 20 | G-rich sequence factor 1 | GRSF1 |
| 21.0036 | 22.96674 | 23.48185 | 22.39024 | 7 | 7 | 7 | 18.6 | 18.6 | 18.6 | 41.268 | 54.209 | 105120000 | 13 | Spermine synthase | SMS |
| 22.73684 | 22.66642 | 23.48173 | 19.87957 | 3 | 3 | 3 | 11.6 | 11.6 | 11.6 | 34.577 | 26.651 | 225080000 | 11 | 26S proteasome non-ATPase regulatory subunit 14 | PSMD14 |
| 22.36837 | 23.36199 | 23.4768 | 21.32603 | 8 | 8 | 8 | 49.8 | 49.8 | 49.8 | 26.922 | 92.819 | 225070000 | 17 | Chloride intracellular channel protein 1 | CLIC1 |
| 24.39676 | 23.31755 | 23.46301 | 24.45808 | 16 | 16 | 16 | 21.8 | 21.8 | 21.8 | 103.1 | 224.89 | 1053900000 | 24 | Lon protease homolog, mitochondrial | LONP1 |
| 25.55532 | 24.90816 | 23.46264 | 24.71759 | 33 | 33 | 33 | 31.3 | 31.3 | 31.3 | 141.54 | 322.53 | 1085200000 | 53 | Structural maintenance of chromosomes protein 3 | SMC3 |
| 22.36928 | 22.74263 | 23.45111 | 19.01368 | 4 | 4 | 4 | 21.8 | 21.8 | 21.8 | 27.994 | 32.075 | 139120000 | 16 | Nucleolar protein of 40 kDa | ZCCHC17 |
| 22.45719 | 22.71802 | 23.44935 | 22.8565 | 10 | 10 | 10 | 24.9 | 24.9 | 24.9 | 45.098 | 67.712 | 240030000 | 14 | 2,3-cyclic-nucleotide 3-phosphodiesterase | CNP |
| 23.71135 | 23.31852 | 23.44733 | 23.93094 | 8 | 8 | 8 | 27.4 | 27.4 | 27.4 | 36.418 | 55.041 | 326050000 | 17 | Ubiquitin carboxyl-terminal hydrolase isozyme L5 | UCHL5 |
| 23.3868 | 22.83842 | 23.44733 | 20.39666 | 14 | 7 | 7 | 53.7 | 32.6 | 32.6 | 32.866 | 239.61 | 360990000 | 10 | ADP/ATP translocase 3;ADP/ATP translocase 3, N-terminally processed;ADP/ATP translocase 1 | SLC25A6;SLC25A4 |
| 22.65161 | 23.09333 | 23.44354 | 19.58679 | 4 | 4 | 4 | 19.4 | 19.4 | 19.4 | 36.587 | 30.898 | 206540000 | 11 | Vacuolar protein sorting-associated protein 26A | VPS26A |
| 24.8739 | 25.18033 | 23.4367 | 23.42406 | 15 | 15 | 15 | 24.5 | 24.5 | 24.5 | 101.43 | 255.73 | 1034100000 | 40 | Zinc finger CCCH-type antiviral protein 1 | ZC3HAV1 |
| 23.6826 | 23.67508 | 23.43301 | 23.66014 | 5 | 5 | 5 | 28.3 | 28.3 | 28.3 | 27.191 | 54.239 | 517030000 | 12 | Heterogeneous nuclear ribonucleoprotein D-like | HNRNPDL |
| 17.97616 | 22.62596 | 23.42905 | 23.36786 | 4 | 4 | 4 | 12.2 | 12.2 | 12.2 | 45.972 | 29.935 | 100220000 | 10 | NAD kinase 2, mitochondrial | NADK2 |
| 23.99326 | 24.6274 | 23.42445 | 24.49283 | 12 | 12 | 12 | 16.3 | 16.3 | 16.3 | 85.104 | 108.33 | 913670000 | 21 | Heterogeneous nuclear ribonucleoprotein U-like protein 2 | HNRNPUL2;HNRNPUL2-BSCL2 |
| 21.98652 | 23.14908 | 23.42034 | 23.89013 | 9 | 9 | 9 | 18.7 | 18.7 | 18.7 | 72.911 | 66.48 | 244790000 | 17 | Alkyldihydroxyacetonephosphate synthase, peroxisomal | AGPS |
| 22.29894 | 21.88118 | 23.41545 | 22.58197 | 4 | 4 | 4 | 6.2 | 6.2 | 6.2 | 89.786 | 26.195 | 86593000 | 15 | Protein-glutamine gamma-glutamyltransferase K | TGM1 |
| 22.06178 | 22.66607 | 23.41222 | 23.01737 | 8 | 8 | 8 | 30.7 | 30.7 | 30.7 | 29.717 | 54.144 | 192850000 | 15 | LIM and SH3 domain protein 1 | LASP1 |
| 22.40974 | 23.54156 | 23.41222 | 22.35534 | 6 | 6 | 6 | 24.9 | 24.9 | 24.9 | 35.286 | 61.746 | 166610000 | 10 | Cysteine and histidine-rich domain-containing protein 1 | CHORDC1 |
| 24.15012 | 26.65198 | 23.41041 | 22.29412 | 22 | 22 | 22 | 31.9 | 31.9 | 31.9 | 89.301 | 293.68 | 1687300000 | 44 | Probable 28S rRNA (cytosine(4447)-C(5))-methyltransferase | NOP2 |
| 24.67298 | 24.43129 | 23.40834 | 18.07903 | 7 | 7 | 7 | 29 | 29 | 29 | 25.476 | 71.147 | 769400000 | 35 | Serine/arginine-rich splicing factor 2 | SRSF2 |
| 21.6657 | 23.22363 | 23.40302 | 22.73399 | 8 | 8 | 8 | 23.8 | 23.8 | 23.8 | 41.465 | 57.727 | 144200000 | 12 | Alpha-2-macroglobulin receptor-associated protein | LRPAP1 |
| 22.45571 | 23.42124 | 23.40237 | 23.51965 | 9 | 9 | 9 | 34.5 | 34.5 | 34.5 | 38.646 | 123.71 | 204440000 | 22 | DNA-directed RNA polymerases I and III subunit RPAC1 | POLR1C |
| 21.8771 | 23.33115 | 23.39793 | 23.63954 | 9 | 9 | 9 | 5.9 | 5.9 | 5.9 | 186.49 | 56.263 | 340280000 | 20 | Protein transport protein Sec16A | SEC16A |
| 23.83779 | 17.41955 | 23.39663 | 25.23874 | 4 | 4 | 4 | 22.1 | 22.1 | 22.1 | 23.207 | 31.121 | 152770000 | 13 | Rho GDP-dissociation inhibitor 1 | ARHGDIA |
| 22.36383 | 23.89865 | 23.39571 | 24.99412 | 13 | 13 | 13 | 30 | 30 | 30 | 69.284 | 119.07 | 387540000 | 22 | Leukotriene A-4 hydrolase | LTA4H |
| 21.86661 | 22.84558 | 23.39113 | 22.46538 | 7 | 7 | 7 | 19.4 | 19.4 | 19.4 | 44.743 | 48.7 | 122000000 | 14 | Obg-like ATPase 1 | OLA1 |
| 22.37666 | 23.79699 | 23.38232 | 24.16791 | 8 | 8 | 8 | 20.4 | 20.4 | 20.4 | 49.973 | 66.255 | 280770000 | 17 | Ribonuclease inhibitor | RNH1 |
| 22.35558 | 23.29838 | 23.38179 | 23.90251 | 11 | 11 | 11 | 35 | 35 | 35 | 44.809 | 78.578 | 248710000 | 17 | Dynactin subunit 2 | DCTN2 |
| 22.81623 | 22.6548 | 23.37901 | 23.44872 | 8 | 8 | 8 | 22.2 | 22.2 | 22.2 | 46.51 | 63.609 | 281170000 | 15 | Succinyl-CoA ligase [GDP-forming] subunit beta, mitochondrial | SUCLG2 |
| 24.17972 | 23.80272 | 23.37584 | 24.08659 | 10 | 10 | 10 | 26.9 | 26.9 | 26.9 | 51.311 | 93.648 | 526400000 | 23 | 26S proteasome non-ATPase regulatory subunit 5 | PSMD5 |
| 22.92579 | 23.30994 | 23.37 | 23.39872 | 7 | 7 | 7 | 11.7 | 11.7 | 11.7 | 92.721 | 69.472 | 436290000 | 12 | Catenin alpha-1 | CTNNA1 |
| 22.5904 | 24.49033 | 23.36399 | 20.05364 | 7 | 7 | 7 | 12.2 | 12.2 | 12.2 | 83.654 | 67.498 | 381560000 | 7 | Nucleolar GTP-binding protein 2 | GNL2 |
| 22.47484 | 22.76673 | 23.36319 | 24.14328 | 7 | 7 | 7 | 17.3 | 17.3 | 17.3 | 45.283 | 44.773 | 291020000 | 18 | LanC-like protein 1 | LANCL1 |
| 23.19429 | 24.63633 | 23.36306 | 22.31241 | 17 | 17 | 17 | 18.2 | 18.2 | 18.2 | 139.76 | 125.63 | 324760000 | 23 | Tripeptidyl-peptidase 2 | TPP2 |
| 22.73892 | 22.81004 | 23.35743 | 23.48395 | 10 | 10 | 10 | 31.9 | 31.9 | 31.9 | 58.14 | 85.875 | 277760000 | 14 | Eukaryotic translation initiation factor 3 subunit D | EIF3D |
| 18.93761 | 27.74849 | 23.35272 | 22.5062 | 8 | 8 | 8 | 25.7 | 25.7 | 25.7 | 21.022 | 87.822 | 1202300000 | 48 | Syndecan-2;Syndecan | SDC2 |
| 25.30219 | 23.68035 | 23.35084 | 21.3197 | 8 | 8 | 7 | 34.4 | 34.4 | 31.6 | 33.665 | 112.97 | 777370000 | 18 | Transformer-2 protein homolog beta | TRA2B |
| 22.64104 | 24.71639 | 23.35043 | 24.57548 | 6 | 6 | 6 | 49 | 49 | 49 | 17.04 | 43.822 | 117400000 | 15 | 40S ribosomal protein S15 | RPS15 |
| 21.41282 | 23.17245 | 23.34638 | 18.08142 | 6 | 6 | 6 | 33.5 | 33.5 | 33.5 | 24.583 | 48.292 | 175870000 | 12 | Proteasome subunit alpha type;Proteasome subunit alpha type-4;Proteasome subunit beta type | PSMA4 |
| 23.78686 | 20.79265 | 23.34597 | 23.81783 | 4 | 4 | 4 | 14.7 | 14.7 | 14.7 | 48.227 | 56.383 | 401230000 | 12 | KH domain-containing, RNA-binding, signal transduction-associated protein 1 | KHDRBS1 |
| 25.43262 | 24.69896 | 23.34042 | 23.72754 | 15 | 15 | 13 | 30.7 | 30.7 | 26.8 | 63.704 | 122.21 | 1050600000 | 28 | Insulin-like growth factor 2 mRNA-binding protein 3 | IGF2BP3 |
| 23.15593 | 23.49228 | 23.3332 | 23.69636 | 7 | 7 | 7 | 15 | 15 | 15 | 48.039 | 43.448 | 295900000 | 12 | Succinyl-CoA ligase [ADP-forming] subunit beta, mitochondrial | SUCLA2 |
| 23.57589 | 24.72562 | 23.33183 | 22.30293 | 12 | 12 | 12 | 46.4 | 46.4 | 46.4 | 36.104 | 99.45 | 456700000 | 25 | Replication factor C subunit 5 | RFC5 |
| 22.33985 | 22.36592 | 23.33101 | 22.52576 | 5 | 5 | 5 | 19.4 | 19.4 | 19.4 | 38.989 | 36.324 | 189000000 | 11 | Translation initiation factor eIF-2B subunit beta | EIF2B2 |
| 21.40109 | 22.82479 | 23.33033 | 23.21592 | 8 | 8 | 8 | 17.1 | 17.1 | 17.1 | 55.676 | 57.023 | 163080000 | 16 | UTP--glucose-1-phosphate uridylyltransferase | UGP2 |
| 23.0941 | 23.93975 | 23.32992 | 23.56349 | 8 | 8 | 8 | 29.3 | 29.3 | 29.3 | 36.426 | 74.19 | 297720000 | 26 | Malate dehydrogenase, cytoplasmic;Malate dehydrogenase | MDH1 |
| 23.56395 | 23.41377 | 23.32882 | 22.8358 | 8 | 8 | 8 | 21.2 | 21.2 | 21.2 | 45.935 | 64.778 | 304880000 | 21 | Transducin beta-like protein 2 | TBL2 |
| 24.60535 | 25.95318 | 23.32869 | 23.60071 | 12 | 12 | 12 | 35.4 | 35.4 | 35.4 | 49.419 | 165.28 | 845890000 | 23 | Glutamate-rich WD repeat-containing protein 1 | GRWD1 |
| 24.75389 | 23.0798 | 23.31382 | 23.20361 | 7 | 5 | 4 | 32.4 | 23.7 | 19.8 | 23.668 | 48.795 | 91795000 | 11 | Ras-related protein Rab-8A | RAB8A |
| 22.43934 | 22.12647 | 23.31161 | 19.64677 | 5 | 5 | 5 | 31.6 | 31.6 | 31.6 | 24.763 | 60.589 | 189120000 | 9 | Elongation factor 1-beta | EEF1B2 |
| 22.10856 | 22.50176 | 23.3041 | 23.99395 | 8 | 8 | 8 | 23.5 | 23.5 | 23.5 | 55.096 | 76.262 | 177010000 | 14 | Serine/threonine-protein phosphatase;Serine/threonine-protein phosphatase 5 | PPP5C |
| 23.72796 | 22.33593 | 23.30396 | 23.07169 | 5 | 5 | 5 | 25.5 | 25.5 | 25.5 | 32.611 | 88.515 | 500800000 | 10 | UBX domain-containing protein 1 | UBXN1 |
| 23.50373 | 23.2195 | 23.29726 | 24.30375 | 11 | 11 | 11 | 13 | 13 | 13 | 104.74 | 91.513 | 645620000 | 23 | DNA mismatch repair protein Msh2 | MSH2 |
| 22.30758 | 23.54804 | 23.29628 | 22.78333 | 8 | 8 | 8 | 26 | 26 | 26 | 41.329 | 56.515 | 131780000 | 17 | 28S ribosomal protein S27, mitochondrial | MRPS27 |
| 24.20284 | 22.66997 | 23.28785 | 23.15429 | 11 | 11 | 11 | 64 | 64 | 64 | 28.768 | 212.18 | 525460000 | 27 | Proliferating cell nuclear antigen | PCNA |
| 16.47418 | 19.21651 | 23.28221 | 17.37221 | 14 | 1 | 1 | 56.6 | 3.7 | 3.7 | 27.85 | 7.7142 | 92381000 | 9 | 14-3-3 protein beta/alpha;14-3-3 protein beta/alpha, N-terminally processed | YWHAB |
| 22.35955 | 23.06686 | 23.2737 | 24.79417 | 4 | 4 | 4 | 8 | 8 | 8 | 64.149 | 26.147 | 215420000 | 10 | NADP-dependent malic enzyme | ME1 |
| 24.02657 | 22.49953 | 23.26828 | 18.16602 | 4 | 4 | 4 | 13.4 | 13.4 | 13.4 | 46.637 | 47.273 | 380900000 | 9 | Platelet-activating factor acetylhydrolase IB subunit alpha | PAFAH1B1 |
| 23.38535 | 22.54626 | 23.26685 | 21.05715 | 6 | 6 | 6 | 13.4 | 13.4 | 13.4 | 58.86 | 52.357 | 407360000 | 11 | Disabled homolog 2 | DAB2 |
| 23.13585 | 22.46531 | 23.2558 | 22.7387 | 9 | 9 | 9 | 18.4 | 18.4 | 18.4 | 57.21 | 56.421 | 187570000 | 17 | Coatomer subunit delta | ARCN1 |
| 24.43154 | 24.85253 | 23.25523 | 23.47581 | 12 | 12 | 12 | 52.5 | 52.5 | 52.5 | 30.84 | 176.27 | 860100000 | 36 | Heterogeneous nuclear ribonucleoprotein A0 | HNRNPA0 |
| 20.75385 | 23.96235 | 23.25494 | 23.77012 | 10 | 10 | 10 | 6 | 6 | 6 | 200.48 | 73.535 | 248780000 | 17 | Laminin subunit beta-1 | LAMB1 |
| 24.30647 | 24.88879 | 23.25309 | 24.49631 | 30 | 30 | 30 | 21.8 | 21.8 | 21.8 | 204.29 | 226.6 | 583420000 | 48 | Proteasome-associated protein ECM29 homolog | ECM29 |
| 24.21734 | 23.12379 | 23.25171 | 21.80085 | 17 | 15 | 14 | 28.1 | 25.8 | 24.6 | 85.182 | 217.29 | 733920000 | 20 | ATP-dependent 6-phosphofructokinase, muscle type | PFKM |
| 20.72276 | 23.66861 | 23.24432 | 23.24892 | 17 | 17 | 17 | 7.7 | 7.7 | 7.7 | 274.37 | 117.58 | 353640000 | 24 | Cation-independent mannose-6-phosphate receptor | IGF2R |
| 23.70493 | 24.6833 | 23.24258 | 22.11579 | 17 | 17 | 17 | 31.1 | 31.1 | 31.1 | 89.034 | 272.81 | 798210000 | 26 | Transducin beta-like protein 3 | TBL3 |
| 22.60637 | 17.82874 | 23.24255 | 22.09758 | 5 | 5 | 5 | 16.6 | 16.6 | 16.6 | 55.832 | 94.258 | 312100000 | 6 | Epsin-1 | EPN1 |
| 24.6725 | 23.16219 | 23.23764 | 22.23596 | 15 | 15 | 15 | 24.1 | 24.1 | 24.1 | 82.431 | 122.66 | 858740000 | 20 | ATP-dependent RNA helicase DDX1 | DDX1 |
| 23.77826 | 22.77388 | 23.23033 | 22.46441 | 13 | 8 | 8 | 44.1 | 30.1 | 30.1 | 49.263 | 98.369 | 427840000 | 19 | Heterogeneous nuclear ribonucleoprotein H2;Heterogeneous nuclear ribonucleoprotein H2, N-terminally processed | HNRNPH2 |
| 23.30048 | 22.77756 | 23.22935 | 23.27469 | 10 | 10 | 10 | 28.5 | 28.5 | 28.5 | 47.346 | 86.442 | 323620000 | 16 | Mitochondrial ribonuclease P protein 1 | TRMT10C |
| 24.40996 | 22.87513 | 23.22828 | 23.16065 | 8 | 8 | 6 | 25 | 25 | 18.4 | 43.867 | 60.529 | 454200000 | 15 | Serine/threonine-protein kinase MST4 | RBM4B;MST4 |
| 23.59787 | 24.01929 | 23.21543 | 24.12605 | 6 | 6 | 6 | 38.2 | 38.2 | 38.2 | 16.46 | 40.825 | 91895000 | 17 | Eukaryotic translation initiation factor 1A, X-chromosomal;Eukaryotic translation initiation factor 1A, Y-chromosomal | EIF1AX;EIF1AY |
| 22.35552 | 21.84529 | 23.21383 | 22.47189 | 6 | 6 | 6 | 19.4 | 19.4 | 19.4 | 44.552 | 35.204 | 73230000 | 14 | Cathepsin D;Cathepsin D light chain;Cathepsin D heavy chain | CTSD |
| 21.80195 | 22.70052 | 23.21079 | 23.91907 | 5 | 5 | 5 | 16.4 | 16.4 | 16.4 | 40.949 | 42.445 | 187700000 | 13 | Actin-related protein 2/3 complex subunit 1B | ARPC1B |
| 24.58698 | 23.82512 | 23.21064 | 19.95899 | 12 | 12 | 11 | 43.1 | 43.1 | 40.4 | 34.095 | 118.82 | 699960000 | 22 | Cyclin-dependent kinase 1 | CDK1 |
| 26.14723 | 22.88189 | 23.19847 | 21.8811 | 5 | 5 | 5 | 13.6 | 13.6 | 13.6 | 49.255 | 53.813 | 1551800000 | 16 | Splicing factor 3A subunit 2 | SF3A2 |
| 25.11227 | 24.04377 | 23.19514 | 22.58206 | 14 | 14 | 14 | 65.7 | 65.7 | 65.7 | 30.772 | 323.31 | 769710000 | 30 | Voltage-dependent anion-selective channel protein 1 | VDAC1 |
| 22.42114 | 22.2641 | 23.18595 | 23.30187 | 4 | 4 | 4 | 24.2 | 24.2 | 24.2 | 24.976 | 27.926 | 46833000 | 10 | Transmembrane emp24 domain-containing protein 10 | TMED10 |
| 24.71853 | 26.3028 | 23.18489 | 23.15085 | 30 | 30 | 30 | 25.8 | 25.8 | 25.8 | 148.85 | 298.18 | 844340000 | 57 | Myb-binding protein 1A | MYBBP1A |
| 22.77276 | 23.02049 | 23.18056 | 23.00249 | 4 | 4 | 4 | 20.5 | 20.5 | 20.5 | 22.275 | 25.398 | 45636000 | 12 | ATP synthase F(0) complex subunit B1, mitochondrial | ATP5F1 |
| 20.41039 | 22.02137 | 23.16927 | 23.57554 | 7 | 7 | 7 | 16.7 | 16.7 | 16.7 | 52.562 | 42.383 | 119960000 | 9 | Bleomycin hydrolase | BLMH |
| 22.83936 | 23.26356 | 23.16512 | 23.52061 | 13 | 13 | 11 | 17 | 17 | 15 | 98.972 | 84.597 | 367830000 | 17 | Hexokinase-2 | HK2 |
| 23.2798 | 22.857 | 23.16306 | 23.63001 | 9 | 9 | 6 | 12.5 | 12.5 | 8.3 | 87.679 | 81.258 | 472750000 | 16 | Cullin-4A | CUL4A |
| 21.57944 | 22.28136 | 23.15999 | 23.27099 | 5 | 5 | 5 | 22.5 | 22.5 | 22.5 | 35.348 | 33.308 | 151840000 | 14 | Aminoacyl tRNA synthase complex-interacting multifunctional protein 2 | AIMP2 |
| 23.39074 | 23.45689 | 23.15201 | 24.10254 | 10 | 10 | 9 | 19.6 | 19.6 | 18.3 | 68.988 | 107.32 | 497290000 | 16 | Prolyl 3-hydroxylase 1 | LEPRE1 |
| 24.22295 | 24.83432 | 23.14218 | 23.41957 | 21 | 21 | 21 | 20.2 | 20.2 | 20.2 | 130.55 | 174.38 | 627110000 | 33 | Putative ATP-dependent RNA helicase DHX30 | DHX30 |
| 22.18423 | 22.44731 | 23.14115 | 19.00103 | 4 | 4 | 4 | 19.3 | 19.3 | 19.3 | 34.583 | 46.581 | 131330000 | 9 | Methionine adenosyltransferase 2 subunit beta | MAT2B |
| 25.75936 | 23.92125 | 23.14093 | 19.14695 | 8 | 7 | 7 | 29.8 | 29.8 | 29.8 | 26.928 | 77.425 | 1223100000 | 22 | Serine/arginine-rich splicing factor 7 | SRSF7 |
| 23.32649 | 23.23137 | 23.13245 | 23.18841 | 9 | 9 | 9 | 26 | 26 | 26 | 47.707 | 62.037 | 409420000 | 12 | Ribosome biogenesis protein WDR12 | WDR12 |
| 22.21205 | 22.06131 | 23.13113 | 22.79555 | 6 | 6 | 6 | 19 | 19 | 19 | 41.489 | 40.229 | 119340000 | 11 | Endophilin-A2 | SH3GL1 |
| 22.68579 | 22.71232 | 23.12291 | 20.35709 | 7 | 7 | 7 | 35.3 | 35.3 | 35.3 | 31.628 | 63.733 | 234440000 | 14 | NADH-cytochrome b5 reductase 3;NADH-cytochrome b5 reductase 3 membrane-bound form;NADH-cytochrome b5 reductase 3 soluble form | CYB5R3 |
| 21.02634 | 23.08803 | 23.12163 | 22.50106 | 9 | 9 | 9 | 10.4 | 10.4 | 10.4 | 90.954 | 57.499 | 218500000 | 11 | Cullin-5 | CUL5 |
| 23.00787 | 23.67508 | 23.11755 | 23.64592 | 6 | 6 | 6 | 20.3 | 20.3 | 20.3 | 34.756 | 155.51 | 250900000 | 12 | Replication factor C subunit 3 | RFC3 |
| 26.62143 | 23.12334 | 23.11521 | 23.41867 | 6 | 6 | 5 | 35 | 35 | 26.2 | 20.811 | 50.923 | 253200000 | 17 | Chromobox protein homolog 3 | CBX3 |
| 24.34949 | 23.20571 | 23.11413 | 23.39074 | 5 | 3 | 3 | 22 | 13 | 13 | 22.541 | 18.707 | 76359000 | 9 | Ras-related protein Rab-10 | RAB10 |
| 23.39126 | 24.59923 | 23.10913 | 24.43422 | 8 | 8 | 7 | 28.1 | 28.1 | 23.6 | 40.736 | 154.52 | 435220000 | 19 | 26S proteasome non-ATPase regulatory subunit 4 | PSMD4 |
| 22.57274 | 22.76739 | 23.10444 | 23.07795 | 9 | 9 | 9 | 15 | 15 | 15 | 83.111 | 78.081 | 325770000 | 11 | Calpain-2 catalytic subunit | CAPN2 |
| 23.12054 | 22.98085 | 23.0993 | 23.99404 | 11 | 11 | 11 | 16.1 | 16.1 | 16.1 | 101.49 | 98.437 | 485280000 | 16 | Importin-4 | IPO4 |
| 18.64536 | 23.08558 | 23.09883 | 22.0806 | 5 | 5 | 4 | 11.6 | 11.6 | 8.5 | 53.166 | 32.22 | 98788000 | 6 | Thioredoxin reductase 1, cytoplasmic | GML;TXNRD1 |
| 22.76933 | 23.98198 | 23.09856 | 23.78397 | 8 | 8 | 8 | 18.5 | 18.5 | 18.5 | 63.472 | 84.053 | 288800000 | 14 | Leucine-rich repeat-containing protein 47 | LRRC47 |
| 21.70974 | 22.794 | 23.09835 | 23.88818 | 25 | 25 | 25 | 7.9 | 7.9 | 7.9 | 480.19 | 253.15 | 614570000 | 26 | E3 ubiquitin-protein ligase HUWE1 | HUWE1 |
| 23.9943 | 22.94942 | 23.09552 | 23.45826 | 10 | 10 | 10 | 27.8 | 27.8 | 27.8 | 53.488 | 184.04 | 443380000 | 18 | Ataxin-10 | ATXN10 |
| 22.89643 | 22.86378 | 23.08252 | 20.53393 | 6 | 5 | 5 | 18.6 | 16.6 | 16.6 | 45.809 | 54.174 | 262430000 | 6 | Aurora kinase A | AURKA |
| 22.43143 | 23.39585 | 23.07858 | 22.86155 | 7 | 7 | 7 | 13.1 | 13.1 | 13.1 | 76.689 | 60.159 | 386600000 | 14 | NADPH--cytochrome P450 reductase | POR |
| 23.10462 | 22.90292 | 23.06833 | 22.5689 | 5 | 5 | 5 | 14.8 | 14.8 | 14.8 | 46.036 | 39.098 | 230390000 | 11 | PCI domain-containing protein 2 | PCID2 |
| 23.48001 | 22.04096 | 23.06771 | 21.54444 | 7 | 7 | 7 | 28.9 | 28.9 | 28.9 | 23.662 | 99.829 | 314340000 | 12 | Clathrin light chain A | CLTA |
| 23.08413 | 22.40826 | 23.06538 | 23.75175 | 5 | 5 | 5 | 16.7 | 16.7 | 16.7 | 27.991 | 27.827 | 43386000 | 11 | B-cell receptor-associated protein 31 | BCAP31 |
| 17.91464 | 22.30304 | 23.0621 | 19.84885 | 4 | 4 | 4 | 14.1 | 14.1 | 14.1 | 34.084 | 35.712 | 106130000 | 8 | Adenosine kinase | ADK |
| 21.91254 | 23.43682 | 23.04247 | 23.93094 | 11 | 11 | 11 | 13.9 | 13.9 | 13.9 | 98.428 | 71.735 | 337710000 | 13 | MAP7 domain-containing protein 3 | MAP7D3 |
| 22.48653 | 21.84969 | 23.04178 | 22.16322 | 5 | 5 | 5 | 19.6 | 19.6 | 19.6 | 48.127 | 46.32 | 163790000 | 11 | Glutaryl-CoA dehydrogenase, mitochondrial | GCDH |
| 18.8176 | 20.46832 | 23.03403 | 21.0461 | 3 | 3 | 3 | 13.5 | 13.5 | 13.5 | 24.158 | 17.119 | 30812000 | 3 | Apolipoprotein D | APOD |
| 23.97885 | 22.8131 | 23.03219 | 18.83027 | 7 | 7 | 7 | 18.9 | 18.9 | 18.9 | 42.272 | 51.268 | 321330000 | 13 | Galactokinase | GALK1 |
| 22.78479 | 22.33979 | 23.02894 | 19.26086 | 8 | 8 | 8 | 11.8 | 11.8 | 11.8 | 106.37 | 91.209 | 348450000 | 8 | Nuclear pore complex protein Nup107 | NUP107 |
| 22.36944 | 21.25949 | 23.02663 | 21.99207 | 3 | 3 | 3 | 16.4 | 16.4 | 16.4 | 22.836 | 19.117 | 26613000 | 8 | Proteasome subunit beta type-2 | PSMB2 |
| 21.5435 | 22.10441 | 23.02508 | 22.60526 | 5 | 5 | 5 | 15.3 | 15.3 | 15.3 | 43.588 | 32.09 | 195620000 | 8 | Cat eye syndrome critical region protein 5 | CECR5 |
| 22.20389 | 22.77388 | 23.02437 | 22.30037 | 4 | 4 | 4 | 11.9 | 11.9 | 11.9 | 42.183 | 26.332 | 159860000 | 9 | Isocitrate dehydrogenase [NAD] subunit beta, mitochondrial | IDH3B |
| 20.85681 | 23.00738 | 23.01951 | 23.42008 | 4 | 4 | 4 | 15.5 | 15.5 | 15.5 | 42.981 | 45.895 | 173050000 | 13 | cAMP-dependent protein kinase type I-alpha regulatory subunit;cAMP-dependent protein kinase type I-alpha regulatory subunit, N-terminally processed | PRKAR1A |
| 21.52009 | 22.58321 | 23.01876 | 24.79094 | 7 | 7 | 7 | 32.7 | 32.7 | 32.7 | 28.804 | 59.643 | 148420000 | 19 | Phosphoglycerate mutase 1;Phosphoglycerate mutase 2 | PGAM1;PGAM2 |
| 22.60619 | 23.91771 | 23.01737 | 18.98852 | 6 | 5 | 5 | 23.8 | 20.4 | 20.4 | 30.858 | 33.184 | 195610000 | 10 | Serine/arginine-rich splicing factor 5 | SRSF5 |
| 21.54948 | 21.72159 | 23.01567 | 22.78808 | 4 | 4 | 3 | 9.1 | 9.1 | 7.5 | 57.206 | 26.506 | 136370000 | 8 | Aldehyde dehydrogenase X, mitochondrial | ALDH1B1 |
| 18.29006 | 22.60187 | 23.00967 | 22.94194 | 4 | 4 | 4 | 21.6 | 21.6 | 21.6 | 24.058 | 28.661 | 83962000 | 9 | UPF0160 protein MYG1, mitochondrial | C12orf10 |
| 25.83473 | 24.83726 | 23.00943 | 24.90265 | 11 | 11 | 9 | 32.2 | 32.2 | 28.1 | 52.164 | 151.78 | 1373500000 | 33 | Ras GTPase-activating protein-binding protein 1 | G3BP1 |
| 22.51492 | 22.62743 | 23.00383 | 22.64121 | 8 | 8 | 8 | 20.2 | 20.2 | 20.2 | 54.794 | 60.443 | 238540000 | 10 | Heat shock 70 kDa protein 14 | HSPA14 |
| 22.73845 | 22.45759 | 22.99699 | 23.05914 | 22 | 6 | 6 | 25.5 | 8.3 | 8.3 | 101.35 | 44.589 | 316770000 | 13 | AP-1 complex subunit beta-1 | AP1B1 |
| 21.93431 | 22.93691 | 22.99042 | 23.39963 | 7 | 7 | 7 | 10.8 | 10.8 | 10.8 | 82.682 | 53.243 | 327950000 | 13 | Niban-like protein 1 | FAM129B |
| 23.74837 | 22.76444 | 22.98997 | 22.58206 | 7 | 7 | 7 | 10.1 | 10.1 | 10.1 | 113.5 | 54.796 | 458720000 | 15 | Ubiquitin-associated protein 2-like | UBAP2L |
| 23.92877 | 22.94726 | 22.98912 | 23.94798 | 19 | 19 | 19 | 22.7 | 22.7 | 22.7 | 123.03 | 244.72 | 782550000 | 25 | Regulator of nonsense transcripts 1 | UPF1 |
| 24.79407 | 22.43545 | 22.98843 | 19.42155 | 8 | 8 | 8 | 16.6 | 16.6 | 16.6 | 68.259 | 79.554 | 685680000 | 12 | Clathrin interactor 1 | CLINT1 |
| 24.72036 | 20.85855 | 22.98789 | 20.96084 | 6 | 6 | 6 | 19 | 19 | 19 | 50.513 | 55.564 | 586000000 | 13 | Carboxypeptidase M | CPM |
| 23.97316 | 24.44335 | 22.98567 | 24.4789 | 24 | 24 | 24 | 26 | 26 | 26 | 139.09 | 249.94 | 488150000 | 34 | Dynactin subunit 1 | DCTN1;DKFZp686E0752 |
| 23.17892 | 22.30254 | 22.98331 | 22.18631 | 6 | 6 | 6 | 29.6 | 29.6 | 29.6 | 22.977 | 35.094 | 39461000 | 9 | Ras-related protein Rab-18 | RAB18 |
| 22.82904 | 22.56477 | 22.97116 | 23.28644 | 7 | 4 | 4 | 22.8 | 13.9 | 13.9 | 38.925 | 29.42 | 107590000 | 9 | Erlin-1 | ERLIN1 |
| 20.19039 | 21.87864 | 22.96904 | 23.34394 | 14 | 14 | 14 | 8.2 | 8.2 | 8.2 | 234.65 | 83.221 | 177390000 | 19 | Myoferlin | MYOF |
| 18.10285 | 22.25026 | 22.96712 | 20.59104 | 4 | 4 | 4 | 17.9 | 17.9 | 17.9 | 34.812 | 27.768 | 91129000 | 7 | RNA-binding protein Musashi homolog 2 | IGSF9B;MSI2 |
| 23.54038 | 22.13962 | 22.9612 | 22.74866 | 4 | 4 | 4 | 14.3 | 14.3 | 14.3 | 32.426 | 30.25 | 94764000 | 18 | Dolichol-phosphate mannosyltransferase subunit 1 | DPM1 |
| 23.38126 | 22.07143 | 22.95975 | 21.72096 | 14 | 14 | 14 | 13 | 13 | 13 | 143.19 | 179.7 | 241520000 | 19 | Cyclin-G-associated kinase | GAK |
| 23.71984 | 21.82194 | 22.95141 | 23.35864 | 6 | 6 | 6 | 21.4 | 21.4 | 21.4 | 37.32 | 47.639 | 337570000 | 12 | Hydroxysteroid dehydrogenase-like protein 2 | HSDL2 |
| 18.12147 | 22.58566 | 22.94254 | 23.08057 | 5 | 5 | 5 | 13.8 | 13.8 | 13.8 | 48.375 | 33.28 | 119330000 | 9 | Prenylcysteine oxidase 1 | PCYOX1 |
| 21.79229 | 23.60953 | 22.94041 | 23.05411 | 7 | 7 | 7 | 17.6 | 17.6 | 17.6 | 63.523 | 61.662 | 179970000 | 11 | Sphingosine-1-phosphate lyase 1 | SGPL1 |
| 24.99589 | 23.07537 | 22.93916 | 22.62692 | 14 | 14 | 14 | 20.7 | 20.7 | 20.7 | 109.13 | 216.57 | 1108900000 | 22 | Kinesin-like protein KIF23 | KIF23 |
| 22.49277 | 21.64055 | 22.93787 | 22.35162 | 5 | 5 | 5 | 17.9 | 17.9 | 17.9 | 36.351 | 30.607 | 178920000 | 10 | cAMP-dependent protein kinase catalytic subunit beta | PRKACB |
| 24.30918 | 22.94086 | 22.93682 | 23.62879 | 8 | 8 | 8 | 27.3 | 27.3 | 27.3 | 36.091 | 58.083 | 569030000 | 17 | ELAV-like protein 1 | ELAVL1 |
| 17.93911 | 22.35262 | 22.92452 | 22.48328 | 5 | 5 | 5 | 14.4 | 14.4 | 14.4 | 46.48 | 35.181 | 105340000 | 7 | Adipocyte plasma membrane-associated protein | APMAP |
| 22.25661 | 22.61495 | 22.9208 | 23.24125 | 8 | 8 | 8 | 11.3 | 11.3 | 11.3 | 109.96 | 98.303 | 321330000 | 14 | Exportin-T | XPOT |
| 23.28193 | 22.999 | 22.91916 | 24.15777 | 9 | 9 | 6 | 31.3 | 31.3 | 15.7 | 40.313 | 161.16 | 359400000 | 16 | RNA-binding protein 4;RNA-binding protein 4B | RBM4;RBM4B |
| 25.16592 | 23.5285 | 22.9111 | 22.30611 | 17 | 17 | 17 | 25.4 | 25.4 | 25.4 | 87.282 | 191 | 1173500000 | 26 | Acyl-CoA dehydrogenase family member 11 | ACAD11 |
| 22.72993 | 23.71837 | 22.91092 | 24.40613 | 8 | 8 | 8 | 29.2 | 29.2 | 29.2 | 40.307 | 76.663 | 465810000 | 14 | Sialic acid synthase | NANS |
| 22.32317 | 22.36733 | 22.90218 | 20.01116 | 4 | 4 | 4 | 11.3 | 11.3 | 11.3 | 48.117 | 28.226 | 124300000 | 7 | 39S ribosomal protein L37, mitochondrial | MRPL37 |
| 24.07096 | 24.43663 | 22.90209 | 23.97421 | 18 | 18 | 18 | 18.7 | 18.7 | 18.7 | 138.83 | 151.56 | 719880000 | 33 | Eukaryotic translation initiation factor 5B | EIF5B |
| 24.78985 | 23.06651 | 22.90095 | 23.05712 | 5 | 5 | 5 | 34.5 | 34.5 | 34.5 | 25.984 | 48.914 | 95948000 | 11 | 3-hydroxyacyl-CoA dehydrogenase type-2 | HSD17B10 |
| 22.17992 | 22.57886 | 22.8973 | 23.56651 | 7 | 7 | 7 | 22.3 | 22.3 | 22.3 | 33.969 | 49.646 | 122840000 | 12 | Transcription elongation factor A protein 1 | TCEA1 |
| 22.15292 | 23.8468 | 22.89062 | 21.88792 | 5 | 5 | 5 | 11.2 | 11.2 | 11.2 | 49.038 | 30.773 | 209450000 | 10 | Periodic tryptophan protein 1 homolog | PWP1 |
| 22.54541 | 22.82637 | 22.87854 | 18.87308 | 9 | 9 | 9 | 8.9 | 8.9 | 8.9 | 138.62 | 61.941 | 122540000 | 14 | DNA-directed RNA polymerase, mitochondrial | POLRMT |
| 24.05871 | 24.16139 | 22.87821 | 19.14274 | 8 | 8 | 8 | 10.2 | 10.2 | 10.2 | 105.32 | 59.97 | 550310000 | 14 | WD repeat-containing protein 36 | WDR36 |
| 22.66023 | 22.71547 | 22.87031 | 19.62728 | 4 | 4 | 4 | 7.3 | 7.3 | 7.3 | 84.685 | 47.31 | 295670000 | 7 | Procollagen-lysine,2-oxoglutarate 5-dioxygenase 2 | PLOD2 |
| 22.3909 | 22.74043 | 22.86987 | 23.55497 | 10 | 10 | 10 | 27.3 | 27.3 | 27.3 | 40.763 | 59.83 | 230560000 | 17 | Peptidyl-prolyl cis-trans isomerase D | PPID |
| 23.91333 | 21.37204 | 22.86961 | 22.01859 | 3 | 3 | 3 | 28.3 | 28.3 | 28.3 | 10.666 | 19.603 | 61623000 | 11 | High mobility group nucleosome-binding domain-containing protein 3;Non-histone chromosomal protein HMG-17 | HMGN3;HMGN2 |
| 23.62634 | 24.27313 | 22.86546 | 24.74443 | 21 | 21 | 20 | 18.4 | 18.4 | 17.8 | 174.97 | 151.17 | 396220000 | 32 | UDP-glucose:glycoprotein glucosyltransferase 1 | UGGT1 |
| 22.13781 | 22.44569 | 22.8644 | 23.05887 | 8 | 8 | 8 | 15 | 15 | 15 | 71.607 | 91.833 | 291050000 | 8 | Cell surface glycoprotein MUC18 | MCAM |
| 24.387 | 24.2103 | 22.86024 | 23.44493 | 11 | 11 | 11 | 21.6 | 21.6 | 21.6 | 76.758 | 156.9 | 759300000 | 26 | Glutamine--fructose-6-phosphate aminotransferase [isomerizing] 1 | GFPT1 |
| 22.32973 | 22.72505 | 22.8596 | 23.70398 | 6 | 6 | 6 | 7.9 | 7.9 | 7.9 | 106.04 | 54.803 | 303120000 | 10 | Isoleucine--tRNA ligase, mitochondrial | IARS2 |
| 23.75624 | 21.82069 | 22.85673 | 22.97351 | 5 | 5 | 3 | 26.8 | 26.8 | 17.2 | 22.367 | 31.525 | 52294000 | 12 | GTP-binding protein SAR1a | SAR1A |
| 24.64218 | 24.27874 | 22.84896 | 21.05119 | 10 | 10 | 10 | 43.1 | 43.1 | 43.1 | 28.415 | 137.45 | 517980000 | 19 | U2 small nuclear ribonucleoprotein A | SNRPA1 |
| 20.74197 | 21.8628 | 22.84623 | 19.15689 | 4 | 4 | 4 | 12.6 | 12.6 | 12.6 | 44.596 | 28.448 | 71746000 | 9 | 39S ribosomal protein L38, mitochondrial | MRPL38 |
| 21.53445 | 22.20272 | 22.84236 | 22.52416 | 3 | 3 | 3 | 12.1 | 12.1 | 12.1 | 32.113 | 22.217 | 128870000 | 8 | Pirin | PIR |
| 23.00136 | 23.21784 | 22.84019 | 23.50252 | 9 | 9 | 9 | 25.9 | 25.9 | 25.9 | 50.608 | 64.544 | 300700000 | 18 | Septin-7 | 07. Sep |
| 22.32699 | 22.16705 | 22.83669 | 22.74651 | 7 | 7 | 7 | 20.8 | 20.8 | 20.8 | 48.349 | 85.459 | 184670000 | 15 | GTPase Era, mitochondrial | ERAL1 |
| 21.47694 | 22.65672 | 22.82844 | 22.16791 | 7 | 7 | 7 | 9.7 | 9.7 | 9.7 | 98.008 | 47.028 | 204320000 | 12 | Extended synaptotagmin-2 | ESYT2 |
| 24.20754 | 23.42969 | 22.81991 | 23.39846 | 11 | 11 | 11 | 14.8 | 14.8 | 14.8 | 95.581 | 88.784 | 656700000 | 18 | Probable ATP-dependent RNA helicase DDX23 | DDX23 |
| 22.22338 | 24.80045 | 22.81623 | 19.44401 | 15 | 15 | 15 | 15.7 | 15.7 | 15.7 | 134.46 | 102.14 | 195670000 | 19 | 1-phosphatidylinositol 4,5-bisphosphate phosphodiesterase beta-4 | PLCB4 |
| 21.73901 | 22.40143 | 22.80189 | 22.72165 | 9 | 9 | 9 | 23.5 | 23.5 | 23.5 | 51.355 | 67.974 | 144160000 | 15 | Mitochondrial import inner membrane translocase subunit TIM44 | TIMM44 |
| 21.61648 | 22.32435 | 22.80182 | 22.93105 | 7 | 7 | 7 | 27.2 | 27.2 | 27.2 | 37.432 | 58.507 | 139460000 | 16 | Glutaredoxin-3 | GLRX3 |
| 21.86106 | 21.83078 | 22.79487 | 19.46945 | 4 | 4 | 4 | 12.4 | 12.4 | 12.4 | 39.474 | 27.37 | 103130000 | 9 | Hsp70-binding protein 1 | HSPBP1 |
| 22.76715 | 22.40112 | 22.79176 | 19.29236 | 6 | 6 | 6 | 10.4 | 10.4 | 10.4 | 94.139 | 49.991 | 231620000 | 6 | AP-1 complex subunit gamma-1 | AP1G1 |
| 20.84927 | 22.41939 | 22.78967 | 21.01038 | 7 | 7 | 7 | 5.4 | 5.4 | 5.4 | 228.18 | 40.838 | 126190000 | 13 | Protein SON | SON |
| 24.38962 | 22.44149 | 22.78766 | 22.22855 | 10 | 10 | 9 | 20.9 | 20.9 | 19.2 | 60.13 | 75.824 | 520140000 | 13 | Copine-3 | CPNE3 |
| 20.43237 | 21.43461 | 22.78724 | 21.35612 | 5 | 5 | 5 | 10.6 | 10.6 | 10.6 | 49.647 | 28.761 | 47382000 | 6 | Pre-mRNA-splicing factor CWC25 homolog | CWC25 |
| 17.87028 | 21.23327 | 22.76707 | 19.73672 | 3 | 3 | 3 | 10.2 | 10.2 | 10.2 | 34.604 | 18.739 | 33627000 | 4 | Acid ceramidase;Acid ceramidase subunit alpha;Acid ceramidase subunit beta | ASAH1 |
| 22.40133 | 22.6971 | 22.7663 | 23.64229 | 5 | 5 | 5 | 13.4 | 13.4 | 13.4 | 42.006 | 33.894 | 189510000 | 12 | Polyadenylate-binding protein-interacting protein 1 | PAIP1 |
| 23.21941 | 22.48099 | 22.75977 | 23.67131 | 5 | 4 | 3 | 32 | 26 | 20.4 | 20.697 | 27.049 | 75858000 | 17 | ADP-ribosylation factor 1;ADP-ribosylation factor 3 | ARF1;ARF3 |
| 21.68769 | 22.80193 | 22.75874 | 23.83084 | 10 | 10 | 10 | 12.1 | 12.1 | 12.1 | 113.89 | 74.958 | 313910000 | 12 | NAD(P) transhydrogenase, mitochondrial | NNT |
| 22.81921 | 24.43517 | 22.75087 | 19.86758 | 6 | 6 | 6 | 20.9 | 20.9 | 20.9 | 35.582 | 49.867 | 172820000 | 13 | Ribosome production factor 2 homolog | RPF2 |
| 18.66682 | 22.1764 | 22.74897 | 23.35528 | 5 | 5 | 5 | 15.3 | 15.3 | 15.3 | 41.389 | 28.842 | 53444000 | 9 | Mitogen-activated protein kinase 1;Mitogen-activated protein kinase | MAPK1 |
| 22.56934 | 22.27025 | 22.74649 | 23.4325 | 22 | 4 | 1 | 50.8 | 10.8 | 2.7 | 49.906 | 29.894 | 214890000 | 13 | Tubulin beta-2A chain | TUBB2A |
| 22.6646 | 25.56924 | 22.74567 | 22.76059 | 19 | 19 | 19 | 26.1 | 26.1 | 26.1 | 91.48 | 225.6 | 948300000 | 24 | ATP-dependent RNA helicase DDX24 | DDX24 |
| 22.35523 | 23.2449 | 22.74242 | 22.93774 | 9 | 9 | 8 | 11 | 11 | 10.1 | 113.09 | 108.24 | 353240000 | 11 | ATP-dependent RNA helicase DHX36 | DHX36 |
| 22.94038 | 22.63786 | 22.72132 | 24.65867 | 8 | 8 | 8 | 12.9 | 12.9 | 12.9 | 93.307 | 108.58 | 504290000 | 18 | Ubiquitin carboxyl-terminal hydrolase 5 | USP5 |
| 24.39578 | 24.01631 | 22.71768 | 23.35703 | 16 | 16 | 16 | 18.4 | 18.4 | 18.4 | 134.62 | 250.98 | 551710000 | 30 | Inverted formin-2 | INF2 |
| 22.25957 | 24.35911 | 22.70331 | 22.24461 | 5 | 5 | 5 | 35.4 | 35.4 | 35.4 | 21.883 | 33.137 | 53141000 | 9 | Uncharacterized protein C7orf50 | C7orf50 |
| 22.60116 | 22.90261 | 22.70107 | 19.56986 | 3 | 3 | 3 | 14.9 | 14.9 | 14.9 | 36.724 | 30.431 | 152300000 | 6 | Methylosome protein 50 | WDR77 |
| 22.71961 | 22.69566 | 22.69897 | 23.39807 | 9 | 9 | 9 | 12.2 | 12.2 | 12.2 | 117.39 | 85.12 | 342450000 | 14 | Extended synaptotagmin-1 | ESYT1 |
| 24.26585 | 25.08982 | 22.69572 | 24.69742 | 8 | 8 | 8 | 28.7 | 28.7 | 28.7 | 33.24 | 102.17 | 715820000 | 28 | Eukaryotic translation initiation factor 3 subunit F | EIF3F |
| 22.11753 | 22.39642 | 22.69551 | 22.98777 | 6 | 6 | 6 | 8 | 8 | 8 | 104.85 | 43.541 | 261950000 | 9 | Catenin delta-1 | CTNND1 |
| 22.15866 | 22.18797 | 22.69439 | 21.89852 | 7 | 7 | 7 | 14.3 | 14.3 | 14.3 | 57.56 | 45.742 | 131710000 | 13 | Apoptosis inhibitor 5 | API5 |
| 23.04014 | 22.82902 | 22.69211 | 23.76213 | 14 | 14 | 14 | 23.6 | 23.6 | 23.6 | 85.95 | 122.83 | 440070000 | 19 | Polyribonucleotide nucleotidyltransferase 1, mitochondrial | PNPT1 |
| 21.98426 | 22.61798 | 22.69151 | 22.2201 | 6 | 6 | 6 | 26.5 | 26.5 | 26.5 | 31.512 | 39.445 | 131700000 | 12 | Sodium/potassium-transporting ATPase subunit beta-3 | ATP1B3 |
| 24.22361 | 23.40158 | 22.68874 | 23.83152 | 9 | 9 | 9 | 28.1 | 28.1 | 28.1 | 52.623 | 106.47 | 492470000 | 20 | FAS-associated factor 2 | FAF2 |
| 22.95395 | 22.08297 | 22.68643 | 22.51761 | 8 | 8 | 8 | 8.5 | 8.5 | 8.5 | 118.82 | 53.144 | 304700000 | 10 | Gem-associated protein 4 | GEMIN4 |
| 21.32855 | 21.54788 | 22.6843 | 22.21931 | 5 | 5 | 5 | 8.2 | 8.2 | 8.2 | 68.76 | 30.6 | 109050000 | 7 | Acyl-CoA dehydrogenase family member 9, mitochondrial | ACAD9 |
| 23.23872 | 22.66466 | 22.67992 | 23.85766 | 16 | 11 | 11 | 26.7 | 21.7 | 21.7 | 67.688 | 85.049 | 342510000 | 18 | Lamin-B2 | LMNB2 |
| 17.22423 | 22.14071 | 22.67988 | 20.14789 | 4 | 4 | 4 | 11.2 | 11.2 | 11.2 | 48.534 | 27.365 | 60995000 | 8 | Ornithine aminotransferase, mitochondrial;Ornithine aminotransferase, hepatic form;Ornithine aminotransferase, renal form | OAT |
| 23.85205 | 22.99341 | 22.67663 | 22.3756 | 12 | 12 | 12 | 58.3 | 58.3 | 58.3 | 26.21 | 114.49 | 322810000 | 20 | Calcyclin-binding protein | CACYBP |
| 22.10197 | 22.47756 | 22.67334 | 20.49275 | 4 | 4 | 4 | 9.9 | 9.9 | 9.9 | 36.788 | 26.072 | 151560000 | 8 | Aldo-keto reductase family 1 member C1;Aldo-keto reductase family 1 member C3;Aldo-keto reductase family 1 member C2;Aldo-keto reductase family 1 member C4 | AKR1C1;AKR1C2;AKR1C3;AKR1C4 |
| 22.77266 | 22.90832 | 22.66843 | 20.07064 | 14 | 14 | 14 | 14.2 | 14.2 | 14.2 | 151.66 | 138.3 | 219990000 | 17 | Enhancer of mRNA-decapping protein 4 | EDC4 |
| 21.4979 | 22.12038 | 22.6664 | 18.77462 | 4 | 4 | 4 | 6.8 | 6.8 | 6.8 | 89.09 | 25.446 | 77495000 | 7 | RelA-associated inhibitor | PPP1R13L |
| 22.51465 | 25.30414 | 22.66125 | 21.82796 | 20 | 20 | 20 | 27.6 | 27.6 | 27.6 | 100.83 | 156.48 | 668300000 | 22 | Exosome component 10 | EXOSC10 |
| 23.05354 | 21.8808 | 22.65556 | 19.67706 | 3 | 3 | 3 | 10.2 | 10.2 | 10.2 | 48.442 | 45.466 | 181990000 | 6 | Cytochrome b-c1 complex subunit 2, mitochondrial | UQCRC2 |
| 16.97727 | 16.92776 | 22.65476 | 22.73616 | 3 | 3 | 2 | 34.9 | 34.9 | 24.4 | 9.8642 | 18.174 | 23251000 | 4 | Stathmin | STMN1 |
| 25.09285 | 23.26413 | 22.64632 | 23.39898 | 3 | 3 | 3 | 25.6 | 25.6 | 25.6 | 14.131 | 23.107 | 116350000 | 7 | Single-stranded DNA-binding protein, mitochondrial | SSBP1 |
| 22.96238 | 24.58669 | 22.63759 | 22.35598 | 7 | 4 | 4 | 32.3 | 23.7 | 23.7 | 40.089 | 105.88 | 317950000 | 11 | Y-box-binding protein 3 | YBX3 |
| 22.42386 | 22.19832 | 22.63436 | 22.2723 | 5 | 5 | 5 | 31.9 | 31.9 | 31.9 | 28.882 | 102.09 | 176070000 | 8 | Twinfilin-2 | TWF2 |
| 22.3103 | 22.40198 | 22.63181 | 22.5742 | 6 | 6 | 6 | 10.2 | 10.2 | 10.2 | 71.223 | 38.486 | 219810000 | 12 | SUMO-activating enzyme subunit 2 | UBA2 |
| 18.2835 | 21.71473 | 22.62999 | 22.53521 | 3 | 3 | 3 | 36.2 | 36.2 | 36.2 | 13.331 | 19.885 | 20789000 | 6 | Gamma-synuclein | SNCG |
| 22.53848 | 21.82648 | 22.60574 | 22.77676 | 6 | 6 | 6 | 18.4 | 18.4 | 18.4 | 43.359 | 43.702 | 153950000 | 11 | Protein pelota homolog | PELO |
| 23.71513 | 22.60585 | 22.59944 | 18.99391 | 14 | 14 | 14 | 51.7 | 51.7 | 51.7 | 34.333 | 166.2 | 386350000 | 25 | Actin-related protein 2/3 complex subunit 2 | ARPC2 |
| 24.24489 | 22.9514 | 22.59833 | 21.96155 | 8 | 8 | 8 | 20.5 | 20.5 | 20.5 | 52.062 | 113.9 | 535810000 | 14 | Platelet glycoprotein 4 | CD36 |
| 21.649 | 21.43705 | 22.59746 | 22.08855 | 4 | 4 | 4 | 8.2 | 8.2 | 8.2 | 55.092 | 25.745 | 96027000 | 8 | COP9 signalosome complex subunit 1 | GPS1 |
| 22.06625 | 22.16883 | 22.59245 | 22.89058 | 5 | 5 | 5 | 10.4 | 10.4 | 10.4 | 57.342 | 32.659 | 164900000 | 9 | Protein phosphatase 1G | PPM1G |
| 22.19827 | 22.29558 | 22.59069 | 22.74024 | 3 | 3 | 3 | 15.9 | 15.9 | 15.9 | 27.041 | 19.947 | 126310000 | 7 | Cytosolic acyl coenzyme A thioester hydrolase | ACOT7 |
| 24.80858 | 23.6767 | 22.58538 | 23.3648 | 20 | 20 | 20 | 21.6 | 21.6 | 21.6 | 109.54 | 154.81 | 569060000 | 26 | Aminopeptidase N | ANPEP |
| 23.09589 | 22.99599 | 22.58396 | 22.76314 | 4 | 4 | 4 | 20.5 | 20.5 | 20.5 | 29.204 | 26.106 | 66207000 | 6 | Proteasome subunit beta type-4 | PSMB4 |
| 22.88616 | 23.82357 | 22.57971 | 18.78321 | 8 | 8 | 7 | 15.7 | 15.7 | 14.4 | 74.324 | 70.308 | 424800000 | 9 | SRSF protein kinase 1 | SRPK1 |
| 23.43097 | 22.44927 | 22.57861 | 21.59377 | 7 | 7 | 7 | 13.2 | 13.2 | 13.2 | 65.653 | 49.68 | 392000000 | 6 | DBIRD complex subunit ZNF326 | ZNF326 |
| 23.2266 | 22.57094 | 22.57649 | 22.98735 | 5 | 5 | 4 | 33.3 | 33.3 | 28.7 | 21.671 | 31.593 | 42600000 | 8 | Membrane-associated progesterone receptor component 1 | PGRMC1 |
| 23.9534 | 22.80487 | 22.57489 | 23.04624 | 3 | 3 | 3 | 25.7 | 25.7 | 25.7 | 23.845 | 27.309 | 68082000 | 8 | Ubiquitin-conjugating enzyme E2 S | UBE2S |
| 23.04763 | 22.48038 | 22.56246 | 22.75216 | 4 | 4 | 4 | 12.1 | 12.1 | 12.1 | 44.414 | 38.721 | 222110000 | 7 | UDP-glucose 6-dehydrogenase | UGDH |
| 23.52612 | 22.67495 | 22.55565 | 23.86467 | 8 | 8 | 8 | 14.1 | 14.1 | 14.1 | 82.936 | 81.037 | 589060000 | 17 | ATP-binding cassette sub-family D member 1 | ABCD1 |
| 23.69996 | 22.66891 | 22.55513 | 23.64702 | 4 | 3 | 3 | 22.4 | 16.7 | 16.7 | 21.45 | 19.074 | 78003000 | 10 | Ras-related C3 botulinum toxin substrate 1;Ras-related C3 botulinum toxin substrate 3;Ras-related C3 botulinum toxin substrate 2 | RAC1;RAC3;RAC2 |
| 24.66568 | 23.33456 | 22.55302 | 19.92994 | 13 | 13 | 13 | 56.8 | 56.8 | 56.8 | 31.566 | 205.42 | 660660000 | 27 | Voltage-dependent anion-selective channel protein 2 | VDAC2 |
| 24.93481 | 22.99298 | 22.55105 | 23.02596 | 8 | 8 | 8 | 37.3 | 37.3 | 37.3 | 15.945 | 48.949 | 130020000 | 12 | Coactosin-like protein | COTL1 |
| 24.15051 | 18.80367 | 22.54494 | 18.99169 | 3 | 3 | 3 | 27.8 | 27.8 | 27.8 | 11.985 | 21.05 | 66640000 | 4 | CD59 glycoprotein | CD59 |
| 17.63714 | 22.21036 | 22.5417 | 22.71812 | 6 | 6 | 6 | 17.7 | 17.7 | 17.7 | 41.071 | 38.737 | 57784000 | 12 | Protein-L-isoaspartate O-methyltransferase domain-containing protein 2 | PCMTD2 |
| 24.58377 | 22.78883 | 22.53457 | 22.66899 | 10 | 7 | 7 | 21.1 | 17.7 | 17.7 | 55.277 | 69.645 | 542360000 | 12 | Inosine-5-monophosphate dehydrogenase;Inosine-5-monophosphate dehydrogenase 1 | IMPDH1 |
| 20.99921 | 22.30458 | 22.51879 | 23.2166 | 3 | 3 | 3 | 20.5 | 20.5 | 20.5 | 18.488 | 18.234 | 40873000 | 7 | Transmembrane emp24 domain-containing protein 2 | TMED2 |
| 25.59281 | 24.36005 | 22.50373 | 23.03961 | 33 | 33 | 33 | 29.3 | 29.3 | 29.3 | 143.23 | 317.83 | 936780000 | 53 | Structural maintenance of chromosomes protein 1A | SMC1A |
| 25.46214 | 27.7433 | 22.50045 | 22.68919 | 27 | 27 | 27 | 42.7 | 42.7 | 42.7 | 54.972 | 323.31 | 2278600000 | 65 | Ribosomal L1 domain-containing protein 1 | RSL1D1 |
| 23.48493 | 24.03959 | 22.49285 | 23.67885 | 6 | 6 | 6 | 11.6 | 11.6 | 11.6 | 54.75 | 45.36 | 335400000 | 10 | Delta(24)-sterol reductase | DHCR24 |
| 23.75837 | 21.49033 | 22.49145 | 19.19007 | 9 | 4 | 4 | 28 | 13 | 13 | 40.361 | 32.755 | 297020000 | 5 | Guanine nucleotide-binding protein G(i) subunit alpha-1 | GNAI1 |
| 19.48101 | 21.57413 | 22.48986 | 18.41251 | 3 | 3 | 3 | 12.9 | 12.9 | 12.9 | 37.578 | 25.514 | 66055000 | 8 | COP9 signalosome complex subunit 5 | COPS5 |
| 21.96504 | 22.12019 | 22.47806 | 22.94097 | 4 | 4 | 4 | 14.8 | 14.8 | 14.8 | 37.636 | 24.197 | 83071000 | 7 | AH receptor-interacting protein | AIP |
| 20.55244 | 23.15443 | 22.47721 | 19.95812 | 18 | 5 | 4 | 29 | 11.1 | 8.4 | 68.563 | 37.534 | 203220000 | 7 | Radixin | RDX |
| 23.11304 | 22.94011 | 22.47472 | 22.91929 | 8 | 8 | 5 | 18.7 | 18.7 | 11.4 | 60.029 | 59.005 | 284280000 | 12 | Importin subunit alpha-7;Importin subunit alpha-6 | KPNA6;KPNA5 |
| 22.85386 | 18.77727 | 22.47293 | 21.9673 | 4 | 4 | 4 | 16.1 | 16.1 | 16.1 | 27.085 | 23.382 | 24880000 | 8 | 39S ribosomal protein L47, mitochondrial | MRPL47 |
| 23.31672 | 18.17373 | 22.47217 | 23.308 | 3 | 3 | 3 | 15.4 | 15.4 | 15.4 | 23.31 | 20.715 | 95466000 | 11 | Ran-specific GTPase-activating protein | RANBP1 |
| 17.23055 | 22.42806 | 22.47214 | 22.5799 | 6 | 6 | 6 | 23.5 | 23.5 | 23.5 | 38.324 | 43.086 | 75460000 | 7 | Sorbitol dehydrogenase | SORD |
| 22.57699 | 23.49338 | 22.46431 | 24.40743 | 5 | 5 | 5 | 34.6 | 34.6 | 34.6 | 17.499 | 32.139 | 72771000 | 16 | Adenylate kinase 2, mitochondrial;Adenylate kinase 2, mitochondrial;Adenylate kinase 2, mitochondrial, N-terminally processed | AK2 |
| 21.39634 | 22.08521 | 22.46311 | 22.65042 | 5 | 5 | 5 | 10.3 | 10.3 | 10.3 | 58.252 | 30.909 | 110640000 | 8 | Mitochondrial-processing peptidase subunit alpha | PMPCA |
| 24.98407 | 22.00724 | 22.45876 | 21.27088 | 10 | 10 | 6 | 40.3 | 40.3 | 24.4 | 37.377 | 167.08 | 656270000 | 23 | Guanine nucleotide-binding protein G(I)/G(S)/G(T) subunit beta-1 | GNB1 |
| 21.17773 | 22.07019 | 22.45551 | 22.61043 | 8 | 8 | 8 | 3.8 | 3.8 | 3.8 | 227.92 | 50.568 | 182820000 | 10 | Nuclear pore complex protein Nup205 | NUP205 |
| 23.43746 | 22.14576 | 22.45551 | 22.87596 | 7 | 7 | 7 | 16.1 | 16.1 | 16.1 | 53.753 | 51.362 | 289890000 | 11 | Putative adenosylhomocysteinase 2;Adenosylhomocysteinase;Putative adenosylhomocysteinase 3 | AHCYL1;AHCYL2 |
| 23.28546 | 23.21549 | 22.45071 | 22.97654 | 6 | 6 | 6 | 17.6 | 17.6 | 17.6 | 45.004 | 42.778 | 280800000 | 10 | Cytoplasmic dynein 1 light intermediate chain 2 | DYNC1LI2 |
| 21.89493 | 23.40393 | 22.45003 | 23.90931 | 9 | 9 | 9 | 18.5 | 18.5 | 18.5 | 63.502 | 59.267 | 244410000 | 14 | Septin-9 | 09. Sep |
| 22.2567 | 21.40328 | 22.44968 | 21.85209 | 5 | 5 | 5 | 11.6 | 11.6 | 11.6 | 62.288 | 33.717 | 110460000 | 11 | Eukaryotic translation initiation factor 2A;Eukaryotic translation initiation factor 2A, N-terminally processed | EIF2A |
| 16.02375 | 22.56957 | 22.44524 | 22.89999 | 7 | 7 | 7 | 9 | 9 | 9 | 86.982 | 45.376 | 152900000 | 8 | Cullin-2 | CUL2 |
| 20.24679 | 23.3644 | 22.44471 | 22.23497 | 12 | 12 | 7 | 7 | 7 | 4.2 | 215.31 | 79.683 | 270500000 | 19 | Chromodomain-helicase-DNA-binding protein 4 | CHD4 |
| 22.17011 | 24.38929 | 22.43865 | 22.12161 | 4 | 4 | 4 | 20.8 | 20.8 | 20.8 | 34.91 | 102.69 | 181620000 | 8 | Transcriptional activator protein Pur-alpha | PURA |
| 21.77941 | 22.30755 | 22.43743 | 23.45501 | 5 | 5 | 5 | 5.9 | 5.9 | 5.9 | 116.92 | 38.298 | 223520000 | 6 | UDP-N-acetylglucosamine--peptide N-acetylglucosaminyltransferase 110 kDa subunit | OGT |
| 21.06243 | 22.70415 | 22.43428 | 22.58786 | 12 | 12 | 10 | 5.5 | 5.5 | 4.7 | 308.6 | 89.572 | 381280000 | 18 | Inositol 1,4,5-trisphosphate receptor type 1 | ITPR1 |
| 21.58348 | 21.82022 | 22.43199 | 22.37398 | 8 | 8 | 8 | 12.7 | 12.7 | 12.7 | 85.676 | 56.287 | 276880000 | 10 | Nuclear pore complex protein Nup98-Nup96;Nuclear pore complex protein Nup98;Nuclear pore complex protein Nup96 | NUP98 |
| 24.08797 | 23.74232 | 22.43079 | 18.67173 | 11 | 11 | 11 | 42.6 | 42.6 | 42.6 | 35.079 | 91.218 | 504450000 | 18 | Electron transfer flavoprotein subunit alpha, mitochondrial | ETFA |
| 23.41209 | 22.22072 | 22.43041 | 19.44385 | 7 | 7 | 7 | 33.8 | 33.8 | 33.8 | 27.872 | 74.629 | 299290000 | 11 | Splicing factor U2AF 35 kDa subunit | U2AF1 |
| 22.19349 | 23.13381 | 22.43038 | 22.36239 | 7 | 7 | 7 | 24.4 | 24.4 | 24.4 | 41.044 | 61.097 | 122660000 | 12 | 28S ribosomal protein S29, mitochondrial | DAP3 |
| 21.9281 | 24.42085 | 22.42995 | 24.55918 | 6 | 6 | 6 | 12.6 | 12.6 | 12.6 | 54.412 | 60.528 | 213190000 | 14 | Podocalyxin | PODXL |
| 19.08838 | 18.04951 | 22.42524 | 19.42659 | 3 | 3 | 3 | 22.2 | 22.2 | 22.2 | 9.898 | 18.588 | 46018000 | 6 | Secretoglobin family 1D member 1 | SCGB1D1 |
| 26.89331 | 22.02059 | 22.41803 | 22.83665 | 6 | 6 | 2 | 36.6 | 36.6 | 14.1 | 21.258 | 49.777 | 357380000 | 12 | Cell division control protein 42 homolog | CDC42 |
| 22.21092 | 18.08223 | 22.41129 | 19.44316 | 8 | 8 | 7 | 10.1 | 10.1 | 8.6 | 106.84 | 66.531 | 300260000 | 9 | Protein-methionine sulfoxide oxidase MICAL3 | MICAL3 |
| 22.90524 | 22.24766 | 22.4109 | 23.26513 | 11 | 11 | 11 | 13.8 | 13.8 | 13.8 | 101.67 | 82.071 | 360320000 | 13 | Protein unc-45 homolog A | UNC45A |
| 23.94798 | 24.0648 | 22.40624 | 23.3644 | 17 | 17 | 17 | 17.8 | 17.8 | 17.8 | 136.22 | 132.74 | 525670000 | 24 | Protein transport protein Sec31A | SEC31A |
| 22.26588 | 24.36172 | 22.39613 | 22.30758 | 8 | 8 | 8 | 26.6 | 26.6 | 26.6 | 33.24 | 55.245 | 170360000 | 11 | Transcriptional activator protein Pur-beta | PURB |
| 24.47128 | 22.49612 | 22.39574 | 22.38145 | 5 | 5 | 5 | 19.5 | 19.5 | 19.5 | 23.51 | 31.471 | 67546000 | 7 | Mitotic spindle assembly checkpoint protein MAD2A | MAD2L1 |
| 23.98076 | 22.7575 | 22.39268 | 22.9173 | 7 | 7 | 7 | 16.8 | 16.8 | 16.8 | 65.391 | 72.765 | 436350000 | 13 | SNW domain-containing protein 1 | SNW1 |
| 22.19964 | 22.17387 | 22.3894 | 22.89791 | 3 | 3 | 3 | 16 | 16 | 16 | 24.347 | 18.887 | 29656000 | 8 | Ras-related protein Rab-21 | RAB21 |
| 24.8723 | 25.82675 | 22.38472 | 24.59565 | 27 | 27 | 27 | 35.6 | 35.6 | 35.6 | 104.78 | 245.65 | 781320000 | 44 | Hypoxia up-regulated protein 1 | HYOU1 |
| 23.18224 | 24.53011 | 22.37716 | 22.20866 | 14 | 14 | 14 | 13 | 13 | 13 | 139.41 | 116.06 | 303570000 | 21 | Nardilysin | NRD1 |
| 22.51523 | 22.71009 | 22.37528 | 22.97647 | 3 | 3 | 3 | 5 | 5 | 5 | 86.537 | 28.445 | 94638000 | 9 | CD97 antigen;CD97 antigen subunit alpha;CD97 antigen subunit beta | CD97 |
| 18.88395 | 22.32573 | 22.37488 | 22.76316 | 3 | 3 | 3 | 9.1 | 9.1 | 9.1 | 42.064 | 23.367 | 46919000 | 8 | Glutamine synthetase | GLUL |
| 22.5121 | 22.21463 | 22.37433 | 19.97499 | 5 | 4 | 4 | 14 | 11.8 | 11.8 | 42.777 | 60.553 | 177760000 | 9 | Cyclin-dependent kinase 9 | CDK9 |
| 21.00203 | 21.37973 | 22.3722 | 22.02394 | 7 | 3 | 3 | 20.3 | 9.1 | 9.1 | 39.309 | 17.946 | 65685000 | 6 | Inhibitor of nuclear factor kappa-B kinase-interacting protein | IKBIP |
| 26.43552 | 22.41302 | 22.36762 | 22.65007 | 71 | 58 | 58 | 35.4 | 31 | 31 | 271.32 | 323.31 | 4978600000 | 69 | Spectrin beta chain, non-erythrocytic 2 | SPTBN2 |
| 26.99367 | 23.39598 | 22.36746 | 21.49644 | 37 | 37 | 5 | 34.6 | 34.6 | 4.3 | 144.75 | 323.31 | 2270600000 | 53 | Protein flightless-1 homolog | FLII |
| 25.4928 | 22.32655 | 22.36744 | 22.31465 | 13 | 13 | 11 | 18.5 | 18.5 | 15.9 | 69.72 | 107.7 | 1025600000 | 20 | Fragile X mental retardation syndrome-related protein 1 | FXR1 |
| 23.39637 | 22.57964 | 22.36453 | 23.09055 | 7 | 7 | 7 | 14.6 | 14.6 | 14.6 | 87.98 | 166.84 | 479300000 | 12 | Signal transducer and activator of transcription 3;Signal transducer and activator of transcription | STAT3 |
| 17.41279 | 22.52097 | 22.35947 | 22.68629 | 5 | 5 | 5 | 15.4 | 15.4 | 15.4 | 41.35 | 30.116 | 57265000 | 8 | Acetyl-CoA acetyltransferase, cytosolic | ACAT2 |
| 21.7503 | 21.32312 | 22.35512 | 18.55272 | 3 | 3 | 3 | 7.8 | 7.8 | 7.8 | 51.428 | 29.331 | 107490000 | 7 | Lanosterol 14-alpha demethylase | CYP51A1 |
| 28.26369 | 21.73095 | 22.35485 | 19.75386 | 32 | 31 | 3 | 47 | 45.8 | 7.7 | 72.731 | 323.31 | 7413700000 | 65 | Nexilin | NEXN |
| 21.22594 | 23.8768 | 22.34611 | 19.51933 | 6 | 6 | 6 | 31.4 | 31.4 | 31.4 | 34.222 | 47.335 | 100720000 | 8 | MKI67 FHA domain-interacting nucleolar phosphoprotein | NIFK |
| 21.93632 | 21.6553 | 22.3427 | 22.53635 | 4 | 4 | 4 | 7.6 | 7.6 | 7.6 | 71.648 | 25.547 | 229840000 | 5 | A-kinase anchor protein 8-like | AKAP8L |
| 22.87429 | 22.46216 | 22.33922 | 22.92756 | 4 | 4 | 3 | 22.7 | 22.7 | 17.6 | 23.482 | 26.477 | 37897000 | 10 | Ras-related protein Rab-5C | RAB5C |
| 21.87189 | 22.59409 | 22.32721 | 22.50952 | 7 | 7 | 7 | 18.2 | 18.2 | 18.2 | 55.873 | 64.722 | 122750000 | 9 | Coiled-coil domain-containing protein 47 | CCDC47 |
| 21.70734 | 18.01942 | 22.3243 | 22.39514 | 5 | 5 | 5 | 13 | 13 | 13 | 47.431 | 38.354 | 79467000 | 9 | Torsin-1A-interacting protein 1 | TOR1AIP1 |
| 17.71565 | 22.43448 | 22.3121 | 22.51951 | 4 | 4 | 4 | 12.8 | 12.8 | 12.8 | 37.493 | 27.559 | 61267000 | 5 | Reticulocalbin-3 | RCN3 |
| 21.81761 | 23.0731 | 22.30975 | 22.78961 | 6 | 6 | 6 | 17.1 | 17.1 | 17.1 | 67.454 | 61.528 | 128470000 | 10 | Mitochondrial import receptor subunit TOM70 | TOMM70A |
| 24.05499 | 22.33497 | 22.30663 | 22.44769 | 15 | 15 | 15 | 16.5 | 16.5 | 16.5 | 118.23 | 126.6 | 522990000 | 18 | U2 snRNP-associated SURP motif-containing protein | U2SURP |
| 26.79835 | 24.48118 | 22.30327 | 23.79054 | 45 | 45 | 45 | 42.1 | 42.1 | 42.1 | 135.65 | 323.31 | 2058700000 | 67 | Structural maintenance of chromosomes protein 2 | SMC2 |
| 22.9225 | 24.57485 | 22.2916 | 22.32562 | 13 | 13 | 13 | 20.3 | 20.3 | 20.3 | 92.25 | 120 | 507140000 | 18 | Cell division cycle 5-like protein | CDC5L |
| 22.25658 | 22.48146 | 22.28805 | 23.53089 | 8 | 8 | 8 | 12.7 | 12.7 | 12.7 | 98.398 | 51.642 | 251990000 | 11 | Cytoplasmic aconitate hydratase | ACO1;IRP1 |
| 24.18283 | 23.16136 | 22.28611 | 24.31707 | 10 | 10 | 10 | 27.5 | 27.5 | 27.5 | 47.371 | 99.873 | 672940000 | 17 | Actin-related protein 3 | ACTR3 |
| 18.20321 | 21.719 | 22.27526 | 23.17257 | 4 | 4 | 4 | 18.7 | 18.7 | 18.7 | 29.41 | 26.044 | 57677000 | 8 | DNA fragmentation factor subunit alpha | DFFA |
| 21.94353 | 22.05199 | 22.27273 | 22.5588 | 7 | 7 | 7 | 14.8 | 14.8 | 14.8 | 54.548 | 43.75 | 125890000 | 8 | Xaa-Pro dipeptidase | PEPD |
| 24.27909 | 22.45926 | 22.27247 | 23.16183 | 13 | 13 | 13 | 16.7 | 16.7 | 16.7 | 98.117 | 125.83 | 606750000 | 16 | Eukaryotic translation initiation factor 4 gamma 2 | EIF4G2 |
| 21.41627 | 22.46998 | 22.25148 | 22.97034 | 6 | 6 | 6 | 20.7 | 20.7 | 20.7 | 46.648 | 41.409 | 135160000 | 9 | Sorting nexin-6;Sorting nexin-6, N-terminally processed | SNX6 |
| 23.91752 | 22.25327 | 22.2511 | 22.33131 | 17 | 17 | 17 | 17.3 | 17.3 | 17.3 | 124.2 | 125.23 | 411090000 | 17 | Actin-binding protein anillin | ANLN |
| 16.9887 | 21.68123 | 22.24389 | 22.12871 | 4 | 4 | 4 | 13.1 | 13.1 | 13.1 | 37.805 | 25.932 | 60612000 | 6 | Annexin;Annexin A7 | ANXA7 |
| 21.61831 | 23.37518 | 22.23219 | 19.05414 | 7 | 7 | 7 | 29 | 29 | 29 | 26.505 | 47.964 | 118800000 | 12 | Proteasome subunit alpha type-1 | PSMA1 |
| 21.90965 | 22.71789 | 22.23105 | 22.97568 | 7 | 7 | 7 | 18.2 | 18.2 | 18.2 | 47.267 | 43.052 | 121830000 | 8 | Glutathione reductase, mitochondrial | GSR |
| 22.29603 | 23.10446 | 22.22999 | 18.49943 | 6 | 6 | 6 | 19.4 | 19.4 | 19.4 | 43.215 | 43.125 | 141300000 | 9 | Methionine aminopeptidase 1 | METAP1 |
| 23.30563 | 22.18804 | 22.22005 | 22.15919 | 10 | 10 | 10 | 18.2 | 18.2 | 18.2 | 69.837 | 74.217 | 276700000 | 11 | Probable ATP-dependent RNA helicase DDX41 | DDX41 |
| 21.27594 | 21.08343 | 22.21659 | 22.65347 | 5 | 5 | 5 | 12.7 | 12.7 | 12.7 | 48.67 | 33.057 | 124270000 | 7 | Signal recognition particle 54 kDa protein | SRP54 |
| 23.26671 | 22.82445 | 22.21056 | 23.59503 | 9 | 9 | 9 | 14.7 | 14.7 | 14.7 | 82.091 | 65.967 | 555810000 | 15 | Vesicle-fusing ATPase | NSF |
| 22.13031 | 22.15922 | 22.20768 | 22.43774 | 5 | 5 | 4 | 14.7 | 14.7 | 11.9 | 46.097 | 35.743 | 148950000 | 10 | Sorting nexin-2 | SNX2 |
| 22.65799 | 21.3483 | 22.20699 | 21.74061 | 4 | 4 | 4 | 8.8 | 8.8 | 8.8 | 58.095 | 37.193 | 236780000 | 8 | ATP-dependent Clp protease ATP-binding subunit clpX-like, mitochondrial | DKFZp586J151;CLPX |
| 21.77386 | 22.84973 | 22.19994 | 22.8982 | 7 | 7 | 7 | 18.1 | 18.1 | 18.1 | 62.942 | 51.867 | 150750000 | 10 | Asparagine--tRNA ligase, cytoplasmic | NARS |
| 23.49692 | 23.42008 | 22.19568 | 22.24269 | 14 | 14 | 5 | 13.4 | 13.4 | 5.1 | 142.58 | 97.572 | 272520000 | 23 | Cytoplasmic FMR1-interacting protein 2 | CYFIP2 |
| 23.20932 | 22.86278 | 22.19481 | 22.35834 | 6 | 6 | 6 | 25.9 | 25.9 | 25.9 | 27.843 | 42.142 | 128520000 | 14 | Electron transfer flavoprotein subunit beta | ETFB |
| 21.81843 | 22.07904 | 22.189 | 22.47603 | 4 | 4 | 4 | 6.8 | 6.8 | 6.8 | 73.457 | 27.866 | 112610000 | 8 | ATP-dependent DNA helicase Q1 | RECQL |
| 20.51268 | 22.45623 | 22.1845 | 22.76965 | 7 | 7 | 7 | 4.5 | 4.5 | 4.5 | 218.52 | 46.717 | 185000000 | 15 | Cytoskeleton-associated protein 5 | CKAP5 |
| 22.65423 | 21.85901 | 22.17368 | 19.69731 | 4 | 4 | 4 | 11.1 | 11.1 | 11.1 | 37.567 | 27.848 | 168340000 | 7 | Casein kinase I isoform alpha | CSNK1A1 |
| 22.60815 | 20.86083 | 22.15915 | 21.48444 | 3 | 3 | 3 | 4.6 | 4.6 | 4.6 | 70.737 | 20.93 | 144740000 | 6 | Protein TBRG4 | TBRG4 |
| 17.67171 | 21.33751 | 22.15304 | 22.6657 | 4 | 4 | 4 | 11 | 11 | 11 | 41.9 | 25.1 | 52395000 | 7 | Sterol-4-alpha-carboxylate 3-dehydrogenase, decarboxylating | NSDHL |
| 23.54333 | 22.13151 | 22.15264 | 23.36386 | 3 | 3 | 3 | 21.9 | 21.9 | 21.9 | 17.692 | 22.915 | 56871000 | 8 | Cellular nucleic acid-binding protein | CNBP |
| 23.32485 | 23.41196 | 22.14722 | 19.97795 | 7 | 7 | 7 | 20.7 | 20.7 | 20.7 | 45.169 | 102.49 | 278730000 | 9 | Probable ATP-dependent RNA helicase DDX47 | DDX47 |
| 21.40707 | 23.09062 | 22.14681 | 19.63056 | 7 | 7 | 7 | 25.9 | 25.9 | 25.9 | 36.43 | 43.064 | 109180000 | 9 | PHD finger protein 6 | PHF6 |
| 25.12274 | 23.23056 | 22.14404 | 22.21018 | 11 | 11 | 11 | 35.7 | 35.7 | 35.7 | 40.282 | 252.96 | 789600000 | 23 | Twinfilin-1 | TWF1 |
| 22.42514 | 22.52617 | 22.13101 | 22.90204 | 4 | 4 | 4 | 31.7 | 31.7 | 31.7 | 17.827 | 25.791 | 32521000 | 6 | Deoxyuridine 5-triphosphate nucleotidohydrolase, mitochondrial | DUT |
| 21.18483 | 22.88547 | 22.12584 | 23.02685 | 11 | 11 | 11 | 4.8 | 4.8 | 4.8 | 290.46 | 71.37 | 255030000 | 13 | Probable ubiquitin carboxyl-terminal hydrolase FAF-X | USP9X |
| 22.10252 | 22.64537 | 22.12553 | 18.49019 | 7 | 7 | 7 | 31.3 | 31.3 | 31.3 | 39.385 | 44.355 | 113360000 | 8 | 4-hydroxyphenylpyruvate dioxygenase-like protein | HPDL |
| 22.521 | 21.71879 | 22.12133 | 22.12127 | 3 | 3 | 3 | 16.6 | 16.6 | 16.6 | 24.279 | 19.653 | 29469000 | 4 | GrpE protein homolog 1, mitochondrial;GrpE protein homolog | GRPEL1 |
| 22.65347 | 22.65777 | 22.11972 | 22.32207 | 7 | 7 | 4 | 20.7 | 20.7 | 15 | 57.886 | 53.804 | 213380000 | 11 | Importin subunit alpha-3 | KPNA4 |
| 25.97845 | 22.21978 | 22.11623 | 22.08314 | 16 | 16 | 16 | 29.6 | 29.6 | 29.6 | 81.074 | 240.25 | 1615600000 | 23 | FACT complex subunit SSRP1 | SSRP1 |
| 23.91798 | 22.53162 | 22.11254 | 22.49067 | 9 | 9 | 9 | 14.7 | 14.7 | 14.7 | 87.334 | 102.9 | 508850000 | 13 | Signal transducer and activator of transcription 1-alpha/beta | STAT1 |
| 22.63148 | 23.66936 | 22.11012 | 22.59894 | 9 | 9 | 9 | 14.3 | 14.3 | 14.3 | 83.629 | 89.711 | 401440000 | 15 | Ribosome biogenesis protein BOP1 | BOP1 |
| 22.14541 | 22.47266 | 22.10974 | 18.79382 | 7 | 7 | 7 | 25.4 | 25.4 | 25.4 | 31.192 | 45.388 | 154470000 | 10 | Four and a half LIM domains protein 3 | FHL3 |
| 21.78001 | 21.73153 | 22.10955 | 21.40826 | 5 | 5 | 5 | 19.9 | 19.9 | 19.9 | 38.926 | 31.719 | 105170000 | 8 | DDB1- and CUL4-associated factor 7 | DCAF7 |
| 18.34984 | 21.39713 | 22.1046 | 22.2204 | 5 | 5 | 5 | 10.1 | 10.1 | 10.1 | 75.775 | 34.302 | 50736000 | 7 | Transmembrane 9 superfamily member 2 | TM9SF2 |
| 21.6587 | 21.46049 | 22.10185 | 21.93481 | 4 | 4 | 4 | 6.4 | 6.4 | 6.4 | 73.321 | 26.566 | 199880000 | 5 | Palladin | PALLD |
| 23.37901 | 23.81792 | 22.09777 | 23.27113 | 6 | 6 | 4 | 14.3 | 14.3 | 9.3 | 55.102 | 48.693 | 383570000 | 13 | Histone deacetylase 1 | HDAC1 |
| 23.18418 | 22.36217 | 22.09713 | 22.77934 | 11 | 11 | 11 | 21.5 | 21.5 | 21.5 | 51.419 | 65.735 | 277480000 | 16 | Integrin-linked protein kinase | ILK |
| 21.54463 | 21.02925 | 22.09639 | 19.07553 | 5 | 4 | 4 | 28.7 | 20.5 | 20.5 | 22.276 | 26.915 | 88137000 | 7 | Acidic leucine-rich nuclear phosphoprotein 32 family member B | ANP32B |
| 21.76316 | 22.72132 | 22.09529 | 21.7911 | 6 | 6 | 6 | 9.6 | 9.6 | 9.6 | 80.521 | 40.585 | 231070000 | 11 | Double-strand break repair protein MRE11A | MRE11A |
| 23.10875 | 22.91061 | 22.09165 | 19.23408 | 9 | 9 | 9 | 12.2 | 12.2 | 12.2 | 102.45 | 75.67 | 351770000 | 9 | Periodic tryptophan protein 2 homolog | PWP2 |
| 27.8725 | 23.76122 | 22.08939 | 24.61487 | 12 | 12 | 3 | 35.2 | 35.2 | 9.9 | 21.364 | 97.062 | 3971200000 | 36 | Histone H1.2 | HIST1H1C |
| 20.89215 | 22.32031 | 22.08932 | 22.25989 | 7 | 7 | 7 | 19.5 | 19.5 | 19.5 | 58.042 | 52.79 | 124540000 | 11 | Serine/threonine-protein kinase PAK 2;PAK-2p27;PAK-2p34 | PAK2 |
| 22.59293 | 21.88107 | 22.08851 | 19.20573 | 4 | 4 | 4 | 13.2 | 13.2 | 13.2 | 29.842 | 23.774 | 29955000 | 8 | 28S ribosomal protein S15, mitochondrial | MRPS15 |
| 22.63646 | 22.11851 | 22.08014 | 21.92263 | 3 | 3 | 3 | 6.2 | 6.2 | 6.2 | 70.973 | 26.931 | 181930000 | 5 | E3 ubiquitin/ISG15 ligase TRIM25 | TRIM25 |
| 21.28059 | 21.41957 | 22.07708 | 22.13185 | 3 | 3 | 3 | 5.5 | 5.5 | 5.5 | 66.755 | 22.655 | 107710000 | 5 | NudC domain-containing protein 1 | NUDCD1 |
| 21.89393 | 21.83441 | 22.07316 | 20.19993 | 5 | 5 | 5 | 12.5 | 12.5 | 12.5 | 48.337 | 32.027 | 122710000 | 7 | G2/mitotic-specific cyclin-B1 | CCNB1 |
| 21.41885 | 21.52114 | 22.07048 | 23.89458 | 6 | 6 | 6 | 32 | 32 | 32 | 25.2 | 39.097 | 60235000 | 13 | Eukaryotic translation initiation factor 4H | EIF4H |
| 22.83673 | 22.53129 | 22.06996 | 22.36177 | 4 | 4 | 2 | 7.2 | 7.2 | 4 | 61.163 | 29.206 | 187630000 | 9 | YTH domain-containing family protein 2;YTH domain-containing family protein 1 | YTHDF2;YTHDF1 |
| 21.62221 | 22.05815 | 22.06933 | 22.79162 | 6 | 6 | 6 | 5.9 | 5.9 | 5.9 | 134.32 | 40.579 | 139490000 | 8 | Nodal modulator 1;Nodal modulator 2;Nodal modulator 3 | NOMO1;NOMO2;NOMO3 |
| 21.88803 | 19.3047 | 22.06497 | 23.30605 | 5 | 5 | 5 | 19.9 | 19.9 | 19.9 | 33.879 | 32.577 | 112460000 | 5 | Vacuolar protein sorting-associated protein VTA1 homolog | VTA1 |
| 21.82598 | 21.65425 | 22.0627 | 18.69242 | 5 | 5 | 5 | 25.3 | 25.3 | 25.3 | 32.66 | 39.985 | 95535000 | 10 | Inorganic pyrophosphatase | PPA1 |
| 21.75487 | 21.897 | 22.06016 | 18.68088 | 5 | 5 | 5 | 9.5 | 9.5 | 9.5 | 91.838 | 36.927 | 189170000 | 6 | Nuclear cap-binding protein subunit 1 | NCBP1 |
| 16.99676 | 21.11788 | 22.0593 | 22.78243 | 5 | 4 | 4 | 8.2 | 6.6 | 6.6 | 82.688 | 28.725 | 114310000 | 4 | Leucine-rich repeat flightless-interacting protein 1 | LRRFIP1 |
| 23.52456 | 21.33718 | 22.05384 | 21.78827 | 5 | 5 | 5 | 30.6 | 30.6 | 30.6 | 19.186 | 30.425 | 39513000 | 5 | Protein lin-7 homolog C | LIN7C |
| 21.92955 | 23.308 | 22.03983 | 20.27769 | 7 | 7 | 7 | 20.7 | 20.7 | 20.7 | 43.963 | 42.103 | 95422000 | 10 | p21-activated protein kinase-interacting protein 1 | PAK1IP1 |
| 21.61711 | 18.96776 | 22.03956 | 22.30221 | 4 | 4 | 4 | 13.2 | 13.2 | 13.2 | 39.829 | 33.998 | 94828000 | 9 | Vasodilator-stimulated phosphoprotein | VASP |
| 21.43654 | 22.70212 | 22.03752 | 22.15283 | 3 | 3 | 3 | 11.2 | 11.2 | 11.2 | 44.77 | 20.052 | 97846000 | 5 | SWI/SNF-related matrix-associated actin-dependent regulator of chromatin subfamily E member 1 | SMARCE1 |
| 23.31866 | 22.09194 | 22.03607 | 22.83142 | 10 | 10 | 9 | 17.9 | 17.9 | 16.8 | 80.622 | 79.237 | 442750000 | 11 | Ribosomal protein S6 kinase alpha-3 | RPS6KA3 |
| 18.29732 | 22.79998 | 22.03483 | 21.86041 | 3 | 3 | 3 | 6.1 | 6.1 | 6.1 | 70.098 | 40.916 | 178540000 | 3 | Signal recognition particle subunit SRP72 | SRP72 |
| 24.47389 | 22.14492 | 22.02042 | 19.77029 | 10 | 5 | 5 | 33.8 | 19.1 | 19.1 | 41.13 | 59.488 | 435970000 | 11 | Guanine nucleotide-binding protein G(i) subunit alpha-2 | GNAI2 |
| 18.6916 | 18.7968 | 22.02036 | 22.17606 | 4 | 4 | 4 | 10.1 | 10.1 | 10.1 | 68.283 | 22.107 | 60389000 | 3 | Phosphoglucomutase-2 | PGM2 |
| 22.26605 | 22.14833 | 22.02012 | 21.94335 | 3 | 3 | 3 | 15.8 | 15.8 | 15.8 | 21.77 | 18.106 | 23742000 | 5 | 28S ribosomal protein S23, mitochondrial | MRPS23 |
| 22.437 | 21.52497 | 22.01934 | 23.59742 | 7 | 7 | 7 | 14.3 | 14.3 | 14.3 | 83.541 | 91.888 | 351280000 | 6 | Nuclear pore complex protein Nup88 | NUP88 |
| 24.15027 | 18.5621 | 22.01934 | 22.17457 | 3 | 3 | 3 | 24.8 | 24.8 | 24.8 | 16.996 | 19.566 | 64390000 | 5 | DNA-directed RNA polymerases I, II, and III subunit RPABC3 | POLR2H |
| 22.26224 | 21.64504 | 22.00587 | 19.17737 | 5 | 5 | 5 | 21.8 | 21.8 | 21.8 | 28.993 | 29.93 | 65016000 | 9 | Endoplasmic reticulum resident protein 29 | ERP29 |
| 18.51135 | 21.76385 | 22.00007 | 18.48373 | 3 | 3 | 3 | 10.9 | 10.9 | 10.9 | 38.598 | 20.284 | 46654000 | 5 | Arfaptin-1 | ARFIP1 |
| 22.1354 | 24.32553 | 21.98992 | 22.4837 | 16 | 16 | 16 | 12.7 | 12.7 | 12.7 | 177.6 | 118.76 | 224740000 | 22 | Laminin subunit gamma-1 | LAMC1 |
| 18.42714 | 20.68594 | 21.98978 | 19.8559 | 3 | 3 | 3 | 10.6 | 10.6 | 10.6 | 44.017 | 19.168 | 21579000 | 3 | Cysteine desulfurase, mitochondrial | NFS1 |
| 17.88721 | 22.11623 | 21.98885 | 18.2411 | 3 | 3 | 3 | 11.9 | 11.9 | 11.9 | 38.47 | 21.066 | 58275000 | 6 | Lysophospholipid acyltransferase 7 | MBOAT7 |
| 20.86204 | 25.43822 | 21.98663 | 21.64068 | 11 | 11 | 11 | 28.8 | 28.8 | 28.8 | 43.614 | 97.357 | 277990000 | 16 | Cell growth-regulating nucleolar protein | LYAR |
| 24.97176 | 21.89682 | 21.98019 | 22.37356 | 6 | 6 | 6 | 48.2 | 48.2 | 48.2 | 12.405 | 46.768 | 110050000 | 11 | PHD finger-like domain-containing protein 5A | PHF5A |
| 21.14208 | 17.06293 | 21.97932 | 23.43415 | 5 | 5 | 5 | 27.3 | 27.3 | 27.3 | 21.225 | 40.649 | 259540000 | 10 | Ferritin heavy chain;Ferritin heavy chain, N-terminally processed;Ferritin | FTH1 |
| 21.63489 | 21.85472 | 21.96261 | 22.79668 | 3 | 3 | 3 | 15.9 | 15.9 | 15.9 | 21.493 | 20.414 | 41128000 | 11 | Receptor expression-enhancing protein 5 | REEP5 |
| 20.97512 | 21.61787 | 21.95674 | 19.2515 | 3 | 3 | 3 | 14.5 | 14.5 | 14.5 | 29.044 | 20.433 | 65482000 | 5 | Arfaptin-2 | ARFIP2 |
| 21.79598 | 21.16177 | 21.95674 | 21.18834 | 3 | 3 | 3 | 11 | 11 | 11 | 26.227 | 17.2 | 16535000 | 6 | Cleavage and polyadenylation specificity factor subunit 5 | NUDT21 |
| 22.26774 | 21.61921 | 21.95276 | 19.12427 | 3 | 3 | 3 | 6.8 | 6.8 | 6.8 | 51.964 | 20.317 | 152130000 | 4 | Fatty acid desaturase 1 | FADS1 |
| 24.12092 | 22.15536 | 21.9517 | 21.48281 | 6 | 6 | 6 | 24.9 | 24.9 | 24.9 | 24.665 | 45.047 | 339310000 | 13 | RNA-binding protein Raly | RALY |
| 16.59441 | 23.03429 | 21.94699 | 20.06214 | 4 | 4 | 4 | 11.7 | 11.7 | 11.7 | 40.063 | 24.651 | 53733000 | 4 | 3-5 exoribonuclease 1 | ERI1 |
| 21.81127 | 22.4542 | 21.94324 | 19.09468 | 5 | 5 | 5 | 14.1 | 14.1 | 14.1 | 45.834 | 36.609 | 104140000 | 6 | 28S ribosomal protein S9, mitochondrial | MRPS9 |
| 23.82454 | 17.95985 | 21.93758 | 23.49644 | 4 | 4 | 4 | 19.4 | 19.4 | 19.4 | 23.635 | 28.399 | 224430000 | 14 | Bone marrow stromal antigen 2 | BST2 |
| 23.92025 | 22.53993 | 21.93686 | 22.33726 | 7 | 7 | 7 | 25.9 | 25.9 | 25.9 | 39.343 | 100.71 | 376380000 | 12 | WD40 repeat-containing protein SMU1;WD40 repeat-containing protein SMU1, N-terminally processed | SMU1 |
| 17.64538 | 21.77068 | 21.93639 | 19.25196 | 3 | 3 | 3 | 11.4 | 11.4 | 11.4 | 39.609 | 20.324 | 38748000 | 5 | Enoyl-CoA delta isomerase 2, mitochondrial | ECI2 |
| 22.73818 | 21.30062 | 21.93301 | 19.84871 | 4 | 2 | 2 | 21.2 | 11.6 | 11.6 | 22.41 | 15.519 | 23110000 | 4 | GTP-binding protein SAR1b | SAR1B;DKFZp434B2017 |
| 18.31862 | 23.77676 | 21.93297 | 21.84311 | 9 | 9 | 9 | 22.3 | 22.3 | 22.3 | 54.178 | 59.679 | 107250000 | 10 | SPATS2-like protein | SPATS2L |
| 21.56834 | 18.827 | 21.93067 | 18.98777 | 3 | 3 | 3 | 7.5 | 7.5 | 7.5 | 44.875 | 19.56 | 89089000 | 4 | Transmembrane protein 43 | TMEM43 |
| 23.24153 | 22.05361 | 21.92987 | 18.72116 | 5 | 5 | 5 | 6 | 6 | 6 | 124.98 | 53.134 | 326910000 | 8 | cGMP-inhibited 3,5-cyclic phosphodiesterase A | PDE3A |
| 21.53664 | 21.593 | 21.92416 | 22.31437 | 3 | 3 | 3 | 12.6 | 12.6 | 12.6 | 37.646 | 20.308 | 113190000 | 5 | CD2 antigen cytoplasmic tail-binding protein 2 | CD2BP2 |
| 21.52344 | 23.84029 | 21.92053 | 20.39018 | 10 | 10 | 10 | 34.4 | 34.4 | 34.4 | 39.542 | 65.042 | 91812000 | 12 | Dimethyladenosine transferase 1, mitochondrial | TFB1M |
| 23.08171 | 21.75438 | 21.91994 | 21.7613 | 4 | 4 | 4 | 21.4 | 21.4 | 21.4 | 23.025 | 26.507 | 33056000 | 7 | Ras-related protein Rab-35 | RAB35 |
| 23.2303 | 22.2602 | 21.91229 | 19.95296 | 11 | 11 | 11 | 50.3 | 50.3 | 50.3 | 31.73 | 108.56 | 262480000 | 15 | Erythrocyte band 7 integral membrane protein | STOM |
| 22.66375 | 22.12405 | 21.90643 | 22.63808 | 3 | 3 | 3 | 6.8 | 6.8 | 6.8 | 59.711 | 28.514 | 183330000 | 10 | Splicing factor 1 | SF1 |
| 18.08324 | 17.07349 | 21.90434 | 22.4139 | 4 | 4 | 4 | 10.8 | 10.8 | 10.8 | 39.149 | 22.62 | 31738000 | 5 | Methylthioribose-1-phosphate isomerase | MRI1 |
| 17.27489 | 21.95426 | 21.9036 | 20.22706 | 3 | 3 | 3 | 10.8 | 10.8 | 10.8 | 42.15 | 44.567 | 68597000 | 7 | Nucleoporin Nup43 | NUP43 |
| 21.77925 | 21.82958 | 21.89855 | 18.35034 | 4 | 4 | 4 | 12 | 12 | 12 | 42.033 | 26.827 | 78055000 | 8 | Polymerase delta-interacting protein 2 | POLDIP2 |
| 23.33756 | 22.86816 | 21.89353 | 21.28014 | 5 | 5 | 5 | 13.3 | 13.3 | 13.3 | 47.46 | 127.15 | 263660000 | 12 | Actin-like protein 6A | ACTL6A |
| 23.45601 | 17.85373 | 21.88275 | 21.692 | 3 | 3 | 3 | 29.5 | 29.5 | 29.5 | 13.752 | 18.387 | 33970000 | 4 | Ras-related protein Rap-1b;Ras-related protein Rap-1b-like protein;Ras-related protein Rap-1A | RAP1B;RAP1A |
| 21.91962 | 21.78277 | 21.87245 | 21.41998 | 4 | 4 | 4 | 13.4 | 13.4 | 13.4 | 39.31 | 38.068 | 136940000 | 8 | U5 small nuclear ribonucleoprotein 40 kDa protein | SNRNP40;DKFZp434D199 |
| 22.25661 | 23.14912 | 21.86609 | 19.09291 | 4 | 4 | 4 | 10.8 | 10.8 | 10.8 | 47.843 | 28.802 | 228300000 | 5 | Translocation protein SEC63 homolog | SEC63 |
| 22.76909 | 22.66293 | 21.86151 | 22.59837 | 6 | 6 | 6 | 18.9 | 18.9 | 18.9 | 44.561 | 53.176 | 220980000 | 12 | Peptidyl-prolyl cis-trans isomerase FKBP8 | FKBP8 |
| 20.65944 | 22.05029 | 21.85806 | 19.6967 | 5 | 5 | 5 | 5.9 | 5.9 | 5.9 | 102.99 | 32.956 | 112020000 | 8 | Puromycin-sensitive aminopeptidase;Puromycin-sensitive aminopeptidase-like protein | NPEPPS;NPEPPSL1 |
| 24.33067 | 22.02662 | 21.85056 | 19.51711 | 7 | 5 | 4 | 36 | 26.6 | 23.2 | 22.774 | 31.179 | 59730000 | 7 | Ras-related protein Rab-13 | RAB13 |
| 20.93632 | 20.88666 | 21.84567 | 19.29019 | 4 | 4 | 4 | 13.5 | 13.5 | 13.5 | 34.012 | 24.465 | 68549000 | 6 | Tricarboxylate transport protein, mitochondrial | SLC25A1 |
| 17.93378 | 20.72309 | 21.84472 | 20.22868 | 6 | 6 | 6 | 18.4 | 18.4 | 18.4 | 42.1 | 36.284 | 40839000 | 7 | Serpin B5 | SERPINB5 |
| 23.9004 | 21.22099 | 21.84242 | 21.64974 | 6 | 6 | 6 | 12.8 | 12.8 | 12.8 | 52.535 | 41.849 | 363960000 | 9 | Death-associated protein kinase 3 | DAPK3 |
| 22.55861 | 22.31857 | 21.83784 | 22.72017 | 10 | 9 | 9 | 12.8 | 12.1 | 12.1 | 105.79 | 61.59 | 311730000 | 13 | Monofunctional C1-tetrahydrofolate synthase, mitochondrial | MTHFD1L |
| 21.45362 | 21.48935 | 21.81851 | 21.92611 | 3 | 3 | 3 | 10.1 | 10.1 | 10.1 | 45.034 | 19.2 | 68640000 | 5 | Protein AAR2 homolog | AAR2 |
| 25.40067 | 20.25026 | 21.81386 | 20.62266 | 31 | 31 | 29 | 18.2 | 18.2 | 17.3 | 238.27 | 323.31 | 1892400000 | 36 | Dedicator of cytokinesis protein 7 | DOCK7 |
| 29.00985 | 21.26052 | 21.81014 | 19.48295 | 40 | 26 | 0 | 83.1 | 58.5 | 0 | 32.876 | 323.31 | 1.1213E+10 | 75 | Tropomyosin alpha-1 chain | TPM1 |
| 21.80424 | 21.20866 | 21.80692 | 21.72908 | 4 | 4 | 4 | 8.3 | 8.3 | 8.3 | 47.405 | 26.202 | 116870000 | 7 | WD repeat-containing protein 18 | WDR18 |
| 23.57681 | 21.66319 | 21.804 | 22.03463 | 4 | 4 | 4 | 23.1 | 23.1 | 23.1 | 24.997 | 31.497 | 41138000 | 6 | Ras-related protein Rab-32 | RAB32 |
| 21.35132 | 22.80215 | 21.80247 | 22.53825 | 7 | 7 | 7 | 11.7 | 11.7 | 11.7 | 71.24 | 47.697 | 164660000 | 13 | GMP synthase [glutamine-hydrolyzing] | GMPS |
| 21.24516 | 21.227 | 21.80243 | 21.96737 | 5 | 4 | 4 | 13.4 | 9.9 | 9.9 | 44.551 | 23.748 | 79626000 | 7 | Serine hydroxymethyltransferase, cytosolic | SHMT1 |
| 22.61014 | 23.43326 | 21.80018 | 21.34468 | 6 | 6 | 6 | 15.8 | 15.8 | 15.8 | 59.498 | 52.871 | 194450000 | 9 | Spermatogenesis-associated serine-rich protein 2 | SPATS2 |
| 21.5108 | 21.66653 | 21.79816 | 22.13025 | 5 | 5 | 5 | 8.6 | 8.6 | 8.6 | 73.352 | 30.746 | 149490000 | 7 | Fermitin family homolog 2 | FERMT2 |
| 24.8274 | 18.39369 | 21.79808 | 19.47935 | 17 | 17 | 17 | 16.7 | 16.7 | 16.7 | 135.62 | 143.57 | 565340000 | 22 | Protein cordon-bleu | COBL |
| 21.40421 | 22.2613 | 21.79701 | 24.3676 | 11 | 11 | 11 | 12.5 | 12.5 | 12.5 | 119.16 | 91.216 | 370350000 | 16 | Kinesin-like protein KIF11 | KIF11 |
| 21.21401 | 23.11629 | 21.79229 | 22.92423 | 6 | 6 | 6 | 20.2 | 20.2 | 20.2 | 47.713 | 42.364 | 181700000 | 13 | Calpastatin | CAST |
| 23.42701 | 22.0889 | 21.79038 | 22.13618 | 6 | 6 | 6 | 6.8 | 6.8 | 6.8 | 104.99 | 40.077 | 300990000 | 9 | Inner centromere protein | INCENP |
| 22.41784 | 23.00263 | 21.7854 | 21.89981 | 14 | 14 | 14 | 9.8 | 9.8 | 9.8 | 188.15 | 104 | 257570000 | 19 | Transcription activator BRG1 | SMARCA4 |
| 21.28861 | 22.39853 | 21.78409 | 21.14227 | 5 | 5 | 2 | 11.3 | 11.3 | 4.4 | 61.257 | 31.601 | 84255000 | 6 | Phosphatidylinositol 4-phosphate 5-kinase type-1 alpha | PIP5K1A |
| 21.21187 | 23.26957 | 21.78361 | 22.94735 | 7 | 7 | 7 | 19.6 | 19.6 | 19.6 | 44.891 | 49.173 | 138120000 | 9 | Zinc finger CCCH domain-containing protein 15 | ZC3H15 |
| 21.44385 | 22.37526 | 21.78233 | 21.92053 | 5 | 5 | 5 | 9.4 | 9.4 | 9.4 | 68.6 | 47.88 | 152660000 | 7 | Eukaryotic peptide chain release factor GTP-binding subunit ERF3A;Eukaryotic peptide chain release factor GTP-binding subunit ERF3B | GSPT1;GSPT2 |
| 22.5633 | 21.54014 | 21.78145 | 19.54417 | 5 | 5 | 5 | 18.2 | 18.2 | 18.2 | 31.676 | 36.85 | 143810000 | 8 | V-type proton ATPase subunit d 1 | ATP6V0D1 |
| 20.42111 | 22.19259 | 21.77587 | 21.81656 | 11 | 11 | 11 | 5.7 | 5.7 | 5.7 | 242.37 | 81.939 | 216440000 | 11 | HEAT repeat-containing protein 1;HEAT repeat-containing protein 1, N-terminally processed | HEATR1 |
| 23.30772 | 24.5242 | 21.77346 | 24.24351 | 17 | 17 | 17 | 15.6 | 15.6 | 15.6 | 134.55 | 148.9 | 388670000 | 30 | Golgi apparatus protein 1 | GLG1 |
| 23.30786 | 21.72841 | 21.76418 | 19.96805 | 12 | 12 | 12 | 17.1 | 17.1 | 17.1 | 102.97 | 89.51 | 356170000 | 13 | ATP-dependent RNA helicase DDX42 | DDX42 |
| 21.8694 | 21.28957 | 21.75503 | 19.77309 | 3 | 3 | 3 | 30.1 | 30.1 | 30.1 | 10.713 | 19.281 | 90981000 | 5 | 5-AMP-activated protein kinase subunit gamma-1;5-AMP-activated protein kinase subunit gamma-2 | PRKAG1;PRKAG2 |
| 29.95435 | 22.36407 | 21.74891 | 22.84797 | 33 | 33 | 33 | 59.9 | 59.9 | 59.9 | 71.428 | 323.31 | 2.58E+10 | 90 | Drebrin | DBN1 |
| 20.86937 | 22.63839 | 21.74882 | 22.14105 | 5 | 5 | 5 | 10.5 | 10.5 | 10.5 | 66.829 | 34.754 | 72099000 | 12 | CREB-regulated transcription coactivator 3 | CRTC3 |
| 22.12025 | 21.61464 | 21.74731 | 19.53211 | 4 | 4 | 4 | 15.5 | 15.5 | 15.5 | 32.89 | 28.647 | 134950000 | 7 | THO complex subunit 6 homolog | THOC6 |
| 26.66804 | 21.83341 | 21.74435 | 21.68278 | 101 | 101 | 98 | 16.4 | 16.4 | 15.9 | 880.55 | 323.31 | 6287800000 | 106 | Dystonin | DST |
| 21.76681 | 21.62761 | 21.74431 | 22.18468 | 4 | 4 | 4 | 7 | 7 | 7 | 121.89 | 35.731 | 177100000 | 5 | Constitutive coactivator of PPAR-gamma-like protein 1 | FAM120A |
| 24.91998 | 22.34973 | 21.73802 | 23.1038 | 7 | 7 | 7 | 23 | 23 | 23 | 44.969 | 106.14 | 885560000 | 19 | Regulator of chromosome condensation | RCC1 |
| 22.57381 | 22.7224 | 21.7369 | 19.40635 | 9 | 9 | 9 | 33.8 | 33.8 | 33.8 | 33.824 | 70.723 | 189570000 | 14 | Spermidine synthase | SRM |
| 16.59701 | 21.75344 | 21.73306 | 20.74647 | 5 | 5 | 5 | 13.7 | 13.7 | 13.7 | 43.482 | 30.224 | 24358000 | 6 | 3-hydroxyisobutyryl-CoA hydrolase, mitochondrial | HIBCH |
| 21.02378 | 21.3338 | 21.73227 | 22.22841 | 5 | 5 | 5 | 8.6 | 8.6 | 8.6 | 78.839 | 31.164 | 88255000 | 7 | Thimet oligopeptidase | THOP1 |
| 22.01682 | 21.64636 | 21.72667 | 20.3166 | 8 | 8 | 8 | 28 | 28 | 28 | 23.181 | 52.193 | 110820000 | 13 | Clathrin light chain B | CLTB |
| 19.46136 | 23.56081 | 21.72372 | 22.29134 | 13 | 13 | 13 | 3.1 | 3.1 | 3.1 | 571.85 | 81.499 | 147030000 | 16 | E3 ubiquitin-protein ligase UBR4 | UBR4 |
| 22.83605 | 21.10827 | 21.71762 | 21.36009 | 5 | 5 | 5 | 20.9 | 20.9 | 20.9 | 22.222 | 30.848 | 26171000 | 5 | UMP-CMP kinase | CMPK1 |
| 20.43482 | 22.07914 | 21.7128 | 22.1501 | 11 | 11 | 11 | 5.2 | 5.2 | 5.2 | 266.38 | 69.419 | 185690000 | 15 | CCR4-NOT transcription complex subunit 1 | CNOT1 |
| 18.23402 | 21.82734 | 21.70734 | 20.74387 | 3 | 3 | 3 | 40.9 | 40.9 | 40.9 | 7.5737 | 19.562 | 52791000 | 4 | RNA-binding protein NOB1 | NOB1 |
| 20.71482 | 22.82621 | 21.70354 | 21.5382 | 7 | 7 | 7 | 12.7 | 12.7 | 12.7 | 87.198 | 57.714 | 155790000 | 7 | TBC1 domain family member 10B | TBC1D10B |
| 22.31526 | 21.62078 | 21.70024 | 22.22134 | 7 | 5 | 5 | 13.3 | 9 | 9 | 74.435 | 41.436 | 249680000 | 5 | Long-chain-fatty-acid--CoA ligase 4 | ACSL4 |
| 21.6309 | 23.19245 | 21.69183 | 21.32471 | 6 | 6 | 6 | 14.3 | 14.3 | 14.3 | 47.26 | 35.971 | 125420000 | 6 | DNA-directed RNA polymerase I subunit RPA49 | POLR1E |
| 18.8677 | 20.74263 | 21.68799 | 21.86929 | 4 | 4 | 4 | 14.6 | 14.6 | 14.6 | 32.895 | 26.48 | 71349000 | 6 | Prostaglandin reductase 1 | PTGR1 |
| 23.48787 | 21.3977 | 21.6874 | 21.86733 | 8 | 8 | 8 | 11.6 | 11.6 | 11.6 | 78.401 | 65.316 | 287030000 | 11 | ATP-binding cassette sub-family D member 3 | ABCD3 |
| 21.27532 | 22.92962 | 21.68419 | 23.7291 | 7 | 7 | 7 | 20.9 | 20.9 | 20.9 | 44.996 | 50.171 | 215700000 | 14 | Cartilage-associated protein | CRTAP |
| 21.97719 | 21.4247 | 21.68307 | 21.872 | 3 | 3 | 3 | 7.3 | 7.3 | 7.3 | 46.319 | 18.919 | 113620000 | 8 | Isovaleryl-CoA dehydrogenase, mitochondrial | IVD |
| 20.57117 | 21.02749 | 21.67982 | 18.22635 | 3 | 3 | 3 | 14.2 | 14.2 | 14.2 | 33.332 | 21.558 | 50693000 | 5 | Actin-related protein 10 | ACTR10 |
| 23.36266 | 21.80195 | 21.67917 | 22.01937 | 6 | 6 | 6 | 12.7 | 12.7 | 12.7 | 66.147 | 51.437 | 291550000 | 7 | Protein regulator of cytokinesis 1 | PRC1 |
| 23.70988 | 20.4957 | 21.67349 | 20.96346 | 13 | 13 | 13 | 9.7 | 9.7 | 9.7 | 210.13 | 156.23 | 837990000 | 22 | Pericentriolar material 1 protein | PCM1 |
| 22.32199 | 21.69715 | 21.6728 | 22.42245 | 7 | 7 | 7 | 8.7 | 8.7 | 8.7 | 106.1 | 50.95 | 265540000 | 8 | WD repeat-containing protein 3 | WDR3 |
| 24.635 | 20.51682 | 21.66683 | 20.83159 | 11 | 11 | 11 | 13.6 | 13.6 | 13.6 | 114.18 | 100.74 | 673510000 | 14 | Cytospin-B | SPECC1 |
| 20.25811 | 17.5366 | 21.6654 | 20.97099 | 9 | 9 | 9 | 3.6 | 3.6 | 3.6 | 308.57 | 55.9 | 117770000 | 11 | E3 ubiquitin-protein ligase UBR5 | UBR5 |
| 21.56584 | 21.95003 | 21.6651 | 18.39804 | 6 | 6 | 6 | 18.9 | 18.9 | 18.9 | 42.674 | 40.882 | 115650000 | 8 | Protein DEK | DEK |
| 23.86372 | 22.0021 | 21.66375 | 19.83364 | 3 | 3 | 3 | 25.6 | 25.6 | 25.6 | 14.726 | 18.331 | 47152000 | 6 | UPF0568 protein C14orf166 | C14orf166 |
| 22.11366 | 23.06991 | 21.66027 | 22.46481 | 4 | 4 | 2 | 9.6 | 9.6 | 5.4 | 49.005 | 27.134 | 178390000 | 7 | Septin-11 | 11. Sep |
| 21.7602 | 24.09192 | 21.65535 | 22.29788 | 9 | 9 | 9 | 11.4 | 11.4 | 11.4 | 91.809 | 62.155 | 318580000 | 12 | Pre-rRNA-processing protein TSR1 homolog | TSR1 |
| 18.28749 | 21.17877 | 21.65123 | 21.14911 | 3 | 3 | 3 | 5.3 | 5.3 | 5.3 | 76.12 | 24.629 | 66167000 | 6 | Nuclear fragile X mental retardation-interacting protein 2 | NUFIP2 |
| 26.50572 | 21.14656 | 21.65084 | 19.77269 | 25 | 25 | 25 | 34.5 | 34.5 | 34.5 | 116.44 | 323.31 | 2278400000 | 28 | Unconventional myosin-Ig;Minor histocompatibility antigen HA-2 | MYO1G |
| 21.62827 | 21.72005 | 21.64786 | 22.26488 | 5 | 5 | 5 | 6.9 | 6.9 | 6.9 | 109 | 29.3 | 173750000 | 6 | Exosome complex exonuclease RRP44 | DIS3 |
| 22.08083 | 21.71301 | 21.64482 | 22.60436 | 7 | 7 | 7 | 19.4 | 19.4 | 19.4 | 52.221 | 51.131 | 193940000 | 13 | Uridine 5-monophosphate synthase;Orotate phosphoribosyltransferase;Orotidine 5-phosphate decarboxylase | UMPS |
| 24.57364 | 21.45979 | 21.63688 | 21.81198 | 7 | 6 | 6 | 12.9 | 11 | 11 | 54.234 | 38.382 | 493610000 | 7 | Coronin-1B;Coronin | CORO1B |
| 21.58376 | 18.19145 | 21.63423 | 18.46525 | 3 | 3 | 3 | 14.9 | 14.9 | 14.9 | 25.377 | 16.728 | 38658000 | 4 | DnaJ homolog subfamily B member 6 | DNAJB6 |
| 22.26038 | 21.43964 | 21.6268 | 22.17837 | 7 | 7 | 6 | 7.6 | 7.6 | 6.6 | 113.08 | 75.654 | 214390000 | 15 | Golgin subfamily A member 2 | GOLGA2 |
| 25.44802 | 24.01521 | 21.62574 | 21.08427 | 28 | 28 | 28 | 26.8 | 26.8 | 26.8 | 150.25 | 323.31 | 730700000 | 42 | Elongator complex protein 1 | IKBKAP |
| 23.29109 | 23.08068 | 21.61962 | 19.36435 | 7 | 7 | 6 | 33 | 33 | 28.2 | 35.594 | 108.19 | 298040000 | 13 | Serine/threonine-protein phosphatase 2A catalytic subunit alpha isoform;Serine/threonine-protein phosphatase 2A catalytic subunit beta isoform | PPP2CA;PPP2CB |
| 21.01134 | 22.07032 | 21.61823 | 21.9813 | 3 | 3 | 3 | 8.8 | 8.8 | 8.8 | 53.203 | 27.274 | 127080000 | 6 | Nibrin | NBN |
| 22.39822 | 18.36659 | 21.61176 | 20.83007 | 4 | 4 | 4 | 20.9 | 20.9 | 20.9 | 25.125 | 44.089 | 132600000 | 6 | Acidic leucine-rich nuclear phosphoprotein 32 family member E | ANP32E |
| 22.06888 | 22.17898 | 21.60463 | 21.88933 | 3 | 3 | 3 | 18.3 | 18.3 | 18.3 | 18.819 | 17.059 | 25918000 | 4 | Transmembrane and coiled-coil domain-containing protein 1 | TMCO1 |
| 23.5715 | 21.5739 | 21.58545 | 21.62243 | 8 | 8 | 8 | 13 | 13 | 13 | 71.289 | 50.021 | 281630000 | 7 | ATP-binding cassette sub-family F member 2 | ABCF2 |
| 18.21592 | 20.21881 | 21.58458 | 19.81143 | 3 | 3 | 3 | 15.8 | 15.8 | 15.8 | 29.245 | 19.774 | 31409000 | 6 | Pyridoxal kinase | PDXK |
| 22.17667 | 22.89613 | 21.58422 | 20.17267 | 6 | 6 | 6 | 10 | 10 | 10 | 73.584 | 38.437 | 142840000 | 9 | Pumilio domain-containing protein KIAA0020 | KIAA0020 |
| 21.41741 | 22.80812 | 21.58344 | 22.55518 | 8 | 8 | 7 | 6.2 | 6.2 | 5.7 | 194.81 | 52.727 | 82159000 | 12 | DNA-directed RNA polymerase I subunit RPA1;DNA-directed RNA polymerase | POLR1A |
| 20.7086 | 21.88204 | 21.57819 | 21.85984 | 3 | 3 | 3 | 14 | 14 | 14 | 33.526 | 20.82 | 69082000 | 5 | Tetratricopeptide repeat protein 1 | TTC1 |
| 23.75797 | 21.05457 | 21.57681 | 21.33794 | 3 | 3 | 3 | 31.6 | 31.6 | 31.6 | 9.0495 | 20.214 | 35598000 | 5 | Programmed cell death protein 10 | PDCD10 |
| 18.82976 | 22.12089 | 21.57478 | 23.23997 | 3 | 3 | 3 | 9.5 | 9.5 | 9.5 | 36.152 | 24.968 | 71971000 | 5 | Na(+)/H(+) exchange regulatory cofactor NHE-RF2 | SLC9A3R2 |
| 19.97197 | 22.2974 | 21.55908 | 19.11947 | 4 | 4 | 4 | 6.4 | 6.4 | 6.4 | 84.471 | 29.362 | 107120000 | 6 | Cysteine--tRNA ligase, cytoplasmic | CARS |
| 21.84253 | 20.97589 | 21.53564 | 20.11249 | 6 | 6 | 6 | 30.8 | 30.8 | 30.8 | 38.287 | 54.469 | 91249000 | 9 | Secretory carrier-associated membrane protein 3 | SCAMP3 |
| 18.7494 | 21.52841 | 21.52143 | 22.14905 | 4 | 2 | 1 | 13.2 | 8 | 4.3 | 35.9 | 13.852 | 53408000 | 6 | Ig gamma-2 chain C region;Ig gamma-4 chain C region | IGHG2;IGHG4 |
| 20.70514 | 21.29821 | 21.5173 | 21.91627 | 3 | 3 | 2 | 11.6 | 11.6 | 6.3 | 29.642 | 20.315 | 43399000 | 7 | Phosphoribosyl pyrophosphate synthase-associated protein 2 | PRPSAP2 |
| 23.47914 | 24.35837 | 21.50426 | 18.50976 | 7 | 7 | 7 | 18.9 | 18.9 | 18.9 | 41.193 | 48.236 | 354280000 | 13 | Ribosome biogenesis regulatory protein homolog | RRS1 |
| 21.42772 | 22.35464 | 21.50223 | 19.15522 | 6 | 6 | 6 | 22.1 | 22.1 | 22.1 | 34.324 | 37.35 | 110300000 | 9 | Estradiol 17-beta-dehydrogenase 12 | HSD17B12 |
| 16.81694 | 21.34781 | 21.49311 | 17.84803 | 3 | 2 | 2 | 12.3 | 9.8 | 9.8 | 36.938 | 11.814 | 47364000 | 3 | Cyclin-dependent kinase 6 | CDK6 |
| 26.42089 | 21.46578 | 21.49272 | 19.38281 | 9 | 9 | 9 | 45.5 | 45.5 | 45.5 | 22.225 | 65.516 | 200840000 | 12 | Chromobox protein homolog 5 | CBX5 |
| 23.73789 | 22.11022 | 21.48429 | 19.15145 | 4 | 4 | 4 | 28.9 | 28.9 | 28.9 | 14.173 | 26.194 | 48125000 | 7 | NHP2-like protein 1;NHP2-like protein 1, N-terminally processed | NHP2L1 |
| 20.6352 | 18.45152 | 21.47887 | 23.45136 | 3 | 3 | 3 | 12.8 | 12.8 | 12.8 | 35.479 | 19.758 | 77459000 | 8 | Selenide, water dikinase 1 | SEPHS1 |
| 20.51653 | 21.94163 | 21.45879 | 19.09675 | 6 | 6 | 6 | 37.9 | 37.9 | 37.9 | 32.444 | 58.142 | 78675000 | 11 | Syntenin-1 | SDCBP |
| 20.1598 | 18.1124 | 21.45739 | 18.45716 | 3 | 3 | 3 | 24.2 | 24.2 | 24.2 | 22.16 | 31.8 | 55768000 | 4 | Endoplasmic reticulum-Golgi intermediate compartment protein 1 | ERGIC1 |
| 23.95056 | 21.19599 | 21.42404 | 20.5265 | 8 | 8 | 8 | 9.3 | 9.3 | 9.3 | 114.33 | 115.18 | 456470000 | 9 | Condensin complex subunit 3 | NCAPG |
| 22.62999 | 23.22756 | 21.42122 | 22.35925 | 3 | 3 | 3 | 9.9 | 9.9 | 9.9 | 27.571 | 16.546 | 46916000 | 8 | Translation machinery-associated protein 16 | TMA16 |
| 17.72707 | 18.00338 | 21.4173 | 18.93223 | 3 | 3 | 3 | 8 | 8 | 8 | 41.729 | 22.053 | 35266000 | 5 | tRNA pseudouridine synthase;tRNA pseudouridine synthase A, mitochondrial | PUS1 |
| 23.3098 | 21.22594 | 21.40078 | 18.79339 | 3 | 3 | 3 | 19.4 | 19.4 | 19.4 | 20.491 | 66.76 | 249330000 | 6 | Flotillin-1 | FLOT1 |
| 17.18902 | 21.33074 | 21.39477 | 19.01544 | 8 | 8 | 8 | 5.4 | 5.4 | 5.4 | 181.79 | 47.082 | 82986000 | 8 | 182 kDa tankyrase-1-binding protein | TNKS1BP1 |
| 20.66058 | 20.99693 | 21.39456 | 20.04644 | 3 | 3 | 3 | 7.5 | 7.5 | 7.5 | 38.792 | 19.036 | 49776000 | 6 | ATPase ASNA1 | ASNA1 |
| 21.30686 | 22.31642 | 21.39116 | 18.82709 | 10 | 4 | 4 | 33 | 15.4 | 15.4 | 34.834 | 26.946 | 86833000 | 7 | Ribose-phosphate pyrophosphokinase 1 | PRPS1 |
| 25.27711 | 20.87207 | 21.38084 | 20.64711 | 8 | 8 | 8 | 29.7 | 29.7 | 29.7 | 35.822 | 68.3 | 808570000 | 12 | Coiled-coil domain-containing protein 50 | CCDC50 |
| 22.60016 | 23.00893 | 21.37305 | 18.91244 | 8 | 8 | 8 | 43.6 | 43.6 | 43.6 | 31.821 | 113.44 | 171250000 | 15 | Exosome complex component RRP42 | EXOSC7 |
| 21.42388 | 21.65101 | 21.36986 | 18.79725 | 4 | 4 | 4 | 4.8 | 4.8 | 4.8 | 109.42 | 30.948 | 119910000 | 4 | ER membrane protein complex subunit 1 | EMC1 |
| 21.67926 | 21.31113 | 21.36175 | 21.80802 | 5 | 5 | 5 | 9.6 | 9.6 | 9.6 | 75.022 | 31.682 | 147260000 | 6 | Metastasis-associated protein MTA2 | MTA2 |
| 17.54869 | 18.30545 | 21.36089 | 19.32885 | 3 | 3 | 3 | 6.1 | 6.1 | 6.1 | 66.734 | 19.217 | 46732000 | 1 | Phospholipase A-2-activating protein | PLAA |
| 21.59796 | 21.87699 | 21.35821 | 22.48677 | 4 | 4 | 4 | 8.4 | 8.4 | 8.4 | 56.238 | 23.159 | 127140000 | 8 | Hydroxymethylglutaryl-CoA synthase, cytoplasmic | HMGCS1 |
| 18.18558 | 21.60526 | 21.3463 | 20.63476 | 4 | 4 | 4 | 5.1 | 5.1 | 5.1 | 105.58 | 27.199 | 140650000 | 5 | Squamous cell carcinoma antigen recognized by T-cells 3 | SART3 |
| 25.09587 | 21.85121 | 21.33734 | 20.34733 | 20 | 20 | 20 | 16.8 | 16.8 | 16.8 | 161.69 | 183.8 | 609520000 | 21 | CD109 antigen | CD109 |
| 17.02357 | 21.3009 | 21.32899 | 22.09838 | 5 | 5 | 5 | 19.9 | 19.9 | 19.9 | 42.315 | 35.5 | 52609000 | 6 | Protein phosphatase methylesterase 1 | PPME1 |
| 18.18548 | 18.5304 | 21.3274 | 20.75369 | 6 | 6 | 6 | 3.7 | 3.7 | 3.7 | 214.84 | 37.205 | 41229000 | 8 | Agrin;Agrin N-terminal 110 kDa subunit;Agrin C-terminal 110 kDa subunit;Agrin C-terminal 90 kDa fragment;Agrin C-terminal 22 kDa fragment | AGRN |
| 17.97985 | 17.70398 | 21.32147 | 20.16719 | 4 | 2 | 2 | 2.1 | 1.1 | 1.1 | 222.86 | 11.396 | 20097000 | 3 | Chromodomain-helicase-DNA-binding protein 3 | CHD3 |
| 20.66492 | 23.93328 | 21.32042 | 21.29154 | 5 | 5 | 5 | 14.2 | 14.2 | 14.2 | 46.977 | 40.018 | 129220000 | 9 | Exosome complex component RRP45 | EXOSC9 |
| 18.18939 | 18.41262 | 21.317 | 19.04584 | 3 | 2 | 2 | 4.2 | 2.8 | 2.8 | 76.932 | 10.914 | 158760000 | 2 | Putative pre-mRNA-splicing factor ATP-dependent RNA helicase DHX16 | DHX16 |
| 21.3093 | 21.48576 | 21.30919 | 21.36325 | 3 | 3 | 3 | 12.6 | 12.6 | 12.6 | 31.17 | 17.903 | 14474000 | 4 | Tumor protein D52 | TPD52 |
| 23.17612 | 22.31396 | 21.2976 | 21.62123 | 7 | 7 | 7 | 35.4 | 35.4 | 35.4 | 29.702 | 60.142 | 151920000 | 12 | Signal recognition particle receptor subunit beta | SRPRB |
| 19.28153 | 18.46397 | 21.29513 | 19.53718 | 8 | 3 | 3 | 24.2 | 14.6 | 14.6 | 42.293 | 24.101 | 45110000 | 6 | Beta-centractin | ACTR1B |
| 23.60456 | 23.9597 | 21.28376 | 21.58513 | 15 | 15 | 14 | 12 | 12 | 12 | 143.7 | 111.78 | 338930000 | 22 | RRP12-like protein | RRP12 |
| 22.0126 | 23.72837 | 21.28082 | 19.34271 | 8 | 8 | 8 | 9.3 | 9.3 | 9.3 | 128.18 | 64.124 | 179840000 | 12 | Replication factor C subunit 1 | RFC1 |
| 21.76531 | 22.8515 | 21.27821 | 21.08848 | 4 | 4 | 4 | 9.7 | 9.7 | 9.7 | 57.181 | 36.519 | 116160000 | 7 | Pleiotropic regulator 1 | PLRG1 |
| 20.71297 | 21.47526 | 21.2752 | 20.18813 | 3 | 3 | 3 | 10.2 | 10.2 | 10.2 | 44.047 | 18.147 | 52348000 | 5 | Queuine tRNA-ribosyltransferase | QTRT1 |
| 23.75715 | 21.16723 | 21.25153 | 19.16447 | 3 | 3 | 3 | 28 | 28 | 28 | 14.585 | 18.915 | 44654000 | 5 | Splicing factor 3B subunit 6 | SF3B6 |
| 21.24447 | 18.63699 | 21.24847 | 20.25257 | 6 | 6 | 6 | 26.8 | 26.8 | 26.8 | 29.506 | 43.692 | 66008000 | 8 | Proteasome activator complex subunit 3 | PSME3 |
| 18.23491 | 22.14824 | 21.244 | 19.66952 | 3 | 3 | 3 | 7.4 | 7.4 | 7.4 | 52.788 | 18.323 | 86980000 | 2 | RNA polymerase II-associated factor 1 homolog | PAF1 |
| 23.74796 | 21.33325 | 21.23116 | 21.57321 | 4 | 4 | 4 | 25.3 | 25.3 | 25.3 | 21.508 | 45.176 | 40974000 | 6 | Core-binding factor subunit beta | CBFB |
| 23.03009 | 19.24531 | 21.22641 | 19.75985 | 4 | 4 | 4 | 31.7 | 31.7 | 31.7 | 18.469 | 26.434 | 20790000 | 4 | Vacuolar protein sorting-associated protein 28 homolog | VPS28 |
| 20.77555 | 23.3534 | 21.22488 | 19.4335 | 7 | 7 | 7 | 35.1 | 35.1 | 35.1 | 27.56 | 50.642 | 114050000 | 15 | mRNA turnover protein 4 homolog | MRTO4 |
| 22.05864 | 18.12783 | 21.21904 | 19.96127 | 3 | 3 | 3 | 12.3 | 12.3 | 12.3 | 36.049 | 27.144 | 84788000 | 4 | PDZ domain-containing protein GIPC1 | GIPC1 |
| 22.85857 | 20.79337 | 21.20926 | 18.87183 | 5 | 5 | 3 | 10 | 10 | 7.8 | 44.424 | 41.283 | 150370000 | 9 | Dual specificity mitogen-activated protein kinase kinase 2 | MAP2K2 |
| 21.0764 | 24.3664 | 21.20425 | 19.71197 | 4 | 4 | 4 | 29.7 | 29.7 | 29.7 | 24.992 | 41.314 | 110430000 | 8 | UPF0488 protein C8orf33 | C8orf33 |
| 19.87617 | 22.56813 | 21.20311 | 19.71096 | 5 | 5 | 5 | 11.2 | 11.2 | 11.2 | 47.144 | 35.801 | 48544000 | 5 | Suppressor of SWI4 1 homolog | PPAN |
| 22.74585 | 17.90063 | 21.19394 | 21.31113 | 6 | 6 | 6 | 14.5 | 14.5 | 14.5 | 53.137 | 44.566 | 146390000 | 6 | Flotillin-2 | FLOT2 |
| 22.10601 | 21.8066 | 21.19292 | 19.87088 | 5 | 5 | 5 | 23.6 | 23.6 | 23.6 | 34.559 | 38.71 | 78205000 | 7 | Mitochondrial import receptor subunit TOM34 | TOMM34 |
| 20.99079 | 23.02788 | 21.18991 | 19.09999 | 5 | 5 | 5 | 8.4 | 8.4 | 8.4 | 78.863 | 37.512 | 190960000 | 5 | U3 small nucleolar ribonucleoprotein protein MPP10 | MPHOSPH10 |
| 20.35477 | 20.3955 | 21.18954 | 20.34544 | 4 | 4 | 4 | 3.5 | 3.5 | 3.5 | 157.46 | 23.127 | 52120000 | 5 | GTPase-activating protein and VPS9 domain-containing protein 1 | GAPVD1 |
| 23.06719 | 21.22899 | 21.18441 | 23.33361 | 5 | 5 | 5 | 31.6 | 31.6 | 31.6 | 17.605 | 29.424 | 42242000 | 9 | Sorcin | SRI |
| 20.83946 | 21.94546 | 21.18362 | 18.14081 | 3 | 3 | 3 | 17.6 | 17.6 | 17.6 | 25.314 | 18.894 | 53310000 | 3 | Uroporphyrinogen decarboxylase | UROD |
| 21.68714 | 21.2588 | 21.18132 | 19.48177 | 3 | 3 | 3 | 9.8 | 9.8 | 9.8 | 32.992 | 20.196 | 81795000 | 5 | Pre-mRNA-splicing factor ISY1 homolog | ISY1 |
| 27.21336 | 23.9385 | 21.17316 | 23.49448 | 39 | 39 | 39 | 39.3 | 39.3 | 39.3 | 119.91 | 323.31 | 2729800000 | 66 | FACT complex subunit SPT16 | SUPT16H |
| 21.10181 | 22.40673 | 21.16907 | 19.65122 | 4 | 4 | 4 | 18.7 | 18.7 | 18.7 | 32.118 | 31.701 | 72528000 | 9 | Purine nucleoside phosphorylase | PNP |
| 20.99838 | 21.6289 | 21.15752 | 17.99225 | 3 | 3 | 3 | 12.4 | 12.4 | 12.4 | 31.484 | 22.95 | 74237000 | 5 | RRP15-like protein | RRP15 |
| 22.00344 | 20.3595 | 21.14588 | 19.05223 | 4 | 4 | 4 | 15.2 | 15.2 | 15.2 | 39.134 | 25.273 | 57125000 | 6 | Cell division cycle protein 123 homolog | CDC123 |
| 20.7091 | 21.23526 | 21.14065 | 19.5972 | 5 | 5 | 2 | 24.2 | 24.2 | 9.4 | 30.375 | 41.856 | 59204000 | 7 | Carbonyl reductase [NADPH] 1 | CBR1 |
| 24.43981 | 21.08401 | 21.12786 | 20.14757 | 9 | 5 | 2 | 32.9 | 17.1 | 9.1 | 37.331 | 96.466 | 472170000 | 11 | Guanine nucleotide-binding protein G(I)/G(S)/G(T) subunit beta-2 | GNB2 |
| 20.0355 | 22.88859 | 21.12515 | 21.31849 | 4 | 4 | 4 | 10.8 | 10.8 | 10.8 | 49.601 | 29.436 | 69921000 | 4 | Bystin | BYSL |
| 21.6917 | 22.59955 | 21.10649 | 19.19501 | 5 | 5 | 5 | 19.1 | 19.1 | 19.1 | 32.996 | 42.696 | 67881000 | 10 | ATP synthase subunit gamma, mitochondrial;ATP synthase subunit gamma | ATP5C1 |
| 21.11121 | 21.70506 | 21.09578 | 18.87592 | 4 | 4 | 4 | 7.3 | 7.3 | 7.3 | 79.621 | 29.303 | 120740000 | 6 | Dynamin-1-like protein | DNM1L |
| 22.9018 | 22.61646 | 21.09249 | 19.72855 | 6 | 6 | 6 | 22.3 | 22.3 | 22.3 | 35.386 | 78.001 | 198480000 | 10 | Retinol dehydrogenase 11 | RDH11 |
| 30.00785 | 21.0551 | 21.09075 | 20.47853 | 29 | 29 | 28 | 52.7 | 52.7 | 50.8 | 53.248 | 323.31 | 1.9693E+10 | 67 | Coronin-1C;Coronin | CORO1C |
| 21.821 | 21.02972 | 21.08245 | 18.24986 | 5 | 5 | 5 | 26.9 | 26.9 | 26.9 | 25.835 | 56.455 | 96626000 | 8 | Coiled-coil domain-containing protein 124 | CCDC124 |
| 21.58784 | 21.56076 | 21.07803 | 19.46982 | 4 | 4 | 4 | 16.9 | 16.9 | 16.9 | 35.716 | 59.506 | 76635000 | 8 | Thymidylate synthase | TYMS |
| 23.87989 | 22.85964 | 21.07685 | 23.18582 | 19 | 19 | 19 | 14.6 | 14.6 | 14.6 | 167.93 | 135.1 | 281470000 | 25 | DNA (cytosine-5)-methyltransferase 1;Cytosine-specific methyltransferase | DNMT1 |
| 23.60116 | 21.81937 | 21.06789 | 20.30191 | 3 | 3 | 3 | 15.7 | 15.7 | 15.7 | 24.551 | 22.08 | 34363000 | 8 | DNA-directed RNA polymerases I, II, and III subunit RPABC1 | POLR2E |
| 21.10879 | 21.30268 | 21.0557 | 19.43273 | 5 | 5 | 5 | 14.9 | 14.9 | 14.9 | 33.249 | 32.163 | 57101000 | 7 | 28S ribosomal protein S2, mitochondrial | MRPS2 |
| 26.10863 | 21.42645 | 21.05006 | 21.2307 | 20 | 20 | 20 | 30.8 | 30.8 | 30.8 | 89.726 | 295.79 | 1726000000 | 25 | E3 ubiquitin-protein ligase UHRF1 | UHRF1 |
| 23.23137 | 22.89521 | 21.04267 | 21.37581 | 7 | 7 | 7 | 12.8 | 12.8 | 12.8 | 70.182 | 47.685 | 191340000 | 10 | Nuclear RNA export factor 1 | NXF1 |
| 20.2764 | 21.46383 | 21.03745 | 20.51911 | 5 | 5 | 5 | 21.8 | 21.8 | 21.8 | 31.54 | 39.598 | 58875000 | 10 | Phosphatidylinositol transfer protein beta isoform | PITPNB |
| 21.88453 | 21.33347 | 21.01359 | 21.39289 | 6 | 6 | 6 | 6.8 | 6.8 | 6.8 | 125.82 | 40.913 | 115740000 | 7 | Fibronectin type-III domain-containing protein 3A | FNDC3A |
| 18.68936 | 18.70078 | 21.01154 | 20.26451 | 3 | 3 | 3 | 12.2 | 12.2 | 12.2 | 36.405 | 32.046 | 59065000 | 3 | WD repeat-containing protein 74 | WDR74 |
| 17.21386 | 21.19189 | 21.00826 | 19.76777 | 3 | 3 | 3 | 10.1 | 10.1 | 10.1 | 34.032 | 18.616 | 41976000 | 4 | 39S ribosomal protein L39, mitochondrial | MRPL39 |
| 24.74919 | 21.04906 | 21.00635 | 21.26006 | 9 | 9 | 9 | 22 | 22 | 22 | 56.811 | 121.43 | 551720000 | 10 | Intestinal-type alkaline phosphatase | ALPI |
| 24.66519 | 20.19099 | 21.00058 | 19.47273 | 8 | 8 | 8 | 16.5 | 16.5 | 16.5 | 62.788 | 71.018 | 575840000 | 11 | Anthrax toxin receptor 1 | ANTXR1 |
| 17.7546 | 17.60443 | 20.99673 | 19.73201 | 3 | 3 | 3 | 11.7 | 11.7 | 11.7 | 36.899 | 17.299 | 22531000 | 2 | Regulation of nuclear pre-mRNA domain-containing protein 1B | RPRD1B |
| 23.38798 | 20.95978 | 20.99445 | 21.09719 | 7 | 6 | 6 | 17 | 15.4 | 15.4 | 62.233 | 48.475 | 230280000 | 8 | Copine-8 | CPNE8 |
| 22.91017 | 21.28664 | 20.99072 | 19.74068 | 5 | 5 | 5 | 6.1 | 6.1 | 6.1 | 103.7 | 41.527 | 239900000 | 5 | Calcium homeostasis endoplasmic reticulum protein | CHERP |
| 22.74665 | 20.23281 | 20.98607 | 21.42634 | 3 | 3 | 3 | 15.1 | 15.1 | 15.1 | 21.539 | 18.525 | 27685000 | 4 | ADP-ribosylation factor-like protein 8B;ADP-ribosylation factor-like protein 8A | ARL8B;ARL8A |
| 20.39142 | 22.23034 | 20.98211 | 21.28025 | 3 | 3 | 3 | 7.3 | 7.3 | 7.3 | 62.027 | 19.536 | 72608000 | 3 | DnaJ homolog subfamily C member 21 | DNAJC21 |
| 19.82255 | 19.63948 | 20.95617 | 19.21303 | 4 | 4 | 4 | 22.3 | 22.3 | 22.3 | 26.87 | 23.8 | 39005000 | 7 | Proteasome activator complex subunit 1 | PSME1 |
| 22.30037 | 17.9347 | 20.9502 | 20.32159 | 4 | 4 | 4 | 6.1 | 6.1 | 6.1 | 60.801 | 25.192 | 107520000 | 4 | Tyrosine-protein kinase Yes;Tyrosine-protein kinase Fyn;Proto-oncogene tyrosine-protein kinase Src | YES1;FYN;SRC |
| 23.83161 | 20.30919 | 20.94084 | 19.89644 | 7 | 7 | 7 | 25.9 | 25.9 | 25.9 | 40.569 | 184.38 | 313370000 | 5 | Tropomodulin-1 | TMOD1 |
| 19.92877 | 21.25107 | 20.94056 | 22.35272 | 3 | 3 | 3 | 5.8 | 5.8 | 5.8 | 47.808 | 16.829 | 67194000 | 5 | Peptidyl-prolyl cis-trans isomerase FKBP9 | FKBP9 |
| 17.78637 | 17.422 | 20.93603 | 20.37784 | 3 | 1 | 1 | 7.4 | 3.1 | 3.1 | 47.711 | 11.115 | 131870000 | 1 | Putative heat shock protein HSP 90-alpha A4 | HSP90AA4P |
| 25.19565 | 20.58637 | 20.93005 | 21.11401 | 6 | 4 | 4 | 39.8 | 28.9 | 28.9 | 18.736 | 29.982 | 113390000 | 6 | Cofilin-2 | CFL2 |
| 18.01636 | 20.64051 | 20.92506 | 20.96219 | 3 | 3 | 3 | 7.3 | 7.3 | 7.3 | 48.475 | 18.275 | 27901000 | 3 | L-2-hydroxyglutarate dehydrogenase, mitochondrial | L2HGDH |
| 23.08255 | 22.20129 | 20.92412 | 23.96992 | 3 | 3 | 3 | 10 | 10 | 10 | 60.593 | 67.004 | 239560000 | 9 | Golgi resident protein GCP60 | ACBD3 |
| 18.3143 | 17.82703 | 20.91605 | 20.73983 | 6 | 6 | 6 | 3.8 | 3.8 | 3.8 | 218.97 | 34.855 | 29275000 | 6 | Probable helicase with zinc finger domain | HELZ |
| 28.05502 | 22.39095 | 20.90353 | 20.67943 | 62 | 62 | 49 | 40 | 40 | 32.4 | 174.38 | 323.31 | 4777500000 | 75 | DNA topoisomerase 2-alpha | TOP2A |
| 19.70176 | 22.48062 | 20.89763 | 21.60282 | 8 | 8 | 8 | 2.7 | 2.7 | 2.7 | 399.73 | 52.795 | 91745000 | 12 | Laminin subunit alpha-5 | LAMA5 |
| 20.53555 | 20.66848 | 20.87912 | 21.89844 | 3 | 3 | 3 | 4.8 | 4.8 | 4.8 | 76.096 | 19.511 | 80484000 | 4 | Cleft lip and palate transmembrane protein 1 | CLPTM1 |
| 23.71607 | 21.60734 | 20.86695 | 19.3235 | 3 | 3 | 3 | 22.6 | 22.6 | 22.6 | 21.701 | 18.245 | 33647000 | 3 | Protein LSM12 homolog | LSM12 |
| 22.72351 | 21.83043 | 20.86051 | 20.52318 | 8 | 8 | 8 | 8.5 | 8.5 | 8.5 | 160.99 | 62.88 | 153180000 | 8 | Arf-GAP with Rho-GAP domain, ANK repeat and PH domain-containing protein 1 | ARAP1 |
| 23.85529 | 22.11493 | 20.8443 | 23.95784 | 14 | 6 | 6 | 52.9 | 20.2 | 20.2 | 37.186 | 74.856 | 496880000 | 16 | Serine/threonine-protein phosphatase PP1-beta catalytic subunit;Serine/threonine-protein phosphatase | PPP1CB |
| 24.25811 | 21.88387 | 20.84391 | 20.04281 | 10 | 10 | 10 | 40.5 | 40.5 | 40.5 | 32.253 | 92.449 | 422510000 | 13 | WD repeat-containing protein 61;WD repeat-containing protein 61, N-terminally processed | WDR61 |
| 18.24639 | 18.66657 | 20.84161 | 18.18242 | 4 | 2 | 2 | 8.7 | 6.3 | 6.3 | 40.763 | 14.237 | 26277000 | 2 | Dual specificity mitogen-activated protein kinase kinase 1 | MAP2K1 |
| 18.7958 | 20.34679 | 20.81886 | 20.46589 | 3 | 3 | 3 | 11.4 | 11.4 | 11.4 | 30.158 | 18.204 | 17334000 | 4 | Serine/threonine-protein phosphatase 2A activator | PPP2R4 |
| 22.96021 | 20.8433 | 20.80002 | 20.56923 | 3 | 3 | 3 | 10.9 | 10.9 | 10.9 | 37.19 | 24.462 | 185570000 | 6 | Protein FAM98B | FAM98B |
| 21.09371 | 21.78796 | 20.78542 | 22.48207 | 4 | 4 | 4 | 14 | 14 | 14 | 37.481 | 25.604 | 69276000 | 7 | Fatty acyl-CoA reductase 1 | FAR1 |
| 22.37806 | 20.94757 | 20.78437 | 19.26035 | 4 | 4 | 4 | 21.7 | 21.7 | 21.7 | 24.176 | 43.296 | 113020000 | 4 | Splicing factor 45 | RBM17 |
| 21.49799 | 21.32053 | 20.75987 | 18.86391 | 3 | 3 | 3 | 16.3 | 16.3 | 16.3 | 26.888 | 30.681 | 56426000 | 4 | THO complex subunit 4 | ALYREF |
| 25.03095 | 21.35159 | 20.74927 | 20.55112 | 18 | 18 | 4 | 25.2 | 25.2 | 6.9 | 109.93 | 237.11 | 594170000 | 18 | LIM and calponin homology domains-containing protein 1 | LIMCH1 |
| 24.17364 | 22.03265 | 20.74821 | 20.79543 | 28 | 15 | 15 | 24.3 | 13.5 | 13.5 | 130.48 | 118.7 | 349280000 | 17 | DNA topoisomerase 2;DNA topoisomerase 2-beta | TOP2B |
| 21.6546 | 17.59879 | 20.73917 | 18.62641 | 3 | 3 | 3 | 37.7 | 37.7 | 37.7 | 7.6977 | 16.98 | 12696000 | 3 | Nicotinamide N-methyltransferase | USP15;NNMT |
| 21.704 | 21.5063 | 20.73355 | 22.18861 | 4 | 4 | 4 | 20.7 | 20.7 | 20.7 | 31.387 | 23.571 | 18199000 | 7 | Enoyl-CoA hydratase, mitochondrial | ECHS1 |
| 23.13549 | 20.98183 | 20.73198 | 21.26511 | 9 | 9 | 9 | 31.8 | 31.8 | 31.8 | 32.004 | 62.844 | 184190000 | 9 | Serine/threonine-protein phosphatase PGAM5, mitochondrial | PGAM5 |
| 22.04733 | 20.45954 | 20.72692 | 20.55037 | 5 | 5 | 5 | 9 | 9 | 9 | 66.558 | 32.964 | 99318000 | 5 | Signal recognition particle receptor subunit alpha | SRPR |
| 20.58509 | 18.29108 | 20.71557 | 21.25776 | 3 | 3 | 3 | 18.1 | 18.1 | 18.1 | 21.732 | 17.491 | 11059000 | 4 | AP-3 complex subunit sigma-1 | AP3S1 |
| 21.72883 | 18.06766 | 20.71099 | 18.70863 | 6 | 6 | 6 | 28.9 | 28.9 | 28.9 | 31.323 | 50.002 | 67445000 | 6 | Borealin | CDCA8 |
| 21.25442 | 17.87577 | 20.70024 | 18.85197 | 4 | 4 | 4 | 5.7 | 5.7 | 5.7 | 108.66 | 26.84 | 68271000 | 4 | Thyroid hormone receptor-associated protein 3 | THRAP3 |
| 19.41458 | 16.91172 | 20.68834 | 18.33286 | 4 | 2 | 2 | 22.5 | 12.4 | 12.4 | 24.033 | 13.747 | 23821000 | 4 | High mobility group protein B2 | HMGB2 |
| 22.69884 | 20.86355 | 20.66943 | 21.29799 | 4 | 4 | 4 | 23.6 | 23.6 | 23.6 | 20.546 | 27.594 | 27241000 | 4 | Actin-related protein 2/3 complex subunit 3 | ARPC3 |
| 23.6525 | 21.45181 | 20.66796 | 20.37178 | 12 | 12 | 12 | 8.8 | 8.8 | 8.8 | 186.49 | 84.78 | 222200000 | 11 | Kinesin-like protein KIF14 | KIF14 |
| 20.73132 | 21.25113 | 20.66327 | 18.20276 | 4 | 4 | 4 | 17 | 17 | 17 | 35.236 | 25.958 | 66391000 | 7 | Hydroxyacyl-coenzyme A dehydrogenase, mitochondrial | HADH |
| 21.71708 | 24.1141 | 20.65517 | 20.9151 | 9 | 9 | 9 | 12.9 | 12.9 | 12.9 | 77.672 | 59.53 | 342680000 | 12 | NF-kappa-B-repressing factor | NKRF |
| 23.44594 | 19.96452 | 20.6479 | 18.89507 | 7 | 7 | 7 | 10.7 | 10.7 | 10.7 | 109.13 | 159.36 | 315310000 | 7 | Unconventional myosin-XIX | MYO19 |
| 23.32196 | 20.40811 | 20.64676 | 20.53003 | 4 | 4 | 4 | 6.1 | 6.1 | 6.1 | 110.13 | 113.66 | 283480000 | 6 | E3 ubiquitin-protein ligase MIB1 | MIB1 |
| 20.29379 | 23.13576 | 20.64218 | 19.97206 | 4 | 4 | 4 | 16.2 | 16.2 | 16.2 | 28.235 | 24.863 | 59808000 | 6 | Exosome complex component MTR3 | EXOSC6 |
| 26.03004 | 18.6637 | 20.62605 | 19.72502 | 19 | 19 | 19 | 28.9 | 28.9 | 28.9 | 75.276 | 123.5 | 1335700000 | 24 | Annexin A6;Annexin | ANXA6 |
| 21.86178 | 20.68663 | 20.61522 | 18.85458 | 4 | 3 | 3 | 14.8 | 11.3 | 11.3 | 37.806 | 50.107 | 88220000 | 5 | DnaJ homolog subfamily B member 4 | DNAJB4 |
| 18.09687 | 20.41442 | 20.61423 | 19.46665 | 3 | 2 | 2 | 11.8 | 7.9 | 7.9 | 39.393 | 13.705 | 10192000 | 3 | Phosphoribosyl pyrophosphate synthase-associated protein 1 | PRPSAP1 |
| 24.65785 | 21.06992 | 20.59423 | 21.3858 | 6 | 5 | 5 | 24 | 20.8 | 20.8 | 25.542 | 33.398 | 82164000 | 7 | Serine/arginine-rich splicing factor 9 | SRSF9 |
| 22.83285 | 17.04366 | 20.58637 | 19.40249 | 6 | 5 | 5 | 25.3 | 25.3 | 25.3 | 27.893 | 39.202 | 153680000 | 9 | Vesicle-associated membrane protein-associated protein A | VAPA |
| 21.45849 | 20.86037 | 20.57579 | 19.09804 | 4 | 4 | 4 | 28.5 | 28.5 | 28.5 | 23.583 | 46.268 | 65912000 | 6 | Tubulin-folding cofactor B | TBCB |
| 22.04307 | 22.08625 | 20.57052 | 18.26103 | 7 | 7 | 7 | 37.1 | 37.1 | 37.1 | 22.793 | 45.141 | 140030000 | 10 | NAD(P)H dehydrogenase [quinone] 1 | NQO1 |
| 17.95268 | 19.70308 | 20.55946 | 20.80144 | 3 | 3 | 3 | 5.3 | 5.3 | 5.3 | 63.779 | 17.007 | 59537000 | 4 | Serine/threonine-protein phosphatase 6 regulatory subunit 3 | PPP6R3 |
| 20.17974 | 21.20955 | 20.51855 | 19.6415 | 6 | 6 | 6 | 24.7 | 24.7 | 24.7 | 33.232 | 38.801 | 45891000 | 8 | Alpha-soluble NSF attachment protein | NAPA |
| 21.45337 | 20.80861 | 20.50882 | 21.17249 | 3 | 3 | 3 | 17.2 | 17.2 | 17.2 | 18.657 | 18.739 | 17184000 | 3 | 39S ribosomal protein L43, mitochondrial | MRPL43 |
| 21.60494 | 22.29863 | 20.50426 | 19.39458 | 8 | 8 | 8 | 36.4 | 36.4 | 36.4 | 28.763 | 62.046 | 98956000 | 13 | Ribosome maturation protein SBDS | SBDS |
| 24.06036 | 20.18641 | 20.50388 | 18.6983 | 5 | 5 | 5 | 25.9 | 25.9 | 25.9 | 26.736 | 42.997 | 44166000 | 6 | Cysteine and glycine-rich protein 2 | CSRP2 |
| 22.31487 | 20.76612 | 20.49541 | 21.83175 | 5 | 5 | 5 | 19.8 | 19.8 | 19.8 | 27.049 | 28.324 | 31987000 | 8 | Oxidoreductase HTATIP2 | HTATIP2 |
| 27.31691 | 19.80796 | 20.47298 | 19.41634 | 41 | 7 | 0 | 76.4 | 23.2 | 0 | 32.85 | 141.2 | 3160900000 | 12 | Tropomyosin beta chain | TPM2 |
| 21.74747 | 22.0141 | 20.46979 | 19.3519 | 4 | 4 | 3 | 8.2 | 8.2 | 6.9 | 78.452 | 48.21 | 126160000 | 4 | Serine/threonine-protein kinase MARK2 | MARK2 |
| 19.77399 | 21.06789 | 20.46957 | 20.05954 | 4 | 3 | 3 | 15.6 | 13.1 | 13.1 | 31.279 | 21.121 | 35014000 | 3 | U1 small nuclear ribonucleoprotein A | SNRPA |
| 20.27696 | 20.90426 | 20.44324 | 19.78221 | 3 | 3 | 3 | 7.5 | 7.5 | 7.5 | 59.075 | 18.978 | 37577000 | 5 | SAP domain-containing ribonucleoprotein | SARNP |
| 22.1739 | 21.83472 | 20.43064 | 20.99762 | 7 | 7 | 7 | 7.5 | 7.5 | 7.5 | 134.42 | 74.825 | 123770000 | 8 | Sperm-associated antigen 5 | SPAG5 |
| 18.4859 | 17.89937 | 20.41411 | 19.89353 | 3 | 2 | 2 | 10.9 | 8.1 | 8.1 | 36.171 | 11.762 | 28517000 | 2 | Isocitrate dehydrogenase [NADP], mitochondrial | IDH2 |
| 17.18085 | 21.33995 | 20.414 | 19.44857 | 4 | 4 | 4 | 7.9 | 7.9 | 7.9 | 78.346 | 23.226 | 91634000 | 5 | Translation initiation factor eIF-2B subunit epsilon | EIF2B5 |
| 20.24899 | 22.06618 | 20.40041 | 18.975 | 4 | 4 | 4 | 22.9 | 22.9 | 22.9 | 22.475 | 25.966 | 41476000 | 5 | GTP-binding protein 10 | GTPBP10 |
| 18.61013 | 20.17463 | 20.39927 | 21.62805 | 3 | 3 | 3 | 7.4 | 7.4 | 7.4 | 40.796 | 15.525 | 29933000 | 4 | Endophilin-B1 | SH3GLB1 |
| 23.42534 | 18.09898 | 20.3931 | 20.33745 | 3 | 3 | 3 | 10.1 | 10.1 | 10.1 | 34.295 | 20.418 | 32838000 | 4 | High mobility group protein HMG-I/HMG-Y | HMGA1 |
| 22.51444 | 16.57232 | 20.391 | 20.36239 | 4 | 4 | 4 | 28.2 | 28.2 | 28.2 | 18.868 | 23.544 | 21395000 | 4 | Rho-related GTP-binding protein RhoF | RHOF |
| 22.90942 | 17.84473 | 20.38269 | 20.63644 | 4 | 4 | 4 | 30.6 | 30.6 | 30.6 | 20.201 | 30.818 | 20024000 | 4 | Mid1-interacting protein 1 | MID1IP1 |
| 21.26865 | 20.19063 | 20.38258 | 19.89571 | 4 | 4 | 4 | 14.4 | 14.4 | 14.4 | 33.535 | 28.289 | 66214000 | 5 | 39S ribosomal protein L19, mitochondrial | MRPL19 |
| 17.03332 | 18.12794 | 20.37581 | 18.84826 | 3 | 3 | 3 | 53.2 | 53.2 | 53.2 | 8.6739 | 19.463 | 7885400 | 3 | GTP-binding protein Rheb | RHEB |
| 24.52779 | 21.18453 | 20.36282 | 20.22165 | 19 | 19 | 19 | 16.1 | 16.1 | 16.1 | 170.45 | 171.39 | 501070000 | 20 | Tyrosine-protein kinase BAZ1B | BAZ1B |
| 20.28895 | 20.30908 | 20.35917 | 19.40601 | 4 | 4 | 4 | 24.2 | 24.2 | 24.2 | 20.629 | 25.216 | 35064000 | 5 | ADP-sugar pyrophosphatase | NUDT5 |
| 25.13762 | 20.82842 | 20.35767 | 19.85126 | 17 | 17 | 17 | 29.6 | 29.6 | 29.6 | 85.652 | 225.03 | 869690000 | 20 | Targeting protein for Xklp2 | TPX2;HCA90 |
| 26.29579 | 19.84031 | 20.347 | 19.28744 | 35 | 35 | 35 | 27.1 | 27.1 | 27.1 | 200.84 | 323.31 | 2137800000 | 47 | Supervillin | SVIL |
| 27.69046 | 19.3706 | 20.33898 | 20.09288 | 35 | 9 | 9 | 71.5 | 16.9 | 16.9 | 32.722 | 293.84 | 4140200000 | 16 | Tropomyosin alpha-4 chain | TPM4 |
| 17.99005 | 20.96127 | 20.33876 | 19.21257 | 4 | 4 | 4 | 3.8 | 3.8 | 3.8 | 131.14 | 23.788 | 15543000 | 4 | Alpha-mannosidase 2 | MAN2A1 |
| 17.25147 | 24.54492 | 20.32768 | 19.4604 | 7 | 1 | 1 | 48.8 | 11.4 | 11.4 | 13.519 | 15.632 | 319200000 | 8 |  | PDIA3 |
| 19.66186 | 21.58646 | 20.29541 | 21.43538 | 3 | 3 | 3 | 7.2 | 7.2 | 7.2 | 52.878 | 17.828 | 34582000 | 4 | Cytosolic non-specific dipeptidase | CNDP2 |
| 21.57603 | 20.48827 | 20.28749 | 19.48328 | 5 | 5 | 5 | 24.6 | 24.6 | 24.6 | 30.065 | 39.006 | 77330000 | 6 | Ribosome biogenesis protein NSA2 homolog | NSA2 |
| 20.74197 | 22.96888 | 20.28093 | 19.05883 | 5 | 5 | 5 | 26.3 | 26.3 | 26.3 | 22.487 | 36.517 | 80450000 | 8 | Histone H1x | H1FX |
| 22.7254 | 23.93984 | 20.25914 | 22.91528 | 10 | 10 | 10 | 11.1 | 11.1 | 11.1 | 116.46 | 72.093 | 295980000 | 12 | La-related protein 1 | LARP1 |
| 26.24162 | 20.03443 | 20.25914 | 19.23972 | 14 | 14 | 13 | 38.9 | 38.9 | 36.1 | 48.294 | 183.85 | 1413300000 | 18 | Leucine-rich repeat flightless-interacting protein 2 | LRRFIP2 |
| 22.6327 | 18.15847 | 20.25003 | 19.48952 | 4 | 4 | 4 | 6.3 | 6.3 | 6.3 | 81.572 | 29.861 | 161850000 | 6 | Condensin complex subunit 2 | NCAPH |
| 21.29373 | 17.32596 | 20.24458 | 18.82608 | 3 | 2 | 2 | 14.6 | 9 | 9 | 30.561 | 15.571 | 34924000 | 4 | Serine/threonine-protein phosphatase;Serine/threonine-protein phosphatase 4 catalytic subunit | PPP4C |
| 18.53204 | 22.9607 | 20.23519 | 22.97192 | 4 | 4 | 4 | 40.7 | 40.7 | 40.7 | 13.577 | 25.846 | 57490000 | 6 | Selenium-binding protein 1 | SELENBP1 |
| 20.73909 | 22.41599 | 20.23363 | 20.73032 | 5 | 5 | 5 | 5.2 | 5.2 | 5.2 | 114.02 | 30.104 | 60642000 | 6 | Nuclear export mediator factor NEMF | NEMF |
| 21.80574 | 20.66284 | 20.22329 | 19.73609 | 6 | 6 | 6 | 33.6 | 33.6 | 33.6 | 32.251 | 54.835 | 94065000 | 7 | Thioredoxin-like protein 1 | TXNL1 |
| 21.66696 | 22.00881 | 20.20492 | 19.36966 | 8 | 8 | 8 | 7.9 | 7.9 | 7.9 | 153.1 | 53.3 | 81049000 | 10 | Phosphoinositide 3-kinase regulatory subunit 4 | PIK3R4 |
| 21.63201 | 21.14202 | 20.19208 | 19.25181 | 4 | 4 | 4 | 4.5 | 4.5 | 4.5 | 121.85 | 25.733 | 70956000 | 5 | Formin-like protein 1 | FMNL1 |
| 22.39778 | 21.73256 | 20.16829 | 22.02168 | 5 | 5 | 5 | 7.9 | 7.9 | 7.9 | 100.18 | 38.809 | 242960000 | 6 | RNA-binding protein 25 | RBM25 |
| 17.72813 | 27.98381 | 20.16189 | 19.57979 | 9 | 9 | 9 | 42.4 | 42.4 | 42.4 | 21.641 | 108.72 | 1197300000 | 26 | Syndecan-4;Syndecan | SDC4 |
| 20.65683 | 22.11721 | 20.11883 | 20.00089 | 4 | 4 | 4 | 24.6 | 24.6 | 24.6 | 30.039 | 33.139 | 53951000 | 6 | Exosome complex component RRP43 | EXOSC8;DKFZp564C0482 |
| 19.78016 | 21.82279 | 20.11337 | 19.43899 | 4 | 4 | 4 | 11.8 | 11.8 | 11.8 | 35.236 | 24.218 | 38155000 | 5 | Probable dimethyladenosine transferase | DIMT1 |
| 20.76239 | 17.56677 | 20.1107 | 19.61768 | 3 | 3 | 3 | 4.3 | 4.3 | 4.3 | 62.328 | 16.449 | 39279000 | 3 | Bifunctional coenzyme A synthase;Phosphopantetheine adenylyltransferase;Dephospho-CoA kinase | COASY |
| 21.6439 | 17.83157 | 20.1061 | 18.94065 | 3 | 3 | 3 | 24.7 | 24.7 | 24.7 | 18.925 | 18.359 | 9941600 | 3 | PEST proteolytic signal-containing nuclear protein | PCNP |
| 22.42019 | 18.20745 | 20.10018 | 17.74426 | 5 | 4 | 3 | 3.9 | 3.4 | 2.6 | 223.73 | 43.413 | 141180000 | 7 | Myosin-6;Myosin-7 | MYH6;MYH7 |
| 19.37886 | 21.27065 | 20.06631 | 20.7123 | 3 | 3 | 3 | 1.8 | 1.8 | 1.8 | 183.56 | 33.104 | 83098000 | 7 | Collagen alpha-1(V) chain | COL5A1 |
| 22.76452 | 19.74614 | 20.06105 | 19.8795 | 6 | 6 | 6 | 9.4 | 9.4 | 9.4 | 101.34 | 68.361 | 204460000 | 6 | Smoothelin | SMTN |
| 22.84232 | 18.65508 | 20.05272 | 19.23474 | 8 | 8 | 8 | 46.4 | 46.4 | 46.4 | 34.482 | 143.39 | 151090000 | 9 | Coatomer subunit epsilon | COPE |
| 22.77163 | 17.70352 | 20.04634 | 19.04883 | 8 | 8 | 6 | 50.6 | 50.6 | 41.8 | 26.927 | 105.2 | 141820000 | 9 | EF-hand domain-containing protein D1 | EFHD1 |
| 23.77836 | 17.53715 | 20.03899 | 20.01 | 10 | 10 | 5 | 14 | 14 | 7.3 | 120.63 | 168.07 | 289550000 | 10 | A-kinase anchor protein 2 | AKAP2 |
| 22.14323 | 17.70132 | 20.03658 | 19.51857 | 5 | 5 | 5 | 17.1 | 17.1 | 17.1 | 40.254 | 37.549 | 96879000 | 5 | AP-1 complex subunit mu-1 | AP1M1 |
| 22.85783 | 18.54963 | 20.03525 | 20.57801 | 6 | 6 | 6 | 19.1 | 19.1 | 19.1 | 43.159 | 72.093 | 187660000 | 9 | Very-long-chain (3R)-3-hydroxyacyl-CoA dehydratase 3 | PTPLAD1 |
| 26.71175 | 20.18677 | 20.02797 | 20.18665 | 52 | 47 | 16 | 39 | 32.4 | 16.8 | 555.61 | 323.31 | 5516800000 | 76 | Epiplakin | EPPK1 |
| 20.71188 | 22.18274 | 20.02689 | 20.21074 | 5 | 5 | 5 | 24.9 | 24.9 | 24.9 | 32.789 | 36.667 | 73507000 | 9 | Exosome complex component RRP4 | EXOSC2 |
| 23.66568 | 20.93596 | 20.0155 | 21.29216 | 10 | 10 | 10 | 20.2 | 20.2 | 20.2 | 68.254 | 65.225 | 264590000 | 11 | Serine/threonine-protein kinase PLK1 | PLK1;Plk-1 delta |
| 23.83547 | 16.68563 | 20.00138 | 18.41075 | 20 | 1 | 1 | 91.7 | 5.4 | 5.4 | 26.934 | 6.4415 | 291850000 | 1 |  | ACTG1 |
| 23.63766 | 21.44955 | 19.99824 | 20.18737 | 14 | 14 | 12 | 14.1 | 14.1 | 12.7 | 150.83 | 172.24 | 218530000 | 15 | Sister chromatid cohesion protein PDS5 homolog A | PDS5A |
| 21.30686 | 21.46523 | 19.97487 | 22.01431 | 3 | 3 | 3 | 12.2 | 12.2 | 12.2 | 21.614 | 17.139 | 21142000 | 4 | PRA1 family protein 3 | ARL6IP5 |
| 22.84311 | 21.97267 | 19.96323 | 22.15885 | 6 | 6 | 6 | 15.8 | 15.8 | 15.8 | 48.486 | 56.061 | 166800000 | 12 | Protein kinase C and casein kinase substrate in neurons protein 3 | PACSIN3 |
| 21.12149 | 20.60268 | 19.9624 | 19.60612 | 5 | 5 | 5 | 17.4 | 17.4 | 17.4 | 38.963 | 35.784 | 69377000 | 5 | Ubiquitin-associated domain-containing protein 2 | UBAC2 |
| 18.12137 | 19.61852 | 19.96113 | 21.70961 | 3 | 3 | 3 | 9.8 | 9.8 | 9.8 | 32.922 | 15.846 | 20138000 | 3 | Ubiquitin-conjugating enzyme E2 Z | UBE2Z |
| 18.93637 | 19.1556 | 19.94598 | 22.50045 | 3 | 3 | 3 | 3.1 | 3.1 | 3.1 | 111.13 | 17.199 | 41366000 | 5 | Integrin alpha-V;Integrin alpha-V heavy chain;Integrin alpha-V light chain | ITGAV |
| 19.55595 | 17.24359 | 19.91724 | 18.65625 | 3 | 3 | 3 | 2.6 | 2.6 | 2.6 | 166.8 | 16.913 | 13790000 | 3 | Lipopolysaccharide-responsive and beige-like anchor protein | LRBA |
| 24.14849 | 24.6542 | 19.8952 | 22.98975 | 16 | 16 | 5 | 14.3 | 14.3 | 3.7 | 146.67 | 124.81 | 520950000 | 25 | Clustered mitochondria protein homolog | CLUH |
| 21.46653 | 23.06336 | 19.88853 | 22.89852 | 9 | 9 | 9 | 11.3 | 11.3 | 11.3 | 129.15 | 58.307 | 100400000 | 13 | Plasma membrane calcium-transporting ATPase 1;Calcium-transporting ATPase;Plasma membrane calcium-transporting ATPase 4 | ATP2B1;ATP2B4 |
| 20.50949 | 18.07978 | 19.88573 | 19.2784 | 3 | 3 | 3 | 10.4 | 10.4 | 10.4 | 29.247 | 18.18 | 34089000 | 3 | Replication protein A 32 kDa subunit | RPA2 |
| 22.08758 | 16.91274 | 19.87283 | 18.98453 | 4 | 4 | 4 | 13.6 | 13.6 | 13.6 | 42.129 | 54.231 | 94779000 | 5 | Protein HGH1 homolog | HGH1 |
| 19.55446 | 18.15628 | 19.87237 | 19.91712 | 3 | 3 | 3 | 16.3 | 16.3 | 16.3 | 26.381 | 19.672 | 14942000 | 3 | Cytosolic Fe-S cluster assembly factor NUBP2 | NUBP2 |
| 22.13286 | 21.1657 | 19.8663 | 21.8589 | 11 | 3 | 3 | 10.6 | 3.7 | 3.7 | 117.26 | 19.92 | 182170000 | 3 | Probable global transcription activator SNF2L1 | SMARCA1 |
| 22.35353 | 20.65805 | 19.86197 | 21.96349 | 3 | 3 | 3 | 6.1 | 6.1 | 6.1 | 58.022 | 18.749 | 140640000 | 5 | Serine/threonine-protein kinase OSR1 | OXSR1 |
| 26.43164 | 18.40213 | 19.84562 | 19.7258 | 7 | 2 | 0 | 50 | 7.9 | 0 | 13.906 | 12.407 | 268160000 | 3 | Histone H2B type 1-O | HIST1H2BO |
| 21.64302 | 19.64516 | 19.83047 | 20.04074 | 7 | 5 | 5 | 35.8 | 27.1 | 27.1 | 26.697 | 67.595 | 71554000 | 6 | EF-hand domain-containing protein D2 | EFHD2 |
| 22.07975 | 19.16787 | 19.81989 | 19.97458 | 12 | 1 | 1 | 12.7 | 2.1 | 2.1 | 121.44 | 29.493 | 85499000 | 1 | POTE ankyrin domain family member F | POTEF |
| 21.66505 | 22.62205 | 19.81928 | 19.48853 | 5 | 5 | 5 | 12.4 | 12.4 | 12.4 | 50.24 | 31.766 | 104690000 | 7 | Translation initiation factor eIF-2B subunit gamma | EIF2B3 |
| 17.9307 | 19.45471 | 19.81885 | 19.98995 | 3 | 1 | 1 | 11.4 | 3.8 | 3.8 | 38.226 | 6.3714 | 12070000 | 1 | Serine/threonine-protein kinase 24;Serine/threonine-protein kinase 24 36 kDa subunit;Serine/threonine-protein kinase 24 12 kDa subunit | STK24 |
| 24.44727 | 21.67943 | 19.81737 | 19.03299 | 5 | 5 | 5 | 23.4 | 23.4 | 23.4 | 26.72 | 32.13 | 57317000 | 6 | Ribosomal RNA small subunit methyltransferase NEP1 | EMG1 |
| 24.37643 | 22.02908 | 19.81597 | 20.99348 | 9 | 9 | 9 | 8.5 | 8.5 | 8.5 | 132.65 | 81.784 | 562340000 | 12 | Double-stranded RNA-specific adenosine deaminase | ADAR |
| 20.59295 | 18.02629 | 19.80592 | 20.58972 | 4 | 4 | 4 | 23.8 | 23.8 | 23.8 | 28.783 | 39.789 | 31219000 | 4 | Multivesicular body subunit 12A | MVB12A |
| 21.62716 | 21.91849 | 19.79799 | 19.54643 | 4 | 4 | 4 | 4.6 | 4.6 | 4.6 | 138.99 | 26.811 | 75159000 | 3 | STE20-like serine/threonine-protein kinase | SLK |
| 20.95355 | 22.53379 | 19.78346 | 18.162 | 6 | 6 | 6 | 6.6 | 6.6 | 6.6 | 129.29 | 36.916 | 50824000 | 8 | Integrin alpha-2 | ITGA2 |
| 23.73593 | 18.19765 | 19.77321 | 18.32578 | 31 | 1 | 0 | 67.6 | 3.9 | 0 | 32.816 | 8.0121 | 269890000 | 2 | Tropomyosin alpha-1 chain | TPM1 |
| 23.57161 | 18.51168 | 19.76974 | 18.77859 | 5 | 5 | 5 | 6 | 6 | 6 | 128.79 | 34.823 | 823710000 | 3 | Nck-associated protein 1 | NCKAP1 |
| 20.59386 | 21.41498 | 19.75968 | 20.04236 | 3 | 3 | 3 | 5.9 | 5.9 | 5.9 | 57.583 | 17.818 | 82828000 | 3 | E3 UFM1-protein ligase 1 | UFL1 |
| 19.9912 | 19.79255 | 19.74916 | 20.61175 | 3 | 3 | 3 | 14.2 | 14.2 | 14.2 | 30.601 | 18.021 | 22332000 | 3 | Cytochrome c-type heme lyase | HCCS |
| 23.09402 | 23.56139 | 19.74504 | 24.12447 | 8 | 8 | 8 | 24.8 | 24.8 | 24.8 | 54.416 | 75.711 | 293500000 | 12 | Probable ATP-dependent RNA helicase DDX6 | DDX6 |
| 20.8226 | 21.64526 | 19.73964 | 19.61002 | 4 | 4 | 4 | 4.1 | 4.1 | 4.1 | 156.78 | 26.909 | 30630000 | 4 | CAP-Gly domain-containing linker protein 1 | CLIP1 |
| 17.99103 | 22.07959 | 19.73494 | 21.82342 | 6 | 6 | 6 | 13.6 | 13.6 | 13.6 | 60.67 | 44.943 | 106020000 | 8 | 60 kDa SS-A/Ro ribonucleoprotein | TROVE2 |
| 22.63176 | 23.02141 | 19.72488 | 22.6356 | 15 | 3 | 1 | 32.9 | 7.3 | 2.7 | 50.432 | 23.209 | 248970000 | 8 | Tubulin beta-3 chain | TUBB3 |
| 23.57923 | 18.10195 | 19.72374 | 19.78518 | 3 | 3 | 3 | 32.7 | 32.7 | 32.7 | 12.417 | 17.747 | 36286000 | 5 | AP-2 complex subunit sigma | AP2S1 |
| 24.02217 | 17.16275 | 19.72268 | 19.19188 | 39 | 2 | 0 | 81.7 | 10.2 | 0 | 32.865 | 80.032 | 354760000 | 4 | Tropomyosin alpha-1 chain | TPM1 |
| 22.26184 | 21.77121 | 19.72067 | 19.72172 | 6 | 6 | 6 | 8.4 | 8.4 | 8.4 | 87.133 | 37.182 | 189390000 | 5 | Ubiquitin carboxyl-terminal hydrolase 10 | USP10 |
| 21.20634 | 22.11356 | 19.71618 | 21.68398 | 3 | 3 | 3 | 4.4 | 4.4 | 4.4 | 83.231 | 18.756 | 108930000 | 7 | Bcl-2-associated transcription factor 1 | BCLAF1 |
| 22.10322 | 21.92937 | 19.70806 | 21.39121 | 3 | 3 | 3 | 15.9 | 15.9 | 15.9 | 26.383 | 18.963 | 26453000 | 9 | Exosome complex component RRP41 | EXOSC4 |
| 19.92408 | 21.80294 | 19.69578 | 21.20233 | 3 | 3 | 3 | 14.3 | 14.3 | 14.3 | 29.395 | 18.286 | 47691000 | 3 | 28S ribosomal protein S18b, mitochondrial | MRPS18B |
| 20.71305 | 21.84759 | 19.68724 | 19.81005 | 6 | 5 | 5 | 5.3 | 4.6 | 4.6 | 153.5 | 27.788 | 46958000 | 5 | DNA-directed RNA polymerase III subunit RPC1 | POLR3A |
| 23.85072 | 19.27991 | 19.6824 | 20.02405 | 15 | 15 | 15 | 14.7 | 14.7 | 14.7 | 139.88 | 143.66 | 260510000 | 15 | Chromosome-associated kinesin KIF4A;Chromosome-associated kinesin KIF4B | KIF4A;KIF4B |
| 23.26141 | 18.87523 | 19.66291 | 20.31226 | 6 | 6 | 6 | 30.4 | 30.4 | 30.4 | 29.841 | 71.851 | 194290000 | 10 | DnaJ homolog subfamily C member 8 | DNAJC8 |
| 19.91733 | 20.512 | 19.65246 | 20.86884 | 4 | 3 | 3 | 3.6 | 2.8 | 2.8 | 155.23 | 20.365 | 29191000 | 6 | ATP-dependent RNA helicase DHX29 | DHX29 |
| 22.25517 | 18.87367 | 19.6482 | 18.67078 | 6 | 6 | 6 | 6.8 | 6.8 | 6.8 | 125.47 | 49.453 | 93899000 | 6 | Pleckstrin homology-like domain family B member 2 | PHLDB2 |
| 22.15842 | 20.11159 | 19.64743 | 19.94894 | 6 | 6 | 6 | 27.2 | 27.2 | 27.2 | 35.079 | 56.143 | 95543000 | 7 | WD repeat-containing protein 82 | WDR82 |
| 18.055 | 18.1373 | 19.64447 | 21.86268 | 3 | 3 | 3 | 10.4 | 10.4 | 10.4 | 52.827 | 19.887 | 31400000 | 4 | Tyrosine-protein phosphatase non-receptor type 11 | PTPN11 |
| 22.79713 | 23.02064 | 19.64004 | 23.32965 | 4 | 4 | 4 | 17.1 | 17.1 | 17.1 | 39.786 | 38.383 | 228370000 | 12 | Histone acetyltransferase type B catalytic subunit | HAT1 |
| 25.59323 | 17.53903 | 19.62715 | 17.73414 | 15 | 15 | 15 | 26.2 | 26.2 | 26.2 | 73.822 | 144.09 | 914420000 | 16 | Actin-binding LIM protein 1 | ABLIM1 |
| 21.50993 | 21.08486 | 19.61389 | 20.61378 | 4 | 4 | 4 | 4.3 | 4.3 | 4.3 | 138.67 | 29.701 | 59591000 | 4 | TBC1 domain family member 9B | TBC1D9B |
| 21.08102 | 17.8966 | 19.61243 | 20.03461 | 23 | 2 | 2 | 90.2 | 5.3 | 5.3 | 26.63 | 11.94 | 56219000 | 2 |  |  |
| 20.15239 | 20.0156 | 19.61205 | 19.71154 | 4 | 4 | 4 | 21.4 | 21.4 | 21.4 | 30.608 | 26.308 | 28255000 | 4 | Omega-amidase NIT2 | NIT2 |
| 18.15285 | 20.37358 | 19.60937 | 20.43923 | 3 | 3 | 3 | 12.6 | 12.6 | 12.6 | 35.111 | 21.358 | 22312000 | 5 | Dehydrogenase/reductase SDR family member 7B | DHRS7B |
| 18.04052 | 20.51355 | 19.60554 | 19.80723 | 4 | 4 | 4 | 1.8 | 1.8 | 1.8 | 251.46 | 23.932 | 19322000 | 5 | Activating signal cointegrator 1 complex subunit 3 | ASCC3 |
| 18.83516 | 22.1054 | 19.60437 | 21.18023 | 3 | 3 | 3 | 9.9 | 9.9 | 9.9 | 54.271 | 19.476 | 47370000 | 3 | Zinc finger protein 622 | ZNF622 |
| 20.11858 | 17.37983 | 19.60058 | 19.42866 | 3 | 3 | 3 | 2.4 | 2.4 | 2.4 | 194.31 | 16.823 | 24725000 | 3 | Serine/threonine-protein kinase MRCK beta | CDC42BPB |
| 21.78173 | 18.13974 | 19.59377 | 19.07934 | 3 | 3 | 3 | 5.6 | 5.6 | 5.6 | 81.123 | 20.049 | 148280000 | 3 | Nucleolar protein 11 | NOL11 |
| 17.05732 | 18.58795 | 19.58773 | 22.20851 | 5 | 5 | 5 | 4.4 | 4.4 | 4.4 | 132.6 | 28.142 | 62541000 | 4 | Tubulin-specific chaperone D | TBCD |
| 16.86085 | 20.43247 | 19.58444 | 17.61731 | 3 | 3 | 3 | 38.4 | 38.4 | 38.4 | 11.255 | 19.366 | 11221000 | 4 | Protein DDI1 homolog 2 | DDI2 |
| 21.68906 | 21.84142 | 19.58385 | 21.81053 | 6 | 6 | 6 | 12.6 | 12.6 | 12.6 | 72.379 | 44.682 | 83660000 | 8 | Sec1 family domain-containing protein 1 | SCFD1 |
| 21.52535 | 17.38352 | 19.5753 | 19.01702 | 3 | 2 | 2 | 4.4 | 3.4 | 3.4 | 102.47 | 11.124 | 95125000 | 2 | Neurofilament medium polypeptide | NEFM |
| 24.16599 | 23.3534 | 19.56116 | 23.60682 | 4 | 4 | 4 | 29.2 | 29.2 | 29.2 | 14.958 | 23.215 | 78147000 | 5 | Prostaglandin E synthase 3 | PTGES3 |
| 21.25742 | 17.71459 | 19.5585 | 19.68124 | 3 | 3 | 3 | 13.8 | 13.8 | 13.8 | 41.884 | 21.665 | 73697000 | 5 | Histone-arginine methyltransferase CARM1 | CARM1 |
| 18.4692 | 19.07966 | 19.55526 | 19.19157 | 3 | 2 | 2 | 17.5 | 13.2 | 13.2 | 21.846 | 12.795 | 14504000 | 3 | Serpin B8 | SERPINB8 |
| 18.0951 | 21.04387 | 19.54919 | 19.89542 | 4 | 4 | 4 | 2.7 | 2.7 | 2.7 | 195.7 | 23.914 | 33163000 | 4 | Thyroid receptor-interacting protein 11 | TRIP11 |
| 26.9069 | 19.22651 | 19.54877 | 19.83963 | 20 | 20 | 20 | 38 | 38 | 38 | 75.356 | 323.31 | 2917000000 | 28 | Mitotic interactor and substrate of PLK1 | MISP |
| 21.65683 | 16.89641 | 19.54475 | 20.0713 | 3 | 3 | 3 | 8.2 | 8.2 | 8.2 | 56.298 | 22.361 | 63463000 | 3 | Calcium/calmodulin-dependent protein kinase type II subunit delta | CAMK2D |
| 18.77478 | 20.45081 | 19.5392 | 20.30765 | 5 | 5 | 5 | 11.8 | 11.8 | 11.8 | 66.034 | 29.214 | 45763000 | 3 | Elongation factor G, mitochondrial | GFM1 |
| 21.41101 | 22.1046 | 19.53564 | 22.83825 | 3 | 3 | 3 | 8.1 | 8.1 | 8.1 | 51.201 | 19.366 | 104750000 | 7 | Acetyltransferase component of pyruvate dehydrogenase complex;Dihydrolipoyllysine-residue acetyltransferase component of pyruvate dehydrogenase complex, mitochondrial | DLAT |
| 21.4761 | 24.68587 | 19.53125 | 19.81719 | 14 | 14 | 14 | 16.5 | 16.5 | 16.5 | 89.834 | 97.406 | 438950000 | 16 | Probable ATP-dependent RNA helicase DDX27 | DDX27 |
| 18.66465 | 20.06553 | 19.51921 | 19.25273 | 4 | 1 | 1 | 17.4 | 3.3 | 3.3 | 42.707 | 6.3248 | 12950000 | 1 | Gamma-enolase;Enolase | ENO2;ENO3 |
| 20.50911 | 21.2021 | 19.51871 | 19.73867 | 3 | 3 | 3 | 12.1 | 12.1 | 12.1 | 31.444 | 21.036 | 40894000 | 4 | TIP41-like protein | TIPRL |
| 22.57004 | 20.81449 | 19.49468 | 22.63199 | 3 | 3 | 3 | 17.6 | 17.6 | 17.6 | 21.007 | 16.955 | 30926000 | 5 | Gamma-glutamylcyclotransferase | GGCT |
| 16.87402 | 22.39835 | 19.48953 | 19.43991 | 3 | 3 | 3 | 12.2 | 12.2 | 12.2 | 39.825 | 26.885 | 36761000 | 3 | ATP-dependent RNA helicase DDX55 | DDX55 |
| 25.22744 | 18.75981 | 19.48805 | 19.58952 | 12 | 12 | 12 | 23.9 | 23.9 | 23.9 | 56.974 | 90.649 | 693650000 | 14 | LEM domain-containing protein 2 | LEMD2 |
| 21.0416 | 17.62704 | 19.48768 | 19.9228 | 4 | 3 | 3 | 31.8 | 27.1 | 27.1 | 18.685 | 25.968 | 47758000 | 4 | Cyclin-dependent kinase 4 | CDK4 |
| 23.13297 | 21.40785 | 19.47341 | 20.9805 | 7 | 7 | 7 | 11.3 | 11.3 | 11.3 | 81.312 | 65.954 | 271050000 | 8 | Kinesin-like protein KIF2C | KIF2C |
| 21.77612 | 17.85882 | 19.46827 | 18.47597 | 5 | 3 | 3 | 8.3 | 5.9 | 5.9 | 74.222 | 21.399 | 98938000 | 3 | Fragile X mental retardation syndrome-related protein 2 | FXR2 |
| 21.6999 | 23.59457 | 19.46586 | 19.43939 | 3 | 3 | 3 | 10.7 | 10.7 | 10.7 | 50.714 | 44.506 | 105160000 | 4 | Ribosomal RNA-processing protein 8 | RRP8 |
| 23.76284 | 21.91492 | 19.46422 | 21.66036 | 7 | 7 | 7 | 11.7 | 11.7 | 11.7 | 71.026 | 66.154 | 402670000 | 11 | Rac GTPase-activating protein 1 | RACGAP1 |
| 22.90987 | 18.98792 | 19.46313 | 18.48627 | 29 | 2 | 2 | 82.7 | 8.2 | 8.2 | 39.001 | 29.957 | 195110000 | 2 |  | ACTN4 |
| 20.896 | 17.08266 | 19.4614 | 19.3437 | 3 | 3 | 1 | 9.5 | 9.5 | 4.2 | 42.123 | 18.102 | 42462000 | 3 | Guanine nucleotide-binding protein subunit alpha-11;Guanine nucleotide-binding protein subunit alpha-14 | GNA11;GNA14 |
| 21.60164 | 22.19584 | 19.45356 | 19.74027 | 11 | 6 | 6 | 11.7 | 7.1 | 7.1 | 122.87 | 66.015 | 96023000 | 7 | SWI/SNF complex subunit SMARCC1 | SMARCC1 |
| 21.01073 | 21.64588 | 19.44544 | 22.90174 | 7 | 7 | 7 | 16 | 16 | 16 | 59.095 | 45.112 | 95138000 | 10 | Merlin | NF2 |
| 21.97638 | 19.05916 | 19.44465 | 18.54464 | 6 | 6 | 6 | 25.6 | 25.6 | 25.6 | 35.144 | 46.135 | 86246000 | 6 | Serine/threonine-protein phosphatase 6 catalytic subunit;Serine/threonine-protein phosphatase 6 catalytic subunit, N-terminally processed | PPP6C |
| 19.73608 | 19.9631 | 19.44329 | 20.53623 | 3 | 3 | 3 | 2.5 | 2.5 | 2.5 | 130.08 | 17.98 | 20205000 | 3 | 5-3 exoribonuclease 1 | XRN1 |
| 23.32882 | 20.6591 | 19.44114 | 19.7459 | 11 | 11 | 11 | 7.9 | 7.9 | 7.9 | 189.67 | 85.727 | 348880000 | 14 | Tight junction protein ZO-1 | TJP1 |
| 21.11394 | 18.77088 | 19.43904 | 20.09107 | 3 | 2 | 2 | 19.9 | 14.1 | 14.1 | 21.308 | 12.344 | 8447600 | 2 | Rho-related GTP-binding protein RhoG | RHOG |
| 20.35111 | 18.87948 | 19.43442 | 19.19507 | 3 | 3 | 3 | 2.8 | 2.8 | 2.8 | 152.57 | 18.696 | 40910000 | 2 | Nuclear pore complex protein Nup214 | NUP214 |
| 22.68524 | 22.86503 | 19.43369 | 23.20371 | 4 | 4 | 4 | 11.3 | 11.3 | 11.3 | 60.541 | 52.388 | 197670000 | 12 | Atlastin-3 | ATL3 |
| 21.15882 | 21.95869 | 19.43218 | 22.28156 | 4 | 4 | 4 | 8.7 | 8.7 | 8.7 | 57.028 | 23.996 | 81371000 | 6 | UDP-N-acetylhexosamine pyrophosphorylase;UDP-N-acetylgalactosamine pyrophosphorylase;UDP-N-acetylglucosamine pyrophosphorylase | UAP1 |
| 18.27633 | 21.75156 | 19.42694 | 20.57542 | 3 | 3 | 3 | 8.7 | 8.7 | 8.7 | 49.192 | 16.734 | 25089000 | 2 | Testis-specific Y-encoded-like protein 1 | TSPYL1 |
| 20.41007 | 19.03627 | 19.42243 | 18.76718 | 3 | 3 | 3 | 2.8 | 2.8 | 2.8 | 152.53 | 29.89 | 30221000 | 3 | PERQ amino acid-rich with GYF domain-containing protein 2 | GIGYF2 |
| 22.06582 | 20.01686 | 19.406 | 19.77742 | 4 | 3 | 3 | 13.2 | 10.3 | 10.3 | 44.179 | 31.042 | 100420000 | 3 | Guanine nucleotide-binding protein G(s) subunit alpha isoforms short;Guanine nucleotide-binding protein G(s) subunit alpha isoforms XLas | GNAS |
| 23.033 | 22.89828 | 19.39985 | 23.87408 | 12 | 12 | 12 | 13 | 13 | 13 | 128.68 | 99.274 | 286260000 | 18 | Epidermal growth factor receptor | EGFR |
| 22.94376 | 15.72267 | 19.39418 | 19.21498 | 14 | 3 | 0 | 14.4 | 5 | 0 | 121.36 | 22.957 | 174210000 | 5 | POTE ankyrin domain family member E;POTE ankyrin domain family member I;POTE ankyrin domain family member J | POTEE;POTEI;POTEJ |
| 25.96603 | 19.32342 | 19.38932 | 19.91599 | 23 | 23 | 23 | 28 | 28 | 28 | 120.88 | 230.7 | 1416700000 | 32 | SLIT-ROBO Rho GTPase-activating protein 2 | SRGAP2 |
| 18.10964 | 24.56186 | 19.3784 | 18.74474 | 3 | 3 | 3 | 1.8 | 1.8 | 1.8 | 138.94 | 20.01 | 43552000 | 6 | Collagen alpha-1(I) chain | COL1A1 |
| 21.07051 | 17.49908 | 19.37372 | 18.19357 | 76 | 1 | 1 | 46.1 | 1 | 1 | 196.44 | 36.696 | 41796000 | 1 | Unconventional myosin-XVIIIa | MYO18A |
| 20.01672 | 20.02324 | 19.36989 | 20.40894 | 3 | 2 | 2 | 8.6 | 5.1 | 5.1 | 61.963 | 11.467 | 38072000 | 2 | Dihydropyrimidinase-related protein 3 | DPYSL3 |
| 19.42407 | 21.30558 | 19.36671 | 20.82997 | 3 | 3 | 3 | 5.3 | 5.3 | 5.3 | 54.854 | 20.796 | 54542000 | 4 | Protein LTV1 homolog | LTV1 |
| 18.45313 | 23.16302 | 19.35503 | 18.45671 | 5 | 5 | 5 | 17.5 | 17.5 | 17.5 | 41.45 | 30.99 | 45069000 | 5 | Surfeit locus protein 6 | SURF6 |
| 21.41905 | 21.75967 | 19.35268 | 18.07177 | 3 | 3 | 3 | 5 | 5 | 5 | 81.242 | 19.446 | 116780000 | 4 | Ribosomal biogenesis protein LAS1L | LAS1L |
| 23.01574 | 22.82846 | 19.35261 | 22.92203 | 5 | 3 | 3 | 14.8 | 9.6 | 9.6 | 51.998 | 23.555 | 243140000 | 5 | Histone deacetylase 2 | HDAC2 |
| 21.61509 | 17.63836 | 19.35163 | 18.61759 | 3 | 2 | 2 | 4.3 | 2.9 | 2.9 | 65.821 | 11.378 | 66051000 | 1 | Fragile X mental retardation protein 1 | FMR1 |
| 22.67349 | 17.13866 | 19.34502 | 19.18376 | 3 | 3 | 3 | 13.2 | 13.2 | 13.2 | 23.743 | 19.129 | 322430000 | 3 | THO complex subunit 7 homolog | THOC7 |
| 18.06176 | 21.98919 | 19.33887 | 22.52499 | 3 | 3 | 3 | 15.4 | 15.4 | 15.4 | 28.627 | 19.843 | 46408000 | 5 | Sodium/potassium-transporting ATPase subunit beta-1 | ATP1B1 |
| 19.14292 | 19.63387 | 19.32247 | 18.71209 | 3 | 1 | 1 | 2 | 0.7 | 0.7 | 223.07 | 8.7631 | 75588000 | 4 | Myosin-4 | MYH4 |
| 22.02861 | 23.08484 | 19.32112 | 19.86461 | 8 | 8 | 8 | 7.3 | 7.3 | 7.3 | 160.88 | 57.937 | 80677000 | 8 | Cleavage and polyadenylation specificity factor subunit 1 | CPSF1 |
| 24.4443 | 19.95035 | 19.30724 | 19.67237 | 19 | 19 | 19 | 21.3 | 21.3 | 21.3 | 117.7 | 162.98 | 460510000 | 23 | Centrosomal protein of 131 kDa | CEP131 |
| 18.51197 | 20.97575 | 19.30462 | 21.42645 | 4 | 4 | 4 | 4.6 | 4.6 | 4.6 | 119.75 | 24.698 | 94910000 | 4 | Protein transport protein Sec24A | SEC24A |
| 21.48586 | 17.61753 | 19.28407 | 19.74716 | 178 | 1 | 1 | 73.7 | 0.7 | 0.7 | 282.28 | 28.441 | 157530000 | 2 | Spectrin alpha chain, non-erythrocytic 1 | SPTAN1 |
| 18.0197 | 19.11894 | 19.28376 | 21.88554 | 3 | 3 | 3 | 5.3 | 5.3 | 5.3 | 80.702 | 18.386 | 76677000 | 3 | Mannosyl-oligosaccharide glucosidase | MOGS |
| 20.76174 | 17.03341 | 19.27453 | 19.75524 | 72 | 2 | 2 | 65.3 | 2.7 | 2.7 | 119.52 | 21.742 | 31454000 | 2 |  | LMO7 |
| 21.39686 | 20.12868 | 19.27209 | 20.41514 | 5 | 5 | 5 | 1.4 | 1.4 | 1.4 | 509.7 | 31.721 | 118750000 | 6 | E3 ubiquitin-protein ligase MYCBP2 | MYCBP2 |
| 23.77937 | 22.59384 | 19.27094 | 19.474 | 5 | 5 | 5 | 8.4 | 8.4 | 8.4 | 95.096 | 72.177 | 242370000 | 6 | Pre-mRNA-processing factor 40 homolog A | PRPF40A |
| 19.60096 | 17.17716 | 19.26879 | 18.90795 | 3 | 1 | 1 | 7 | 3.3 | 3.3 | 70.253 | 11.34 | 13989000 | 1 | Plastin-1 | PLS1 |
| 21.59149 | 18.22519 | 19.2666 | 19.37576 | 4 | 4 | 4 | 9.5 | 9.5 | 9.5 | 52.545 | 25.761 | 67805000 | 3 | NADH dehydrogenase [ubiquinone] iron-sulfur protein 2, mitochondrial | NDUFS2 |
| 17.66312 | 20.26899 | 19.24991 | 19.41796 | 5 | 3 | 3 | 5.1 | 3.2 | 3.2 | 102.31 | 17.919 | 30428000 | 2 | Hexokinase-1 | HK1 |
| 20.02188 | 19.65734 | 19.24785 | 19.28197 | 3 | 3 | 3 | 15.6 | 15.6 | 15.6 | 24.95 | 39.566 | 29490000 | 3 | Charged multivesicular body protein 4b | CHMP4B |
| 21.67878 | 22.18402 | 19.24194 | 21.70202 | 4 | 4 | 4 | 12.1 | 12.1 | 12.1 | 50.496 | 38.024 | 87618000 | 6 | Methionine aminopeptidase 2 | METAP2 |
| 21.42895 | 21.84671 | 19.23187 | 18.38855 | 3 | 3 | 3 | 9.8 | 9.8 | 9.8 | 35.936 | 17.914 | 63924000 | 2 | Parafibromin | CDC73 |
| 21.61823 | 20.11299 | 19.2252 | 19.46895 | 4 | 4 | 4 | 17.5 | 17.5 | 17.5 | 23.687 | 56.622 | 74422000 | 4 | ADP-ribosylation factor-like protein 6-interacting protein 4 | ARL6IP4 |
| 20.58793 | 19.3182 | 19.21772 | 18.96844 | 3 | 3 | 3 | 3.3 | 3.3 | 3.3 | 121.11 | 20.133 | 52469000 | 4 | Manganese-transporting ATPase 13A1 | ATP13A1 |
| 22.91807 | 22.91242 | 19.20793 | 22.28954 | 5 | 5 | 5 | 11.6 | 11.6 | 11.6 | 54.766 | 48.053 | 244780000 | 8 | U4/U6 small nuclear ribonucleoprotein Prp31 | PRPF31 |
| 20.46603 | 19.84882 | 19.19908 | 19.58489 | 3 | 3 | 3 | 5.3 | 5.3 | 5.3 | 77.673 | 18.945 | 60780000 | 4 | Methionine synthase reductase | MTRR |
| 16.96215 | 21.51898 | 19.19383 | 21.21792 | 5 | 2 | 2 | 20.9 | 7.4 | 7.4 | 44.059 | 13.611 | 26376000 | 3 | Beta-enolase;Enolase | ENO3 |
| 17.68748 | 21.45433 | 19.19285 | 20.45916 | 3 | 3 | 3 | 8 | 8 | 8 | 50.755 | 21.035 | 65997000 | 2 | Notchless protein homolog 1 | NLE1 |
| 22.23286 | 22.17405 | 19.18889 | 19.05464 | 7 | 7 | 7 | 8.5 | 8.5 | 8.5 | 137.75 | 46.333 | 102940000 | 8 | Helicase SKI2W | SKIV2L |
| 17.32945 | 21.19063 | 19.16465 | 20.49707 | 3 | 3 | 3 | 3.4 | 3.4 | 3.4 | 114 | 18.597 | 22129000 | 4 | Melanoma inhibitory activity protein 3 | MIA3 |
| 20.53108 | 18.29844 | 19.16443 | 19.03147 | 3 | 3 | 3 | 7 | 7 | 7 | 46.801 | 17.868 | 32764000 | 2 | DNA polymerase delta subunit 3 | POLD3 |
| 19.89266 | 18.72761 | 19.16122 | 19.09275 | 3 | 3 | 3 | 16.4 | 16.4 | 16.4 | 24.793 | 19.282 | 19756000 | 3 | NADH-cytochrome b5 reductase 1 | CYB5R1 |
| 21.28636 | 22.2986 | 19.13601 | 19.35126 | 7 | 7 | 7 | 5.4 | 5.4 | 5.4 | 174.76 | 42.805 | 103370000 | 8 | Glycogen debranching enzyme;4-alpha-glucanotransferase;Amylo-alpha-1,6-glucosidase | AGL |
| 23.54934 | 22.93243 | 19.121 | 22.66761 | 15 | 14 | 14 | 12.4 | 11.9 | 11.9 | 171.29 | 101.52 | 253110000 | 20 | Intron-binding protein aquarius | AQR |
| 24.62817 | 20.64878 | 19.12084 | 20.43125 | 15 | 7 | 7 | 48.5 | 22 | 22 | 50.67 | 103.22 | 567420000 | 14 | Lamina-associated polypeptide 2, isoforms beta/gamma;Thymopoietin;Thymopentin | TMPO |
| 22.85253 | 22.6396 | 19.11819 | 22.56125 | 6 | 6 | 6 | 13.8 | 13.8 | 13.8 | 58.32 | 38.808 | 194000000 | 8 | U4/U6 small nuclear ribonucleoprotein Prp4 | PRPF4 |
| 24.21297 | 23.41455 | 19.11628 | 24.01478 | 9 | 3 | 3 | 55.2 | 20.9 | 20.9 | 22.171 | 21.168 | 79993000 | 10 | Ras-related protein Rab-1B | RAB1B |
| 22.81866 | 22.62836 | 19.11583 | 20.41991 | 11 | 11 | 11 | 10.3 | 10.3 | 10.3 | 149.32 | 75.289 | 204980000 | 14 | Fanconi anemia group I protein | FANCI |
| 23.37531 | 26.13299 | 19.1141 | 19.0155 | 3 | 3 | 3 | 18.1 | 18.1 | 18.1 | 17.254 | 23.926 | 124910000 | 12 | Fibroblast growth factor 2 | FGF2 |
| 21.89549 | 18.02118 | 19.10748 | 20.18184 | 4 | 3 | 3 | 8.3 | 6.6 | 6.6 | 75.726 | 27.638 | 123270000 | 4 | Spermatogenesis-associated protein 5 | SPATA5 |
| 22.27185 | 19.24548 | 19.10383 | 19.35358 | 5 | 3 | 3 | 23.1 | 11.5 | 11.5 | 22.764 | 16.946 | 14082000 | 3 | Myosin light chain 6B | MYL6B |
| 20.85681 | 17.18395 | 19.10017 | 20.03255 | 7 | 1 | 1 | 50 | 8.7 | 8.7 | 13.936 | 7.182 | 5218900 | 1 | Histone H2B type 1-D | HIST1H2BD |
| 22.13461 | 23.41635 | 19.09549 | 19.89041 | 6 | 6 | 6 | 10 | 10 | 10 | 75.406 | 44.403 | 236040000 | 7 | ATP-dependent RNA helicase DDX18 | DDX18 |
| 18.56158 | 23.13688 | 19.08248 | 24.15661 | 4 | 4 | 4 | 8.3 | 8.3 | 8.3 | 47.91 | 26.376 | 152270000 | 7 | Adenylosuccinate synthetase isozyme 2 | ADSS |
| 17.65285 | 18.4492 | 19.08126 | 20.0893 | 5 | 2 | 2 | 4.2 | 2.3 | 2.3 | 179.48 | 21.788 | 8888700 | 2 | Ras GTPase-activating-like protein IQGAP3 | IQGAP3 |
| 17.85219 | 21.15623 | 19.07929 | 19.3757 | 3 | 3 | 3 | 6.8 | 6.8 | 6.8 | 50.435 | 22.959 | 20773000 | 4 | Rho GTPase-activating protein 1 | ARHGAP1 |
| 17.58579 | 22.03808 | 19.07619 | 21.95886 | 5 | 5 | 5 | 12.8 | 12.8 | 12.8 | 59.573 | 45.127 | 51637000 | 6 | Aladin | AAAS |
| 23.9974 | 25.88635 | 19.07239 | 18.08097 | 5 | 2 | 2 | 31 | 7.8 | 7.8 | 14.822 | 12.372 | 132930000 | 4 | 60S ribosomal protein L36a | RPL36A-HNRNPH2;RPL36A |
| 19.93977 | 17.90365 | 19.06933 | 18.87769 | 3 | 3 | 3 | 4.6 | 4.6 | 4.6 | 81.092 | 17.708 | 35654000 | 2 | Cullin-3 | CUL3 |
| 22.10764 | 23.16091 | 19.05617 | 23.27952 | 13 | 5 | 5 | 35.2 | 18.3 | 18.3 | 50.745 | 90.087 | 167940000 | 9 | Spliceosome RNA helicase DDX39B | DDX39B;hCG_2005638 |
| 20.95312 | 18.87526 | 19.05541 | 19.1773 | 5 | 2 | 2 | 18.1 | 7.6 | 7.6 | 27.168 | 10.88 | 11091000 | 2 |  | RPS15A |
| 22.42972 | 19.33031 | 19.05193 | 19.02067 | 6 | 1 | 1 | 18.7 | 2.9 | 2.9 | 42.016 | 8.6339 | 108970000 | 1 | Putative beta-actin-like protein 3 | POTEKP |
| 21.75499 | 16.79531 | 19.04883 | 21.28279 | 3 | 3 | 3 | 4.2 | 4.2 | 4.2 | 110.66 | 19.648 | 99990000 | 2 | Huntingtin-interacting protein 1 | HIP1 |
| 18.69809 | 21.20413 | 19.04407 | 22.26247 | 3 | 3 | 3 | 8.8 | 8.8 | 8.8 | 41.59 | 21.775 | 52419000 | 5 | Nucleolysin TIAR;Nucleolysin TIA-1 isoform p40 | TIAL1;TIA1 |
| 22.8941 | 18.23622 | 19.03925 | 18.94897 | 3 | 3 | 3 | 17 | 17 | 17 | 16.616 | 30.648 | 24321000 | 4 | Adenine phosphoribosyltransferase | APRT |
| 22.0621 | 17.34571 | 19.02893 | 19.6179 | 3 | 3 | 3 | 6 | 6 | 6 | 55.795 | 19.423 | 82283000 | 4 | Melanoma-associated antigen D2 | MAGED2 |
| 19.03109 | 19.20371 | 19.01578 | 19.95087 | 4 | 2 | 2 | 9.6 | 5 | 5 | 39.609 | 11.856 | 28255000 | 3 | UV excision repair protein RAD23 homolog A | RAD23A |
| 25.1671 | 19.1078 | 19.01432 | 19.37843 | 3 | 1 | 1 | 21.3 | 8.2 | 8.2 | 13.163 | 7.0349 | 103450000 | 2 | Histone H2A;Histone H2A.V;Histone H2A.Z | H2AFV;H2AFZ |
| 20.07601 | 17.31318 | 19.00892 | 18.51614 | 259 | 1 | 1 | 55.4 | 0.3 | 0.3 | 518.03 | 8.1152 | 78361000 | 1 | Plectin | PLEC |
| 21.32141 | 19.49397 | 19.00484 | 18.07096 | 5 | 5 | 5 | 14.6 | 14.6 | 14.6 | 39.611 | 34.101 | 56900000 | 5 | 26S proteasome non-ATPase regulatory subunit 8 | PSMD8 |
| 20.68414 | 19.48908 | 19.00255 | 19.01902 | 39 | 3 | 3 | 24.8 | 2.1 | 2.1 | 264.62 | 36.193 | 98196000 | 3 |  |  |
| 20.36784 | 20.90419 | 18.99708 | 18.29943 | 3 | 3 | 3 | 13.7 | 13.7 | 13.7 | 33.631 | 32.302 | 37730000 | 3 | Cell differentiation protein RCD1 homolog | RQCD1 |
| 18.89942 | 20.40239 | 18.99353 | 19.2146 | 3 | 3 | 3 | 2 | 2 | 2 | 254.41 | 37.805 | 33232000 | 3 | DnaJ homolog subfamily C member 13 | DNAJC13 |
| 19.38699 | 18.52645 | 18.99039 | 18.60208 | 21 | 1 | 1 | 93.6 | 8.5 | 8.5 | 29.859 | 18.363 | 43729000 | 1 |  | FLNA |
| 18.71679 | 20.32652 | 18.98367 | 20.47526 | 3 | 3 | 3 | 7.3 | 7.3 | 7.3 | 58.805 | 19.338 | 42764000 | 3 | DNA primase large subunit | PRIM2 |
| 21.34749 | 22.37547 | 18.98337 | 19.88464 | 7 | 7 | 7 | 9.9 | 9.9 | 9.9 | 114.71 | 56.125 | 73698000 | 10 | AP-3 complex subunit delta-1 | AP3D1 |
| 21.47447 | 18.06416 | 18.98317 | 20.01086 | 5 | 4 | 4 | 20.2 | 17.9 | 17.9 | 39.178 | 28.78 | 57129000 | 4 | Cyclin-dependent kinase 2 | CDK2 |
| 16.76046 | 23.31327 | 18.96097 | 19.11655 | 3 | 3 | 3 | 5.1 | 5.1 | 5.1 | 82.785 | 75.328 | 139340000 | 4 | Transcriptional repressor CTCF | CTCF |
| 19.22369 | 18.65805 | 18.96072 | 21.62578 | 5 | 5 | 5 | 2 | 2 | 2 | 376.01 | 30.218 | 54794000 | 5 | Golgin subfamily B member 1 | GOLGB1 |
| 24.39041 | 18.4281 | 18.95989 | 19.17786 | 47 | 1 | 1 | 56.1 | 2 | 2 | 85.223 | 16.317 | 472580000 | 2 | LIM domain and actin-binding protein 1 | LIMA1 |
| 20.0174 | 19.02376 | 18.9577 | 19.83204 | 3 | 3 | 3 | 13.3 | 13.3 | 13.3 | 41.943 | 21.352 | 26249000 | 4 | Prostaglandin E synthase 2;Prostaglandin E synthase 2 truncated form | PTGES2 |
| 18.07717 | 20.82641 | 18.95379 | 19.81234 | 3 | 3 | 3 | 4.1 | 4.1 | 4.1 | 121.82 | 16.708 | 13463000 | 3 | Ubiquitin conjugation factor E4 B | UBE4B |
| 17.8629 | 22.2196 | 18.94757 | 20.00128 | 5 | 5 | 5 | 9.5 | 9.5 | 9.5 | 75.035 | 49.639 | 101760000 | 6 | Pseudouridylate synthase 7 homolog | PUS7 |
| 20.35864 | 22.8756 | 18.94584 | 19.8407 | 3 | 3 | 3 | 9.2 | 9.2 | 9.2 | 56.547 | 20.435 | 79758000 | 3 | Target of EGR1 protein 1 | TOE1 |
| 22.44637 | 17.71779 | 18.94354 | 20.71263 | 3 | 3 | 3 | 6.5 | 6.5 | 6.5 | 76.749 | 40.216 | 250100000 | 2 | Protein kinase C alpha type | PRKCA |
| 17.12399 | 20.83144 | 18.94108 | 18.98245 | 3 | 2 | 2 | 1.5 | 1 | 1 | 202.81 | 10.912 | 33287000 | 3 | Unconventional myosin-Vc | MYO5C |
| 21.86676 | 18.94681 | 18.9398 | 18.31625 | 6 | 6 | 6 | 32.3 | 32.3 | 32.3 | 30.964 | 81.383 | 84026000 | 6 | Elongation factor Ts, mitochondrial | TSFM |
| 22.13649 | 17.51321 | 18.93626 | 18.8009 | 6 | 6 | 6 | 28.7 | 28.7 | 28.7 | 31.441 | 50.636 | 91352000 | 6 | DNA-directed RNA polymerase II subunit RPB3 | POLR2C |
| 22.44268 | 22.4997 | 18.93598 | 23.05999 | 4 | 4 | 4 | 10.5 | 10.5 | 10.5 | 52.269 | 36.172 | 182280000 | 10 | Cleavage and polyadenylation specificity factor subunit 6 | CPSF6 |
| 23.20403 | 18.20884 | 18.93103 | 19.82536 | 13 | 1 | 1 | 55.4 | 7.4 | 7.4 | 18.93 | 14.285 | 280460000 | 4 |  |  |
| 21.85407 | 20.73281 | 18.92673 | 20.41349 | 5 | 5 | 5 | 5.5 | 5.5 | 5.5 | 147.36 | 42.516 | 111060000 | 5 | Apoptotic chromatin condensation inducer in the nucleus | ACIN1 |
| 18.94222 | 21.19755 | 18.92051 | 21.556 | 3 | 3 | 3 | 4.9 | 4.9 | 4.9 | 58.634 | 18.554 | 49557000 | 4 | Copine-1 | CPNE1 |
| 19.23636 | 18.5925 | 18.91743 | 18.92662 | 3 | 2 | 2 | 11.2 | 8.1 | 8.1 | 33.229 | 12.988 | 29641000 | 2 | SRSF protein kinase 2;SRSF protein kinase 2 N-terminal;SRSF protein kinase 2 C-terminal | SRPK2 |
| 22.69939 | 22.40567 | 18.91372 | 21.94742 | 8 | 8 | 8 | 11.6 | 11.6 | 11.6 | 112.59 | 62.535 | 140610000 | 10 | Band 4.1-like protein 2 | EPB41L2 |
| 20.32389 | 22.9484 | 18.91365 | 20.41132 | 8 | 7 | 6 | 7.4 | 6.7 | 5.9 | 155.6 | 44.171 | 55658000 | 9 | Putative ATP-dependent RNA helicase DHX57 | DHX57 |
| 21.03355 | 21.89271 | 18.9049 | 20.27475 | 3 | 3 | 3 | 3.4 | 3.4 | 3.4 | 92.804 | 18.643 | 82077000 | 3 | Ubiquitin carboxyl-terminal hydrolase;Ubiquitin carboxyl-terminal hydrolase 7 | USP7 |
| 21.53792 | 21.24208 | 18.90289 | 19.97682 | 3 | 3 | 1 | 7.8 | 7.8 | 3.1 | 41.293 | 17.744 | 83082000 | 4 | Mitogen-activated protein kinase 14 | MAPK14 |
| 20.18362 | 21.66219 | 18.896 | 19.15634 | 4 | 4 | 4 | 2.9 | 2.9 | 2.9 | 155.68 | 23.931 | 35632000 | 6 | Golgin subfamily A member 3 | GOLGA3 |
| 21.46782 | 22.63014 | 18.89422 | 21.75609 | 6 | 6 | 6 | 10.5 | 10.5 | 10.5 | 66.077 | 41.634 | 122090000 | 8 | Pescadillo homolog | PES1 |
| 22.21582 | 20.45583 | 18.89184 | 20.5243 | 4 | 4 | 4 | 13.5 | 13.5 | 13.5 | 23.865 | 23.122 | 15254000 | 5 | Protein THEM6 | THEM6 |
| 23.22068 | 21.41741 | 18.89097 | 21.71146 | 10 | 10 | 10 | 8.1 | 8.1 | 8.1 | 177.44 | 77.886 | 345950000 | 12 | Nestin | NES |
| 23.15763 | 21.67263 | 18.88832 | 19.86415 | 3 | 3 | 3 | 10.1 | 10.1 | 10.1 | 29.975 | 20.115 | 41415000 | 6 | Small nuclear ribonucleoprotein-associated proteins B and B;Small nuclear ribonucleoprotein-associated protein N | SNRPB;SNRPN |
| 21.87987 | 18.34937 | 18.88637 | 19.31499 | 3 | 3 | 3 | 5.9 | 5.9 | 5.9 | 70.193 | 37.621 | 67889000 | 3 | U3 small nucleolar RNA-associated protein 6 homolog | UTP6 |
| 22.6977 | 21.37172 | 18.87668 | 19.52706 | 3 | 3 | 3 | 9.2 | 9.2 | 9.2 | 56.578 | 22.845 | 156120000 | 6 | Cytoplasmic dynein 1 light intermediate chain 1 | DYNC1LI1 |
| 23.76941 | 22.59792 | 18.87446 | 19.26685 | 3 | 3 | 3 | 27.4 | 27.4 | 27.4 | 13.057 | 21.204 | 53889000 | 4 | Actin-related protein 2/3 complex subunit 4 | ARPC4;ARPC4-TTLL3 |
| 22.1594 | 17.80002 | 18.87428 | 19.12572 | 18 | 2 | 2 | 6.6 | 0.8 | 0.8 | 287.28 | 12.26 | 81113000 | 3 | Filamin-C | FLNC |
| 21.35568 | 17.13561 | 18.87141 | 19.52733 | 3 | 3 | 3 | 11 | 11 | 11 | 43.933 | 19.307 | 72825000 | 3 | Alpha-globin transcription factor CP2 | TFCP2 |
| 21.7982 | 18.13391 | 18.8679 | 19.36456 | 3 | 3 | 3 | 2.7 | 2.7 | 2.7 | 117.15 | 37.96 | 71574000 | 3 | SAFB-like transcription modulator | SLTM |
| 20.20096 | 19.46165 | 18.86143 | 20.86319 | 3 | 3 | 3 | 14.5 | 14.5 | 14.5 | 30.658 | 19.564 | 23745000 | 3 | Voltage-dependent anion-selective channel protein 3 | VDAC3 |
| 23.22114 | 23.12679 | 18.85766 | 23.43007 | 6 | 6 | 6 | 14.4 | 14.4 | 14.4 | 73.747 | 60.811 | 434620000 | 9 | Kinesin-like protein KIFC1 | KIFC1 |
| 20.18942 | 17.19272 | 18.84899 | 20.02744 | 4 | 4 | 4 | 20.4 | 20.4 | 20.4 | 32.577 | 37.232 | 49423000 | 8 | Vesicular integral-membrane protein VIP36 | LMAN2 |
| 20.73653 | 18.76778 | 18.84855 | 20.26679 | 3 | 3 | 3 | 16.7 | 16.7 | 16.7 | 31.689 | 34.172 | 41453000 | 5 | Survival motor neuron protein | SMN1 |
| 16.99366 | 21.53051 | 18.84733 | 22.22747 | 4 | 4 | 4 | 10.9 | 10.9 | 10.9 | 67.867 | 28.068 | 59910000 | 4 | Acetolactate synthase-like protein | ILVBL |
| 18.74202 | 17.63195 | 18.84718 | 21.62158 | 3 | 3 | 3 | 4.8 | 4.8 | 4.8 | 66.966 | 17.604 | 22807000 | 3 | Phosphatidylinositide phosphatase SAC1 | SACM1L |
| 23.70102 | 21.1084 | 18.83898 | 21.43893 | 7 | 7 | 6 | 26.3 | 26.3 | 21.4 | 27.228 | 48.673 | 99692000 | 10 | Vesicle-associated membrane protein-associated protein B/C | VAPB |
| 20.08965 | 17.7636 | 18.83893 | 20.14069 | 7 | 2 | 2 | 24.1 | 10.3 | 10.3 | 37.567 | 13.35 | 19156000 | 2 | Guanine nucleotide-binding protein subunit beta-4 | GNB4 |
| 19.09258 | 22.8279 | 18.8373 | 19.42839 | 9 | 1 | 0 | 26.8 | 3 | 0 | 41.054 | 8.4553 | 117570000 | 6 | HLA class I histocompatibility antigen, A-34 alpha chain;HLA class I histocompatibility antigen, A-66 alpha chain;HLA class I histocompatibility antigen, A-26 alpha chain;HLA class I histocompatibility antigen, A-25 alpha chain;HLA class I histocompatibility antigen, A-43 alpha chain | HLA-A |
| 23.8292 | 24.84249 | 18.83342 | 26.85911 | 51 | 3 | 1 | 69.5 | 6.2 | 1.6 | 60.066 | 55.716 | 857700000 | 28 | Keratin, type II cytoskeletal 6B | KRT6B |
| 21.81683 | 22.8772 | 18.82783 | 18.75268 | 8 | 6 | 6 | 12.8 | 9 | 9 | 79.946 | 40.884 | 197880000 | 8 | ATP-dependent RNA helicase DDX50 | DDX50 |
| 18.6555 | 20.61369 | 18.82771 | 19.73823 | 4 | 4 | 4 | 14.7 | 14.7 | 14.7 | 29.188 | 23.383 | 18462000 | 5 | 3(2),5-bisphosphate nucleotidase 1 | BPNT1 |
| 22.26951 | 20.679 | 18.82387 | 20.96543 | 4 | 4 | 4 | 14.1 | 14.1 | 14.1 | 38.719 | 52.66 | 94926000 | 6 | Neuronal growth regulator 1 | NEGR1 |
| 22.66826 | 18.60593 | 18.82309 | 20.27238 | 3 | 3 | 3 | 4 | 4 | 4 | 86.823 | 21.24 | 185910000 | 5 | Cyclin-dependent kinase 11A;Cyclin-dependent kinase 11B | CDK11A;CDK11B |
| 17.51027 | 21.6977 | 18.82216 | 20.1517 | 8 | 2 | 2 | 22.5 | 6.5 | 6.5 | 46.908 | 13.924 | 27443000 | 3 | Rab GDP dissociation inhibitor alpha | GDI1 |
| 21.03093 | 21.41214 | 18.82011 | 20.2631 | 4 | 4 | 4 | 4.2 | 4.2 | 4.2 | 141.15 | 27.27 | 72878000 | 5 | Symplekin | SYMPK |
| 23.42008 | 24.14872 | 18.81967 | 18.78558 | 11 | 11 | 11 | 16.9 | 16.9 | 16.9 | 119.7 | 108.28 | 292360000 | 16 | Proline-, glutamic acid- and leucine-rich protein 1 | PELP1 |
| 20.25142 | 17.85209 | 18.80293 | 18.97944 | 5 | 1 | 1 | 17.2 | 5.6 | 5.6 | 38.836 | 7.7218 | 5229900 | 1 | Serpin B4 | SERPINB3;SERPINB4 |
| 19.8285 | 21.71837 | 18.80009 | 22.23298 | 3 | 3 | 3 | 3.2 | 3.2 | 3.2 | 124.84 | 33.001 | 113970000 | 4 | Exportin-7 | XPO7 |
| 20.94821 | 20.71339 | 18.79669 | 20.39121 | 3 | 3 | 3 | 5 | 5 | 5 | 75.042 | 20.476 | 64469000 | 4 | Kinesin-like protein KIF2A | KIF2A |
| 19.45288 | 19.25312 | 18.79386 | 19.95903 | 4 | 2 | 2 | 11.1 | 7 | 7 | 49.303 | 14.135 | 16978000 | 2 | Septin-6 | 06. Sep |
| 21.21656 | 18.52823 | 18.77826 | 19.32494 | 4 | 3 | 3 | 20.2 | 17.5 | 17.5 | 33.304 | 20.656 | 47287000 | 3 | Cyclin-dependent-like kinase 5 | CDK5 |
| 19.64222 | 21.81828 | 18.7773 | 20.2665 | 3 | 3 | 3 | 1.8 | 1.8 | 1.8 | 217.49 | 17.545 | 23756000 | 4 | Zinc finger C3H1 domain-containing protein | ZFC3H1 |
| 25.96869 | 18.89525 | 18.77684 | 20.16477 | 8 | 8 | 8 | 31.4 | 31.4 | 31.4 | 39.183 | 131.69 | 1302100000 | 10 | Core histone macro-H2A.1;Histone H2A | H2AFY |
| 19.3107 | 27.23114 | 18.7763 | 19.118 | 10 | 1 | 1 | 15.4 | 1.7 | 1.7 | 71.027 | 15.831 | 6693800000 | 10 | Heat shock 70 kDa protein 6;Putative heat shock 70 kDa protein 7 | HSPA6;HSPA7 |
| 20.77 | 17.73108 | 18.77518 | 20.11526 | 4 | 4 | 4 | 3.6 | 3.6 | 3.6 | 170.51 | 24.608 | 30687000 | 3 | Rho GTPase-activating protein 35 | ARHGAP35;GRLF1 |
| 18.45442 | 24.12266 | 18.77177 | 19.54354 | 37 | 2 | 2 | 91.5 | 4.8 | 4.8 | 41.736 | 82.95 | 8173000000 | 28 | Actin, cytoplasmic 1;Actin, cytoplasmic 1, N-terminally processed | ACTB |
| 24.14577 | 17.38656 | 18.77085 | 19.86224 | 3 | 3 | 3 | 26.2 | 26.2 | 26.2 | 22.882 | 33.147 | 98696000 | 6 | Protein FAM101B | FAM101B |
| 20.71967 | 21.31013 | 18.76892 | 22.70738 | 3 | 3 | 3 | 9 | 9 | 9 | 51.354 | 19.151 | 93599000 | 4 | Calcium-binding mitochondrial carrier protein SCaMC-1 | SLC25A24 |
| 23.08641 | 19.60152 | 18.76772 | 18.7375 | 31 | 2 | 2 | 39 | 2.7 | 2.7 | 79.361 | 31.301 | 220500000 | 2 | Nexilin | NEXN |
| 23.68442 | 19.69919 | 18.76431 | 19.0729 | 4 | 4 | 1 | 20.1 | 20.1 | 3.7 | 21.664 | 26.23 | 51962000 | 8 | Programmed cell death protein 6 | PDCD6 |
| 20.0355 | 17.91371 | 18.76373 | 19.81412 | 3 | 3 | 3 | 0.8 | 0.8 | 0.8 | 527.22 | 22.213 | 72399000 | 3 | E3 ubiquitin-protein ligase HERC2 | HERC2 |
| 18.38483 | 18.26734 | 18.76351 | 19.82693 | 11 | 1 | 1 | 43.6 | 4.7 | 4.7 | 33.8 | 9.8771 | 16977000 | 1 | Poly(rC)-binding protein 2 | PCBP2 |
| 25.2442 | 21.58385 | 18.7544 | 19.67406 | 20 | 20 | 20 | 19.9 | 19.9 | 19.9 | 121.89 | 239.38 | 694250000 | 20 | Voltage-dependent calcium channel subunit alpha-2/delta-1;Voltage-dependent calcium channel subunit alpha-2-1;Voltage-dependent calcium channel subunit delta-1 | CACNA2D1 |
| 20.94271 | 21.23876 | 18.75403 | 21.9636 | 4 | 4 | 4 | 4.3 | 4.3 | 4.3 | 118.27 | 25.296 | 125960000 | 3 | Nuclear pore complex protein Nup133 | NUP133 |
| 21.3399 | 21.11705 | 18.75211 | 20.78327 | 3 | 3 | 3 | 4.6 | 4.6 | 4.6 | 99.061 | 20.023 | 106580000 | 3 | Exocyst complex component 4 | EXOC4 |
| 22.32808 | 21.37666 | 18.74099 | 21.78413 | 4 | 4 | 4 | 11.8 | 11.8 | 11.8 | 50.364 | 31.948 | 123180000 | 7 | 28S ribosomal protein S30, mitochondrial | MRPS30 |
| 18.38249 | 17.51181 | 18.74067 | 20.02354 | 66 | 1 | 0 | 75 | 2 | 0 | 64.168 | 8.8535 | 0 | 1 | Caldesmon | CALD1 |
| 18.97446 | 18.13837 | 18.74006 | 20.81323 | 3 | 3 | 3 | 8.7 | 8.7 | 8.7 | 43.206 | 16.565 | 31999000 | 3 | Acyl-coenzyme A thioesterase 9, mitochondrial | ACOT9 |
| 20.77443 | 19.61981 | 18.73854 | 20.10871 | 3 | 3 | 3 | 4.2 | 4.2 | 4.2 | 75.143 | 18.946 | 36615000 | 3 | Acetoacetyl-CoA synthetase | AACS |
| 21.03644 | 18.92063 | 18.73073 | 19.4845 | 3 | 2 | 2 | 3.4 | 2.4 | 2.4 | 119.94 | 12.978 | 80964000 | 2 | Importin-8 | IPO8 |
| 20.43094 | 18.5137 | 18.72998 | 19.68182 | 3 | 3 | 3 | 14.6 | 14.6 | 14.6 | 30.446 | 23.041 | 28131000 | 2 | Probable U3 small nucleolar RNA-associated protein 11 | UTP11L |
| 23.9657 | 24.14141 | 18.72892 | 24.23125 | 19 | 19 | 19 | 16.3 | 16.3 | 16.3 | 162.12 | 164.54 | 456450000 | 28 | Nuclear pore complex protein Nup160 | NUP160 |
| 18.1371 | 21.51369 | 18.72129 | 21.51099 | 3 | 3 | 3 | 8.3 | 8.3 | 8.3 | 49.966 | 21.583 | 42910000 | 6 | Tyrosine-protein phosphatase non-receptor type 1;Tyrosine-protein phosphatase non-receptor type | PTPN1 |
| 24.63389 | 18.8905 | 18.72122 | 18.47973 | 10 | 10 | 10 | 11 | 11 | 11 | 113.86 | 80.075 | 556100000 | 12 | Rho GTPase-activating protein 11A | ARHGAP11A |
| 16.86314 | 22.64832 | 18.71361 | 22.47704 | 4 | 4 | 4 | 10.1 | 10.1 | 10.1 | 57.398 | 31.272 | 75975000 | 7 | Amidophosphoribosyltransferase | PPAT |
| 23.6184 | 21.8994 | 18.7 | 21.40442 | 4 | 4 | 4 | 27 | 27 | 27 | 19.889 | 30.528 | 43750000 | 7 | RNA-binding protein 8A | RBM8A |
| 22.68069 | 22.99905 | 18.69581 | 22.3314 | 7 | 7 | 7 | 10.7 | 10.7 | 10.7 | 81.613 | 48.454 | 346040000 | 9 | Pinin | PNN |
| 19.94978 | 21.72505 | 18.69546 | 19.09429 | 3 | 3 | 3 | 1.6 | 1.6 | 1.6 | 263.25 | 20.415 | 65036000 | 5 | Ankyrin repeat domain-containing protein 17 | ANKRD17 |
| 25.3964 | 19.22287 | 18.69012 | 23.48812 | 4 | 1 | 1 | 19.9 | 8.1 | 8.1 | 15.43 | 7.3674 | 150630000 | 4 | Histone H3 | HIST2H3PS2 |
| 22.6778 | 21.52597 | 18.67101 | 20.14626 | 5 | 5 | 5 | 8.7 | 8.7 | 8.7 | 95.533 | 61.974 | 149490000 | 6 | BMP-2-inducible protein kinase | BMP2K |
| 17.43918 | 18.34303 | 18.66472 | 22.77036 | 3 | 3 | 3 | 12.2 | 12.2 | 12.2 | 39.933 | 21.506 | 59017000 | 6 | Synapse-associated protein 1 | SYAP1 |
| 19.17023 | 23.02803 | 18.65995 | 19.83441 | 5 | 5 | 5 | 6.9 | 6.9 | 6.9 | 98.594 | 35.611 | 104510000 | 6 | ATP-dependent RNA helicase DDX54 | DDX54 |
| 17.65609 | 21.75116 | 18.65856 | 19.00113 | 4 | 1 | 1 | 35.3 | 12.9 | 12.9 | 15.584 | 25.593 | 83734000 | 3 |  |  |
| 22.14255 | 21.76381 | 18.65842 | 21.56909 | 3 | 3 | 2 | 10.9 | 10.9 | 7.2 | 25.497 | 18.847 | 21725000 | 7 | Protein NipSnap homolog 1 | NIPSNAP1 |
| 21.52148 | 22.45736 | 18.65386 | 22.30975 | 6 | 6 | 6 | 6 | 6 | 6 | 118.7 | 40.089 | 89137000 | 7 | Large proline-rich protein BAG6 | BAG6 |
| 20.10917 | 17.8114 | 18.64828 | 18.61268 | 3 | 3 | 3 | 14.8 | 14.8 | 14.8 | 26.152 | 22.896 | 22298000 | 3 | Galectin-3;Galectin | LGALS3 |
| 20.28636 | 17.76199 | 18.64492 | 19.215 | 4 | 4 | 4 | 5.3 | 5.3 | 5.3 | 102.7 | 26.293 | 33717000 | 4 | RNA-binding protein 12B | RBM12B |
| 18.09373 | 22.93191 | 18.64242 | 22.8452 | 3 | 3 | 3 | 10.8 | 10.8 | 10.8 | 43.28 | 21.844 | 123130000 | 8 | Cleft lip and palate transmembrane protein 1-like protein | CLPTM1L |
| 21.51518 | 18.57045 | 18.63503 | 20.11295 | 3 | 3 | 3 | 5.8 | 5.8 | 5.8 | 68.456 | 28.627 | 61898000 | 3 | Leucine-rich repeat and calponin homology domain-containing protein 3 | LRCH3 |
| 17.95547 | 16.93911 | 18.63101 | 21.87905 | 3 | 2 | 2 | 4.1 | 2.8 | 2.8 | 84.765 | 12.387 | 3857000 | 2 | Catenin beta-1 | CTNNB1 |
| 20.92651 | 21.50237 | 18.62926 | 21.58733 | 7 | 7 | 7 | 8.7 | 8.7 | 8.7 | 111.84 | 51.04 | 96873000 | 7 | DNA polymerase delta catalytic subunit | POLD1 |
| 18.34962 | 21.14675 | 18.62866 | 19.73256 | 29 | 1 | 1 | 28.2 | 2 | 2 | 109.44 | 25.708 | 55173000 | 2 | Neutral alpha-glucosidase AB | GANAB |
| 16.61942 | 21.3078 | 18.62521 | 20.06703 | 3 | 3 | 3 | 8.8 | 8.8 | 8.8 | 63.132 | 25.868 | 84559000 | 2 | Protein AATF | AATF |
| 20.08563 | 20.89888 | 18.62511 | 19.501 | 4 | 4 | 4 | 3 | 3 | 3 | 185.85 | 22.991 | 25783000 | 3 | Protein MON2 homolog | MON2 |
| 22.26554 | 21.1187 | 18.6235 | 18.78408 | 3 | 3 | 3 | 29.5 | 29.5 | 29.5 | 14.745 | 17.733 | 18957000 | 3 | NudC domain-containing protein 2 | NUDCD2 |
| 21.79943 | 22.33854 | 18.61591 | 22.5467 | 5 | 5 | 5 | 11.8 | 11.8 | 11.8 | 61.312 | 38.076 | 107010000 | 9 | Serine--tRNA ligase, cytoplasmic | SARS |
| 23.99386 | 19.74235 | 18.60864 | 19.35222 | 6 | 6 | 1 | 11.8 | 11.8 | 1.8 | 73.666 | 81.359 | 403210000 | 8 | Synaptopodin | SYNPO |
| 19.72988 | 21.55159 | 18.60765 | 21.35933 | 4 | 4 | 4 | 1.6 | 1.6 | 1.6 | 333.2 | 24.07 | 180180000 | 5 | Collagen alpha-1(XII) chain | COL12A1 |
| 17.95673 | 18.49479 | 18.607 | 19.00767 | 3 | 2 | 2 | 8.3 | 5.1 | 5.1 | 41.647 | 10.999 | 3118800 | 2 | Aldehyde dehydrogenase, dimeric NADP-preferring | ALDH3A1 |
| 18.56194 | 18.36931 | 18.6034 | 19.24538 | 28 | 1 | 1 | 22.6 | 0.6 | 0.6 | 158.64 | 6.5204 | 6510100 | 1 | Eukaryotic translation initiation factor 4 gamma 1 | EIF4G1 |
| 21.46239 | 20.97113 | 18.59692 | 21.58971 | 3 | 3 | 3 | 6.8 | 6.8 | 6.8 | 53.29 | 18.779 | 80429000 | 4 | Coiled-coil domain-containing protein 6 | CCDC6 |
| 23.56929 | 18.44689 | 18.59415 | 19.66928 | 4 | 1 | 1 | 20.9 | 4.7 | 4.7 | 21.868 | 6.3408 | 35367000 | 1 | Programmed cell death protein 6 | PDCD6 |
| 21.60137 | 22.57697 | 18.58945 | 19.78375 | 6 | 6 | 6 | 5.5 | 5.5 | 5.5 | 155.98 | 62.951 | 74770000 | 8 | Rab3 GTPase-activating protein non-catalytic subunit | RAB3GAP2 |
| 22.12943 | 21.76753 | 18.5891 | 19.45119 | 4 | 4 | 4 | 6.5 | 6.5 | 6.5 | 95.222 | 41.477 | 170590000 | 6 | Lysine-specific histone demethylase 1A | KDM1A |
| 21.89844 | 22.63168 | 18.58729 | 21.78975 | 6 | 6 | 6 | 14 | 14 | 14 | 50.617 | 37.512 | 152660000 | 8 | U1 small nuclear ribonucleoprotein 70 kDa | SNRNP70 |
| 19.66915 | 20.60811 | 18.5868 | 21.20401 | 3 | 3 | 3 | 3.6 | 3.6 | 3.6 | 99.326 | 17.493 | 62849000 | 3 | Major vault protein | MVP |
| 21.05933 | 21.07607 | 18.57761 | 20.54296 | 4 | 4 | 4 | 8.4 | 8.4 | 8.4 | 64.009 | 44.145 | 62582000 | 5 | 5-AMP-activated protein kinase catalytic subunit alpha-1 | PRKAA1 |
| 21.44521 | 18.23171 | 18.57096 | 20.62961 | 3 | 3 | 3 | 6 | 6 | 6 | 55.142 | 19.715 | 59289000 | 2 | CUGBP Elav-like family member 1 | CELF1 |
| 21.95205 | 18.33883 | 18.57035 | 21.9732 | 7 | 7 | 7 | 13.3 | 13.3 | 13.3 | 73.562 | 44.548 | 103190000 | 8 | Aspartate--tRNA ligase, mitochondrial | DARS2 |
| 23.85119 | 21.29659 | 18.56865 | 20.61162 | 5 | 5 | 5 | 33.1 | 33.1 | 33.1 | 20.913 | 32.715 | 104100000 | 8 | Serine/arginine-rich splicing factor 10 | SRSF10 |
| 22.09697 | 17.93634 | 18.56478 | 19.7224 | 3 | 3 | 3 | 5.3 | 5.3 | 5.3 | 96.955 | 32.531 | 121040000 | 3 | Oxysterol-binding protein-related protein 8;Oxysterol-binding protein | OSBPL8 |
| 18.83931 | 21.8469 | 18.55965 | 23.02455 | 5 | 2 | 2 | 13 | 5.8 | 5.8 | 65.695 | 20.075 | 107380000 | 4 | Ubiquilin-2 | UBQLN2 |
| 22.26854 | 20.28093 | 18.55751 | 18.76095 | 3 | 3 | 3 | 14.5 | 14.5 | 14.5 | 25.565 | 17.164 | 16357000 | 3 | Cytochrome c oxidase subunit 2 | MT-CO2 |
| 22.28782 | 18.77589 | 18.55136 | 19.15481 | 44 | 3 | 3 | 85 | 9.3 | 9.3 | 28.955 | 20.363 | 99362000 | 3 | Tropomyosin alpha-3 chain | TPM3 |
| 17.00514 | 19.475 | 18.54728 | 18.50108 | 3 | 1 | 1 | 24 | 10 | 10 | 17.107 | 6.8431 | 7999700 | 1 | ADP-ribosylation factor 5 | ARF5 |
| 25.42595 | 17.4461 | 18.54726 | 19.38379 | 5 | 5 | 5 | 39.4 | 39.4 | 39.4 | 16.183 | 33.656 | 141870000 | 8 | Allograft inflammatory factor 1-like | AIF1L |
| 16.99101 | 23.66557 | 18.54685 | 20.40527 | 3 | 3 | 3 | 10.4 | 10.4 | 10.4 | 52.13 | 21.315 | 36585000 | 5 | Semenogelin-1;Alpha-inhibin-92;Alpha-inhibin-31;Seminal basic protein | SEMG1 |
| 18.25972 | 20.26774 | 18.54515 | 22.38416 | 4 | 4 | 4 | 7.7 | 7.7 | 7.7 | 68.168 | 26.061 | 52091000 | 6 | Aminopeptidase B | RNPEP |
| 19.3207 | 20.2376 | 18.54496 | 19.2583 | 4 | 1 | 1 | 7.7 | 1.9 | 1.9 | 57.81 | 7.1915 | 38748000 | 1 | Importin subunit alpha-4 | KPNA3 |
| 25.51539 | 17.56419 | 18.54465 | 20.8749 | 10 | 10 | 10 | 18.7 | 18.7 | 18.7 | 59.763 | 96.204 | 896370000 | 11 | Coronin-2A | CORO2A |
| 20.33484 | 20.99479 | 18.52571 | 19.93918 | 3 | 3 | 3 | 12.6 | 12.6 | 12.6 | 26.614 | 21.096 | 34919000 | 4 | 39S ribosomal protein L1, mitochondrial | MRPL1 |
| 27.72689 | 20.82221 | 18.52565 | 19.55348 | 66 | 6 | 0 | 76.9 | 6.8 | 0 | 61.212 | 158.69 | 5598800000 | 13 | Caldesmon | CALD1 |
| 17.89053 | 21.88684 | 18.51992 | 20.28525 | 3 | 3 | 3 | 6.6 | 6.6 | 6.6 | 81.673 | 20.417 | 65905000 | 3 | Acylamino-acid-releasing enzyme | APEH |
| 24.32169 | 22.16057 | 18.50655 | 18.86402 | 11 | 11 | 11 | 13.5 | 13.5 | 13.5 | 126.48 | 112.24 | 435020000 | 14 | Suppression of tumorigenicity 5 protein | ST5 |
| 19.12312 | 17.20568 | 18.50648 | 21.82811 | 13 | 2 | 2 | 21.8 | 3.9 | 3.9 | 85.524 | 13.537 | 29012000 | 4 |  | CLUH |
| 22.01781 | 17.50506 | 18.50083 | 19.53252 | 3 | 3 | 3 | 10.1 | 10.1 | 10.1 | 29.819 | 19.857 | 116910000 | 4 | Folate receptor alpha | FOLR1 |
| 22.48008 | 18.13209 | 18.49025 | 18.73225 | 6 | 6 | 6 | 12.7 | 12.7 | 12.7 | 61.57 | 46.03 | 102910000 | 6 | Actin-binding LIM protein 3 | ABLIM3 |
| 21.55651 | 18.33042 | 18.48921 | 19.84131 | 3 | 3 | 3 | 11.1 | 11.1 | 11.1 | 57.707 | 26.115 | 74447000 | 2 | U3 small nucleolar RNA-associated protein 18 homolog | UTP18 |
| 20.68953 | 22.36938 | 18.48866 | 19.74658 | 4 | 4 | 4 | 9.3 | 9.3 | 9.3 | 81.746 | 42.379 | 112300000 | 5 | TRMT1-like protein | TRMT1L |
| 21.74583 | 18.59626 | 18.48832 | 19.15294 | 5 | 5 | 5 | 4.4 | 4.4 | 4.4 | 197.49 | 32.192 | 83189000 | 5 | Signal-induced proliferation-associated 1-like protein 1 | SIPA1L1 |
| 20.41803 | 21.52373 | 18.48674 | 19.42518 | 3 | 3 | 3 | 7.4 | 7.4 | 7.4 | 50.381 | 21.301 | 38281000 | 4 | G patch domain-containing protein 4 | GPATCH4 |
| 19.7902 | 22.76776 | 18.48379 | 19.79608 | 4 | 4 | 4 | 3.3 | 3.3 | 3.3 | 133.5 | 24.498 | 41214000 | 4 | RNA polymerase-associated protein CTR9 homolog | CTR9 |
| 22.14957 | 21.83422 | 18.48257 | 21.17264 | 6 | 6 | 6 | 8.4 | 8.4 | 8.4 | 98.119 | 40.912 | 190330000 | 8 | Molybdenum cofactor sulfurase | MOCOS |
| 21.55103 | 16.42805 | 18.48214 | 19.4224 | 3 | 3 | 3 | 9.3 | 9.3 | 9.3 | 51.008 | 19.911 | 54051000 | 3 | Inositol-trisphosphate 3-kinase A | ITPKA |
| 20.93207 | 20.67943 | 18.47849 | 21.2307 | 4 | 3 | 3 | 7 | 5.5 | 5.5 | 86.478 | 29.149 | 96990000 | 3 | Protein transport protein Sec23B | SEC23B |
| 17.94888 | 20.97862 | 18.47755 | 21.94339 | 3 | 3 | 3 | 35.3 | 35.3 | 35.3 | 9.8703 | 18.15 | 60519000 | 4 | Translocation protein SEC62 | SEC62 |
| 17.47486 | 19.28046 | 18.45524 | 20.79623 | 10 | 1 | 1 | 9.1 | 1 | 1 | 145.18 | 7.3045 | 10434000 | 1 | Cytoplasmic FMR1-interacting protein 1 | CYFIP1 |
| 18.57089 | 21.48753 | 18.44908 | 19.37577 | 6 | 1 | 1 | 19.1 | 4.4 | 4.4 | 36.526 | 24.219 | 28965000 | 4 | Ig alpha-2 chain C region | IGHA2 |
| 17.68624 | 21.45151 | 18.44778 | 17.99567 | 76 | 1 | 1 | 56.6 | 0.7 | 0.7 | 145.04 | 6.8702 | 126200000 | 2 | Unconventional myosin-VI | MYO6 |
| 17.45663 | 19.05982 | 18.4423 | 28.12391 | 3 | 3 | 3 | 6.9 | 6.9 | 6.9 | 46.277 | 16.842 | 2201500000 | 12 | Acyl-coenzyme A thioesterase 1;Acyl-coenzyme A thioesterase 2, mitochondrial | ACOT1;ACOT2 |
| 21.31617 | 18.6278 | 18.43774 | 22.79733 | 6 | 6 | 6 | 7.2 | 7.2 | 7.2 | 136.31 | 42.904 | 90657000 | 7 | Exportin-5 | XPO5 |
| 21.17579 | 17.89727 | 18.43641 | 19.51445 | 3 | 3 | 3 | 10.6 | 10.6 | 10.6 | 57.858 | 25.746 | 67471000 | 3 | EH domain-containing protein 1 | EHD1 |
| 20.7496 | 17.47541 | 18.42069 | 19.40165 | 3 | 3 | 3 | 3.5 | 3.5 | 3.5 | 128.95 | 21.027 | 31665000 | 3 | Protein diaphanous homolog 3 | DIAPH3 |
| 18.98365 | 17.34366 | 18.41497 | 21.4807 | 3 | 3 | 3 | 6.1 | 6.1 | 6.1 | 52.743 | 16.449 | 63054000 | 3 | Serine palmitoyltransferase 1 | SPTLC1 |
| 24.2844 | 16.15399 | 18.41111 | 19.71984 | 43 | 2 | 0 | 87.1 | 11.7 | 0 | 28.922 | 62.353 | 448660000 | 3 | Tropomyosin alpha-3 chain | TPM3 |
| 22.34546 | 19.43909 | 18.39889 | 18.71318 | 6 | 2 | 2 | 40.1 | 12.2 | 12.2 | 19.827 | 13.804 | 21130000 | 3 | Myosin regulatory light polypeptide 9 | MYL9 |
| 18.40852 | 18.77263 | 18.39735 | 21.65364 | 4 | 4 | 4 | 18.6 | 18.6 | 18.6 | 37.325 | 29.389 | 21288000 | 4 | NSFL1 cofactor p47 | NSFL1C |
| 18.28025 | 22.38343 | 18.39347 | 22.27759 | 6 | 6 | 6 | 13.5 | 13.5 | 13.5 | 62.846 | 43.244 | 64618000 | 8 | Nicalin | NCLN |
| 22.61982 | 18.43534 | 18.37773 | 19.3931 | 3 | 3 | 3 | 12.4 | 12.4 | 12.4 | 45.476 | 41.571 | 124200000 | 3 | Serine/threonine-protein kinase VRK1 | VRK1 |
| 23.93337 | 18.15807 | 18.37317 | 19.33435 | 10 | 10 | 10 | 10.8 | 10.8 | 10.8 | 136.38 | 158.72 | 288920000 | 10 | Uncharacterized protein KIAA1211 | KIAA1211 |
| 21.49287 | 19.1696 | 18.37008 | 20.24684 | 3 | 3 | 3 | 5.1 | 5.1 | 5.1 | 76.644 | 19.221 | 64895000 | 3 | G2 and S phase-expressed protein 1 | GTSE1 |
| 19.91813 | 17.3189 | 18.36743 | 23.97937 | 5 | 5 | 5 | 5.8 | 5.8 | 5.8 | 87.081 | 30.103 | 51311000 | 6 | Plakophilin-3 | PKP3 |
| 22.95065 | 20.58774 | 18.35451 | 18.39158 | 6 | 6 | 6 | 38 | 38 | 38 | 25.468 | 34.903 | 20705000 | 6 | Thymidine kinase, cytosolic | TK1 |
| 18.56931 | 18.80032 | 18.35251 | 17.99068 | 15 | 1 | 1 | 18.6 | 1.7 | 1.7 | 70.02 | 6.4315 | 13307000 | 1 | Heat shock-related 70 kDa protein 2 | HSPA2 |
| 23.07174 | 23.82503 | 18.35028 | 22.14526 | 7 | 7 | 7 | 16 | 16 | 16 | 65.572 | 57.553 | 217810000 | 11 | Guanine nucleotide-binding protein-like 3-like protein | GNL3L |
| 22.09359 | 21.84399 | 18.34863 | 21.24458 | 3 | 3 | 2 | 3.8 | 3.8 | 2.8 | 90.193 | 26.165 | 205620000 | 3 | AFG3-like protein 2 | AFG3L2 |
| 20.65779 | 21.15487 | 18.34764 | 20.46033 | 3 | 3 | 3 | 10.1 | 10.1 | 10.1 | 39.336 | 18.993 | 56631000 | 5 | RNA 3-terminal phosphate cyclase | RTCA |
| 17.70375 | 21.37952 | 18.34651 | 19.70645 | 3 | 3 | 3 | 5 | 5 | 5 | 101.27 | 88.202 | 72823000 | 3 | General transcription factor 3C polypeptide 3 | GTF3C3 |
| 22.28526 | 23.36199 | 18.34074 | 23.33511 | 4 | 4 | 4 | 23.8 | 23.8 | 23.8 | 40.691 | 52.702 | 189440000 | 12 | Protein TFG | TFG |
| 18.07328 | 24.35212 | 18.3366 | 20.4822 | 9 | 1 | 1 | 42.1 | 5.5 | 5.5 | 17.256 | 6.4367 | 38760000 | 2 | 60S ribosomal protein L26-like 1 | RPL26L1 |
| 23.03617 | 17.87783 | 18.33134 | 19.60593 | 3 | 3 | 3 | 18.9 | 18.9 | 18.9 | 22.119 | 20.093 | 20702000 | 4 | Flavin reductase (NADPH) | BLVRB |
| 18.59598 | 22.31197 | 18.32996 | 19.09505 | 3 | 3 | 3 | 4.8 | 4.8 | 4.8 | 80.945 | 18.702 | 70110000 | 3 | Probable ATP-dependent RNA helicase DDX31 | DDX31 |
| 20.82198 | 18.96198 | 18.32938 | 20.03809 | 3 | 3 | 3 | 4.1 | 4.1 | 4.1 | 115.15 | 16.879 | 49977000 | 2 | Zinc finger RNA-binding protein | ZFR |
| 22.61547 | 21.06303 | 18.32825 | 21.37586 | 3 | 3 | 3 | 14.8 | 14.8 | 14.8 | 23.411 | 19.351 | 19778000 | 4 | Adenylate kinase isoenzyme 1 | AK1 |
| 18.72498 | 21.08531 | 18.32824 | 19.47742 | 3 | 3 | 3 | 2.2 | 2.2 | 2.2 | 166.46 | 16.458 | 18511000 | 3 | DNA polymerase;DNA polymerase alpha catalytic subunit | POLA1 |
| 22.10457 | 22.49495 | 18.32594 | 21.44546 | 3 | 3 | 3 | 8.1 | 8.1 | 8.1 | 57.274 | 21.383 | 120950000 | 4 | Probable ATP-dependent RNA helicase DDX56 | DDX56 |
| 18.18729 | 20.61342 | 18.32287 | 18.10663 | 3 | 2 | 2 | 5.9 | 4.4 | 4.4 | 60.386 | 13.178 | 50770000 | 3 | Prolyl 3-hydroxylase 2 | LEPREL1 |
| 21.66982 | 22.6227 | 18.30798 | 22.85455 | 4 | 4 | 4 | 6.9 | 6.9 | 6.9 | 83.353 | 31.69 | 189540000 | 10 | LETM1 and EF-hand domain-containing protein 1, mitochondrial | LETM1 |
| 21.18156 | 16.00086 | 18.30069 | 18.90544 | 5 | 5 | 5 | 25.7 | 25.7 | 25.7 | 29.299 | 34.788 | 48916000 | 5 | Uridine-cytidine kinase 2 | UCK2 |
| 17.36162 | 20.08719 | 18.29782 | 19.64224 | 15 | 1 | 1 | 28.7 | 2.3 | 2.3 | 70.403 | 7.4372 | 34382000 | 1 | Heat shock 70 kDa protein 1-like | HSPA1L |
| 18.72787 | 20.32904 | 18.29612 | 18.26275 | 4 | 1 | 1 | 6.1 | 1.7 | 1.7 | 84.016 | 8.5129 | 38868000 | 1 | Cullin-4B | CUL4B |
| 17.92436 | 21.51412 | 18.29232 | 20.17321 | 4 | 4 | 4 | 4.4 | 4.4 | 4.4 | 131.2 | 25.223 | 23412000 | 5 | 1-phosphatidylinositol 4,5-bisphosphate phosphodiesterase beta-3 | PLCB3 |
| 21.75205 | 21.66371 | 18.28986 | 19.16231 | 5 | 5 | 5 | 3.5 | 3.5 | 3.5 | 205.12 | 31.806 | 74081000 | 6 | Plexin-B2 | PLXNB2 |
| 19.97281 | 21.78185 | 18.28971 | 19.48312 | 3 | 3 | 3 | 11.8 | 11.8 | 11.8 | 31.791 | 21.736 | 25072000 | 4 | Thioredoxin-related transmembrane protein 1 | TMX1 |
| 24.0385 | 22.43997 | 18.26861 | 20.6368 | 6 | 6 | 6 | 13.8 | 13.8 | 13.8 | 49.844 | 36.469 | 336810000 | 9 | PDZ and LIM domain protein 7 | PDLIM7 |
| 22.20138 | 23.67681 | 18.26822 | 24.09329 | 8 | 8 | 8 | 11.6 | 11.6 | 11.6 | 80.529 | 54.47 | 247860000 | 15 | Dolichyl-diphosphooligosaccharide--protein glycosyltransferase subunit STT3A | STT3A |
| 27.99766 | 17.98385 | 18.26751 | 18.93088 | 20 | 20 | 20 | 43.7 | 43.7 | 43.7 | 67.942 | 323.31 | 5224400000 | 31 | Phostensin | PPP1R18 |
| 20.67133 | 18.50908 | 18.26637 | 20.28867 | 3 | 3 | 3 | 3.5 | 3.5 | 3.5 | 106.02 | 21.658 | 44233000 | 3 | DNA ligase 3 | LIG3 |
| 22.55595 | 19.50318 | 18.26406 | 18.95717 | 4 | 4 | 4 | 14.2 | 14.2 | 14.2 | 41.439 | 37.302 | 116100000 | 4 | Zinc finger protein RFP | TRIM27 |
| 23.49338 | 20.2181 | 18.25932 | 20.09109 | 3 | 3 | 3 | 12.9 | 12.9 | 12.9 | 30.241 | 19.981 | 32421000 | 5 | NADH dehydrogenase [ubiquinone] iron-sulfur protein 3, mitochondrial | NDUFS3 |
| 23.57854 | 22.60831 | 18.2548 | 24.06439 | 6 | 6 | 6 | 35.5 | 35.5 | 35.5 | 31.362 | 85.264 | 206030000 | 13 | Complement component 1 Q subcomponent-binding protein, mitochondrial | C1QBP |
| 19.69549 | 17.20963 | 18.25364 | 19.82274 | 3 | 2 | 2 | 1.3 | 0.9 | 0.9 | 304.1 | 21.993 | 72531000 | 5 | Inositol 1,4,5-trisphosphate receptor type 3 | ITPR3 |
| 22.87993 | 22.55161 | 18.25087 | 22.13549 | 5 | 2 | 2 | 12.6 | 5.4 | 5.4 | 60.221 | 29.017 | 212060000 | 7 | Importin subunit alpha-5;Importin subunit alpha-5, N-terminally processed | KPNA1 |
| 20.82345 | 21.37353 | 18.25033 | 20.3717 | 4 | 4 | 4 | 3 | 3 | 3 | 178.7 | 25.962 | 44764000 | 5 | Bromodomain adjacent to zinc finger domain protein 1A | BAZ1A |
| 21.813 | 21.48424 | 18.24503 | 18.91978 | 3 | 3 | 3 | 7.9 | 7.9 | 7.9 | 52.29 | 19.17 | 82924000 | 4 | General transcription factor 3C polypeptide 5 | GTF3C5 |
| 20.85886 | 18.31119 | 18.23514 | 18.10288 | 3 | 3 | 3 | 5.9 | 5.9 | 5.9 | 70.832 | 18.936 | 51530000 | 3 | Bifunctional 3-phosphoadenosine 5-phosphosulfate synthase 1;Sulfate adenylyltransferase;Adenylyl-sulfate kinase | PAPSS1 |
| 23.04554 | 17.79124 | 18.23405 | 19.48445 | 3 | 3 | 3 | 10.6 | 10.6 | 10.6 | 40.251 | 37.504 | 169460000 | 3 | Retinoic acid-induced protein 3 | GPRC5A |
| 22.45096 | 22.05798 | 18.22774 | 21.98961 | 9 | 9 | 9 | 9.7 | 9.7 | 9.7 | 121.69 | 60.155 | 142440000 | 9 | Transcription elongation regulator 1 | TCERG1 |
| 18.27901 | 21.85897 | 18.22416 | 20.85095 | 3 | 3 | 3 | 27.7 | 27.7 | 27.7 | 15.161 | 19.84 | 58238000 | 5 | Glycylpeptide N-tetradecanoyltransferase 1 | NMT1 |
| 22.15601 | 21.98465 | 18.2198 | 19.11379 | 7 | 7 | 7 | 5.5 | 5.5 | 5.5 | 182.77 | 45.274 | 90032000 | 6 | THO complex subunit 2 | THOC2 |
| 21.979 | 20.58948 | 18.21701 | 19.59941 | 3 | 3 | 3 | 5.5 | 5.5 | 5.5 | 51.976 | 21.149 | 82625000 | 3 | Sorting and assembly machinery component 50 homolog | SAMM50 |
| 18.79867 | 16.61536 | 18.19966 | 20.85179 | 4 | 4 | 4 | 12.4 | 12.4 | 12.4 | 51.547 | 29.338 | 32539000 | 3 | Armadillo repeat-containing protein 6 | ARMC6 |
| 19.10135 | 21.84399 | 18.18027 | 22.3431 | 3 | 3 | 3 | 9.3 | 9.3 | 9.3 | 52.598 | 25.825 | 102540000 | 6 | Nuclear pore complex protein Nup85 | NUP85 |
| 25.58322 | 16.9045 | 18.18025 | 19.50344 | 138 | 2 | 2 | 69 | 0.6 | 0.6 | 245.86 | 110.28 | 2342500000 | 9 |  | FLNA |
| 21.7944 | 23.04535 | 18.17486 | 23.87455 | 6 | 6 | 6 | 16.9 | 16.9 | 16.9 | 54.392 | 63.375 | 183750000 | 10 | ERO1-like protein alpha | ERO1L |
| 20.13195 | 18.48109 | 18.16854 | 19.80106 | 3 | 3 | 3 | 11.8 | 11.8 | 11.8 | 33.712 | 20.055 | 31609000 | 4 | Translation initiation factor eIF-2B subunit alpha | EIF2B1 |
| 18.35723 | 19.82865 | 18.16739 | 18.93119 | 15 | 1 | 1 | 27.8 | 3.9 | 3.9 | 44.348 | 7.5828 | 38266000 | 2 | Putative heat shock protein HSP 90-beta 2 | HSP90AB2P |
| 18.85898 | 22.47721 | 18.16727 | 19.93118 | 3 | 3 | 3 | 12.8 | 12.8 | 12.8 | 42.894 | 23.344 | 95862000 | 4 | Polymerase delta-interacting protein 3 | POLDIP3 |
| 22.59541 | 24.98498 | 18.16484 | 18.04485 | 13 | 13 | 13 | 17.7 | 17.7 | 17.7 | 114.71 | 195.13 | 491260000 | 18 | Ribonucleases P/MRP protein subunit POP1 | POP1 |
| 19.68298 | 16.5535 | 18.15033 | 19.13935 | 3 | 3 | 3 | 9.7 | 9.7 | 9.7 | 46.087 | 17.791 | 31170000 | 3 | Thymidine phosphorylase | TYMP |
| 19.43198 | 17.9694 | 18.1446 | 19.19494 | 3 | 3 | 3 | 2.8 | 2.8 | 2.8 | 148.53 | 17.764 | 15370000 | 3 | 1-phosphatidylinositol 4,5-bisphosphate phosphodiesterase gamma-1 | PLCG1 |
| 20.47912 | 18.43239 | 18.14005 | 18.37874 | 6 | 4 | 4 | 3.5 | 2.6 | 2.6 | 229.56 | 30.298 | 87501000 | 4 | Dedicator of cytokinesis protein 6 | DOCK6 |
| 22.47778 | 20.72717 | 18.13692 | 20.90896 | 6 | 5 | 5 | 11.5 | 9.9 | 9.9 | 68.04 | 44.197 | 116270000 | 4 | TRIO and F-actin-binding protein | TRIOBP |
| 20.72949 | 19.8198 | 18.12741 | 18.13261 | 5 | 5 | 4 | 13.3 | 13.3 | 9.8 | 62.293 | 31.787 | 150620000 | 6 | Dihydropyrimidinase-related protein 2 | DPYSL2 |
| 22.49916 | 21.12206 | 18.12633 | 20.58564 | 8 | 8 | 8 | 12.4 | 12.4 | 12.4 | 120.5 | 71.756 | 146080000 | 8 | Transcription elongation factor SPT5 | SUPT5H |
| 18.8166 | 20.44071 | 18.12152 | 17.58135 | 3 | 3 | 3 | 8.3 | 8.3 | 8.3 | 45.31 | 18.291 | 7500300 | 3 | Casein kinase II subunit alpha | CSNK2A1 |
| 23.8227 | 23.02715 | 18.11749 | 22.31205 | 16 | 8 | 8 | 25.1 | 13.1 | 13.1 | 91.2 | 152.37 | 361310000 | 13 | Scaffold attachment factor B1 | SAFB |
| 17.55848 | 19.06863 | 18.11396 | 18.98008 | 5 | 1 | 1 | 7.8 | 1.9 | 1.9 | 85.018 | 7.2181 | 59232000 | 1 | ATP-dependent 6-phosphofructokinase, liver type | PFKL |
| 25.31693 | 24.42098 | 18.09907 | 18.74759 | 26 | 6 | 6 | 16.7 | 5.2 | 5.2 | 180.29 | 42.095 | 1028400000 | 7 | Clathrin heavy chain 2 | CLTCL1 |
| 17.91467 | 18.42588 | 18.09873 | 20.01781 | 8 | 2 | 2 | 18.9 | 5.4 | 5.4 | 61.313 | 13.89 | 14893000 | 2 | Serine/threonine-protein phosphatase 2A 65 kDa regulatory subunit A beta isoform | PPP2R1B |
| 20.44637 | 17.72687 | 18.09579 | 19.65637 | 3 | 3 | 3 | 15.2 | 15.2 | 15.2 | 27.067 | 35.485 | 28005000 | 3 | E3 ubiquitin-protein ligase CHIP | STUB1 |
| 20.89178 | 18.22365 | 18.09538 | 21.28275 | 3 | 3 | 3 | 3.2 | 3.2 | 3.2 | 122.07 | 18.793 | 50910000 | 3 | TELO2-interacting protein 1 homolog | TTI1 |
| 22.29698 | 24.88143 | 18.09512 | 21.18846 | 17 | 17 | 17 | 15.2 | 15.2 | 15.2 | 160.25 | 135.65 | 235540000 | 21 | Probable ATP-dependent RNA helicase YTHDC2 | YTHDC2 |
| 18.1916 | 21.22488 | 18.07992 | 19.71943 | 3 | 3 | 3 | 4.5 | 4.5 | 4.5 | 73.953 | 18.771 | 58373000 | 4 | FAS-associated factor 1 | FAF1 |
| 21.81363 | 21.37692 | 18.05684 | 21.41787 | 4 | 4 | 4 | 8.4 | 8.4 | 8.4 | 62.043 | 25.711 | 93773000 | 5 | U4/U6.U5 tri-snRNP-associated protein 2 | USP39 |
| 26.19286 | 24.02893 | 18.04136 | 23.31921 | 33 | 33 | 33 | 30 | 30 | 30 | 157.18 | 323.31 | 1374200000 | 47 | Condensin complex subunit 1 | NCAPD2 |
| 17.96133 | 23.92778 | 18.03828 | 18.94115 | 3 | 3 | 3 | 10.3 | 10.3 | 10.3 | 32.461 | 33.457 | 74401000 | 5 | Syndecan-1 | SDC1 |
| 20.9667 | 20.98141 | 18.03784 | 18.41218 | 3 | 3 | 2 | 4 | 4 | 2.6 | 113.66 | 22.62 | 87327000 | 3 | E3 ubiquitin-protein ligase BRE1A | RNF20 |
| 21.77547 | 21.99607 | 18.03683 | 21.89811 | 5 | 5 | 5 | 12.6 | 12.6 | 12.6 | 51.091 | 34.099 | 97698000 | 9 | Tubulin gamma-2 chain;Tubulin gamma-1 chain | TUBG2;TUBG1 |
| 21.82376 | 20.79257 | 18.03658 | 20.17998 | 3 | 3 | 3 | 6.9 | 6.9 | 6.9 | 51.84 | 22.222 | 82356000 | 4 | U3 small nucleolar RNA-interacting protein 2 | RRP9 |
| 20.99673 | 22.57609 | 18.03306 | 19.46707 | 6 | 6 | 6 | 10.8 | 10.8 | 10.8 | 75.935 | 46.236 | 114290000 | 5 | Gamma-interferon-inducible protein 16 | IFI16 |
| 22.3787 | 22.48267 | 18.03045 | 19.05835 | 11 | 11 | 11 | 8.3 | 8.3 | 8.3 | 192.22 | 101.3 | 140720000 | 13 | Rapamycin-insensitive companion of mTOR | RICTOR |
| 21.31407 | 18.17124 | 18.02477 | 19.58838 | 4 | 4 | 4 | 20.1 | 20.1 | 20.1 | 39.724 | 43.952 | 53560000 | 4 | Alcohol dehydrogenase class-3 | ADH5 |
| 21.37698 | 24.73691 | 18.01276 | 20.81151 | 9 | 9 | 9 | 16.3 | 16.3 | 16.3 | 96.557 | 74.097 | 293190000 | 15 | pre-rRNA processing protein FTSJ3 | FTSJ3 |
| 21.42721 | 21.09648 | 18.00412 | 18.30766 | 3 | 3 | 3 | 14.8 | 14.8 | 14.8 | 20.9 | 18.033 | 11820000 | 3 | NEDD8-conjugating enzyme Ubc12 | UBE2M |
| 22.30944 | 21.36046 | 17.99952 | 23.52624 | 9 | 9 | 9 | 15.8 | 15.8 | 15.8 | 83.571 | 78.281 | 264890000 | 10 | Pyridoxal-dependent decarboxylase domain-containing protein 1 | PDXDC1 |
| 18.20803 | 18.02213 | 17.98398 | 21.67396 | 3 | 2 | 2 | 9.7 | 6.5 | 6.5 | 29.052 | 10.998 | 6974300 | 3 | Protein NipSnap homolog 2 | GBAS |
| 16.06483 | 21.90257 | 17.98165 | 18.57076 | 4 | 4 | 4 | 8.3 | 8.3 | 8.3 | 100.68 | 24.937 | 54156000 | 4 | General transcription factor 3C polypeptide 2 | GTF3C2 |
| 21.92955 | 20.19147 | 17.96916 | 22.33718 | 7 | 7 | 7 | 5.1 | 5.1 | 5.1 | 175.48 | 62.248 | 116720000 | 11 | Tetratricopeptide repeat protein 37 | TTC37 |
| 21.49838 | 20.90544 | 17.96857 | 19.23529 | 6 | 6 | 4 | 4.3 | 4.3 | 2.6 | 202.04 | 39.16 | 73278000 | 5 | Brefeldin A-inhibited guanine nucleotide-exchange protein 2 | ARFGEF2 |
| 26.84529 | 20.34722 | 17.96469 | 18.97717 | 33 | 6 | 0 | 81 | 15.3 | 0 | 28.747 | 99.559 | 2268000000 | 11 |  | TPM1 |
| 17.88264 | 22.17176 | 17.96046 | 23.12633 | 5 | 5 | 5 | 14.6 | 14.6 | 14.6 | 52.384 | 37.958 | 72781000 | 7 | Glutathione synthetase | GSS |
| 17.53159 | 22.94557 | 17.95926 | 19.41866 | 5 | 5 | 5 | 16.9 | 16.9 | 16.9 | 55.52 | 43.497 | 65997000 | 7 | Nicotinamide phosphoribosyltransferase | NAMPT;NAMPTL |
| 23.28757 | 24.51358 | 17.95791 | 21.70717 | 6 | 6 | 6 | 28.1 | 28.1 | 28.1 | 21.188 | 36.895 | 56957000 | 12 | Nucleolar protein 16 | NOP16 |
| 21.05112 | 15.98066 | 17.95158 | 19.68445 | 4 | 4 | 4 | 11.4 | 11.4 | 11.4 | 48.01 | 25.381 | 59310000 | 5 | Testin | TES |
| 19.57952 | 17.87594 | 17.94501 | 20.41389 | 3 | 3 | 3 | 2.6 | 2.6 | 2.6 | 166.67 | 17.535 | 18066000 | 3 | C-type mannose receptor 2 | MRC2 |
| 23.18929 | 17.24782 | 17.94293 | 18.36764 | 10 | 1 | 1 | 49.7 | 6.2 | 6.2 | 20.124 | 7.7127 | 65476000 | 6 | 60S ribosomal protein L11 | RPL11 |
| 20.81785 | 20.92187 | 17.93545 | 21.09088 | 3 | 3 | 3 | 9.5 | 9.5 | 9.5 | 40.16 | 19.021 | 66402000 | 6 | Drebrin-like protein | DBNL |
| 26.74034 | 24.48708 | 17.91676 | 23.21662 | 8 | 6 | 6 | 29.2 | 22.1 | 22.1 | 22.58 | 67.113 | 2000900000 | 17 | Histone H1.5 | HIST1H1B |
| 20.74862 | 17.92177 | 17.90685 | 19.10831 | 3 | 3 | 3 | 14.7 | 14.7 | 14.7 | 33.734 | 31.542 | 35825000 | 4 | Sororin | CDCA5 |
| 21.74968 | 17.20277 | 17.89534 | 19.88159 | 10 | 1 | 1 | 31.5 | 3.7 | 3.7 | 37.029 | 9.2774 | 80149000 | 3 | Heterogeneous nuclear ribonucleoprotein A3 | HNRNPA3 |
| 19.22021 | 21.58738 | 17.88975 | 19.59697 | 4 | 4 | 4 | 4.5 | 4.5 | 4.5 | 116.19 | 26.084 | 102690000 | 3 | AP-3 complex subunit beta-1 | AP3B1 |
| 23.34584 | 21.83457 | 17.88096 | 17.97193 | 15 | 15 | 15 | 16.1 | 16.1 | 16.1 | 119.2 | 124.09 | 228340000 | 14 | Sperm-specific antigen 2 | SSFA2 |
| 16.94571 | 21.4705 | 17.87558 | 19.29396 | 3 | 3 | 3 | 15.1 | 15.1 | 15.1 | 31.496 | 22.362 | 40280000 | 5 | Protein PRRC1 | PRRC1 |
| 21.62096 | 16.8252 | 17.83044 | 18.84967 | 4 | 4 | 4 | 7.2 | 7.2 | 7.2 | 101.82 | 29.412 | 78642000 | 4 | Signal-induced proliferation-associated protein 1 | SIPA1 |
| 18.71928 | 22.57466 | 17.82922 | 19.50065 | 3 | 3 | 3 | 12.8 | 12.8 | 12.8 | 25.917 | 18.465 | 126770000 | 5 | SWI/SNF-related matrix-associated actin-dependent regulator of chromatin subfamily B member 1 | SMARCB1 |
| 21.81045 | 22.51862 | 17.82529 | 19.21576 | 3 | 3 | 3 | 17.7 | 17.7 | 17.7 | 19.933 | 19.968 | 69459000 | 5 | Bifunctional methylenetetrahydrofolate dehydrogenase/cyclohydrolase, mitochondrial;NAD-dependent methylenetetrahydrofolate dehydrogenase;Methenyltetrahydrofolate cyclohydrolase | MTHFD2 |
| 23.39859 | 17.58066 | 17.82169 | 18.51834 | 5 | 5 | 5 | 10.6 | 10.6 | 10.6 | 60.103 | 46.456 | 219950000 | 8 | PC4 and SFRS1-interacting protein | PSIP1 |
| 22.0012 | 19.77431 | 17.81162 | 19.62337 | 3 | 3 | 3 | 9.8 | 9.8 | 9.8 | 35.282 | 20.278 | 11301000 | 3 | Growth hormone-inducible transmembrane protein | GHITM |
| 23.66817 | 23.84029 | 17.79085 | 19.29613 | 13 | 13 | 13 | 12.1 | 12.1 | 12.1 | 168.59 | 110.61 | 338640000 | 18 | Gem-associated protein 5 | GEMIN5 |
| 23.7349 | 21.39095 | 17.78346 | 19.55589 | 4 | 4 | 4 | 6.3 | 6.3 | 6.3 | 98.099 | 111.27 | 390110000 | 5 | Kinesin-like protein KIF20A | KIF20A |
| 22.72503 | 23.36506 | 17.76276 | 18.92839 | 4 | 4 | 4 | 29.9 | 29.9 | 29.9 | 16.648 | 23.922 | 51019000 | 5 | Prefoldin subunit 2 | PFDN2 |
| 20.64781 | 22.34015 | 17.75577 | 20.05785 | 5 | 5 | 5 | 3.5 | 3.5 | 3.5 | 211.33 | 28.651 | 47222000 | 4 | Proteasome activator complex subunit 4 | PSME4 |
| 21.13671 | 17.29872 | 17.75144 | 19.50524 | 5 | 5 | 5 | 4.4 | 4.4 | 4.4 | 140.53 | 28.016 | 41121000 | 5 | Methionine synthase | MTR |
| 22.51263 | 21.55098 | 17.74737 | 19.38047 | 3 | 3 | 3 | 14.6 | 14.6 | 14.6 | 23.63 | 17.855 | 31413000 | 4 | Peptidyl-tRNA hydrolase ICT1, mitochondrial | ICT1 |
| 21.40494 | 17.96317 | 17.74472 | 20.04082 | 11 | 1 | 1 | 47.4 | 3.3 | 3.3 | 34.95 | 7.6373 | 62070000 | 2 | Serine/threonine-protein phosphatase;Serine/threonine-protein phosphatase PP1-gamma catalytic subunit | PPP1CC |
| 20.26614 | 21.3681 | 17.7413 | 19.19803 | 4 | 4 | 4 | 19.3 | 19.3 | 19.3 | 34.994 | 41.771 | 43098000 | 6 | 2,4-dienoyl-CoA reductase, mitochondrial | DECR1 |
| 21.23847 | 21.84361 | 17.72701 | 19.35458 | 5 | 4 | 4 | 6.7 | 5.3 | 5.3 | 78.832 | 24.628 | 81101000 | 4 | Poly(A) RNA polymerase, mitochondrial | MTPAP |
| 22.15783 | 21.8811 | 17.70112 | 18.72969 | 4 | 4 | 4 | 4.8 | 4.8 | 4.8 | 122.03 | 30.748 | 182340000 | 4 | Nucleolar protein 6 | NOL6 |
| 23.03031 | 16.94404 | 17.69819 | 18.41618 | 8 | 8 | 8 | 9.4 | 9.4 | 9.4 | 134.41 | 59.806 | 150870000 | 8 | Pleckstrin homology domain-containing family G member 3 | PLEKHG3 |
| 22.43832 | 21.76219 | 17.68405 | 18.50931 | 8 | 8 | 8 | 8.2 | 8.2 | 8.2 | 133.06 | 59.732 | 124830000 | 7 | DNA-directed RNA polymerase;DNA-directed RNA polymerase II subunit RPB2 | POLR2B |
| 19.36459 | 18.83697 | 17.67677 | 19.01263 | 3 | 3 | 3 | 3.1 | 3.1 | 3.1 | 144.68 | 20.697 | 17590000 | 4 | C-Jun-amino-terminal kinase-interacting protein 4 | SPAG9 |
| 17.73402 | 21.74731 | 17.6747 | 19.29607 | 4 | 4 | 4 | 8.4 | 8.4 | 8.4 | 64.096 | 35.221 | 129180000 | 5 | Galectin-3-binding protein | LGALS3BP |
| 22.39846 | 21.80392 | 17.66735 | 19.70947 | 5 | 5 | 5 | 5.4 | 5.4 | 5.4 | 151.46 | 48.779 | 102890000 | 4 | Nuclear pore complex protein Nup153 | NUP153 |
| 22.02337 | 21.71733 | 17.66302 | 19.95202 | 5 | 5 | 5 | 5.7 | 5.7 | 5.7 | 120.97 | 50.109 | 100860000 | 5 | CCAAT/enhancer-binding protein zeta | CEBPZ |
| 17.61554 | 20.44121 | 17.66046 | 18.87591 | 3 | 3 | 3 | 12.9 | 12.9 | 12.9 | 35.978 | 17.941 | 21186000 | 3 | Sideroflexin-3 | SFXN3 |
| 18.23639 | 22.4148 | 17.65496 | 22.53488 | 6 | 6 | 6 | 18.1 | 18.1 | 18.1 | 50.576 | 37.483 | 58606000 | 7 | Programmed cell death protein 4 | PDCD4 |
| 25.0068 | 17.60111 | 17.63012 | 20.88439 | 10 | 1 | 1 | 24.3 | 7.2 | 7.2 | 49.775 | 102.44 | 971680000 | 4 | Tubulin beta-8 chain | TUBB8 |
| 18.63877 | 17.91374 | 17.5848 | 17.94954 | 3 | 1 | 1 | 3.3 | 1 | 1 | 142.53 | 7.9821 | 6866100 | 1 | Brefeldin A-inhibited guanine nucleotide-exchange protein 1 | ARFGEF1 |
| 20.92187 | 21.37342 | 17.57549 | 19.61771 | 3 | 3 | 3 | 2.3 | 2.3 | 2.3 | 182.36 | 17.69 | 41632000 | 5 | Nucleoporin NUP188 homolog | NUP188 |
| 21.67077 | 22.82466 | 17.57232 | 20.09871 | 6 | 6 | 6 | 11 | 11 | 11 | 69.922 | 47.785 | 154240000 | 7 | RNA-binding protein 28 | RBM28 |
| 21.02715 | 20.73057 | 17.55628 | 18.91648 | 5 | 3 | 3 | 5.5 | 4 | 4 | 148.74 | 26.914 | 43441000 | 2 | Sister chromatid cohesion protein PDS5 homolog B | PDS5B |
| 20.04727 | 17.9052 | 17.54259 | 19.61817 | 7 | 2 | 2 | 58 | 21.7 | 21.7 | 15.144 | 43.011 | 2977600 | 2 | Histone H2AX;Histone H2A type 1-A;Histone H2A type 2-B | H2AFX;HIST1H2AA;HIST2H2AB |
| 19.31052 | 20.00347 | 17.53965 | 19.93875 | 3 | 3 | 3 | 33.3 | 33.3 | 33.3 | 11.995 | 41.62 | 42823000 | 4 | UDP-glucose 4-epimerase | GALE |
| 22.12534 | 21.30458 | 17.53071 | 19.27639 | 7 | 7 | 4 | 8.3 | 8.3 | 5.8 | 134.07 | 50.998 | 103580000 | 7 | Cohesin subunit SA-2 | STAG2 |
| 17.33536 | 20.09055 | 17.51658 | 19.18371 | 4 | 2 | 2 | 7.5 | 4.4 | 4.4 | 63.86 | 12.472 | 58187000 | 3 | YTH domain-containing family protein 3 | YTHDF3 |
| 21.42552 | 18.17245 | 17.51472 | 21.67908 | 3 | 3 | 3 | 8 | 8 | 8 | 46.665 | 17.277 | 82211000 | 3 | Cleavage stimulation factor subunit 2 | CSTF2 |
| 21.23362 | 17.71297 | 17.51445 | 19.49334 | 34 | 2 | 2 | 34.5 | 2 | 2 | 135.19 | 26.676 | 41481000 | 2 |  |  |
| 17.81878 | 20.38669 | 17.50263 | 21.44965 | 3 | 3 | 3 | 11.9 | 11.9 | 11.9 | 37.72 | 17.878 | 38581000 | 3 | Nuclear distribution protein nudE homolog 1 | NDE1 |
| 21.08984 | 17.25987 | 17.47719 | 19.35976 | 8 | 8 | 7 | 4.9 | 4.9 | 4.4 | 215.04 | 67.662 | 166100000 | 5 | Unconventional myosin-Va | MYO5A |
| 21.88629 | 21.66219 | 17.45599 | 19.96467 | 3 | 3 | 3 | 7.8 | 7.8 | 7.8 | 71.866 | 51.621 | 143980000 | 4 | RNA polymerase II-associated protein 3 | RPAP3 |
| 19.94063 | 18.70512 | 17.4417 | 18.33639 | 4 | 4 | 4 | 23.3 | 23.3 | 23.3 | 23.944 | 24.969 | 25692000 | 4 | Protein FAM207A | FAM207A |
| 17.9122 | 20.44476 | 17.44118 | 20.00508 | 5 | 1 | 1 | 10 | 2.2 | 2.2 | 63.852 | 7.0285 | 92806000 | 2 | Ubiquilin-4 | UBQLN4;UBQLN1 |
| 21.60318 | 22.12795 | 17.42964 | 18.7813 | 5 | 5 | 5 | 5.9 | 5.9 | 5.9 | 111.49 | 28.257 | 144220000 | 4 | Rho guanine nucleotide exchange factor 2 | ARHGEF2 |
| 22.88731 | 19.03754 | 17.42758 | 19.54229 | 3 | 2 | 2 | 17.8 | 13.3 | 13.3 | 20.589 | 19.948 | 171430000 | 5 | Transformer-2 protein homolog alpha | TRA2A |
| 20.74148 | 23.03344 | 17.40754 | 21.8451 | 12 | 12 | 9 | 6 | 6 | 4.9 | 358.2 | 103.05 | 231060000 | 13 | E3 SUMO-protein ligase RanBP2 | RANBP2 |
| 22.85696 | 18.01789 | 17.39416 | 19.62952 | 3 | 3 | 3 | 6.5 | 6.5 | 6.5 | 72.75 | 17.897 | 189920000 | 2 | Beta-galactosidase | GLB1 |
| 21.177 | 21.8482 | 17.37572 | 19.49207 | 5 | 5 | 5 | 3.9 | 3.9 | 3.9 | 165.27 | 30.471 | 59034000 | 5 | Peroxidasin homolog | PXDN |
| 21.89818 | 18.36831 | 17.34753 | 19.77592 | 5 | 5 | 5 | 26.6 | 26.6 | 26.6 | 33.172 | 66.996 | 77518000 | 5 | Nitric oxide synthase-interacting protein | NOSIP |
| 20.02918 | 22.59302 | 17.34354 | 19.68596 | 6 | 6 | 6 | 6.4 | 6.4 | 6.4 | 119.38 | 37.605 | 33222000 | 4 | Zinc finger and BTB domain-containing protein 11 | ZBTB11 |
| 18.48734 | 20.64209 | 17.32559 | 20.57191 | 3 | 3 | 3 | 7.6 | 7.6 | 7.6 | 52.466 | 17.412 | 15319000 | 3 | Phosphoenolpyruvate carboxykinase [GTP], mitochondrial | PCK2 |
| 22.41918 | 17.69706 | 17.3223 | 17.35395 | 21 | 1 | 0 | 70.2 | 6.8 | 0 | 34.255 | 13.027 | 263050000 | 3 |  | LMO7 |
| 21.31877 | 17.13508 | 17.31983 | 21.54165 | 13 | 9 | 9 | 2.2 | 1.6 | 1.6 | 856.87 | 69.99 | 185500000 | 9 | Microtubule-actin cross-linking factor 1, isoforms 1/2/3/5 | MACF1 |
| 26.22617 | 23.42458 | 17.30899 | 22.92546 | 35 | 33 | 33 | 27.9 | 26.9 | 26.9 | 180.58 | 323.31 | 1450900000 | 34 | Ras GTPase-activating-like protein IQGAP2 | IQGAP2 |
| 17.31164 | 17.14482 | 17.28437 | 21.69541 | 3 | 3 | 3 | 8.7 | 8.7 | 8.7 | 52.175 | 19.204 | 24133000 | 3 | 2,5-phosphodiesterase 12 | PDE12 |
| 21.55848 | 22.61183 | 17.26261 | 19.3917 | 8 | 8 | 8 | 5.6 | 5.6 | 5.6 | 170.54 | 51.423 | 69253000 | 10 | Unhealthy ribosome biogenesis protein 2 homolog | URB2 |
| 20.77161 | 22.41784 | 17.2221 | 21.86895 | 5 | 5 | 5 | 4.8 | 4.8 | 4.8 | 162.46 | 31.44 | 56952000 | 6 | Early endosome antigen 1 | EEA1 |
| 21.69298 | 21.69515 | 17.21244 | 19.7594 | 7 | 7 | 7 | 8.2 | 8.2 | 8.2 | 113.67 | 45.919 | 106700000 | 7 | Putative helicase MOV-10 | MOV10 |
| 18.71466 | 21.9226 | 17.17758 | 22.8771 | 5 | 5 | 5 | 7.4 | 7.4 | 7.4 | 106.62 | 44.777 | 142250000 | 9 | Protein transport protein Sec24C | SEC24C |
| 22.09081 | 21.48094 | 16.87905 | 19.45386 | 4 | 4 | 4 | 9.6 | 9.6 | 9.6 | 78.549 | 28.907 | 153330000 | 5 | Pentatricopeptide repeat domain-containing protein 3, mitochondrial | PTCD3 |
| 23.38337 | 21.7268 | 16.79832 | 18.92126 | 15 | 6 | 6 | 19.4 | 9.5 | 9.5 | 103.96 | 106.59 | 339850000 | 6 | AP-2 complex subunit alpha-2 | AP2A2 |
| 23.46451 | 24.20135 | 16.77905 | 20.50724 | 8 | 8 | 8 | 4.3 | 4.3 | 4.3 | 245.44 | 60.511 | 413160000 | 15 | Microtubule-associated protein;Microtubule-associated protein 4 | MAP4 |
| 22.43194 | 17.99823 | 16.55268 | 18.74433 | 142 | 3 | 3 | 66.5 | 1.8 | 1.8 | 251.39 | 97.392 | 301890000 | 4 | Spectrin beta chain, non-erythrocytic 1 | SPTBN1 |
