# Supplementary material for: CDK1-mediated phosphorylation at H2B serine 6 is required for mitotic chromosome segregation
Source: J Cell Biol. 2019 Feb 14;218(4):1164–81. doi: 10.1083/jcb.201806057 (PMC6446833; doi:10.1083/jcb.201806057)
Supplement: Tables S1-S3 (ZIP) [file JCB_201806057_TablesS1-S3.zip › JCB_201806057_TableS3.doc]

**Supplementary Information**

**Supplementary Table 3**

**Antibodies**

| **Primary antibody (clone)** | **Species** | **Supplier** |
| --- | --- | --- |
| anti-14-3-3 | rabbit pAb | Cell signaling |
| anti-acetyl-lysine | rabbit pAb | Cell signaling |
| anti-Aurora B (mAbcam3609) | mouse mAb | Abcam |
| anti-CDK1 (POH1) | mouse mAb | Cell signaling |
| anti-CENP-A (3-19) | mouse mAb | Abcam |
| anti-CENP-C | goat pAb | MBL International |
| anti-Cyclin A (BF683) | mouse mAb | Santa Cruz |
| anti-Cyclin B1 (D-11) | mouse mAb | Santa Cruz |
| anti-Cyclin B1 (GNS11) | mouse mAb | ThermoFisher |
| anti-Flag (M2) | mouse mAb | Sigma |
| anti-GFP (7.1 and 13.1) | mouse mAbs | Roche |
| anti-H2A.X S139ph (20E3) | rabbit mAb | Cell Signaling |
| anti-H2B | rabbit pAb | Abcam |
| anti-H2B S6ph | rabbit pAb | PolyPeptide |
| anti-H3 K9me2/3 (6F12) | mouse mAb | Cell Signaling |
| anti-H3 S10ph (6G3) | mouse mAb | Cell Signaling |
| anti-H3 T3ph (16B2) | mouse mAb | Hiroshi Kimura |
| anti-H3 T3ph (D5G1I) | rabbit mAb | Cell Signaling |
| anti-HA (3F10) | rat mAb | Roche |
| anti-Myc (9E10) | mouse mAb | Sigma |
| anti-p21 (F5) | mouse mAb | Santa Cruz |
| anti-PARP (C-2-10) | mouse mAb | Sigma |
| anti-RAD21 (53A303) | mouse mAb | Millipore |
| anti-SET (F-9) | mouse mAb | Santa Cruz |
| anti-α-Tubulin (12G10) | mouse mAb | DSHB |
| anti-β-Actin | rabbit pAb | Abcam |
| IgG control | mouse pAb | Santa Cruz |
| IgG control | rabbit pAb | Santa Cruz |

| **Secondary antibody** | **Conjugated to** | **Supplier** |
| --- | --- | --- |
| goat-anti-mouse IgG | Alexa Fluor 488 | Dianova |
| goat-anti-mouse IgG | Cy3 | Dianova |
| goat-anti-mouse IgG | HRP | Dianova |
| goat-anti-rabbit IgG | Cy3 | Dianova |
| goat-anti-rabbit IgG | HRP | Dianova |
| goat-anti-rat IgG | HRP | Dianova |

**Plasmids**

| **Plasmid** | **Origin (reference)** | **Insert** | **Vector** | **Cloning/PCR** |
| --- | --- | --- | --- | --- |
| Cyclin B1-Venus | J. Pines, Cambridge (PMID:21336306) | cyclin B1 (NM_031966.3) | pVenus-N1 | cDNA was inserted into vector opened with HindIII & BamHI |
| Cyclin B1-Venus ND | this study | cyclin B1 R42A/L45A | pVenus-N1 | mutagenesis PCR with Cyclin-B1-ND-fw & Cyclin-B1-ND-rv |
| Flag-CDK1 AF | Y. Maehara, Fukuoka (PMID: 27030108) | CDK1 T14A/Y15F | pcDNA3.1 |  |
| Flag-CDK1 KD | Y. Maehara, Fukuoka (PMID: 27030108) | CDK1 D145N | pcDNA3.1 |  |
| GFP | Clontech | GFP | pEGFP-C1 |  |
| GFP-BubR1 | this study | BubR1 (NM_001211.5) | pEGFP-C1 | cDNA was amplified with BUB1B-KpnI-fw & BUB1B-BamHI-rv and inserted into vector opened with KpnI & BamHI |
| GFP-lacI | R. Renkawitz, Giessen (PMID: 25294833) | GFP-lacI | pcDNA3.1 |  |
| GFP-lacI-cyclin B1 ND | this study | cyclin B1 R42A/L45A | pcDNA3.1-GFP-lacI | cyclin B1 ND was excised with BglII & XbaI and inserted into vector opened with BamHI and XbaI |
| GFP-SET WT | Y. Nishimura, Tokyo (PMID: 23251465) | SETβ (NM_003011.3) | pAcGFP-C1 |  |
| GFP-SET ΔC | this study | SETβ 1-218 | pAcGFP-C1 | deletion of C terminal fragment by digestion of GFP-SET with ScaI & SmaI and re-ligation |
| GST | GE Healthcare | GST | pGEX-4T-1 |  |
| H2B-GST | J.M.G. Higgins, Newcastle (PMID: 15681610) | H2B 1-35 (NM_003526.2) | pETGEX-CT | H2B cDNA inserted into vector opened with NcoI |
| H2B S6A-GST | this study | H2B S6A 1-35 | pETGEX-CT | mutagenesis PCR with H2B-S6A-fw & H2B-S6A-rv |
| H2B S6E-GST | this study | H2B S6E 1-35 | pETGEX-CT | mutagenesis PCR with H2B-S6E-fw & H2B-S6E-rv |
| H2B-GFP | Addgene (PMID: 9545195) | H2B (NM_021058.3) | pEGFP-N1 | H2B cDNA inserted into vector opened with KpnI & BamHI |
| H2B S6A-GFP | this study | H2B S6A | pEGFP-N1 | mutagenesis PCR with H2B-S6A-fw & H2B-S6A-rv |
| H2B S6E-GFP | this study | H2B S6E | pEGFP-N1 | mutagenesis PCR with H2B-S6E-fw & H2B-S6E-rv |
| HA-Aurora B WT | T. Stiewe, Marburg | Aurora B (NM_001313951.1) | pCMV6-HA | Aurora B cDNA from donor plasmid was inserted into vector by Gateway cloning |
| HA-Aurora B KD | T. Stiewe, Marburg | Aurora B K65R | pCMV6-HA | Aurora B cDNA from donor plasmid was inserted into vector by Gateway cloning |
| His-SET | M. A. De la Rosa, Sevilla (PMID: 26216969) | SETβ (NM_003011.3) | pET28a(+) | SET cDNA inserted into vector opened with NdeI & XhoI |
| Myc-INCENP | L. Yu-Lee, Houston (PMID: 22751009) | INCENP (NM_001040694.2) | pcDNA3 |  |
| pTet-on-GFP-SETβ | this study | SETβ (NM_003011.3) | pTet-on-Puro | GFP-SET was inserted into vector opened with AgeI & NotI |
| pX459-SET-con-sgRNA | this study | SET-con-sgRNA | pX459 | sgRNA oligos were annealed and ligated into vector opened with BbsI |
| pX459-SET-KO-sgRNA | this study | SET-KO-sgRNA | pX459 | sgRNA oligos were annealed and ligated into vector opened with BbsI |
|  |  |  |  |  |

DNA Oligonucleotides

| **Oligo name** | **Sequence (5´ to 3´)** |
| --- | --- |
| ACTB-qPCR-fw | CATGTACGTTGCTATCCAGGC |
| ACTB-qPCR-rv | CTCCTTAATGTCACGCACGAT |
| BUB1B-KpnI-fw | GAAGGTACCATGGCGGCGGTGAAGAAGGAAG |
| BUB1B-BamHI-rv | GTGAGACTTGATTGCCTAGGATCCTGAAAGAGC |
| Cyclin-B1-ND-fw | GGACTGAGGCCAGCAACAGCTGCTGGGGACATTGGTAACAAAG |
| Cyclin-B1-ND-rv | CAATGTCCCCAGCAGCTGTTGCTGGCCTCAGTCCGGGC |
| H2B-S6A-fw | CAGCGAAGGCTGCTCCCGCCCCGAAAAAG |
| H2B-S6A-rv | CGGGGCGGGAGCAGCCTTCGCTGGCTC |
| H2B-S6E-fw | GAGCCAGCGAAGGAGGCTCCCGCCCCGAAAAAGGGC |
| H2B-S6E-rv | CTTTTTCGGGGCGGGAGCCTCCTTCGCTGGCTCTGGC |
| PPP1CA-qPCR-fw | GAGACCATCTGCCTGCTGCT |
| PPP1CA-qPCR-rv | CAGTTTGATGTTGTAGCGTCTCTTG |
| PPP1CB-qPCR-fw | CGAGTTTGATAATGCTGGTGGAATG |
| PPP1CB-qPCR-rv | GCTGTTCGAGTTGGAGTGAC |
| PPP1CC-qPCR-fw | AAAGAGGCAGTTGGTCACTCTG |
| PPP1CC-qPCR-rv | TTACAGGTCTCGTGGCATTTGG |
| SET-con-sgRNA-fw | CACCGTTATTTAGAAATCGCGCTTG |
| SET-con-sgRNA-rv | AAACCAAGCGCGATTTCTAAATAAC |
| SET-KO-sgRNA-fw | CACCGACATTTGTCAACCATCCACA |
| SET-KO-sgRNA-rv | AAACTGTGGATGGTTGACAAATGTC |

**RNA Oligonucleotides**

| **Oligo name** | **Sequence (5´ to 3´)** |
| --- | --- |
| siPP1alpha | CCGCAUCUAUGGUUUCUAC[dT][dT] |
| siPP1alpha_as | GUAGAAACCAUAGAUGCGG[dT][dT] |
| siPP1beta | UUAUGAGACCUACUGAUGU[dT][dT] |
| siPP1beta_as | ACAUCAGUAGGUCUCAUAA[dT][dT] |
| siPP1gamma | GCAUGAUUUGGAUCUUAUA[dT][dT] |
| siPP1gamma_as | UAUAAGAUCCAAAUCAUGC[dT][dT] |

**Reagents**

| **Name** | **Working conc.** | **Target** | **Source** |
| --- | --- | --- | --- |
| Aphidicolin | 1 µg/ml | DNA polymerase α | Fluka |
| AZD1152 | 1 µM | Aurora B | Sigma |
| Benzonase | 25 U/ml | DNA & RNA | Millipore |
| BI 2536 | 1 µM | Plk1 | Santa Cruz |
| Calyculin A | 10 nM | PP1/PP2A phosphatases | Cell Signaling |
| Camptothecin | 1 µM | type I topoisomerases | Sigma |
| Doxorubicin | 0.5 µg/ml | DNA intercalation | Sigma |
| Etoposide | 1 µM | type II topoisomerases | Biotrend |
| Lambda Protein Phosphatase | 400 units | phosphorylated substrates | NEB |
| LB-100 | 10 µM | PP2A phosphatases | Selleckchem |
| LDN192960 | 10 µM | Haspin | Sigma |
| Lipopolysaccharide | 1 µg/ml | TLR4 agonist | Sigma |
| MG132 | 10 µM | proteasome | Sigma |
| Nocodazole | 100 ng/ml | microtubule polymerization | Sigma |
| Okadaic acid | 1 µM | PP1/PP2A phosphatases | Cell Signaling |
| PP1α | 0.6 units | phosphorylated substrates | Novus Biologicals |
| RO-3306 | 10 µM | CDK1-cyclin B1 | Sigma |
| Taxol/Paclitaxel | 100 nM | microtubule depolymerization | Cell Signaling |
| Thymidine | 2 mM | nucleotide synthesis | Sigma |
| Trichostatin A | 1 µM | class I & II HDACs | Sigma |

**Peptides**

| **Name** | **Sequence (N to C-terminal)** | **Source** |
| --- | --- | --- |
| C-PEPAK | CPEPAKSAPAPKKGSK | Peptide Specialty Laboratories (PSL) |
| H2B(1-20) unmodified | PEPAKSAPAPKKGSKKAVTK[Biotin] | Peptide Specialty Laboratories (PSL) |
| H2B(1-20)  S6ph | PEPAK(Sph)APAPKKGSKKAVTK[Biotin] | Peptide Specialty Laboratories (PSL) |
| H2B(1-20) K5meS6ph | PEPA(Kme)(Sph)APAPKKGSKKAVTK[Biotin] | Peptide Specialty Laboratories (PSL) |
| H2B(1-20) K5acS6ph | PEPA(Kac)(Sph)APAPKKGSKKAVTK[Biotin] | Peptide Specialty Laboratories (PSL) |
| SP120160 | PEPAK(Sph)APAPKKGSKC | PolyPeptide |
